# Functionalization of λ^5^-Phosphinines via metalation strategies

**DOI:** 10.1038/s42004-025-01822-6

**Published:** 2025-12-22

**Authors:** Flavie Rambaud, Bertrand Takam Fotie, Robert Naumann, Katja Heinze, Dorian Didier

**Affiliations:** 1 Technische Universität Darmstadt, Clemens-Schöpf-Institut, Darmstadt, Germany; 2 Johannes Gutenberg Universität Mainz, Department of Chemistry, Mainz, Germany

**Keywords:** Synthetic chemistry methodology, Single-molecule fluorescence, Organometallic chemistry

## Abstract

Phosphinines, or phosphabenzenes, exhibit distinctive electronic properties yet remain underexplored due to the challenges associated with their selective functionalization. We present herein the straightforward functionalization of λ^5^-phosphinine derivatives using organometallic strategies. Halogen-zinc and -magnesium exchanges were successfully performed employing Et_2_Zn·2O*amyl* or *i*-PrMgCl·LiCl species under smooth reaction conditions. Such method allowed access to a wide range of sophisticated architectures, photophysical studies of which demonstrated interesting fluorescence properties. With the possibility of using such fluorescence in biomarking, λ^5^-phosphinines were grafted on a few glycosides, nucleosides and pharmaceutically relevant moieties.

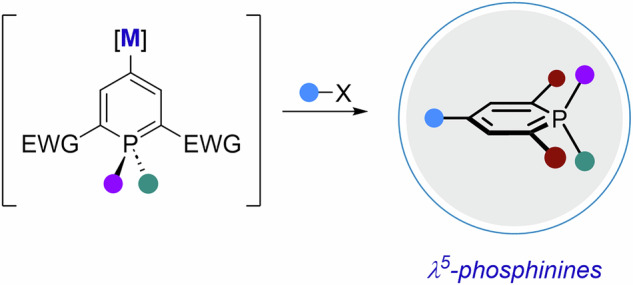

## Abstract (web summary)

Despite their intriguing electronic features and promising functional properties, phosphinines remain underexplored because their selective modification is difficult. In this work, the authors demonstrate that halogen–zinc exchanges provide an efficient route to functionalized λ⁵-phosphinines, operating under mild, functional-group-tolerant conditions and enabling access to diverse architectures with notable fluorescence characteristics.

## Introduction

The concept of *“escaping the flatland”* that refers to moving beyond simple, planar (two-dimensional) structures allows to explore the rich possibilities of three-dimensional molecular architectures^1^. Many organic compounds containing aromatic rings and conjugated systems are depicted as flat, which can limit their properties and interactions due to a restrained variety of exit vectors (orientation of substituents). However, introducing sp³-hybridized atoms, and non-planar ring systems allows chemists to access greater molecular complexity, which often leads to improved biological activity, selectivity, and physicochemical properties in drug design^2–4^. In this regard, we have developed methodologies towards the functionalization of four-membered molecular building blocks such as cyclobutanes^5–8^ and azetidines^9–14^, strained, three-dimensional sp^3^-rich scaffolds that could be used as 3D-surrogates for classical aromatic structures such as phenyl- or pyridyl-moieties.

If sp³-centers possess the advantage of allowing additional exit vectors to be placed in a three-dimensional fashion, escaping flatland usually comes at the cost of reduced conjugation. With the simple, yet challenging idea to explore 3D-structures that retain elements of electronic delocalization, we set out to study the underexplored functionalization of λ⁵-phosphinines.

Although pyridines are fundamental to heteroaromatic chemistry, phosphinines, also known as phosphabenzenes, have a relatively brief history. Nevertheless, molecular characteristics towards their application in–for example—homogeneous catalysis and coordination chemistry have sparked substantial research interest in recent decades, especially concerning their synthesis and functionalization.

**Scheme 1:**
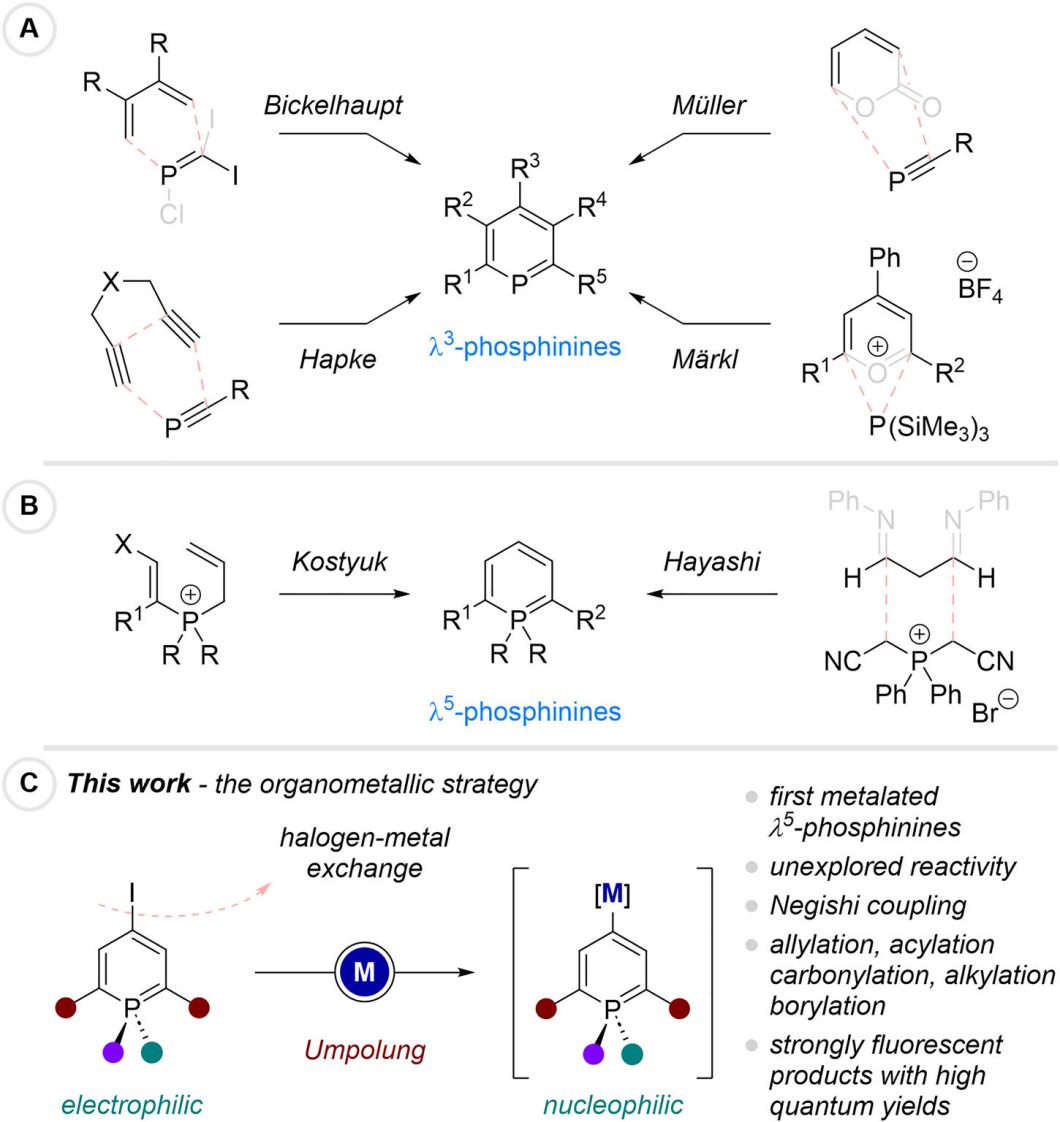
Synthesis of λ^3^- and λ^5^-phosphinines – State of the art and present work. **A** Literature-known access to λ^3^-phosphinines; **B** Literature-known access to λ^5^-phosphinines; **C** Our approach: Access to nucleophilic λ^5^-phosphinines via halogen-metal exchanges.

**Scheme 2:**
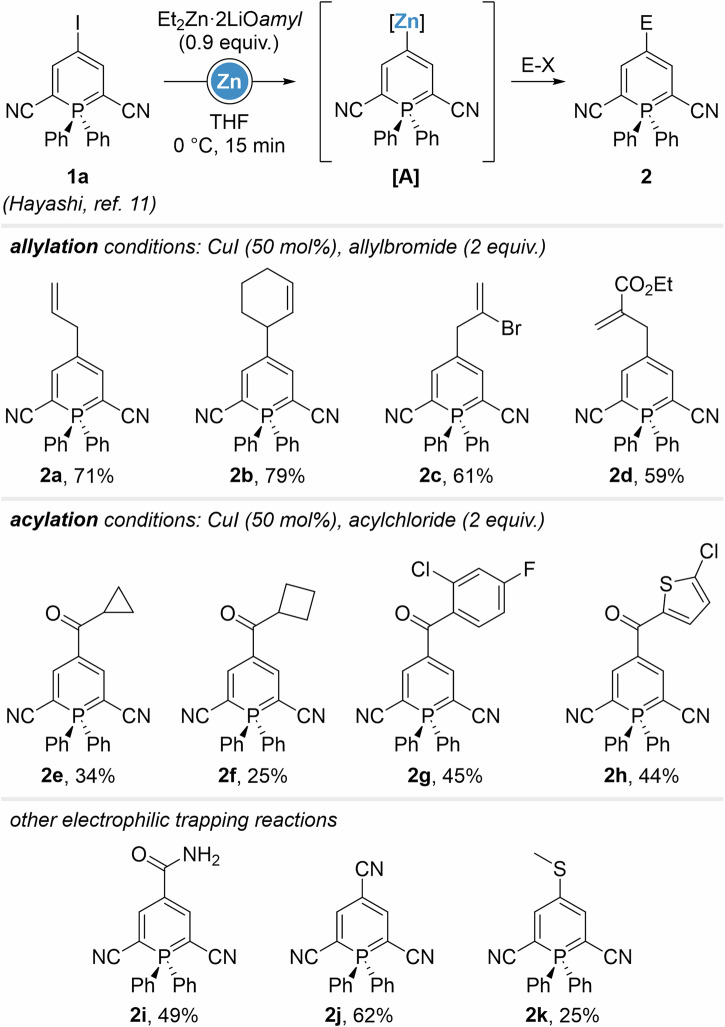
Scope of the iodine-zinc exchange/electrophilic trapping sequence on 2,6-dicyanophosphinines.

A few methods have proven efficient towards the generation of λ^3^-phosphinines (Scheme 1A). Early on, the group of Bickelhaupt showed that adequately substituted methylene-phosphane equivalents could be engaged in [4 + 2]-cycloadditions followed by eliminations^15^. Such cycloaddition strategies were employed by other groups, including impressive work from Müller ([4 + 2]/CO_2_-extrusion)^16^ and Hapke (Co-catalyzed [2 + 2 + 2])^17^. Interestingly, building upon pioneering work from Märkl^18^, pyrilium-derivatives successfully led to target structures in the presence of nucleophilic phosphorus sources^19,20^. While a few methods towards λ^3^-phosphinines are available, strategies for the synthesis of their λ^5^ 3D-analogs are scarcer (Scheme 1B). The most prominent examples were described by the groups of Kostyuk^21^ and Hayashi^22–24^, and rely on electrocyclic ring closure and condensation reactions. The group of Hayashi also demonstrated the possibility of using 4-iodo-derivatives such as **1a** (Scheme 2) as cross-coupling partner for Suzuki and Sonogashira reactions.

## Results and discussion

We envision that the creation of new phosphininyl-metal species would open the possibility of introducing a much larger range of functional groups through electrophilic trapping or Negishi coupling reactions (Scheme 1C). Our study started with 4-iodophoshinine **1a** (The utilization of 4-bromophosphinines in place of 4-iodophosphinines could, in principle, be envisaged. However, DEZA exhibits high selectivity toward iodide–zinc exchange and reacts with organobromides only to a negligible extent. Consequently, the reaction scope is restricted to the use of *i*PrMgCl·LiCl, which, as demonstrated herein, displays inferior performance relative to DEZA). (substrate recently introduced by the group of Hayashi and accessed by halogenation of the corresponding λ^5^-phosphinine, Scheme 1B)^22–24^ and first halogen-metal exchanges were optimized towards the formation of 4-phosphininylzinc species, as we initially thought that metals with lower electronegativity than zinc (Mg or Li) could interfere with the stability of phosphinines.

Optimizations (See Supplementary Information) were carried out employing our newly discovered reagent (DEZA = Et_2_Zn·2O*amyl*) for the generation of intermediate **A** (Scheme 2)^25^. The exchange reaction proceeded smoothly at 0 °C over a short period of time (15 min).

Electrophilic trapping reactions were first evaluated with allylbromides in the presence of copper salt additives, providing structures **2a**-**d** in good yields (up to 79%). Acyl chlorides were tested next under similar reaction conditions, leading to 4-carbonylated products **2e**-**h** in somewhat lower yields up to 45%. Introduction of a free amide was performed via addition of trichloroacetylisocyanate (**2i**) and a cyano group was placed using *p*-toluenesulfonylcyanide (**2j**). Although 2k was only isolated in 25%, chalcogenation proved successful using a disulfide reagent.

**Scheme 3:**
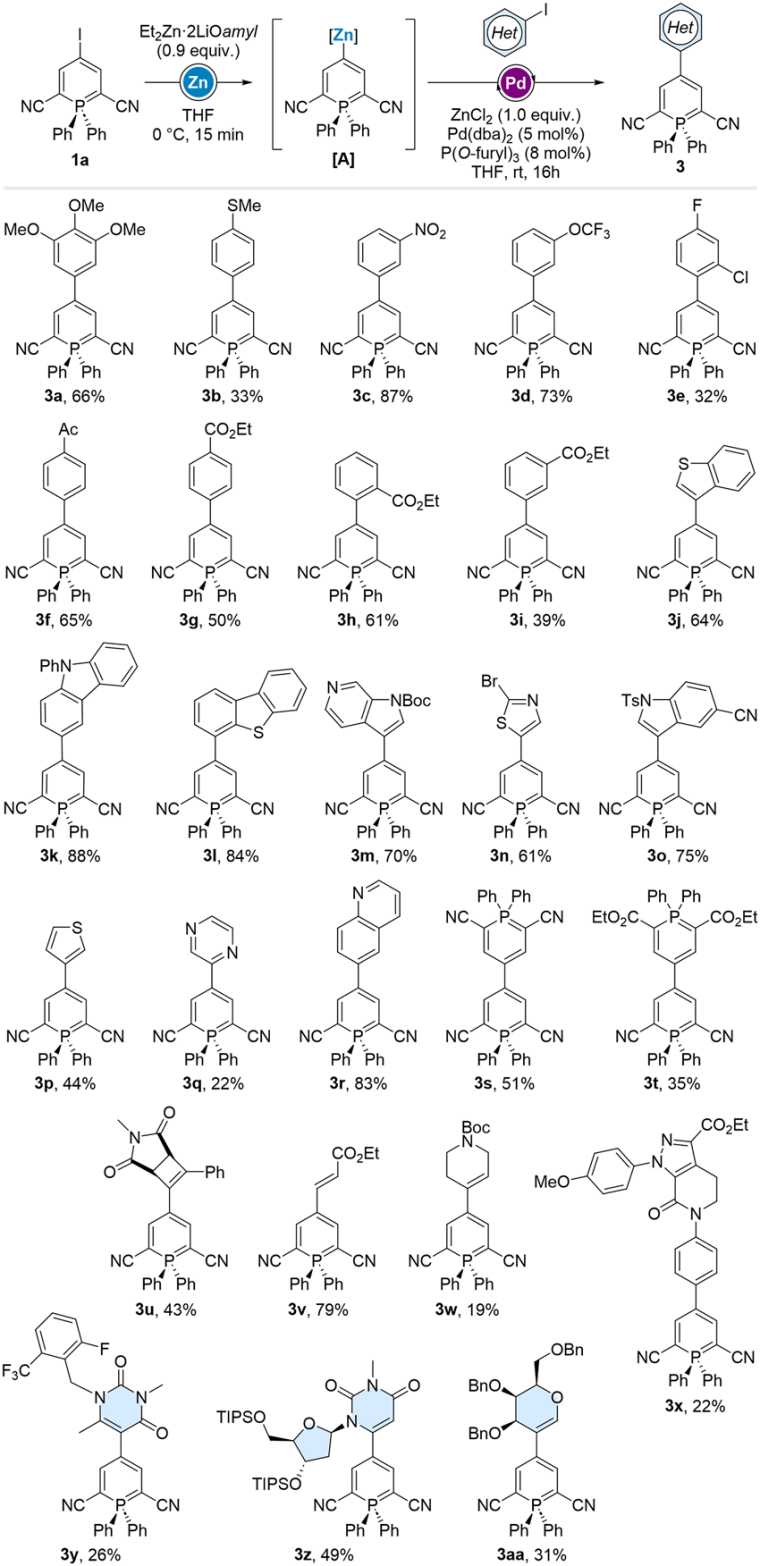
Scope of the iodine-zinc exchange/Negishi coupling sequence on 2,6-dicyanophosphinines.

4-Phosphininylzinc intermediates were engaged in Pd-catalyzed Negishi coupling next (Scheme 3), proceeding through halogen-zinc exchanges under previously described conditions. Short screening showed Pd(dba)_2_/P(*O*-furyl) to be the optimal catalytic system for such substrates, and ZnCl_2_ was preventively added to avoid potential homocoupling reactions^26^. Aryl iodides possessing electron-donating and withdrawing moieties were employed as cross-coupling partners, providing a first row of examples and demonstrating the great tolerance of the method towards functional groups, including nitro-groups, ketones and esters. 4-Arylphosphinines **3a**-**i** were isolated in 32 to 87% yield. A broad selection of heteroaryliodide performed equally well as coupling partners, furnishing **3j**-**r** in generally good yields (up to 88%), with the exception of 1,4-diaziryl-derivatives **3q** (22%). Worthy of note, unique biphosphinines **3s**-**t** were obtained employing 4-iodophosphinines **1a** and **1b** (vide infra) as Negishi coupling partners. Cyclobutenyl-compound **3 u** was synthesized in 43% yield using the corresponding iodide, as well as other alkenyl-derivative, with yields varying from 19 to 79% (**3w** and **3 v**, respectively). Interestingly, we were able to isolate the phosphininyl-conjugates **3x-y** of pharmaceutically-relevant moieties present in Apaxiban^27^ and Elagolix^28^, as well as nucleoside- and galactal-derivatives **3z** and **3aa**.

Organozinc reagents exhibit relatively high tolerance toward functional groups; however, this comes at the cost of reduced reactivity, making the synthesis of aldehydes, carboxylic acids, alkanols, and related organoboron compounds challenging. To overcome this issue, we set out to access more nucleophilic phosphininyl-magnesium species.

**Scheme 4:**
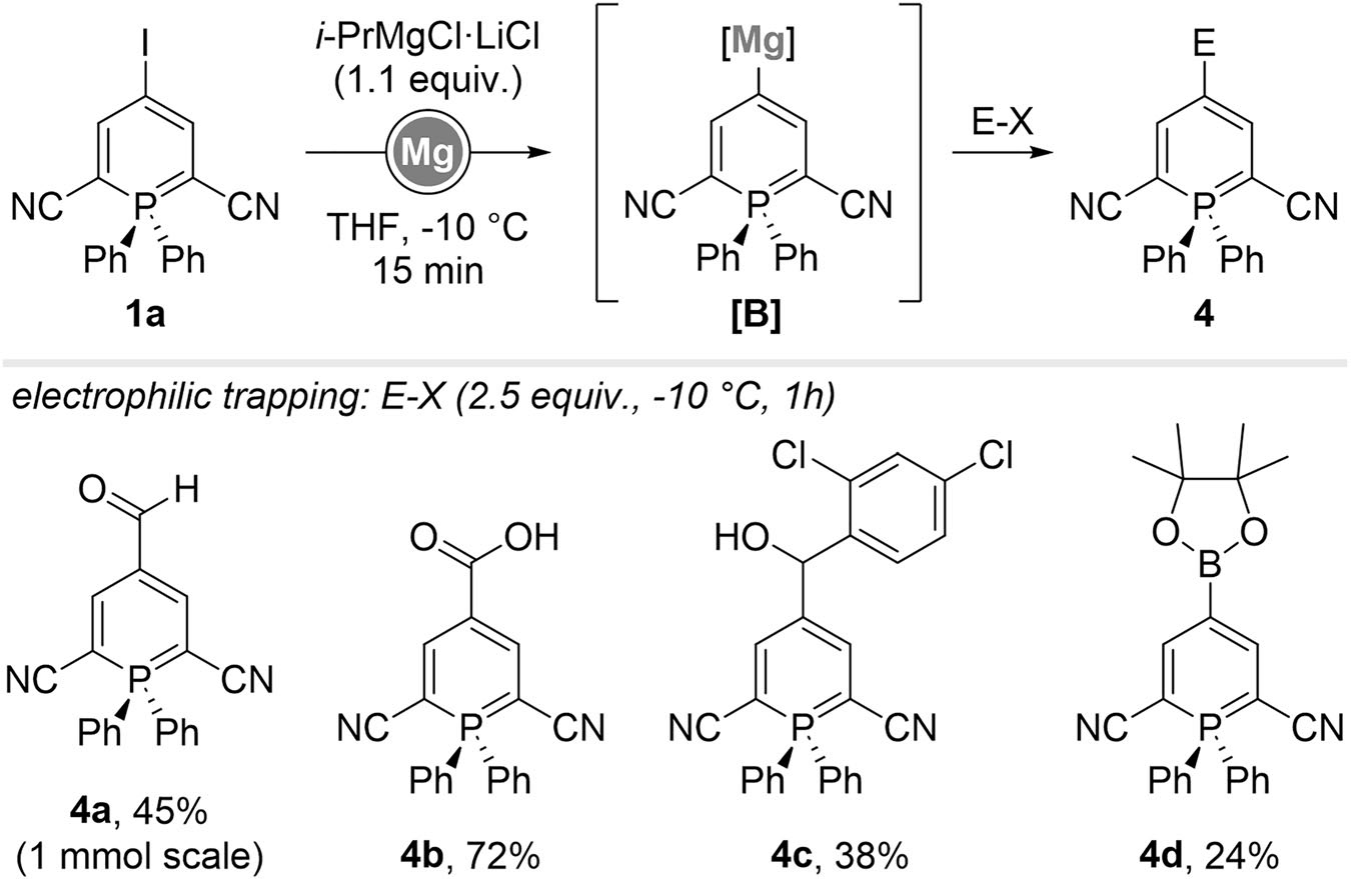
Scope of the iodine-magnesium exchange/electrophilic trapping sequence on 2,6-dicyanophosphinines.

While direct insertion with Mg(0) at room temperature only led to degradation of **1a**, iodine-magnesium exchange with *i*-PrMgCl·LiCl proved reliable towards the intermediate generation of **[B]** at −10 °C (Scheme 4). Subsequent nucleophilic attack onto DMF, CO_2_ or an aromatic aldehyde provided **4a**-**c** in up to 72% yield. A significantly lower yield was obtained when trapping the intermediate organomagnesium species **[B]** with (*i*-PrO)Bpin for the formation of **4 d** (24%), which we attributed to partial degradation during purification.

**Scheme 5:**
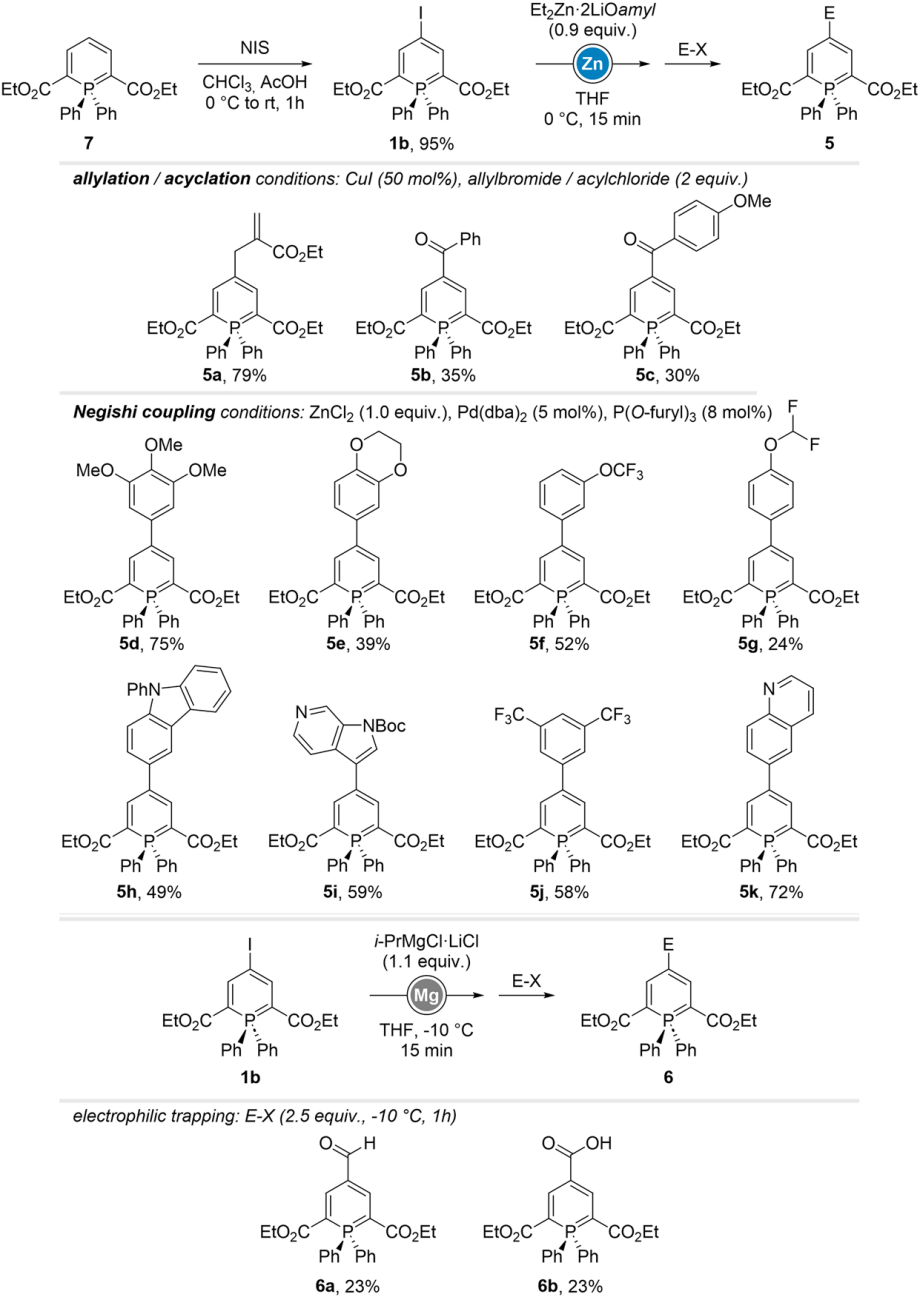
Scope of the iodine-metal exchange and further derivatization on 2,6-dicarboxyethylphosphinines.

In order to vary the substitution pattern around the phosphinine core and generalize the metalation strategy, we synthesized new structures possessing esters at position 2 and 6 (**1b**, 95%. Scheme 5), from the corresponding non-halogenated parent structure **7**.

Halogen-zinc exchanges were achieved once again with DEZA, and although those conditions proved tolerant to the presence of ethyl ester groups, products of electrophilic trapping and cross-coupling reactions were tendentially obtained with lower yields. While allylation provided **5a** with a highest yield of 79%, acylations gave **5b**-**c** in up to 35% yield. Negishi couplings using previously described Pd(dba)_2_/P(*O*-furyl)_3_ furnished **5d**-**k** with yields varying from 24 to 75%.

Surprisingly, halogen-magnesium exchanges did not lead to complete degradation of the starting phosphinine **1b**, as can be expected from substrates bearing sensitive esters groups. Formylation and carboxylation products **6a** and **6b** were isolated in 23% yield.

Fluorescent molecules^29^ are essential in biological imaging^30^, enabling visualization of cells^31^, proteins, and genetic material in real-time using fluorescence microscopy. In medical diagnostics^32^, they are used in techniques like PCR and immunofluorescence to detect diseases, track infections, and analyze blood cells^33^. In materials science, they enhance imaging technologies^34^, contribute to nanotechnology, and are key components in OLED displays^35^ and biosensors^36^.

As initially reported by different groups, phosphinine derivatives usually exalt good fluorescent properties, which can be tuned by modulation of their substituent’s nature. With a large variety of sophisticated λ^5^-phosphinines in hands, we then evaluated the fluorescence properties of selected compounds in chloroform.

**Scheme 6:**
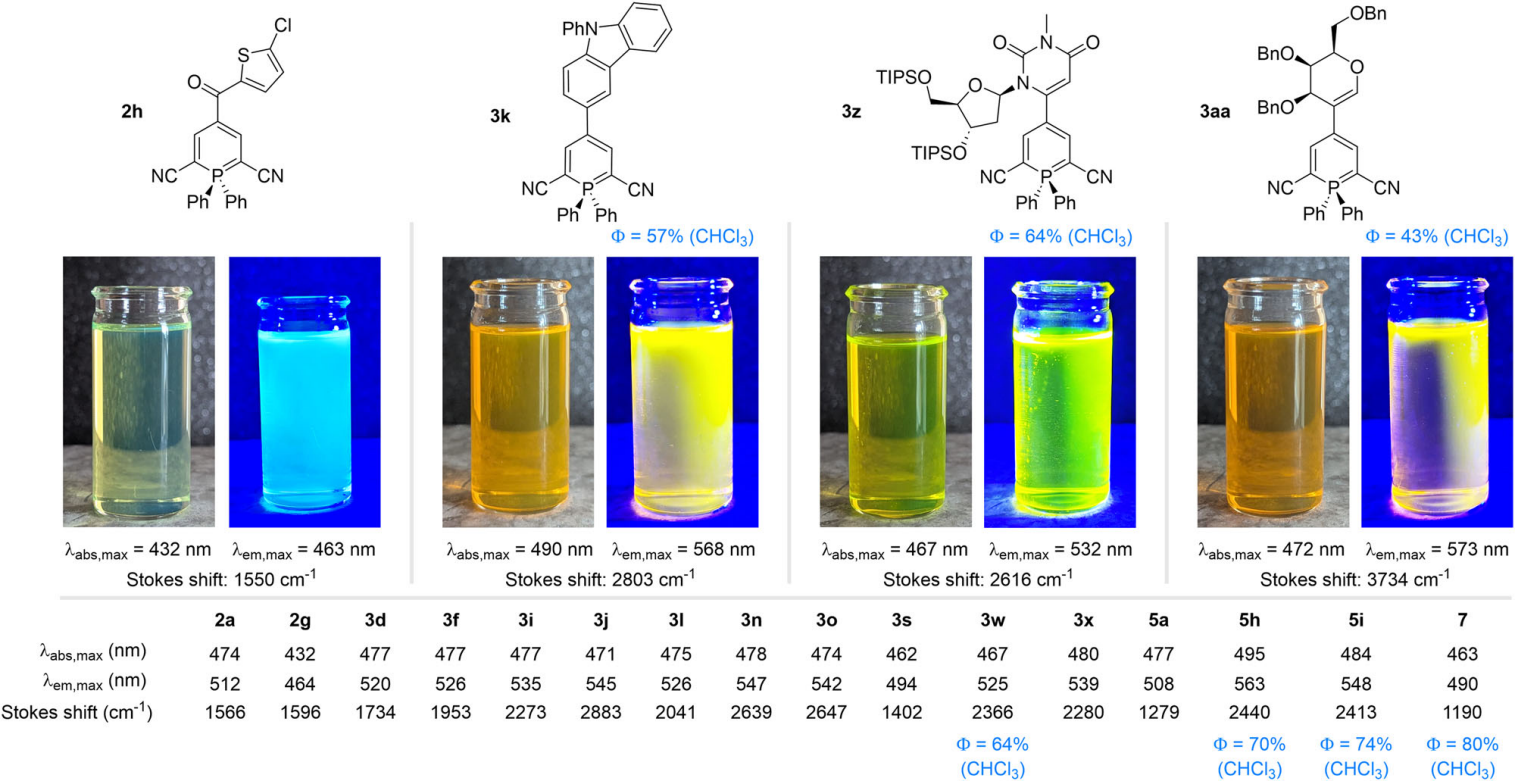
Absorption and fluorescence band maxima for selected λ^5^-phosphinine derivatives in chloroform.

The lowest wavelengths absorption/fluorescence values (432 nm/463 nm) were observed with phosphinine **2 h** bearing a carbonyl group (electron-withdrawing) at position 4 (Scheme 6). On the contrary, **3k** and **5 h** bearing electron-rich carbazole moieties provided the highest wavelength fluorescence values (568 nm and 563 nm, respectively). Very similar absorption/fluorescence wavelengths were obtained for ethyl ester (**5**) and nitrile derivatives (**3**-**4**). A general trend can be observed in which electron-rich moieties promote larger Stokes shifts (e.g. **3aa**, 3734 cm^−1^) compared to electron-deficient ones (e.g **3 s**, 1402 cm^−1^). For reference, we also measured the fluorescence of unsubstituted ethyl ester compound **7**, that show comparable values to the ones reported by Hayashi for the nitrile derivative^22–24^. Noteworthy, moderate-to-high quantum yields were measured on selected compounds of different absorption wavelengths and Stokes shifts (ranging from 58 to 80%).

Finally, we evaluated the photochemical properties of labeled biorelevant scaffolds. On the one hand, nucleoside **3z** showed efficient fluorescence at 532 nm under irradiation at 467 nm, with a quantum yield of 64%. On the other hand, the galactal derivative **3aa** showed fluorescence at 573 nm when irradiated at 472 nm with a somewhat lower–yet reasonable–quantum yield of 43%. It is important to mention that higher quantum yields in average were measured in our case (λ^5^-phosphinines with 2 aryl substituents at the phosphorus) than the ones measured for λ^5^-phosphinines with heteroatoms attached to the phosphorus atom (Φ = 13 to 42%).[9b].

## Conclusion

In summary, we have established a novel strategy for accessing substituted λ⁵-phosphinines via halogen–zinc and –magnesium exchange reactions. The unprecedented generation of 4-phosphininyl-metal intermediates proved highly effective for the introduction of a broad variety of functional groups through electrophilic trapping and cross-coupling methodologies. Furthermore, these distinctive molecular scaffolds exhibited remarkable fluorescent properties, with quantum yields reaching up to 80%, thereby opening new perspectives for their application in biomarking and related fields.

## Supplementary information

Supplementary Information

Description of Additional Supplementary Files

Supplementary Data 1

Supplementary Data 2

## Data Availability

Data for this manuscript has been deposited in figshare: 10.6084/m9.figshare.29245868. “Supplementary Methods” contains detailed protocols for the preparation of substrates, reaction optimizations, scope evaluation and description of analytical data (^1^H and ^13^C NMR, HRMS). “Supplementary Data 1” contains all ^1^H,^13^C, ^31^P and ^19^F NMR spectra. “Supplementary Data 2” contains all absorption and emission (fluorescence) spectra.
