# Supplementary material for: Functionalization of λ5-Phosphinines via metalation strategies
Source: Commun Chem. 2025 Dec 22;8:414. doi: 10.1038/s42004-025-01822-6 (PMC12748612; doi:10.1038/s42004-025-01822-6)

Functionalization of λ5-Phosphinines *via* Metalation Strategies

Flavie Rambaud,[a] Bertrand Takam Fotie,[a] Robert Naumann,[b] Katja Heinze,[b] Dorian Didier*[a]

[a] Technische Universität Darmstadt, Clemens-Schöpf-Institut, Peter-Grünberg-Straße 4, 64287 Darmstadt

[b] Johannes Gutenberg Universität Mainz, Department of Chemistry, Duesbergweg 10-14, 55128 Mainz

Absorption and Emission Spectra of *para*-Substituted λ5-Phosphinines

Spectrometer 1: Fluorescence spectra and absolute emission quantum yields for compounds 3k, 3z, 3aa, 3w, 5h, 5i and 7 were measured with a *FLS1000* spectrometer from *Edinburgh Instruments* equipped with a cooled photomultiplier detector PMT-980. A xenon arc lamp Xe2 (450 W) was used for excitation. Absolute luminescence quantum yields Φ were determined using an integration sphere from *Edinburgh Instruments*. The sample solutions were measured in quartz cuvettes with an optical path length of 1 cm and with a concentration of 0.1 mM. Relative uncertainty of Φ is estimated to be ± 10%.

Spectrometer 2: UV-Vis for compounds 2a, 2g, 2h, 3d, 3f, 3j, 3k, 3l, 3n, 3o, 3r, 3s, 3x and 5a were recorded on Analytik Jena Specord 600 UV-Vis spectrometer, **fluorescence spectra** were recorded on J&M TIDAS S700/CCD UV/NIR 2098 spectrometer combined with J&M TIDAS LSM monochromator with 75 W Xenon light source and thermo-controlled cuvette holder. Samples for emission and absorption measurements were contained in 1 cm quartz cuvette (Hellma Analytics).

Compound 3r

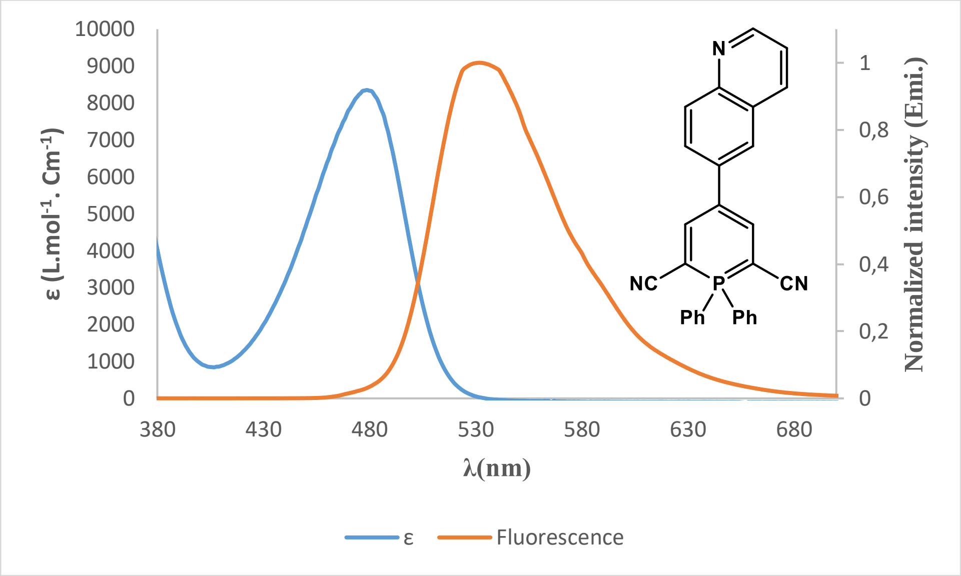

$\lambda_{abs,max}$/nm= 479

$\lambda_{em,max}$/nm= 532

A_{max} = 0.835178

ε = 8351.78 L.mol^{-1}.cm^{-1} Stokes-shifts= 2080 (cm^{-1}) or 53 (nm)

Compound 2h

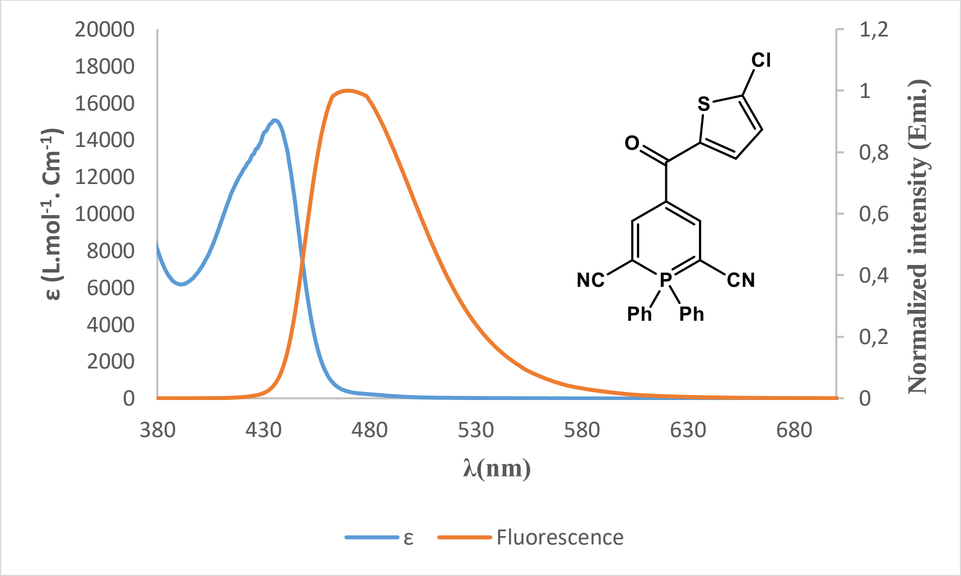

$\lambda_{abs,max}$/nm= 435

$\lambda_{em,max}$/nm= 470

A_{max} = 1.506429

ε = 15064.29 L.mol^{-1}.cm^{-1} Stokes-shifts= 1712 (cm^{-1}) or 35 (nm)

Compound 2g

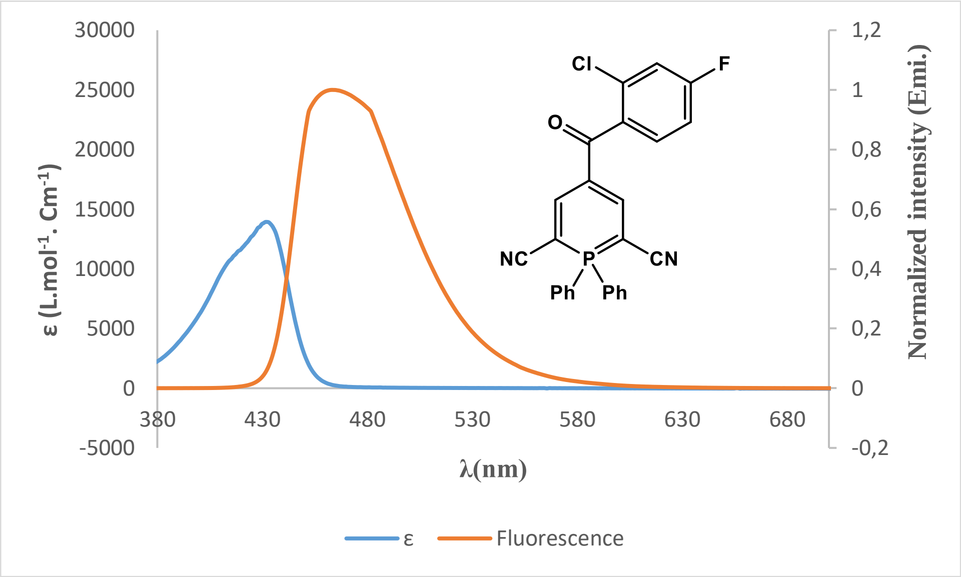

$\lambda_{abs,max}$/nm= 432

$\lambda_{em,max}$/nm= ca. 464

A_{max} = 1.393526

ε = 13935.26 $L.mol^{-1}.cm^{-1}$ Stokes-shifts= 1596 (cm^{-1}) or 32 (nm)

Compound 3d

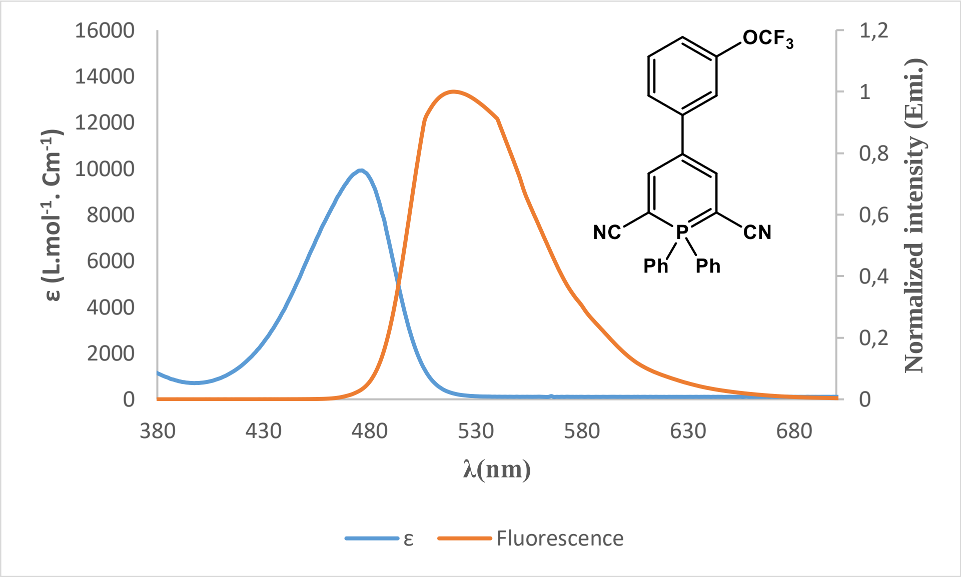

$\lambda_{abs,max}$/nm= 477

$\lambda_{em,max}$/nm= ca. 520

A_{max} = 0.990009

ε = 9900.09 $L.mol^{-1}.cm^{-1}$ Stokes-shifts= 1734 (cm^{-1}) or 43 (nm)

Compound 3l

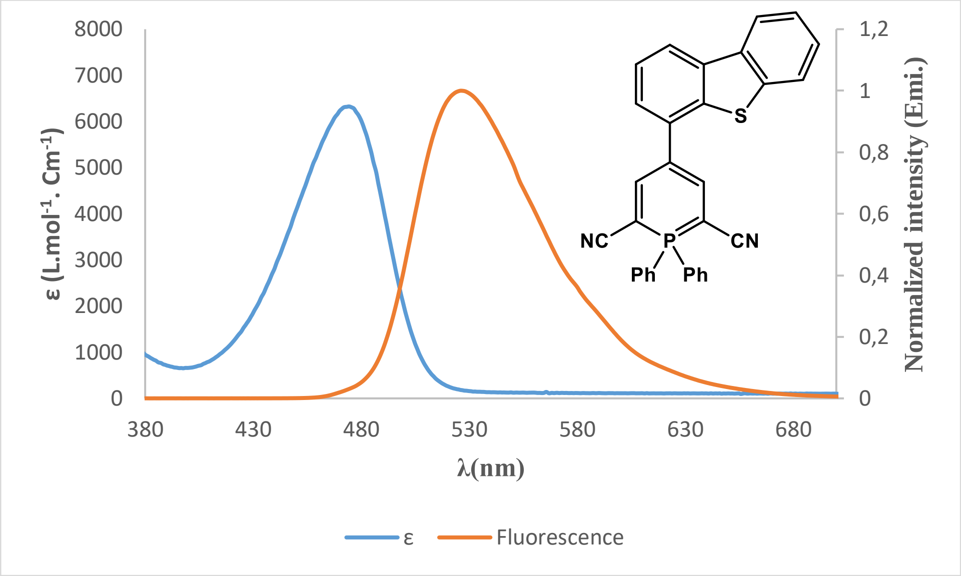

$\lambda_{abs,max}$/nm= 475

$\lambda_{em,max}$/nm= 526

A_{max} = 0.632545

ε = 6325.45 $L.mol^{-1}.cm^{-1}$ Stokes-shifts= 2041 (cm^{-1}) or 51 (nm)

Compound 3k

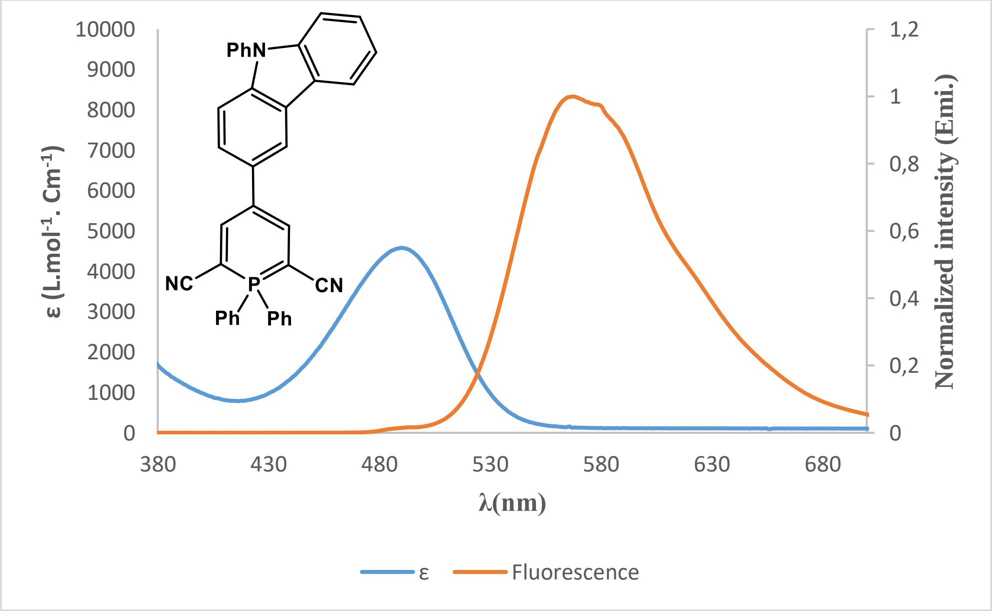

$\lambda_{abs,max}$/nm= 490

$\lambda_{em,max}$/nm= 568

A_{max} = 0.458107

ε = 4581.07 $L.mol^{-1}.cm^{-1}$ Stokes-shifts= 2803 (cm^{-1}) or 78 (nm)

Compound 2a

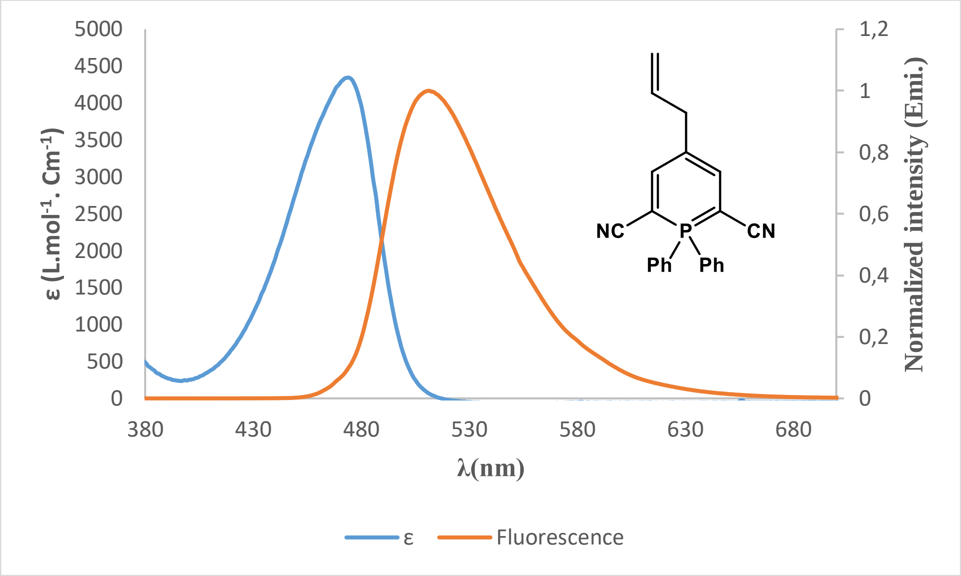

$\lambda_{abs,max}$/nm= 474

$\lambda_{em,max}$/nm= 512

A_{max} = 0.434507

ε = 4345.07 L.mol^{-1}.cm^{-1} Stokes-shifts= 1566 (cm^{-1}) or 38 (nm)

Compound 3s

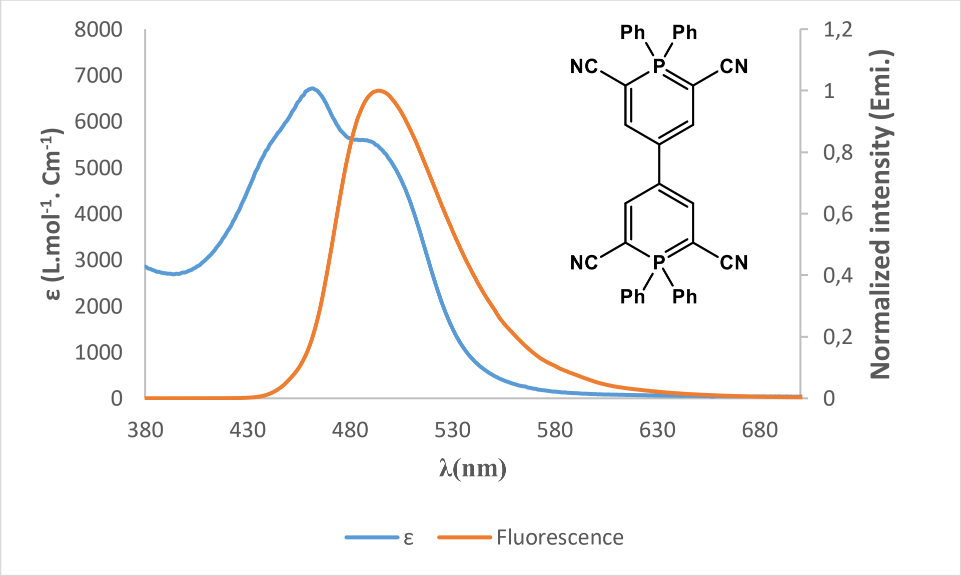

$\lambda_{abs,max}$/nm= 462

$\lambda_{em,max}$/nm= 494

A_{max} = 0.671528

ε = 6715.28 L.mol^{-1}.cm^{-1} Stokes-shifts= 1402 (cm^{-1}) or 32 (nm)

Compound 5a

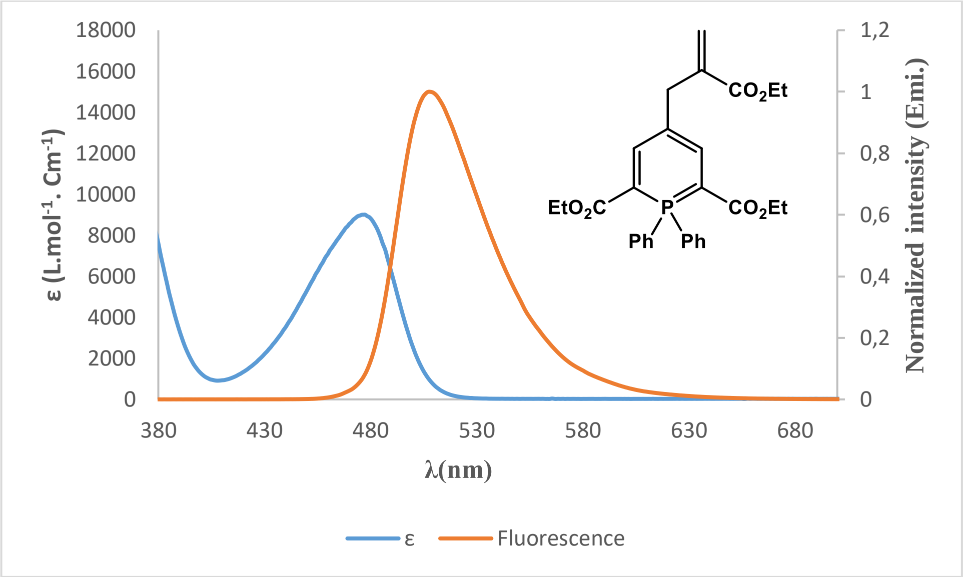

$\lambda_{abs,max}$/nm= 477

$\lambda_{em,max}$/nm= 508

A_{max} = 0.90059

ε = 9005.9 $L.mol^{-1}.cm^{-1}$ Stokes-shifts= 1279 (cm^{-1}) or 31 (nm)

Compound 3f

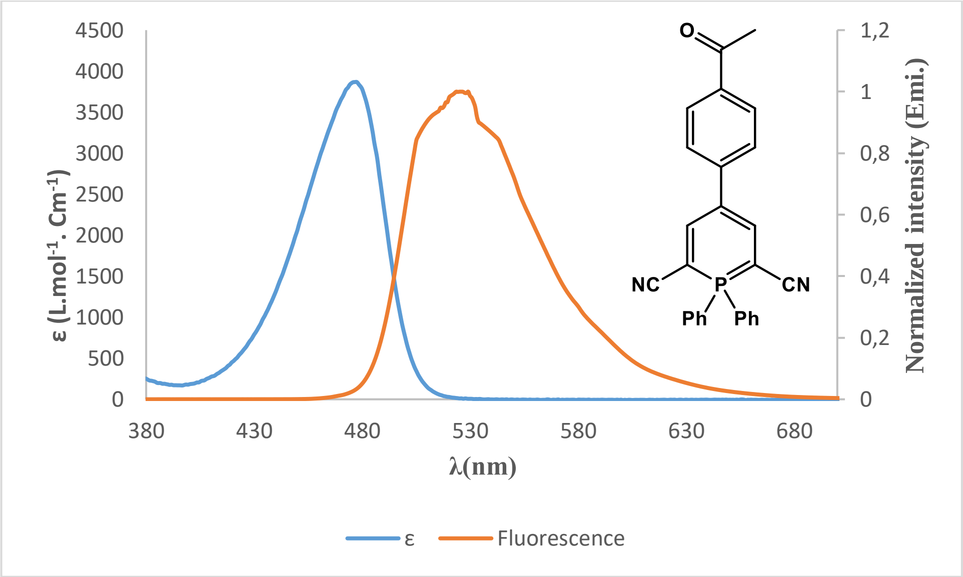

$\lambda_{abs,max}$/nm= 477

$\lambda_{em,max}$/nm= ca. 526

A_{max} = 0.386856

ε = 3868.56 $L.mol^{-1}.cm^{-1}$ Stokes-shifts= 1953 (cm^{-1}) or 49 (nm)

Compound 3x

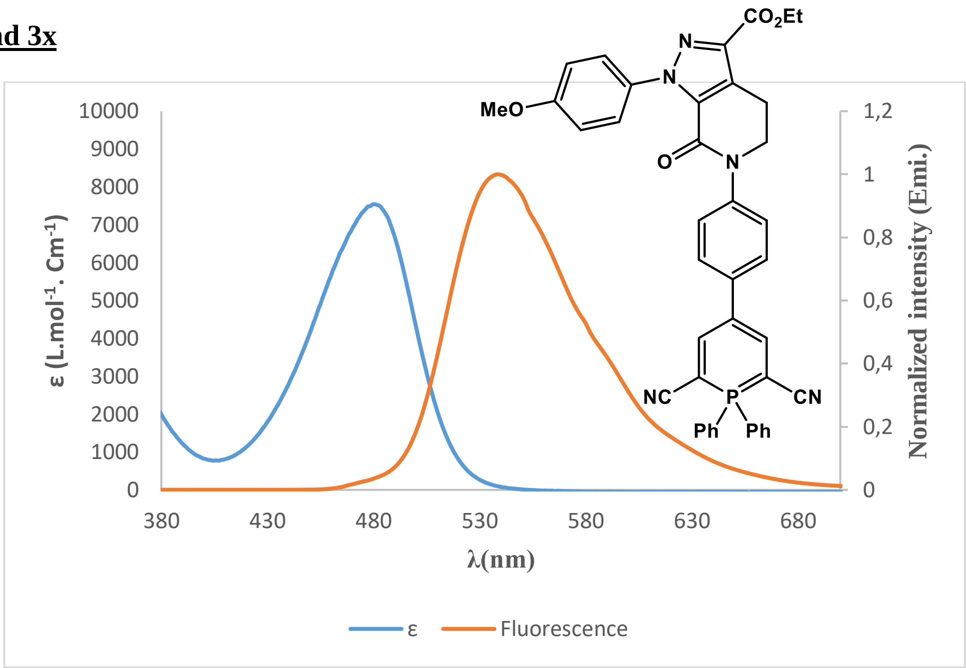

$\lambda_{abs,max}$/nm= 480

$\lambda_{em,max}$/nm= 539

A_{max} = 0.754348

ε = 7543.48 $L.mol^{-1}.cm^{-1}$ Stokes-shifts= 2280 (cm^{-1}) or 59 (nm)

Compound 3j

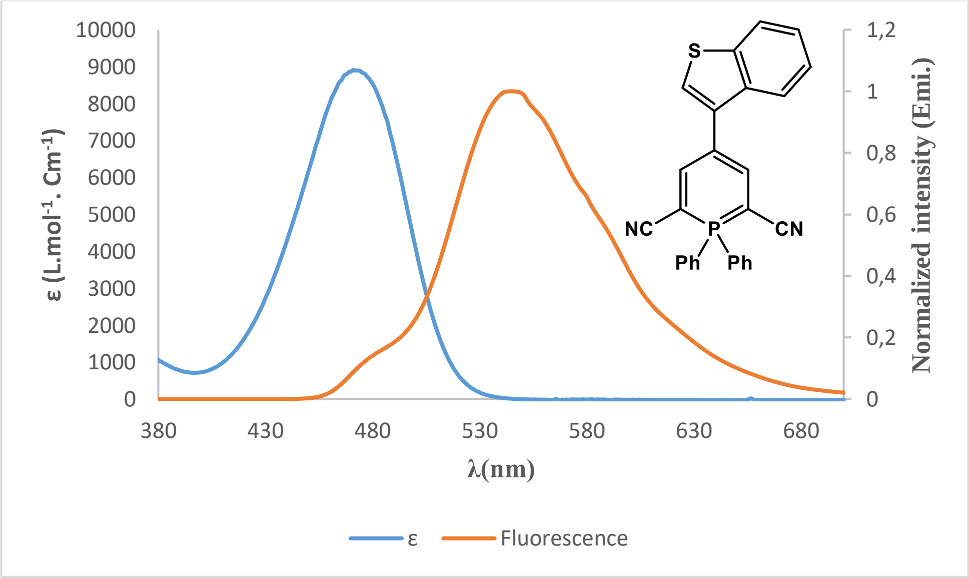

$\lambda_{abs,max}$/nm= 473

$\lambda_{em,max}$/nm= 546

A_{max} = 0.889501

ε = 8895.01 $L.mol^{-1}.cm^{-1}$ Stokes-shifts= 2827 (cm^{-1}) or 73 (nm)

Compound 3n

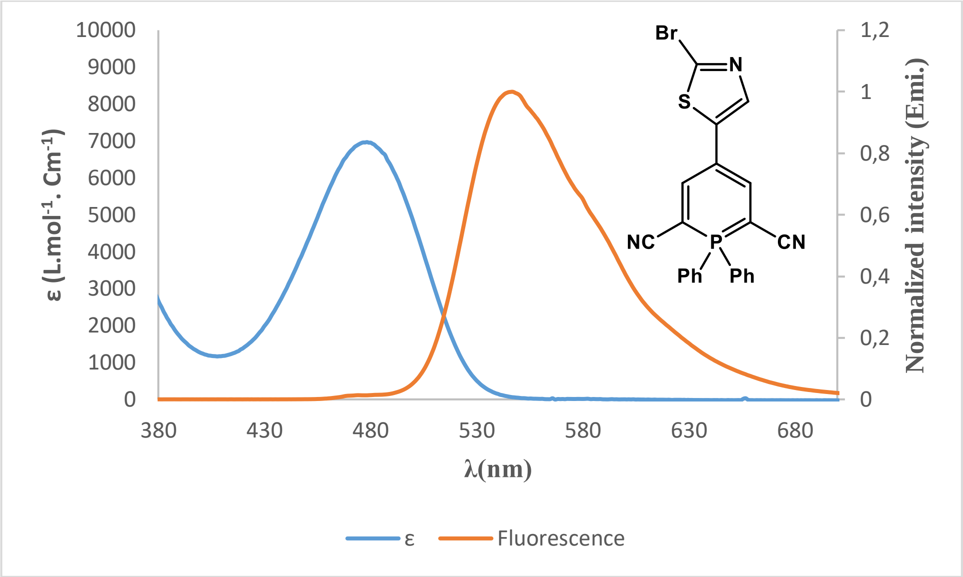

$\lambda_{abs,max}$/nm= 478

$\lambda_{em,max}$/nm= 547

A_{max} = 0.696896

ε = 6968.96 L.mol^{-1}.cm^{-1} Stokes-shifts= 2639 (cm^{-1}) or 69 (nm)

Compound 3o

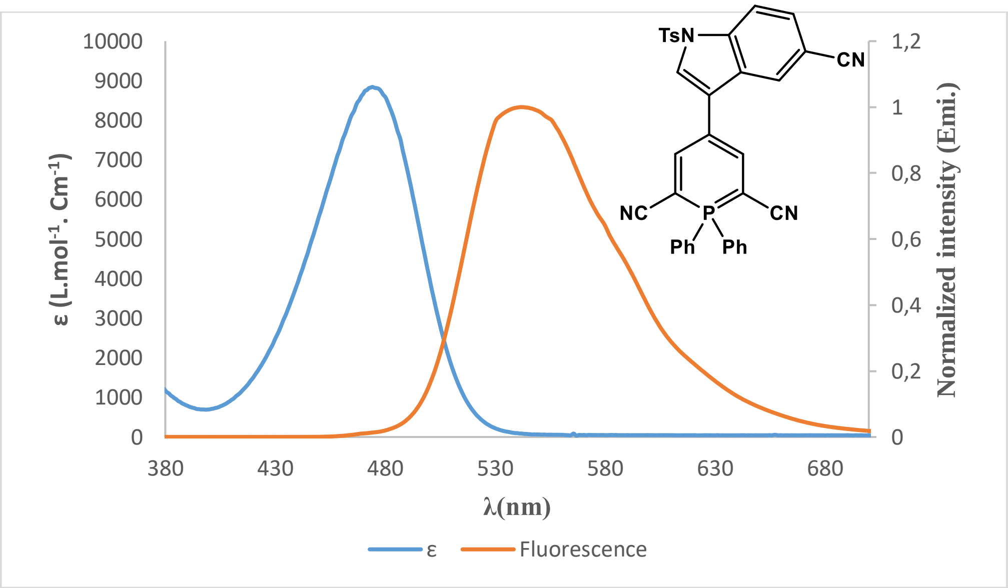

$\lambda_{abs,max}$/nm= 474

$\lambda_{em,max}$/nm= 542

A_{max} = 0.884101

ε = 8841.01 L.mol^{-1}.cm^{-1} Stokes-shifts= 2647 (cm^{-1}) or 68 (nm)

Absorption

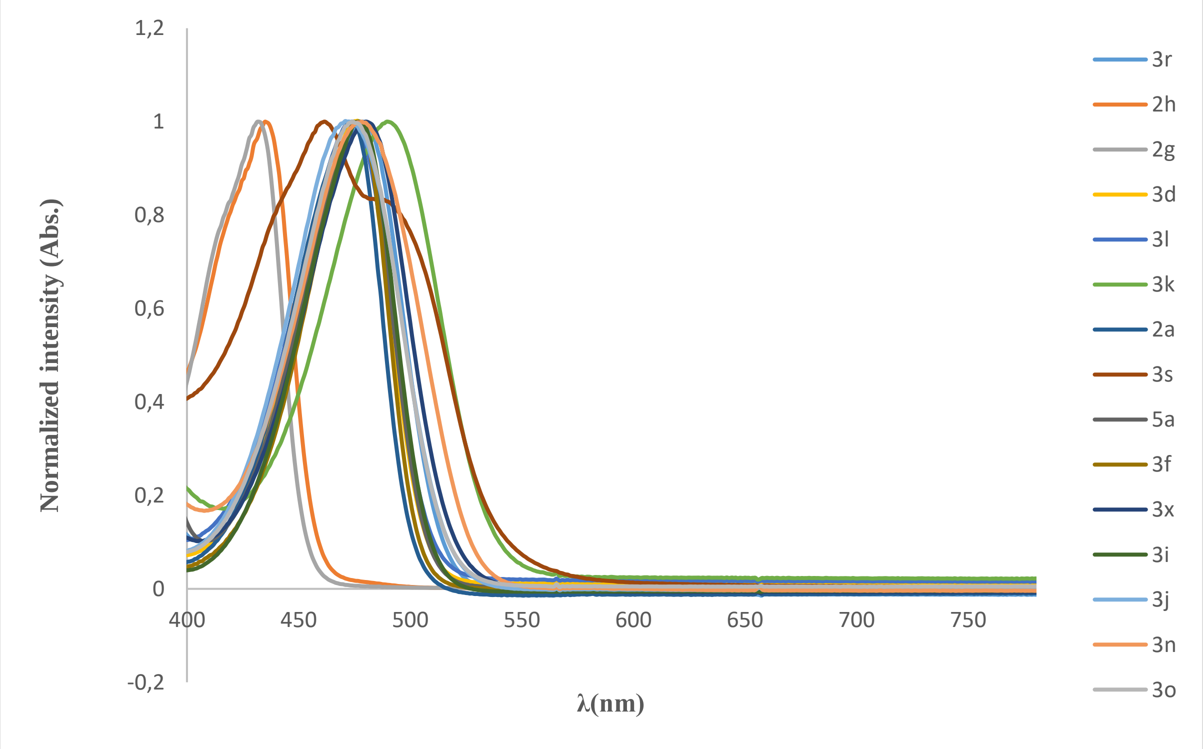

Emission (Fluorescence)

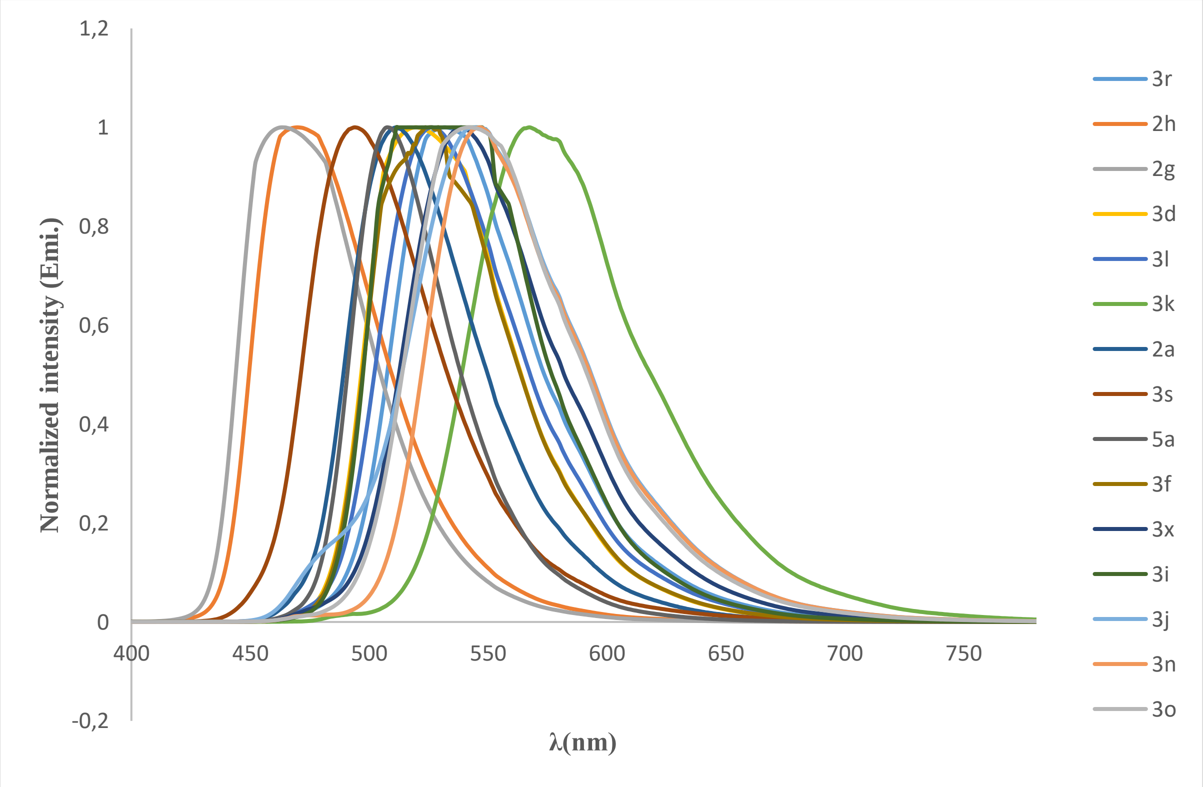

Supplement: Supplementary file 1 — Supplementary Information [file 42004_2025_1822_MOESM1_ESM.pdf]
