# Supplementary material for: Functionalization of λ5-Phosphinines via metalation strategies
Source: Commun Chem. 2025 Dec 22;8:414. doi: 10.1038/s42004-025-01822-6 (PMC12748612; doi:10.1038/s42004-025-01822-6)
Supplement: Supplementary file 2 — Description of Additional Supplementary Files [file 42004_2025_1822_MOESM2_ESM.pdf]

Description of Additional Supplementary Files:

File: Supplementary Data 1

Description: Contains all 1H,13C, 31P and 19F NMR spectra.

File: Supplementary Data 2

Description: Contains all absorption and emission (fluorescence) spectra.
