# Supplementary material for: Functionalization of λ5-Phosphinines via metalation strategies
Source: Commun Chem. 2025 Dec 22;8:414. doi: 10.1038/s42004-025-01822-6 (PMC12748612; doi:10.1038/s42004-025-01822-6)

Functionalization of λ⁵-Phosphinines *via* Metalation Strategies

Flavie Rambaud,[a] Bertrand Takam Fotie,[a] Robert Naumann,[b] Katja Heinze,[b] Dorian Didier*[a]

[a] Technische Universität Darmstadt, Clemens-Schöpf-Institut, Peter-Grünberg-Straße 4, 64287 Darmstadt

[b] Johannes Gutenberg Universität Mainz, Department of Chemistry, Duesbergweg 10-14, 55128 Mainz

NMR Spectra

Preparation of λ5-Phosphinines

(*1E,3E*)-N1,N3-Diphenylpropane-1,3-diimine (SI-1)

^{1}H NMR (500 MHz)

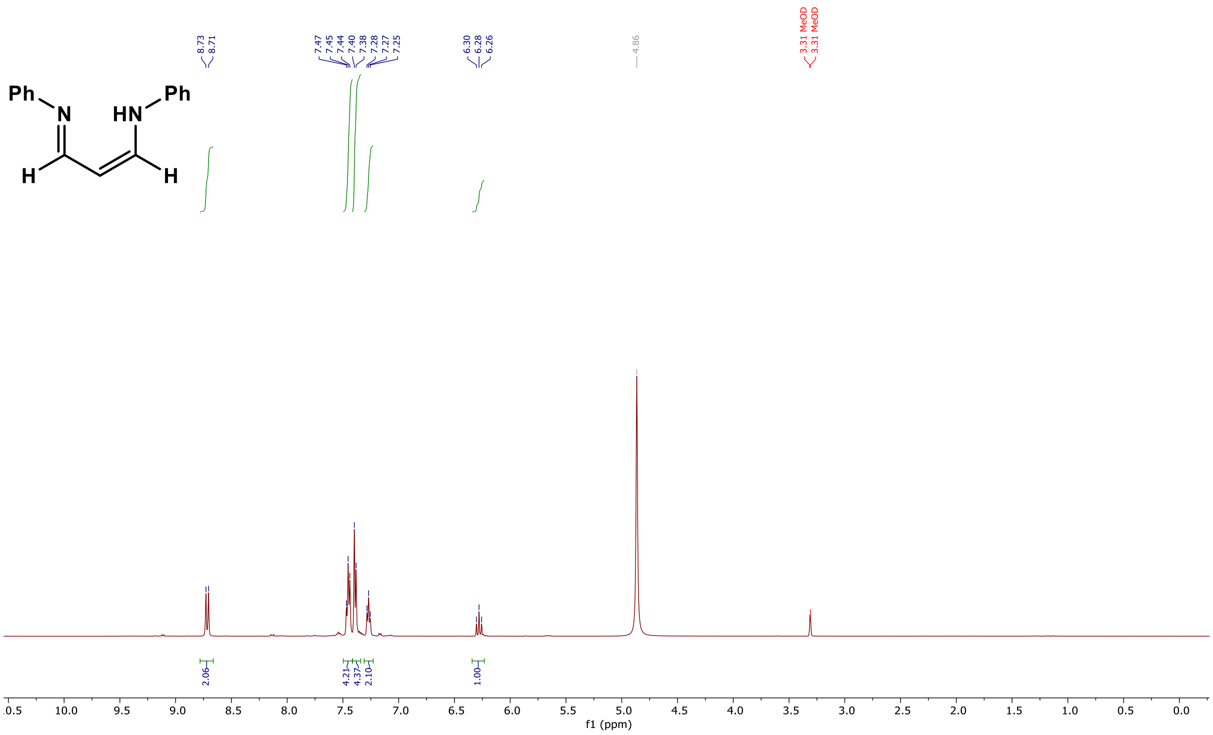

^{13}C NMR (126 MHz)

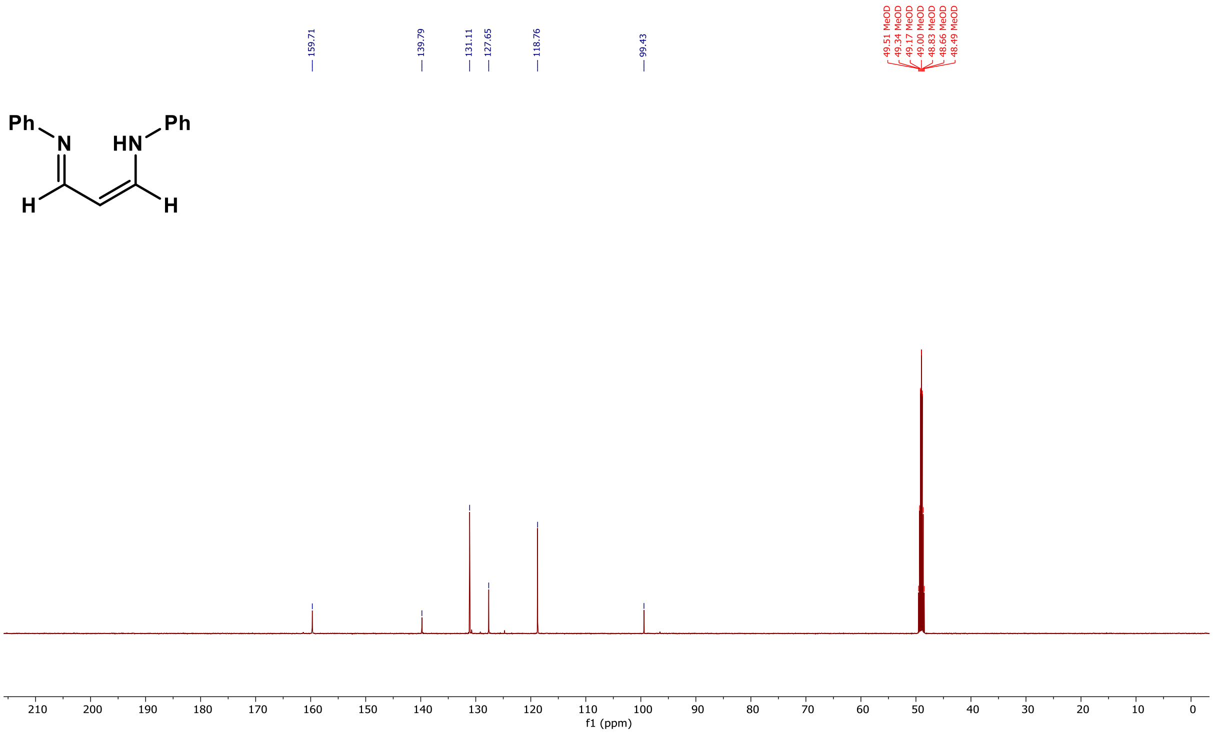

Bis(cyanomethyl)diphenylphosphonium chloride (SI-3a)

^{1}H NMR (500 MHz)

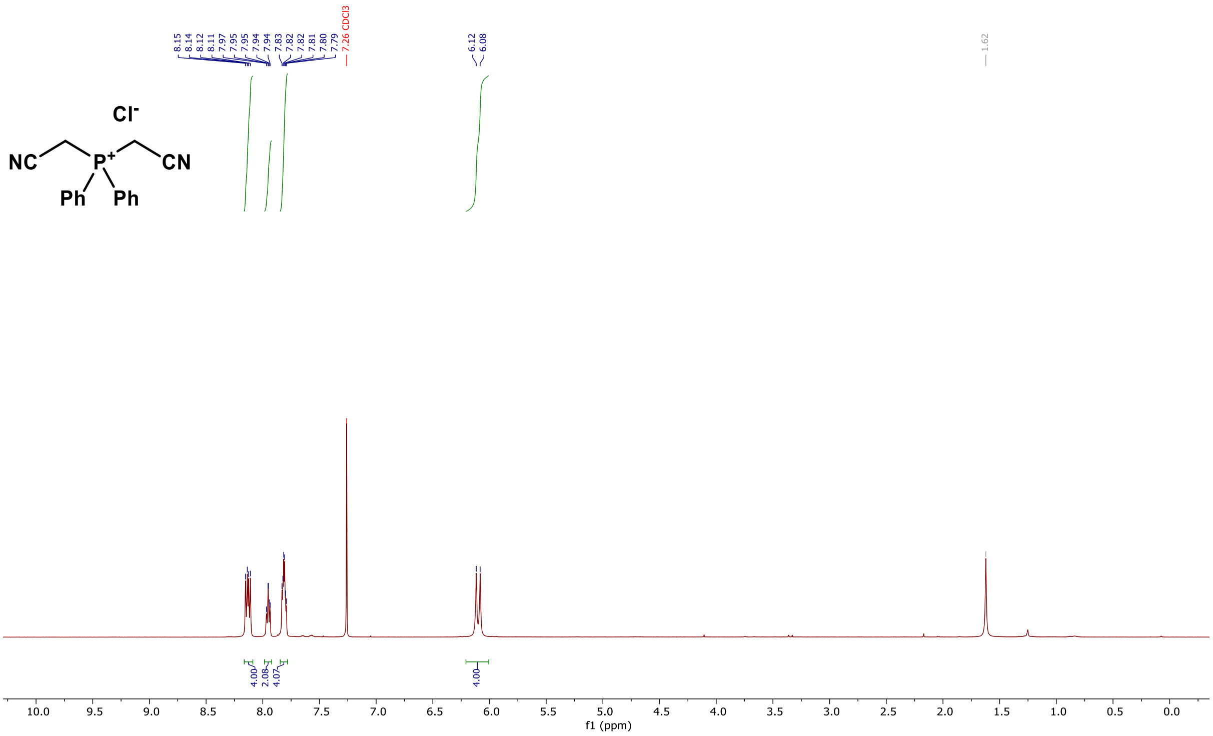

^{13}C NMR (126 MHz)

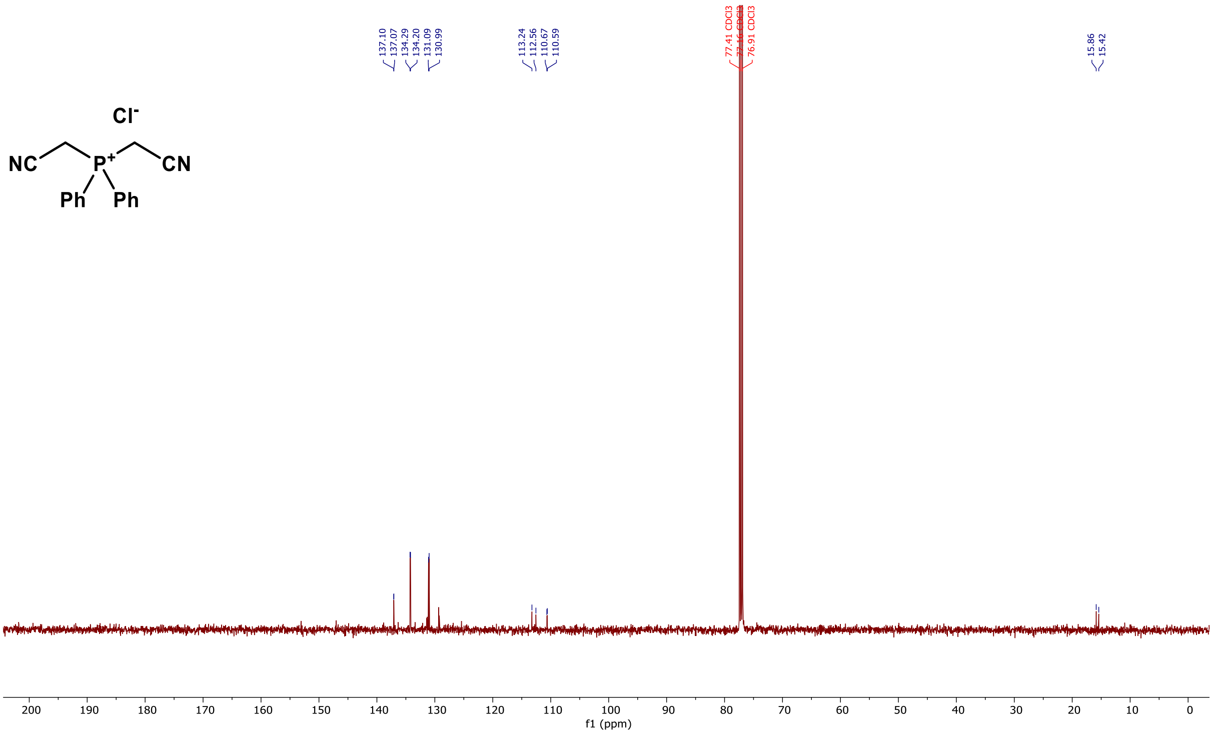

^{31}P NMR (202 MHz)

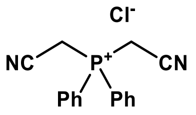

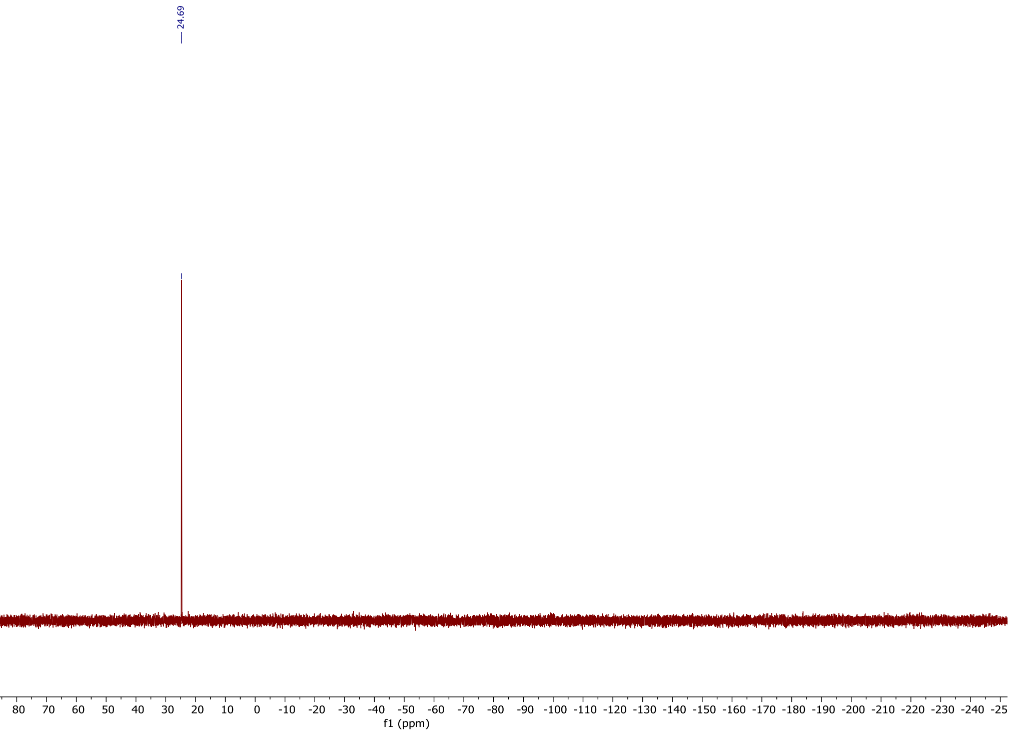

Bis(2-ethoxy-2-oxoethyl)diphenylphosphonium bromide (SI-3b)

^{1}H NMR (500 MHz)

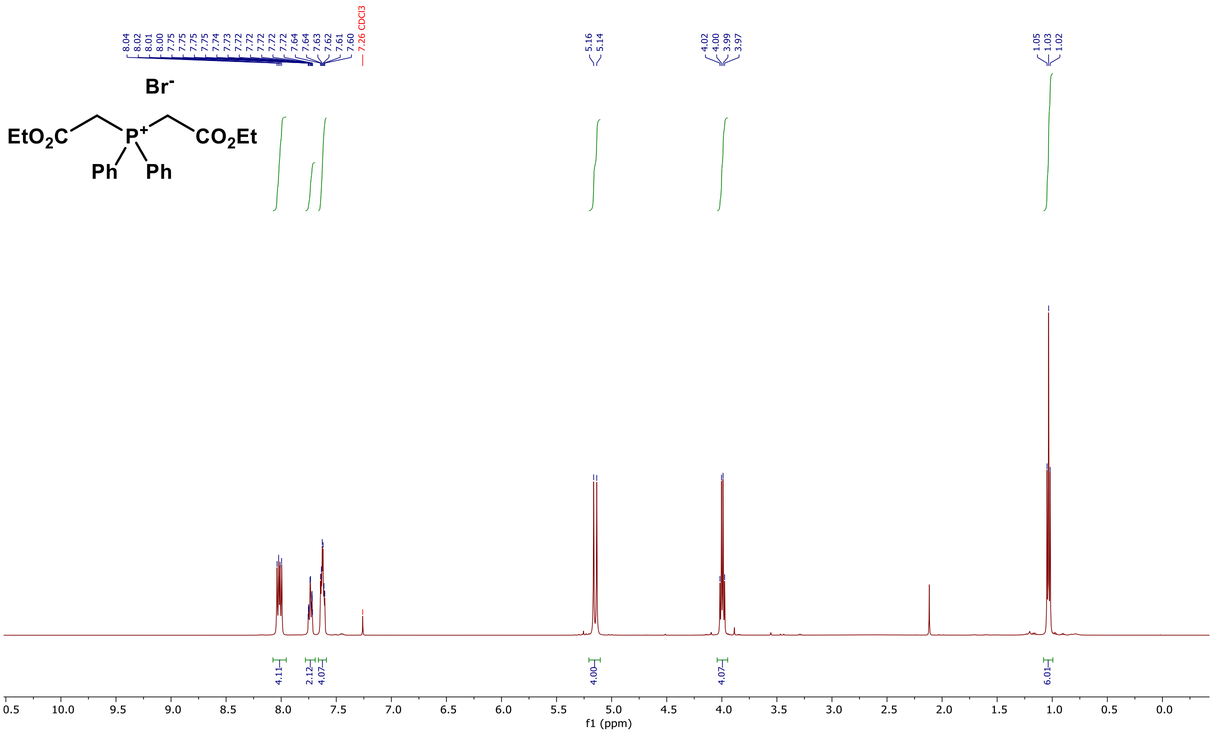

^{13}C NMR (126 MHz)

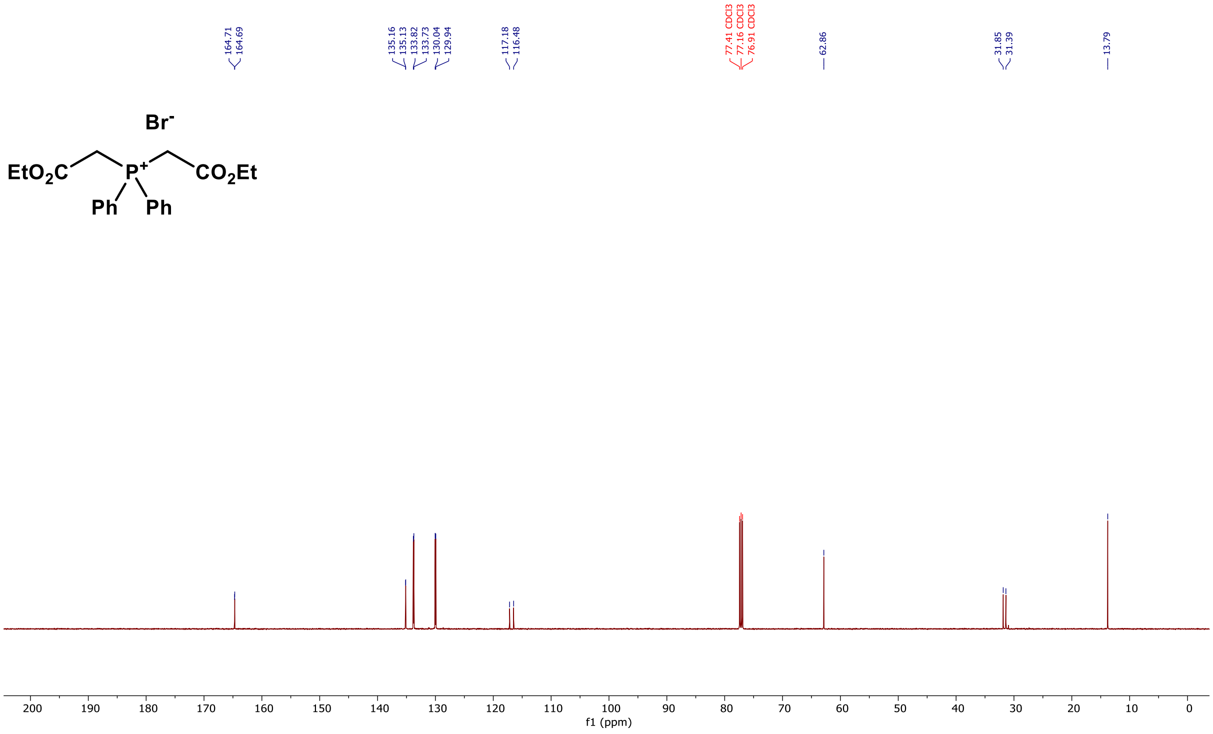

^{31}P NMR (202 MHz)

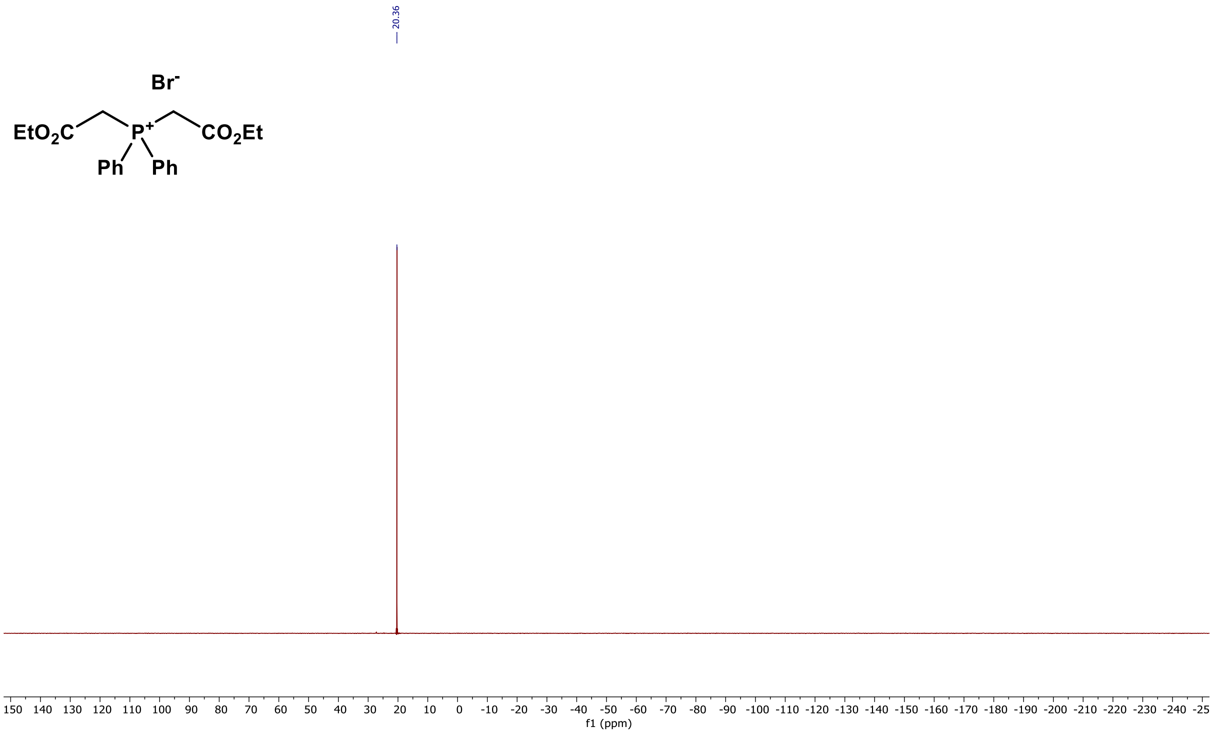

1,1-diphenyl-1λ⁵-phosphinine-2,6-dicarbonitrile (SI-4)

^{1}H NMR (500 MHz)

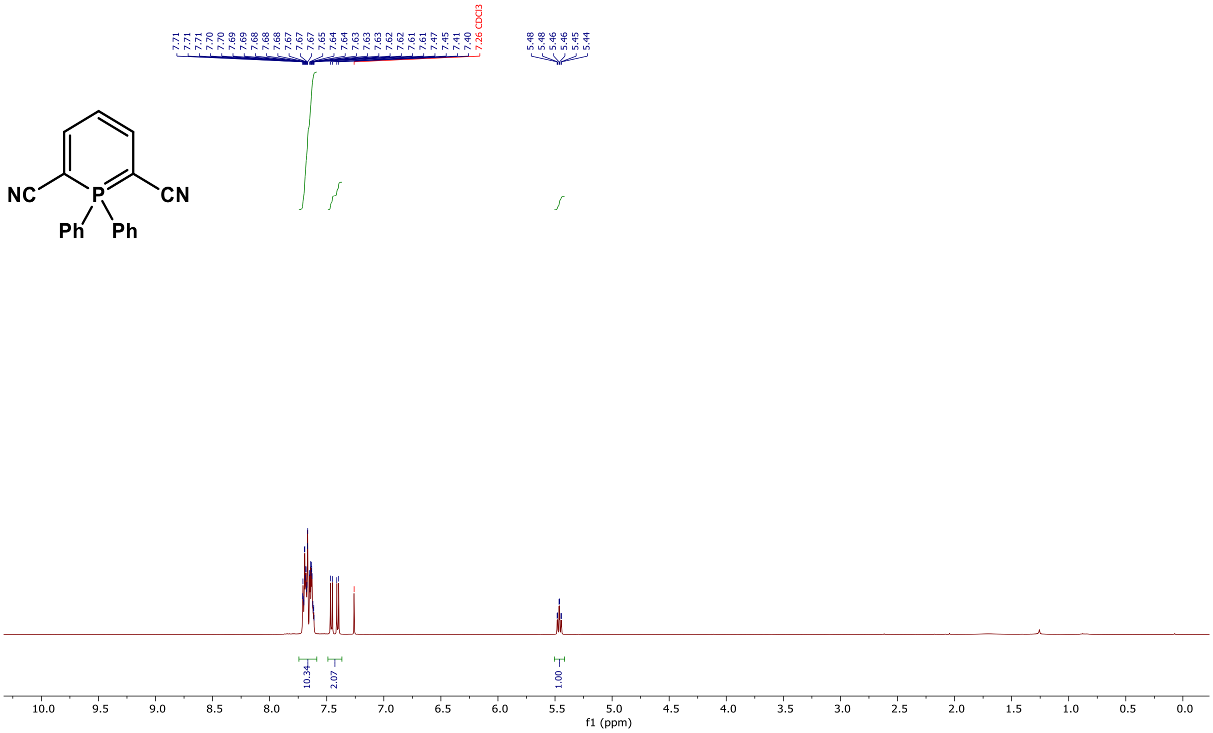

^{13}C NMR (126 MHz)

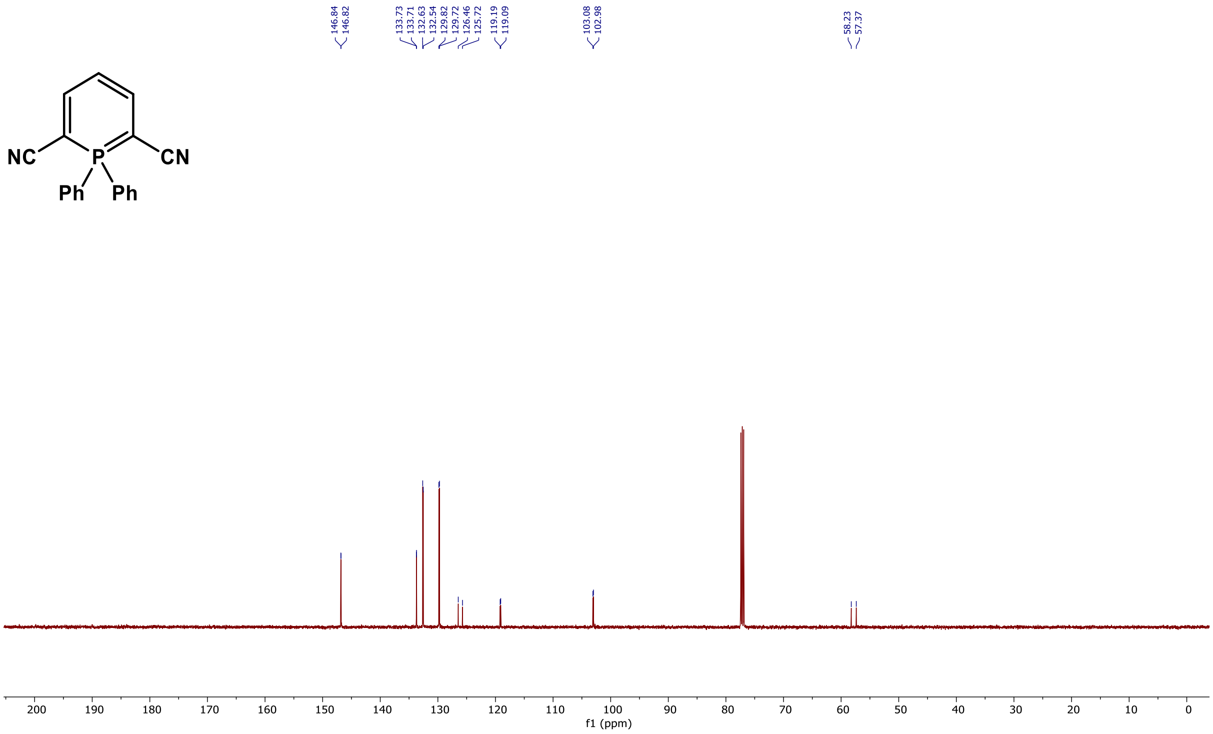

^{31}P NMR (202 MHz)

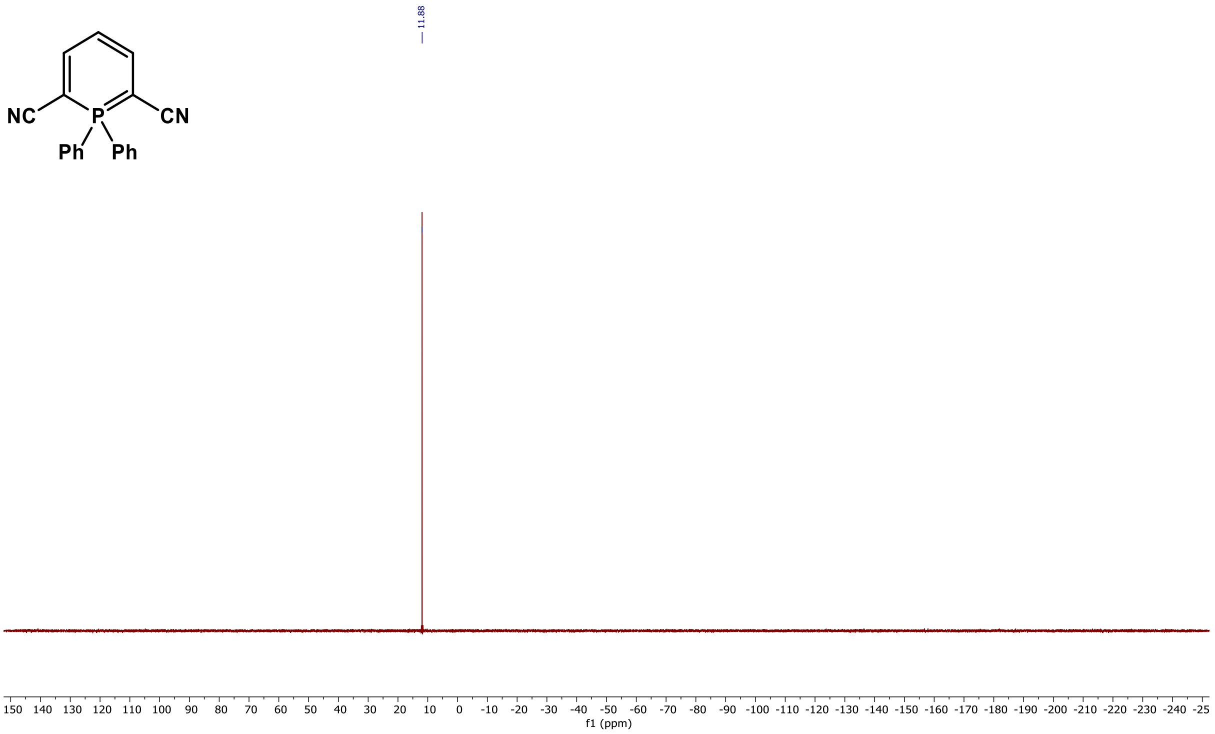

Diethyl 1,1-diphenyl-1λ⁵-phosphinine-2,6-dicarboxylate (7)

^{1}H NMR (500 MHz)

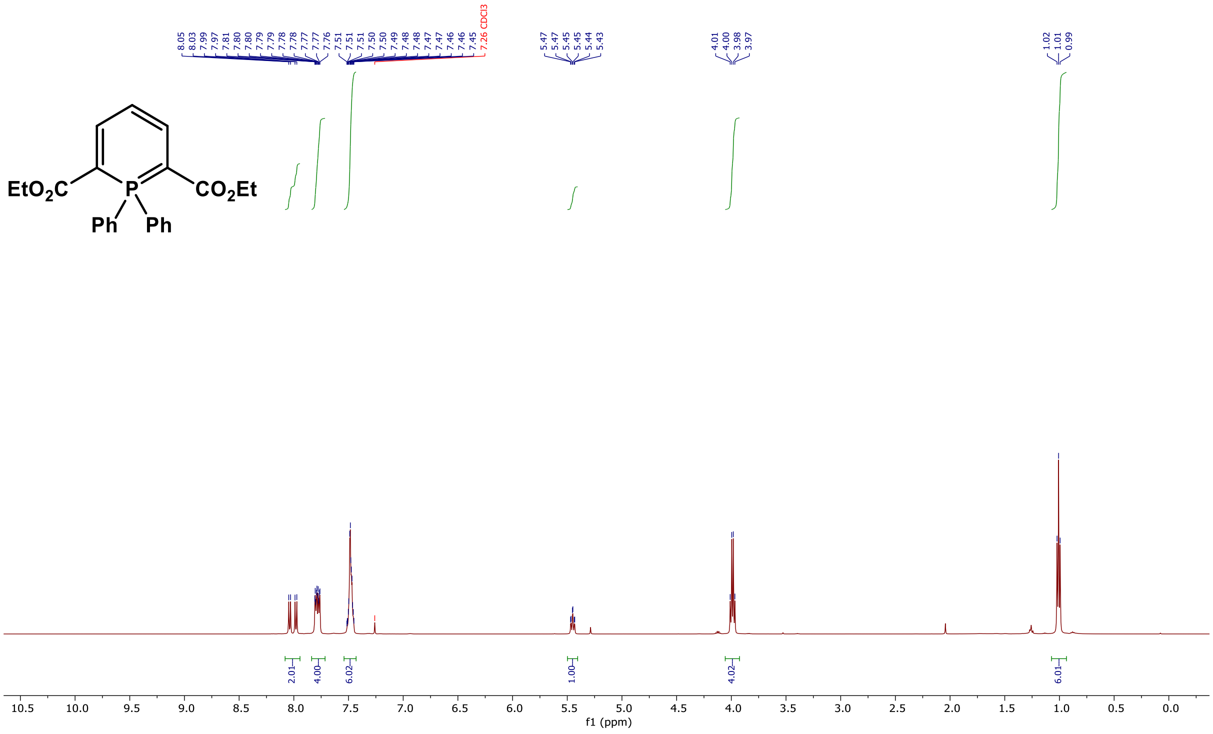

^{13}C NMR (126 MHz)

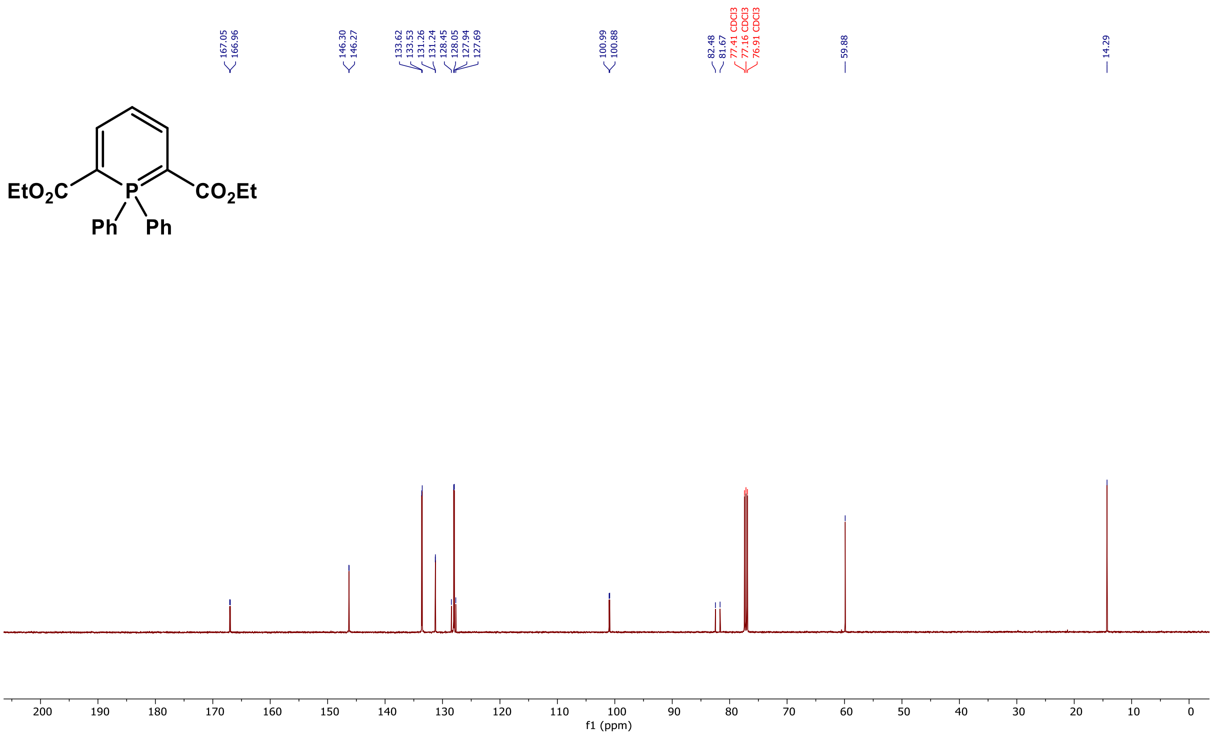

^{31}P NMR (202 MHz)

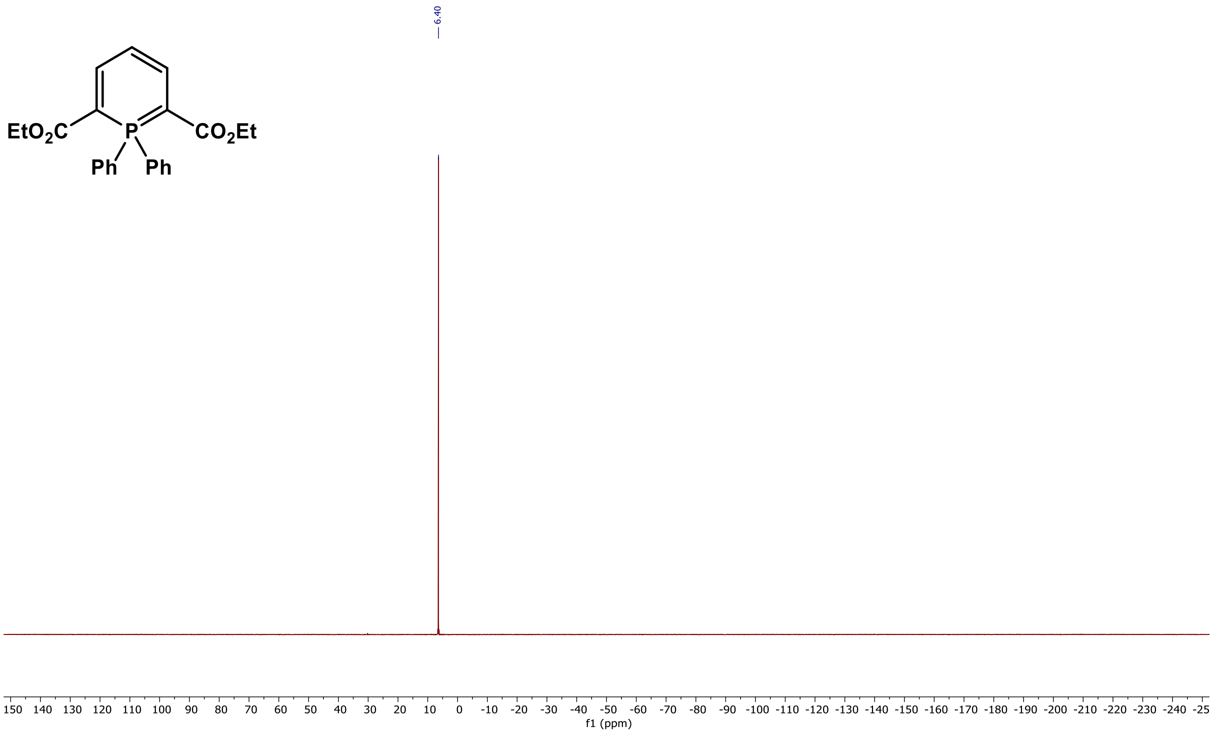

4-iodo-1,1-diphenyl-1λ^5-phosphinine-2,6-dicarbonitrile (1a)

^{1}H NMR (500 MHz)

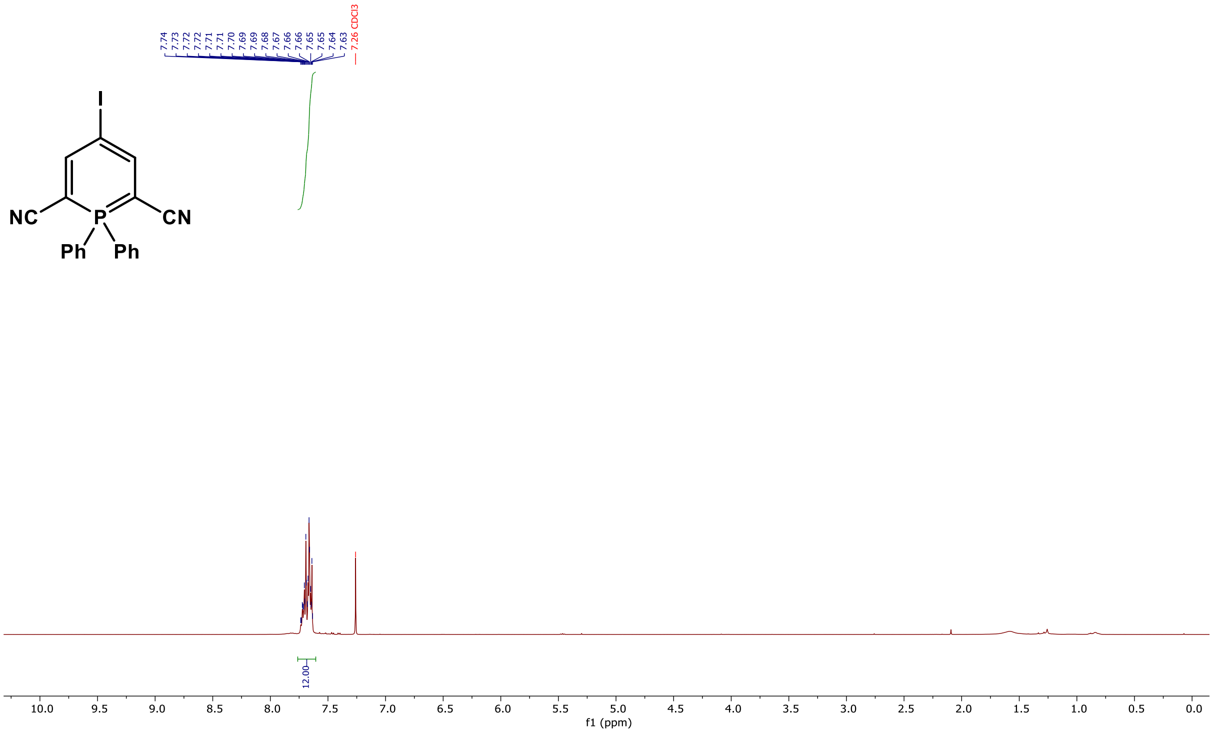

^{13}C NMR (126 MHz)

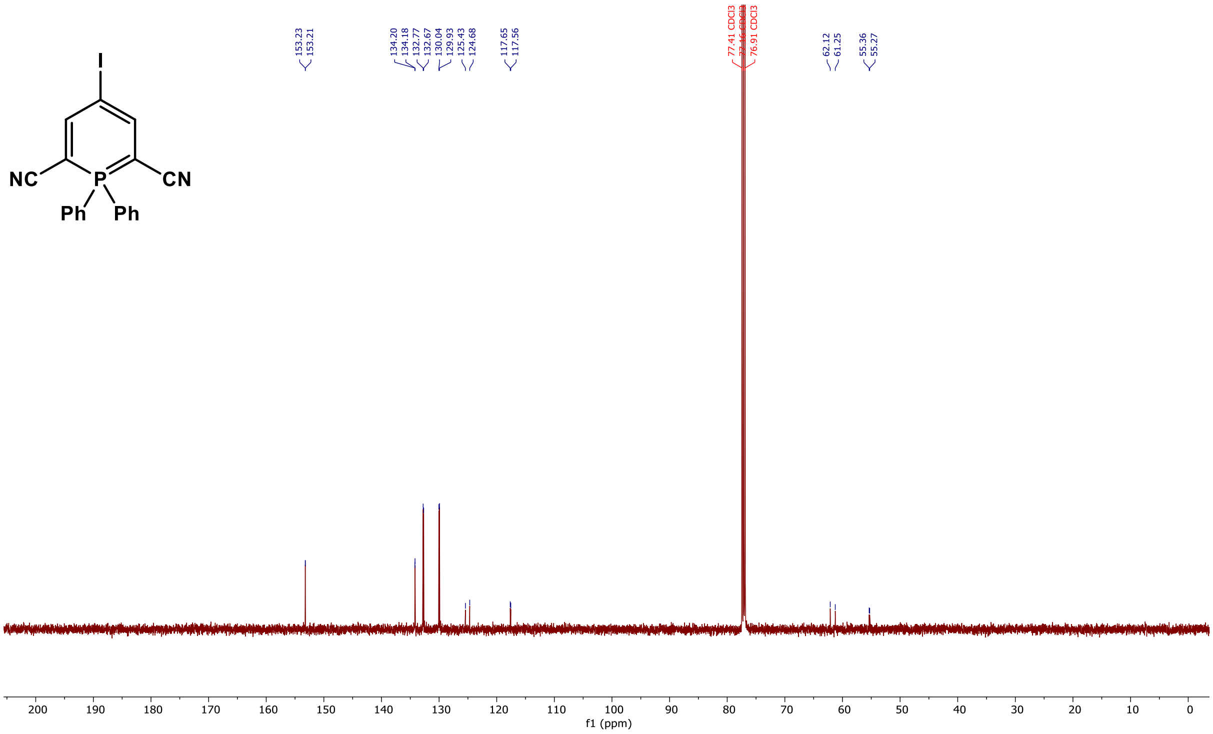

^{31}P NMR (202 MHz)

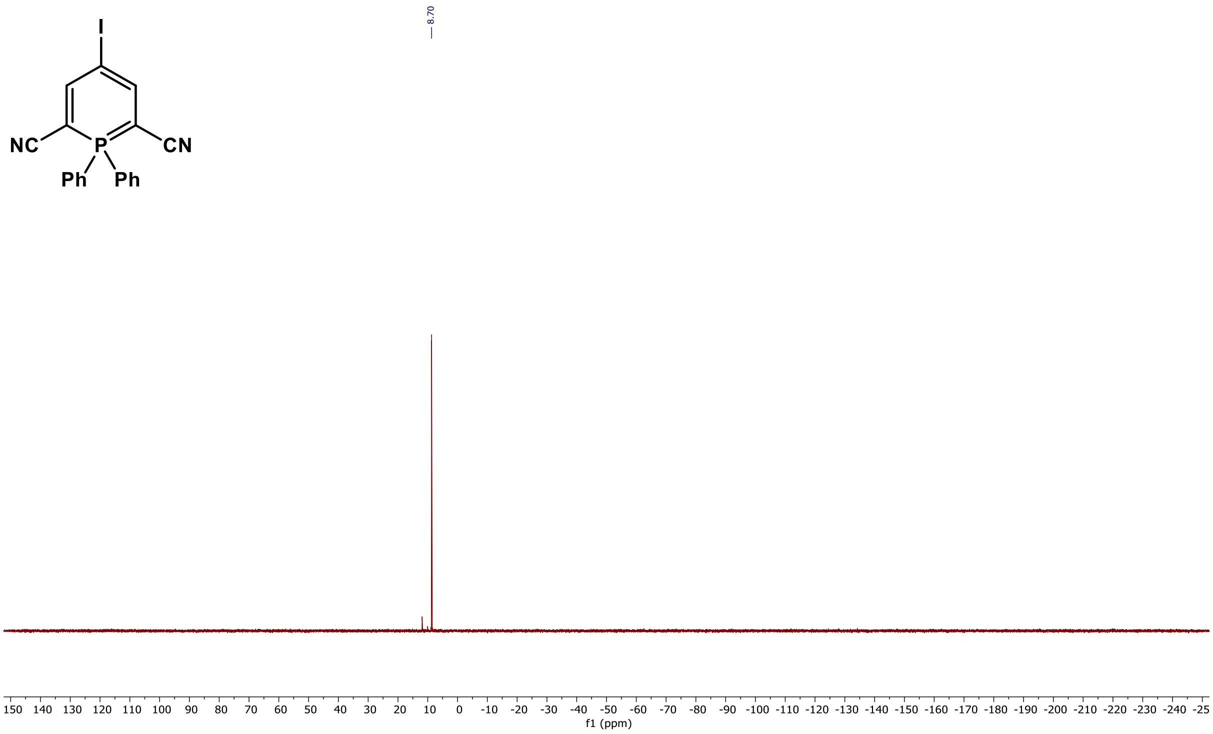

Diethyl 4-iodo-1,1-diphenyl-1λ^5-phosphinine-2,6-dicarboxylate (1b)

^{1}H NMR (500 MHz)

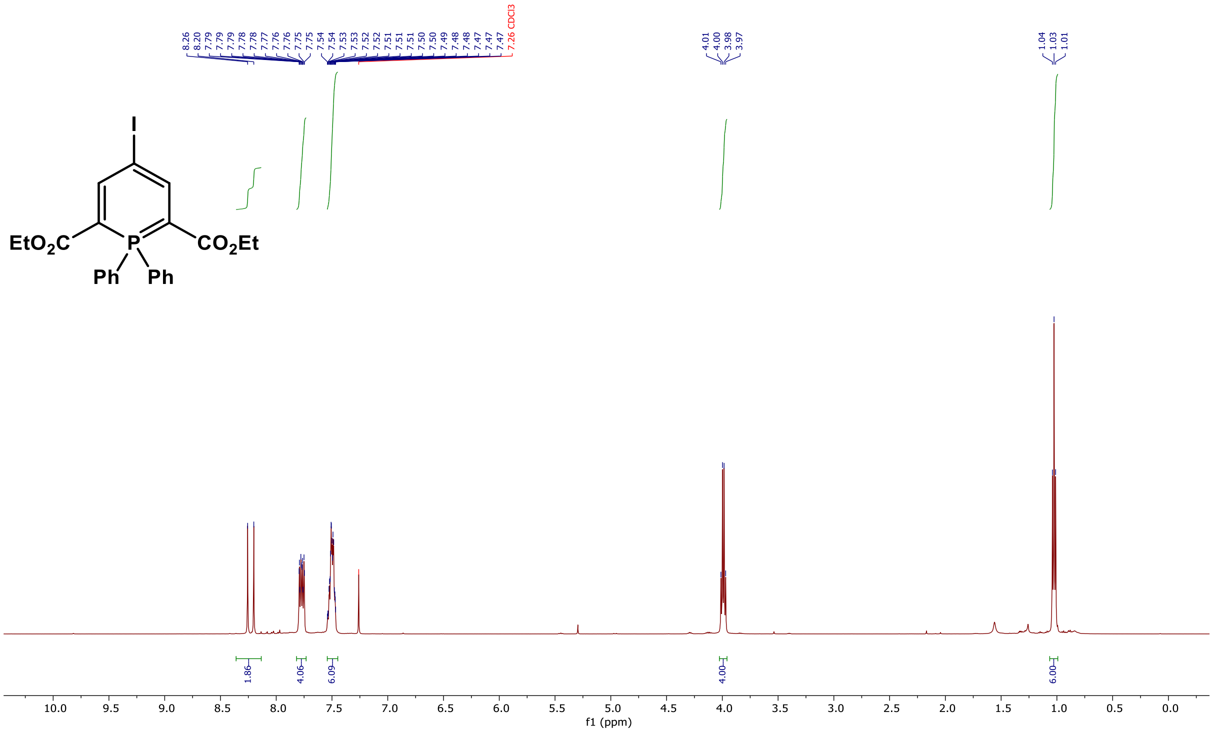

^{13}C NMR (126 MHz)

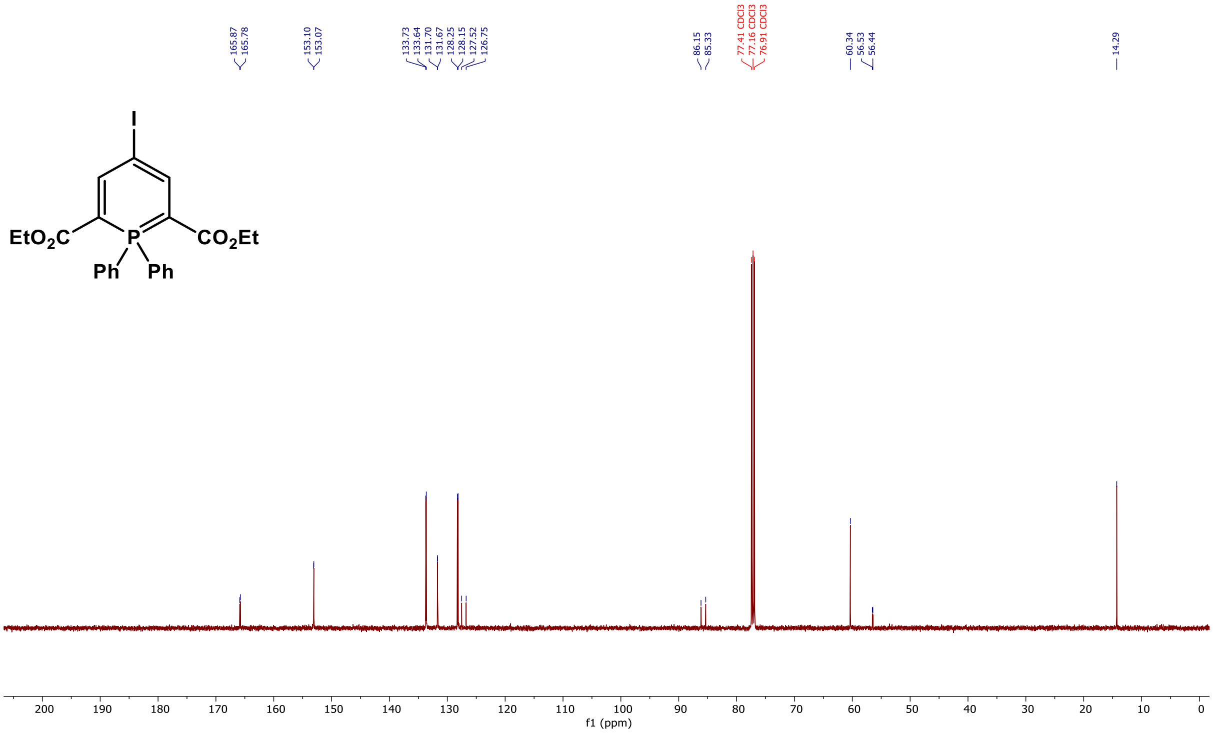

^{31}P NMR (202 MHz)

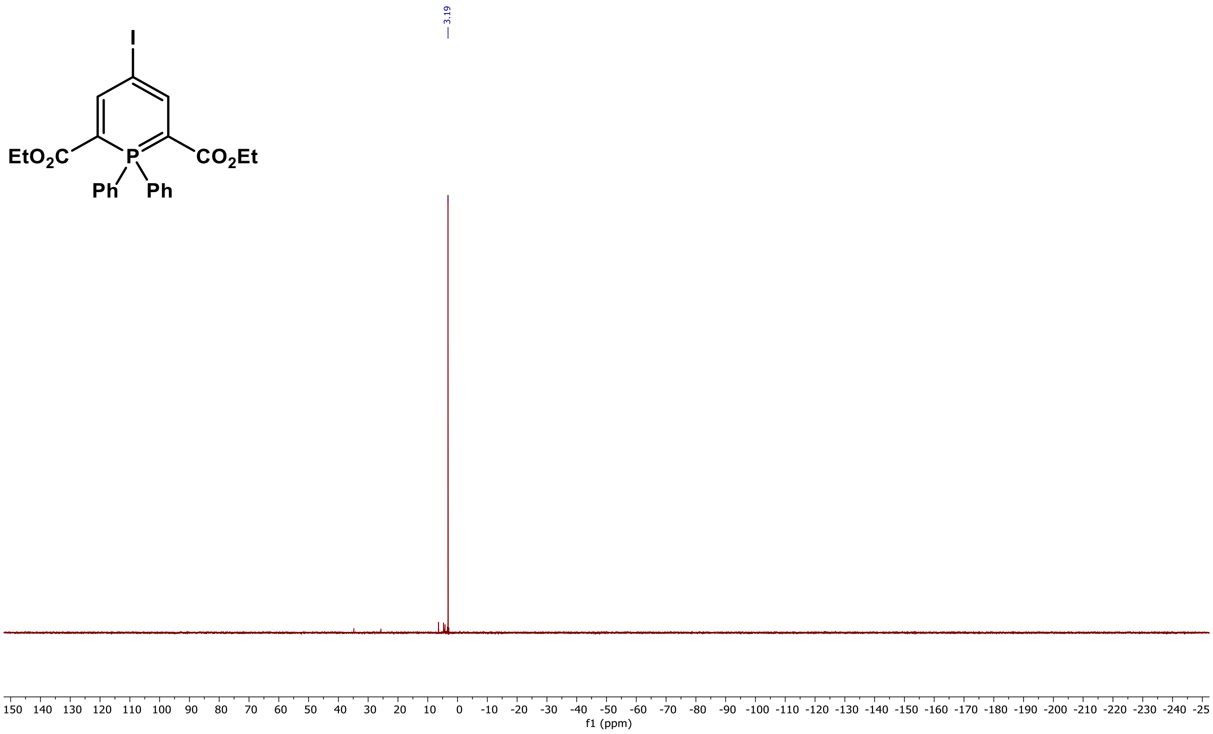

I/Zn Exchange

Allylation, Acylation and Other Functionalizations

4-Allyl-1,1-diphenyl-1λ^5-phosphinine-2,6-dicarbonitrile (2a)

^{1}H NMR (500 MHz)

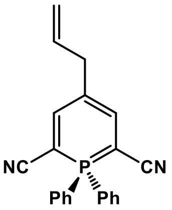

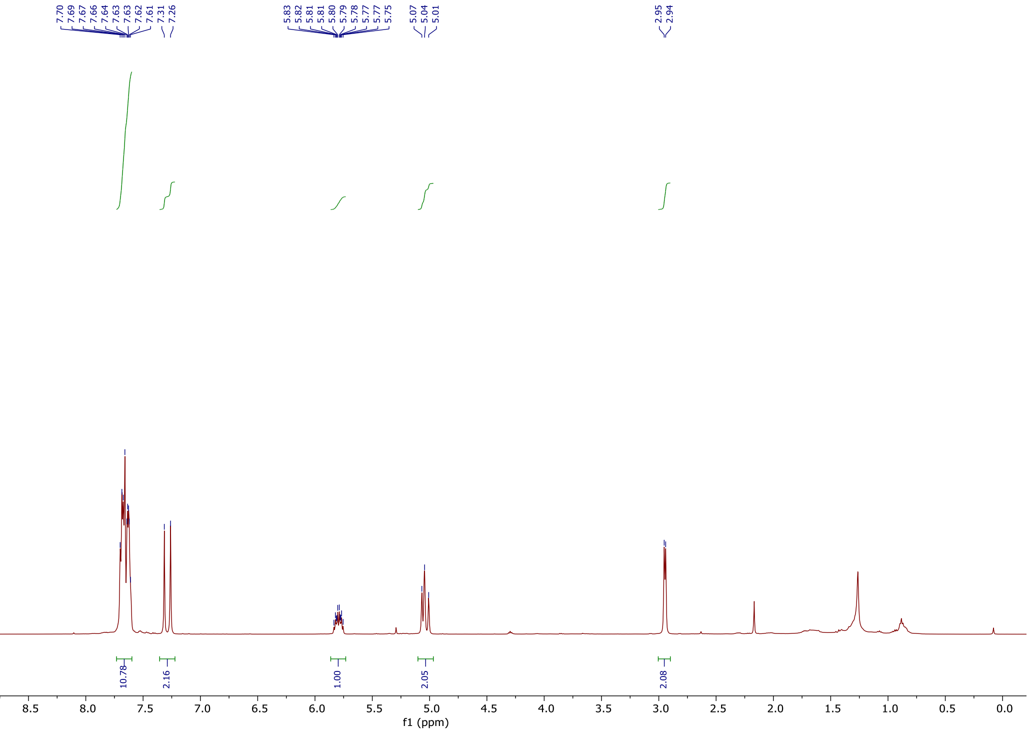

^{13}C NMR (126 MHz)

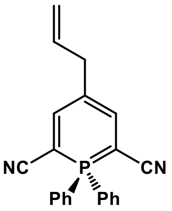

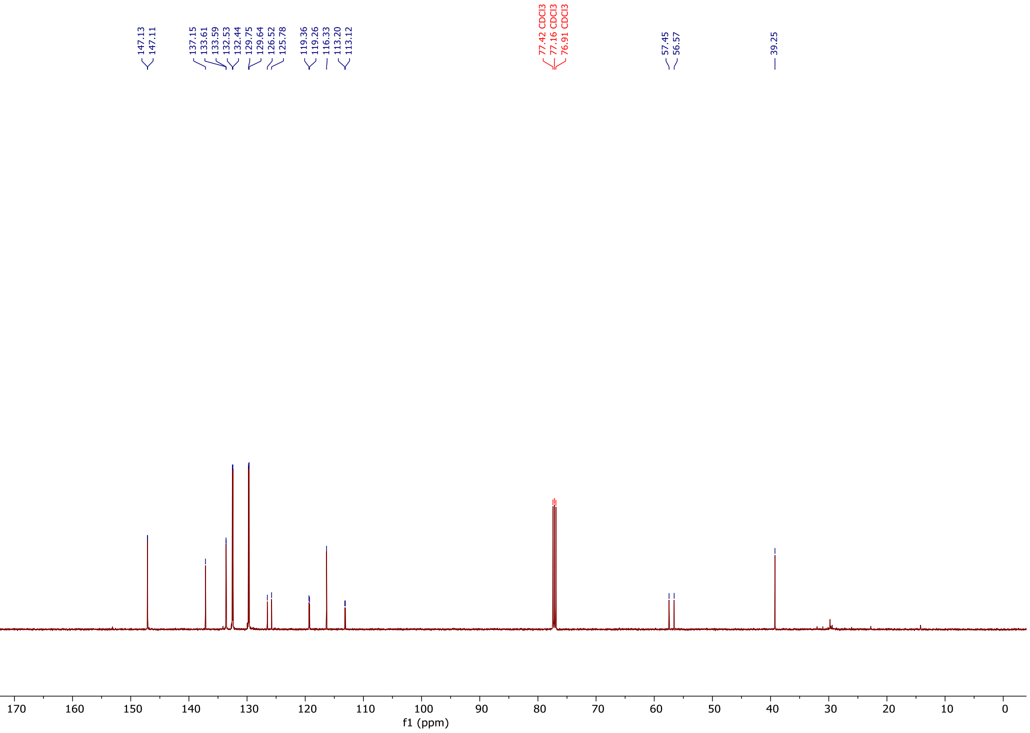

^{31}P NMR (202 MHz)

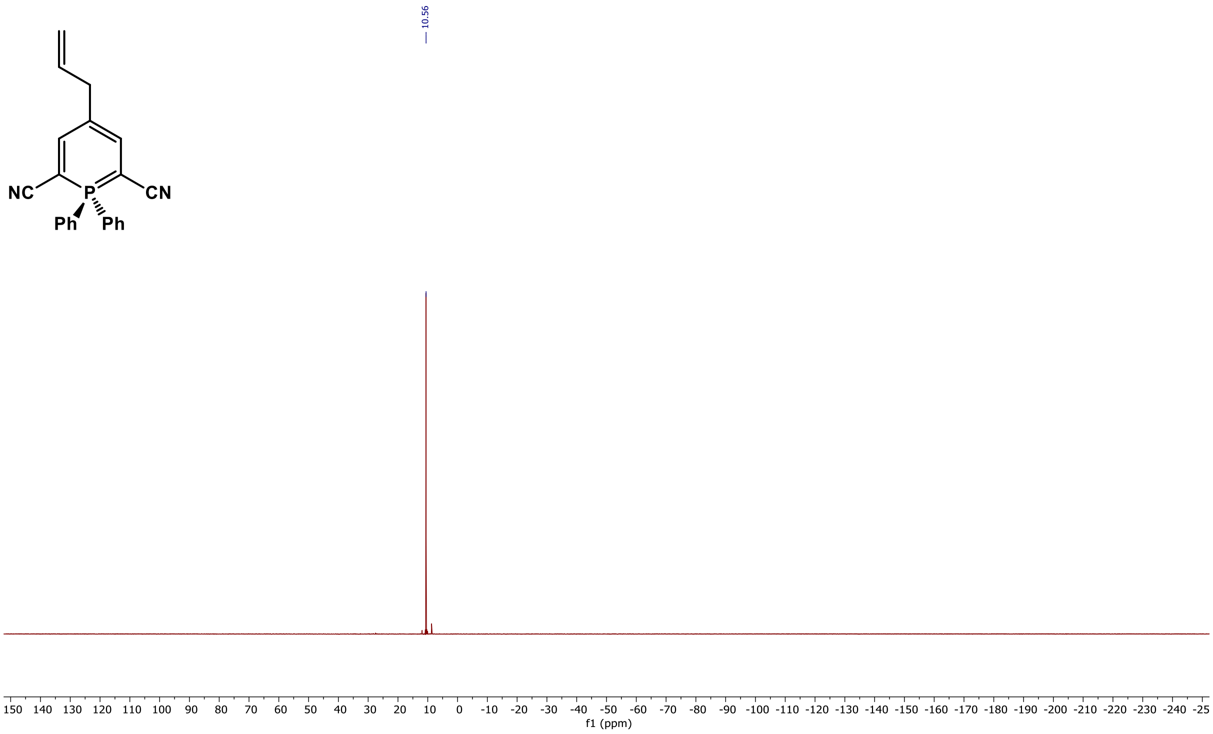

4-(Cyclohex-2-en-1-yl)-1,1-diphenyl-1λ^5-phosphinine-2,6-dicarbonitrile (2b)

^{1}H NMR (500 MHz)

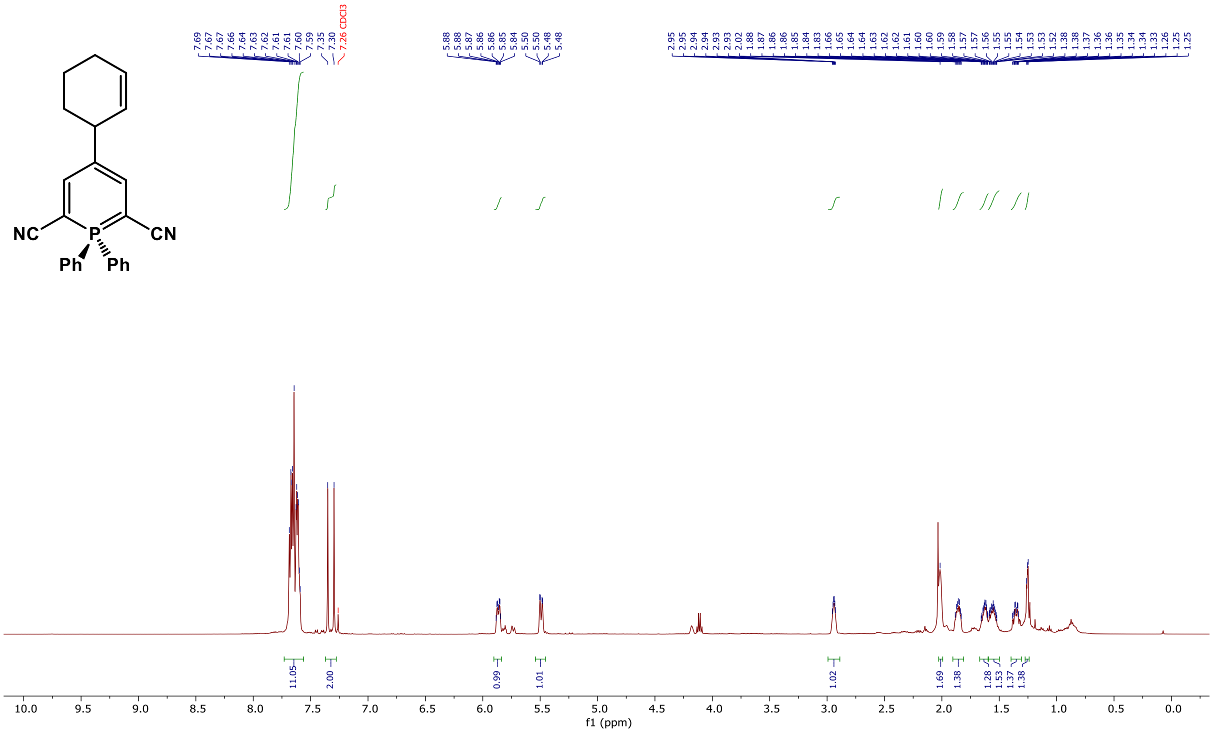

^{13}C NMR (126 MHz)

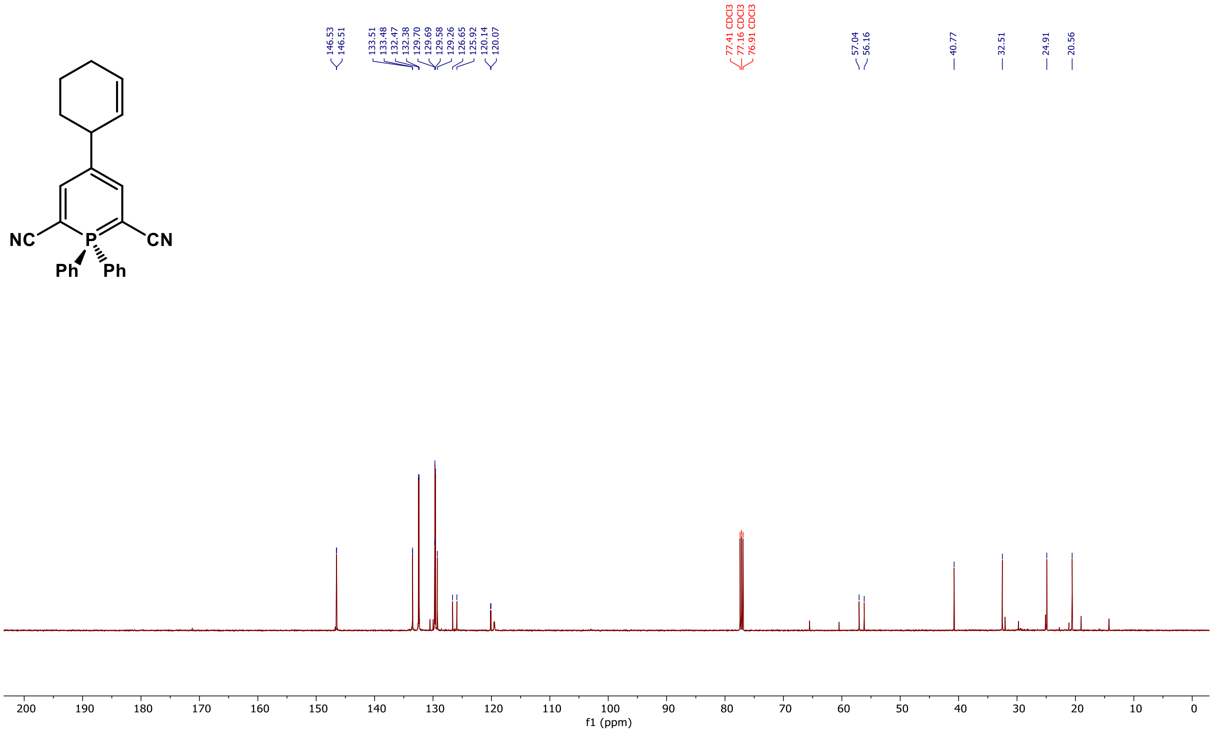

^{31}P NMR (202 MHz)

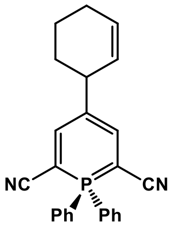

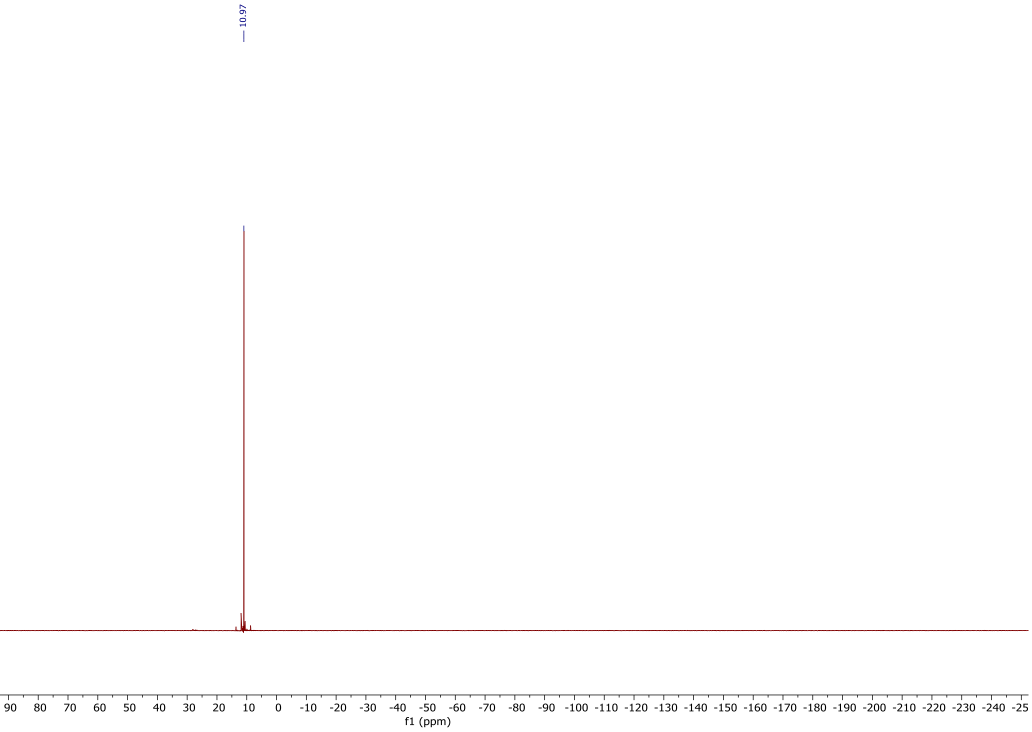

4-(2-Bromoallyl)-1,1-diphenyl-1λ[5]-phosphinine-2,6-dicarbonitrile (2c)

^{1}H NMR (500 MHz)

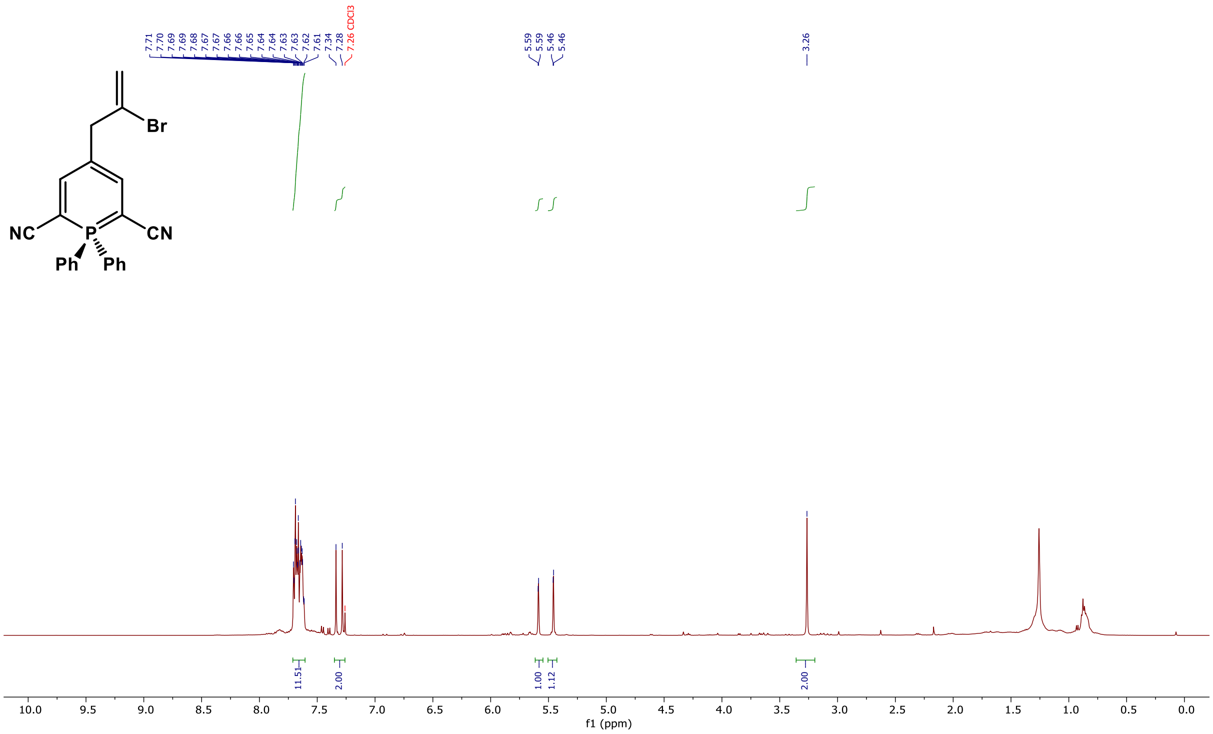

^{13}C NMR (126 MHz)

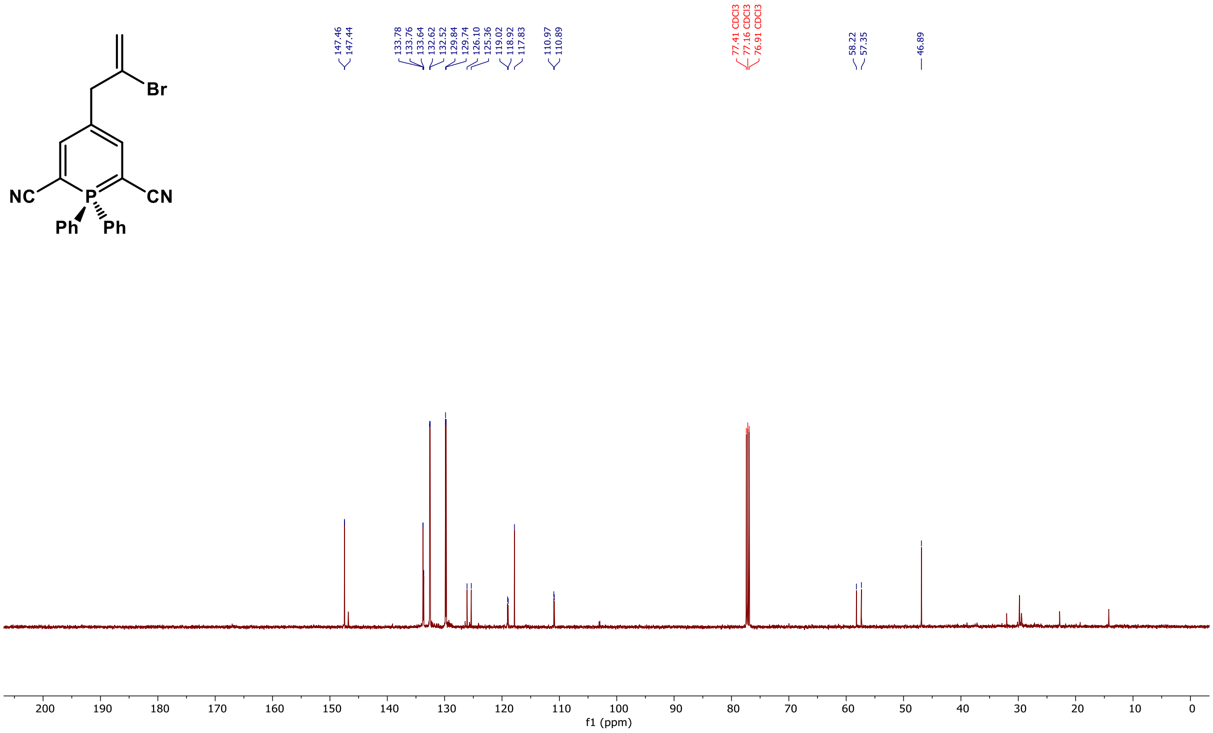

^{31}P NMR (202 MHz)

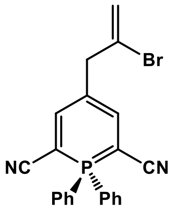

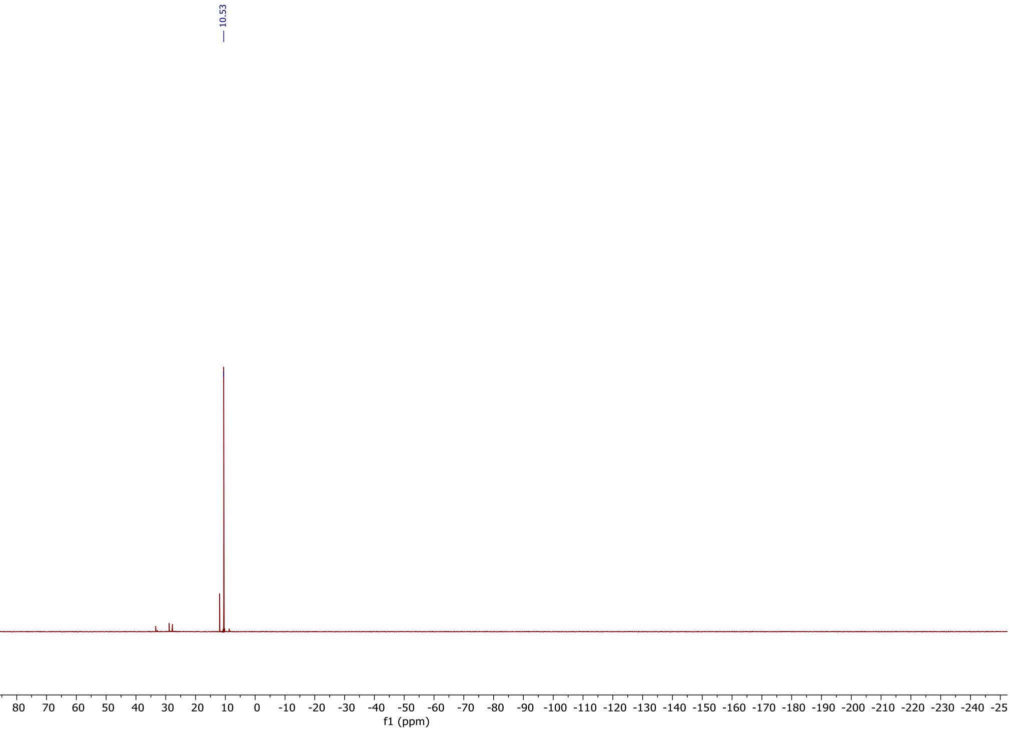

Ethyl 2-((2,6-dicyano-1,1-diphenyl-1λ^5-phosphinin-4-yl)methyl)acrylate (2d)

^{1}H NMR (500 MHz)

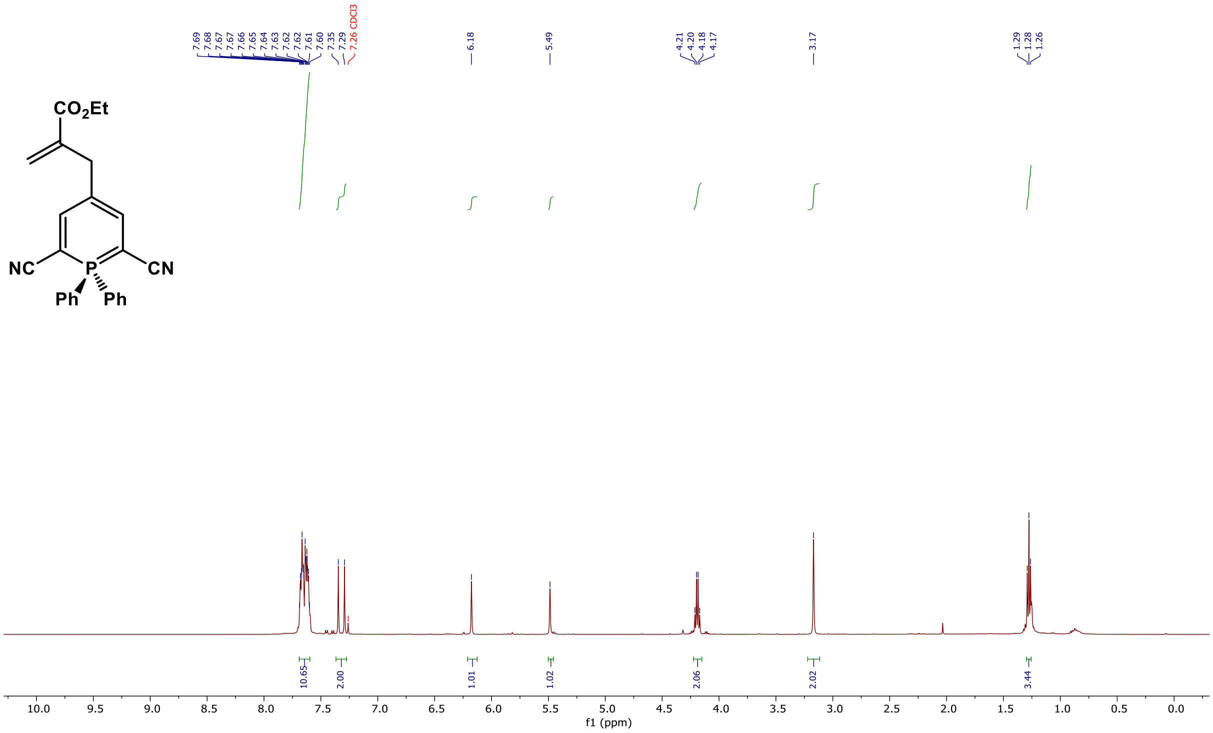

^{13}C NMR (126 MHz)

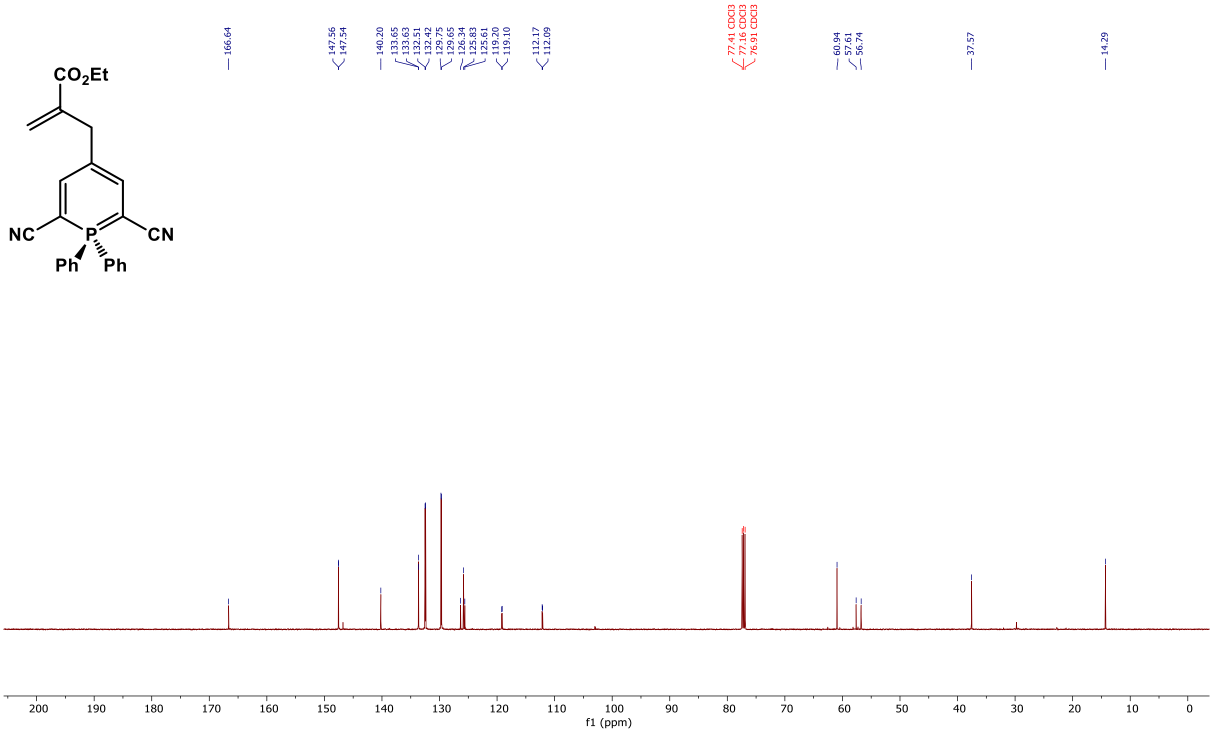

^{31}P NMR (202 MHz)

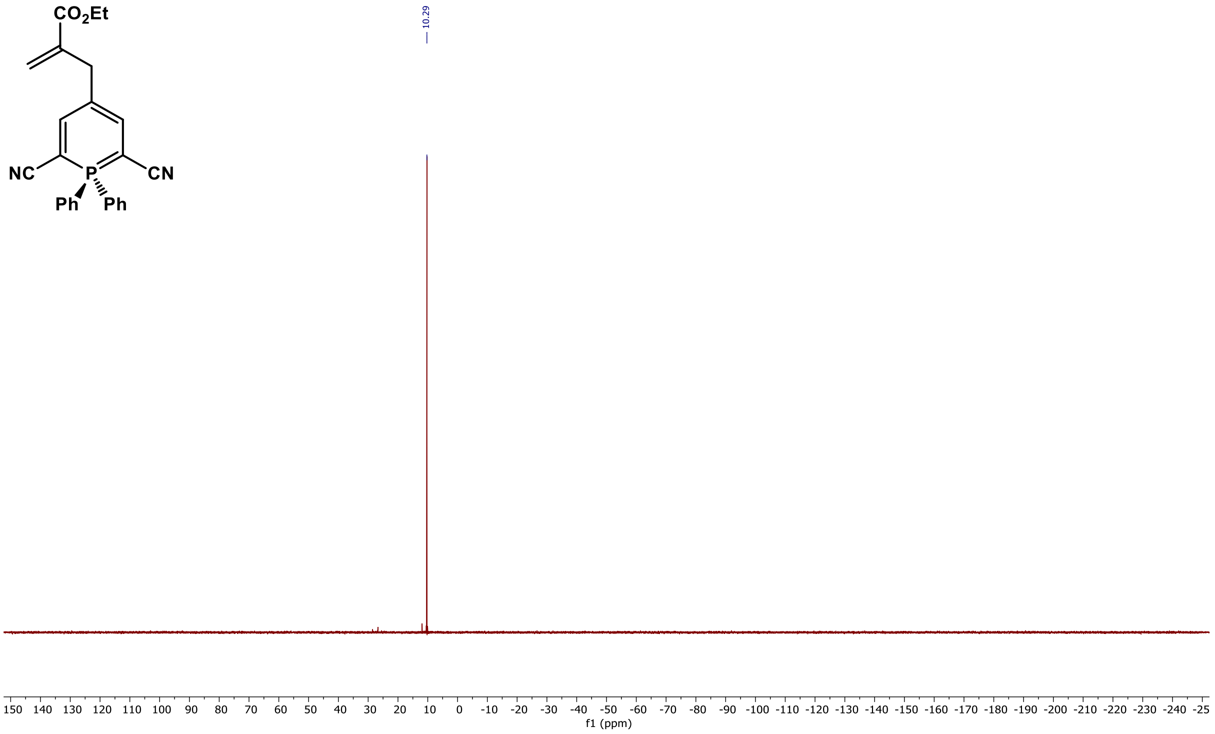

diethyl 4-(2-(ethoxycarbonyl)allyl)-1,1-diphenyl-1λ^5-phosphinine-2,6-dicarboxylate (5a)

^{1}H NMR (500 MHz)

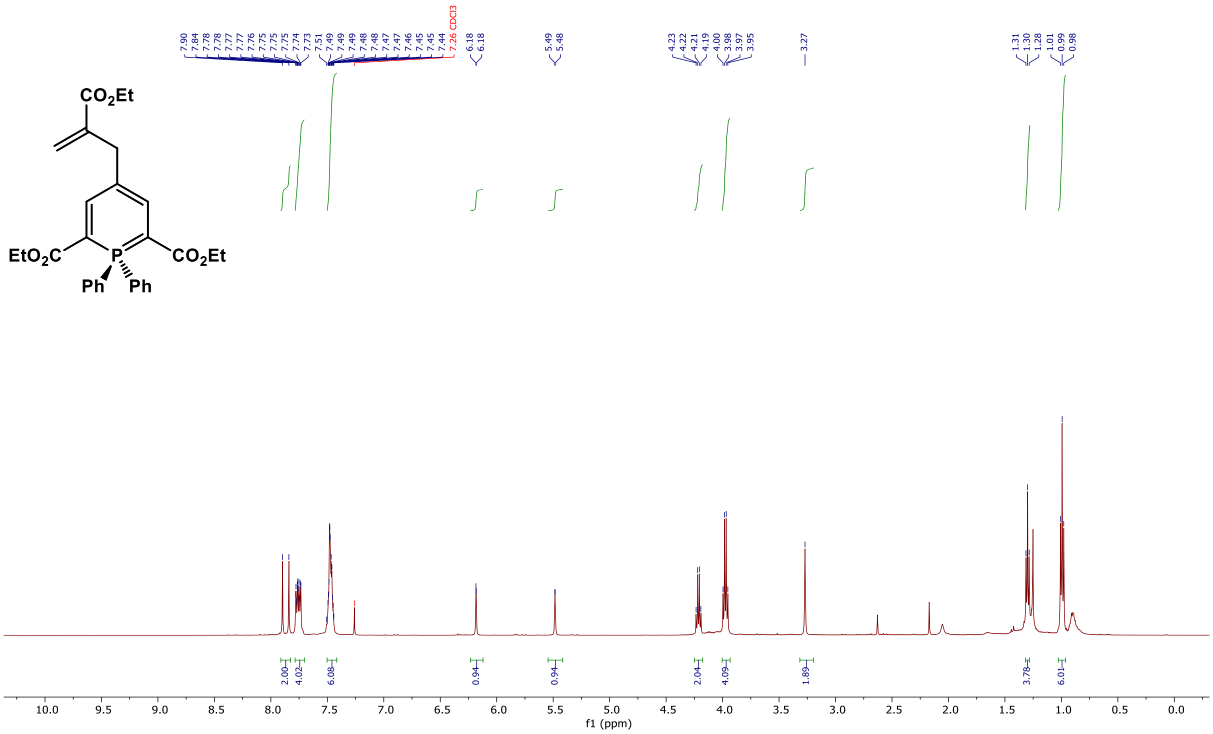

^{13}C NMR (126 MHz)

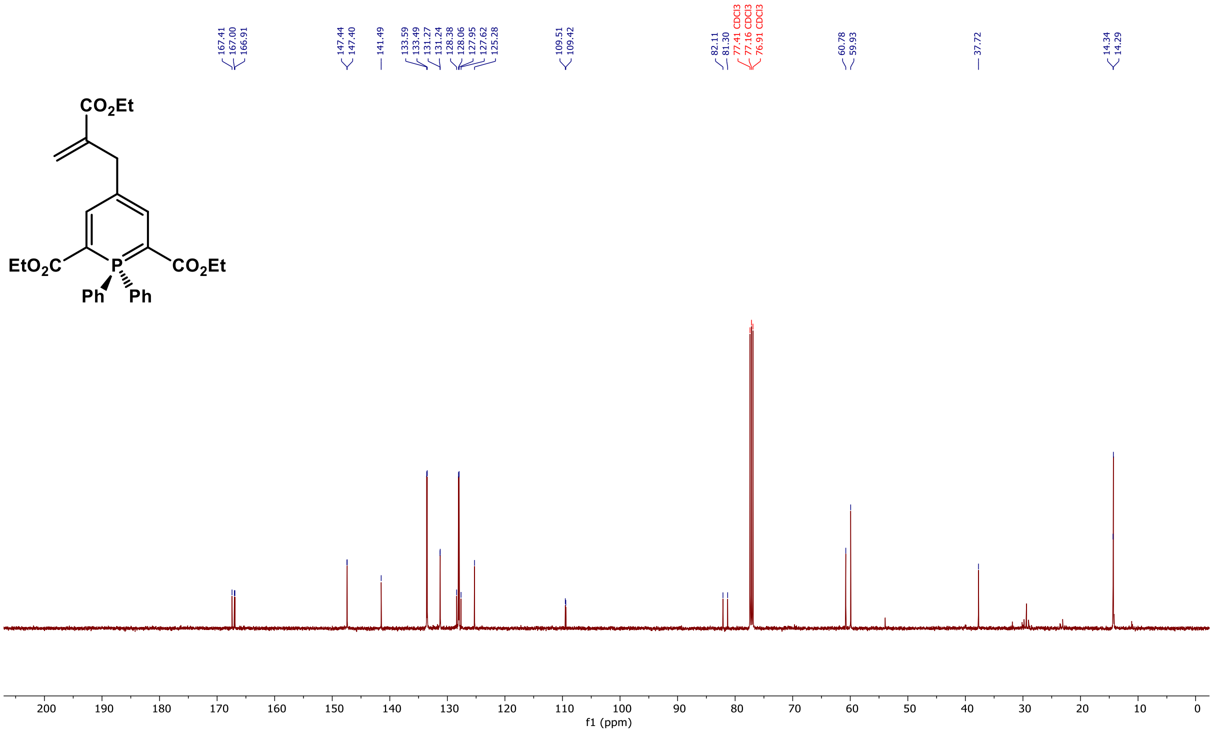

^{31}P NMR (202 MHz)

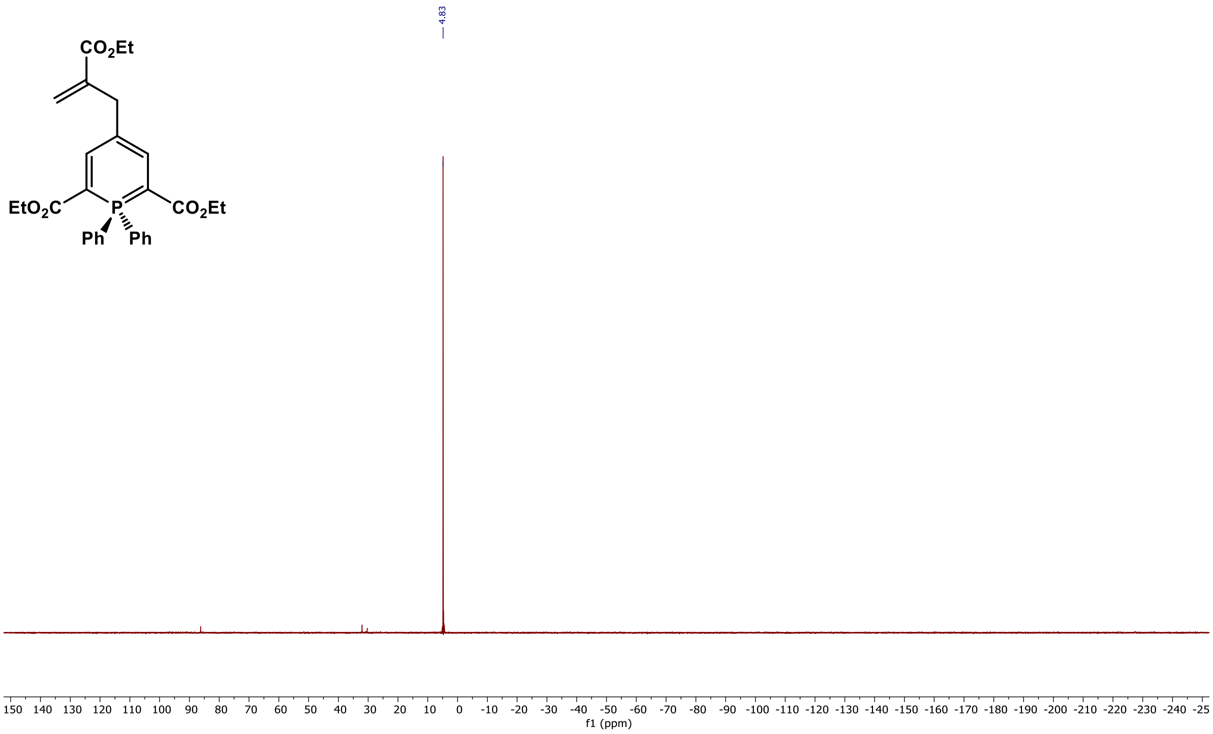

4-(Cyclopropanecarbonyl)-1,1-diphenyl-1λ[5]-phosphinine-2,6-dicarbonitrile (2e)

^{1}H NMR (500 MHz)

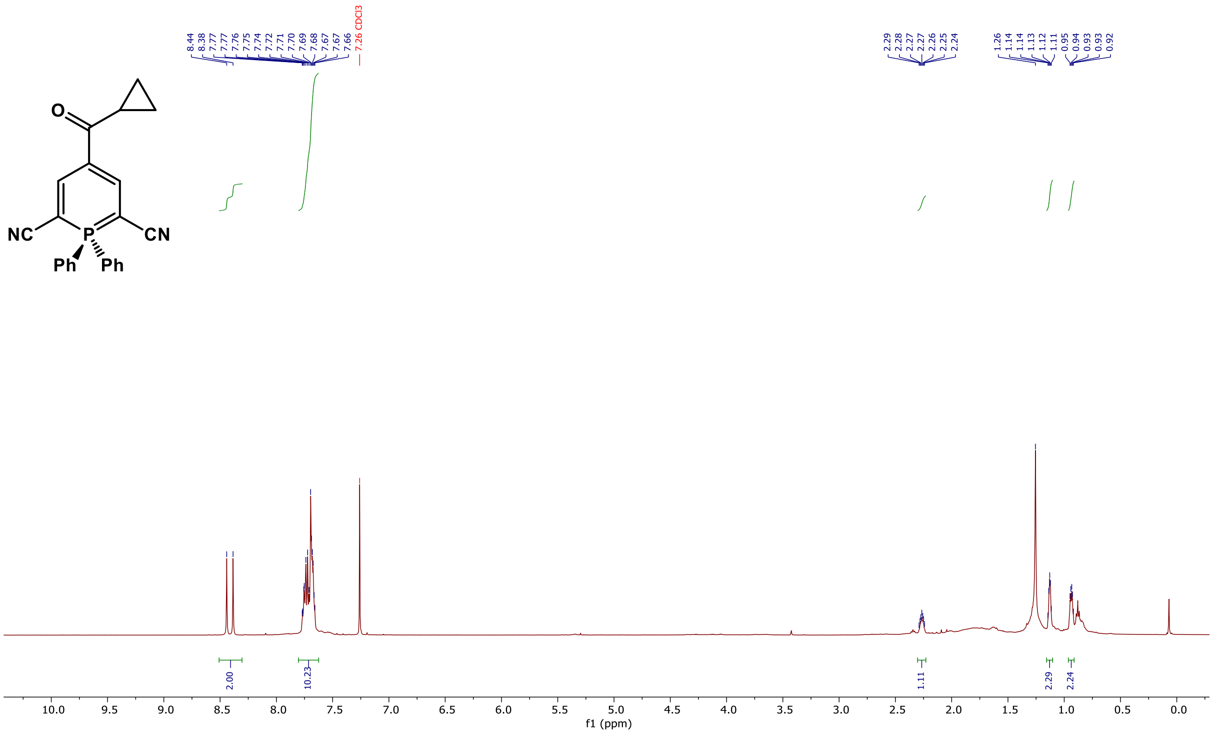

^{13}C NMR (126 MHz)

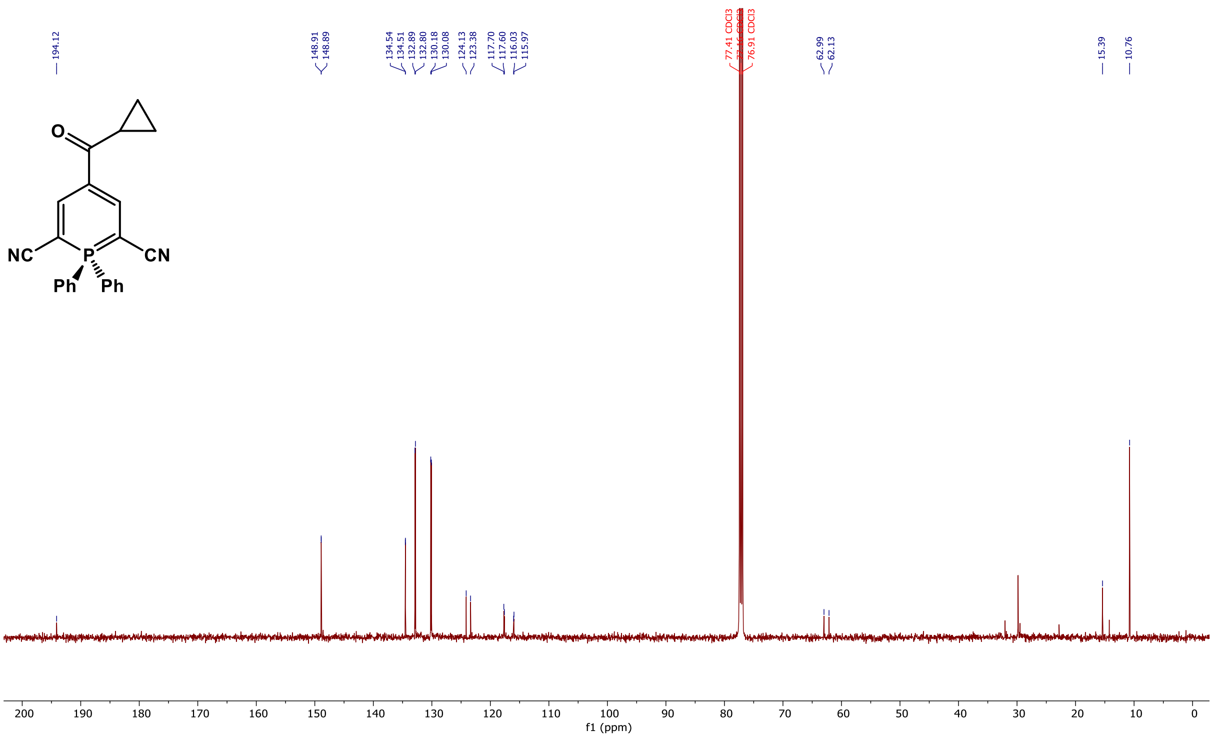

^{31}P NMR (202 MHz)

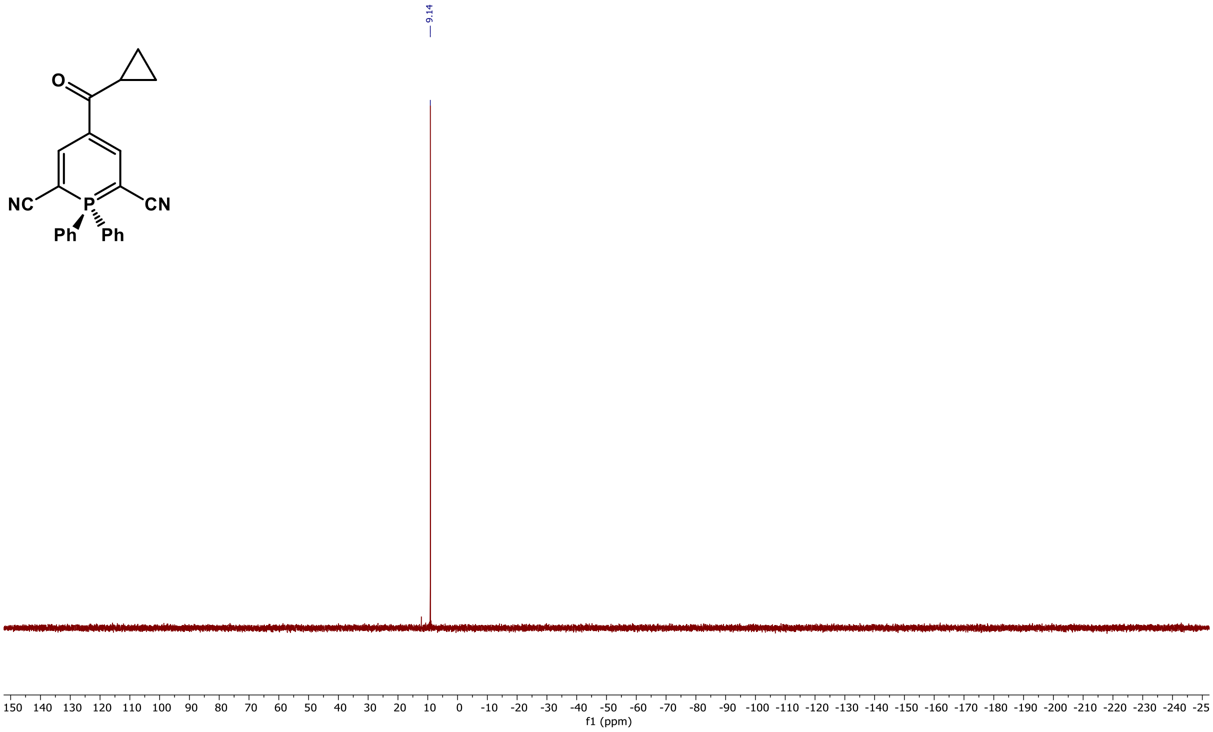

4-(Cyclobutanecarbonyl)-1,1-diphenyl-1λ5-phosphinine-2,6-dicarbonitrile (2f)

^{1}H NMR (500 MHz)

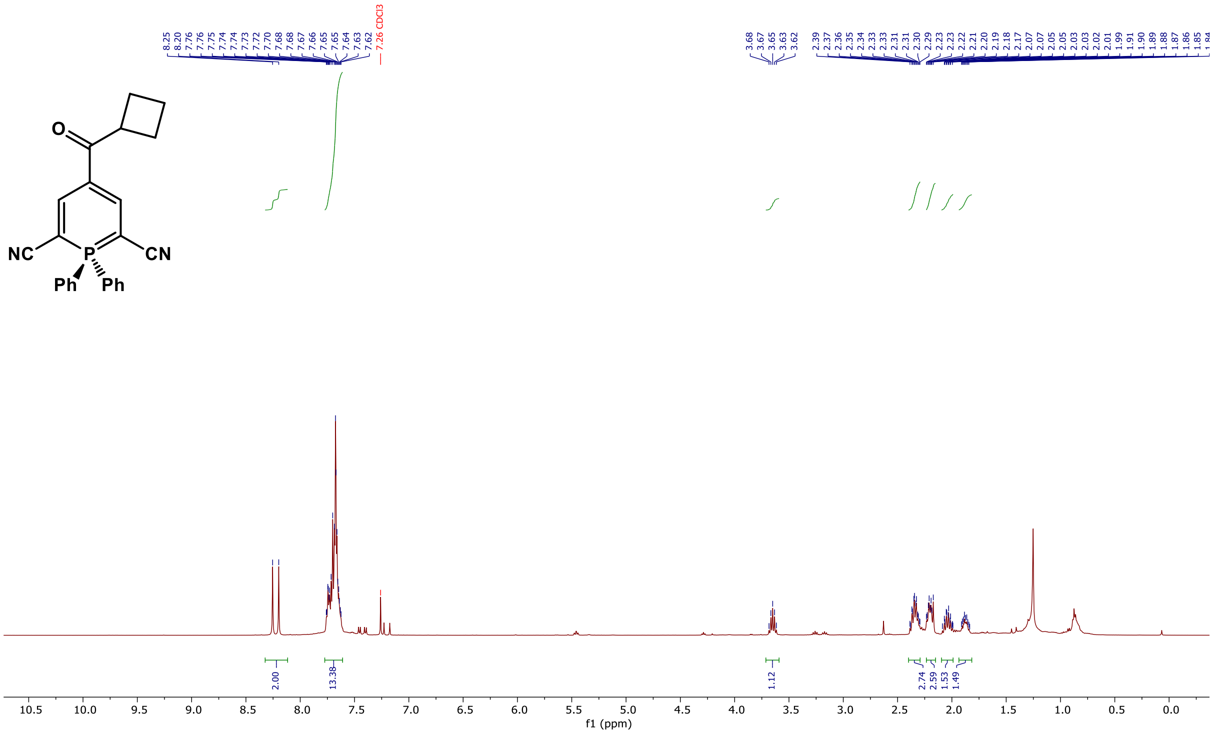

^{13}C NMR (126 MHz)

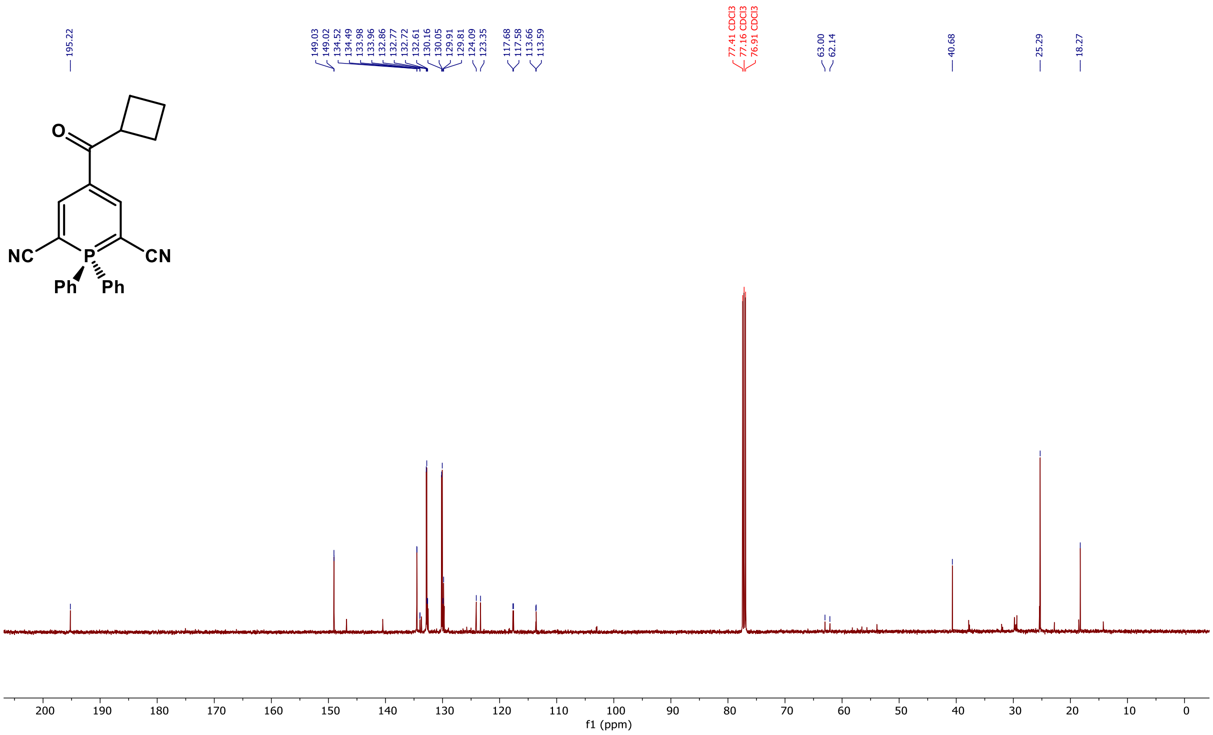

^{31}P NMR (202 MHz)

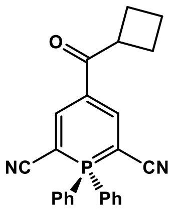

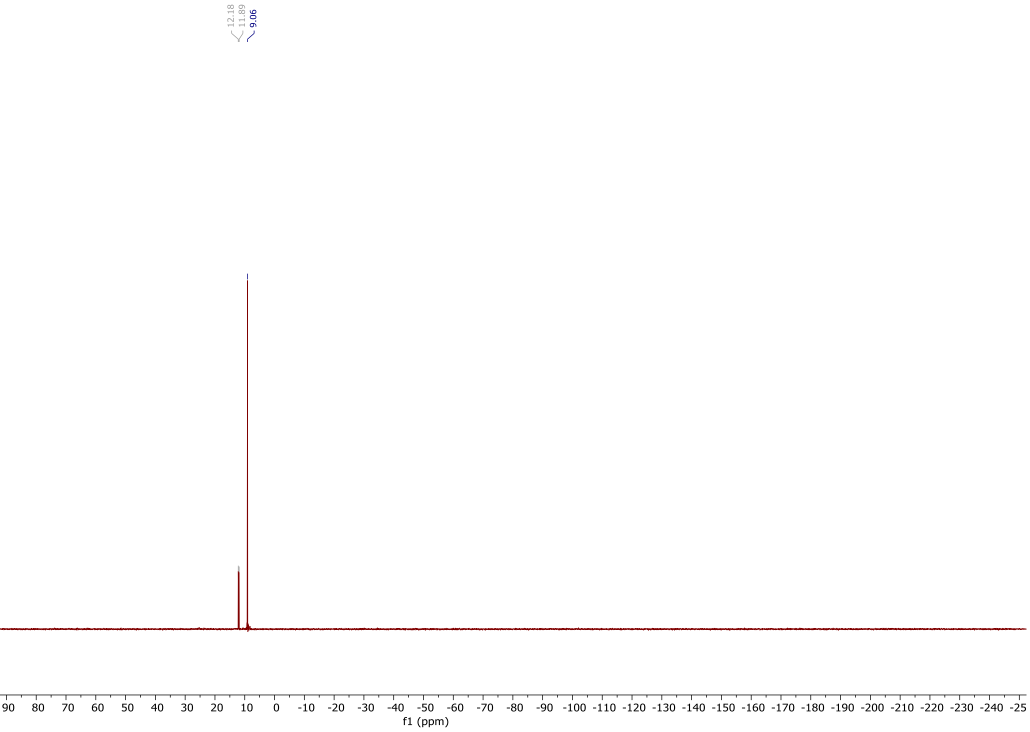

4-(2-Chloro-4-fluorobenzoyl)-1,1-diphenyl-1λ⁵-phosphinine-2,6-dicarbonitrile (2g)

^{1}H NMR (500 MHz)

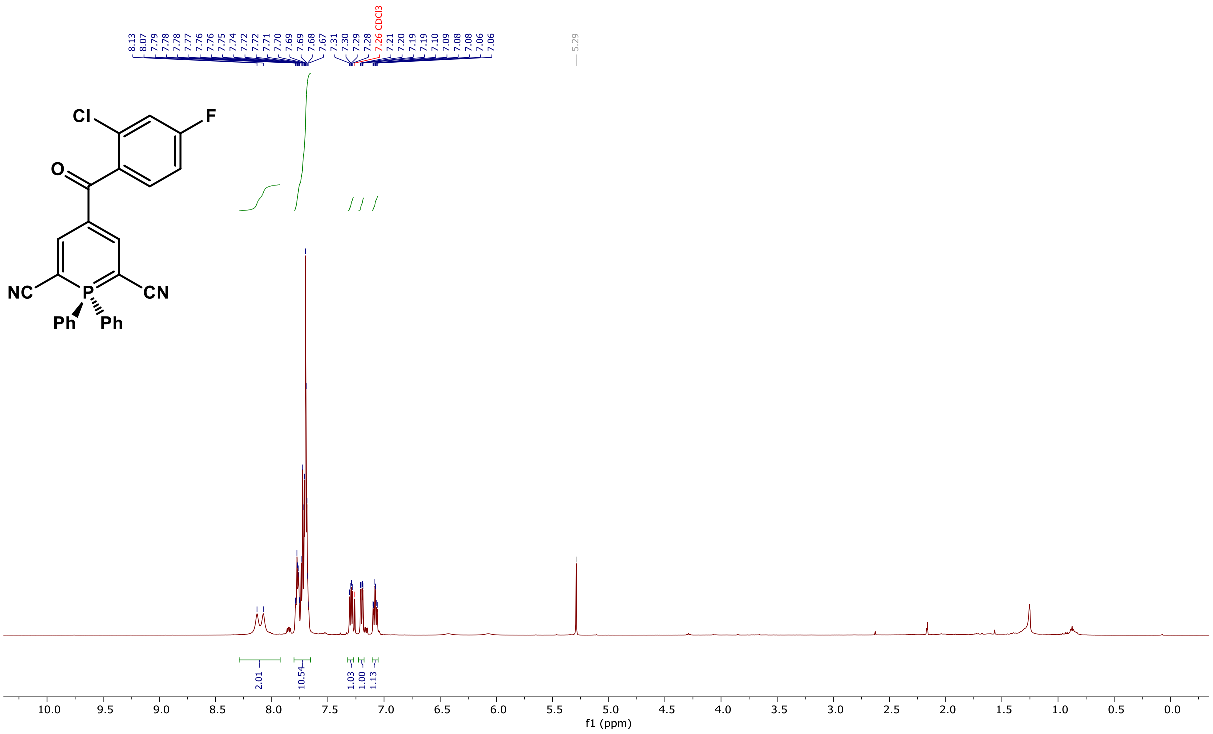

^{13}C NMR (126 MHz)

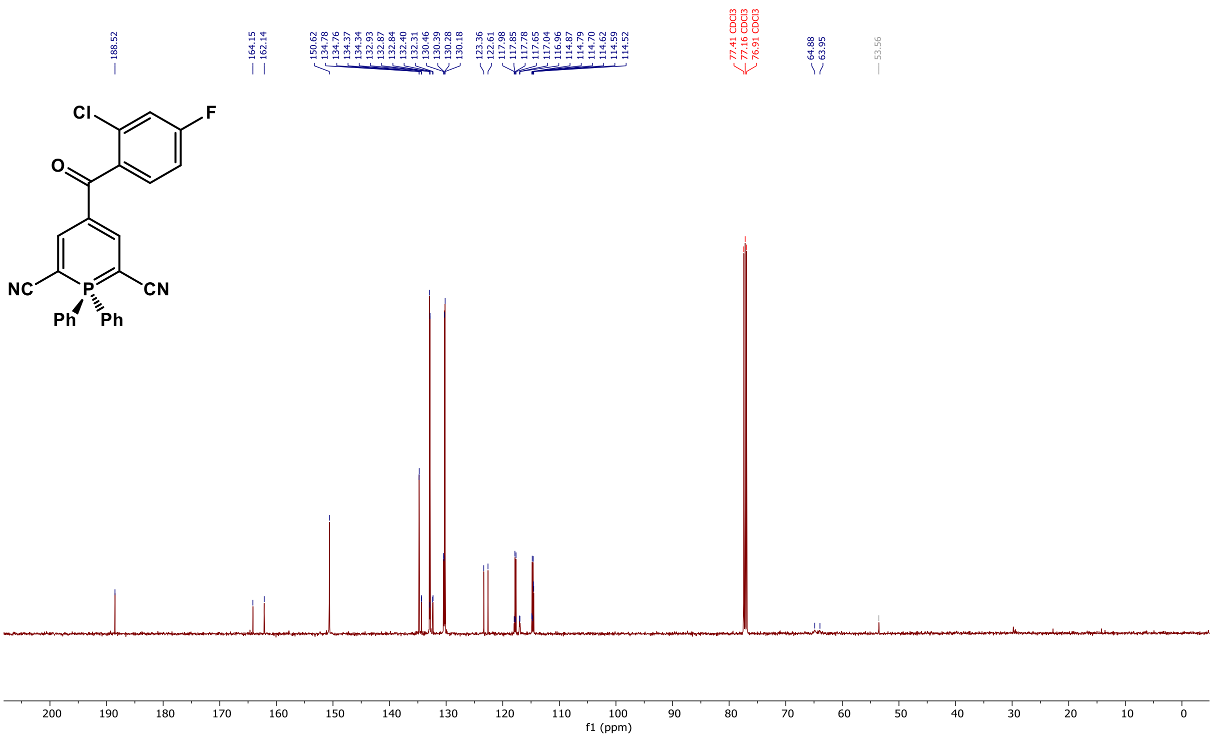

^{31}P NMR (202 MHz)

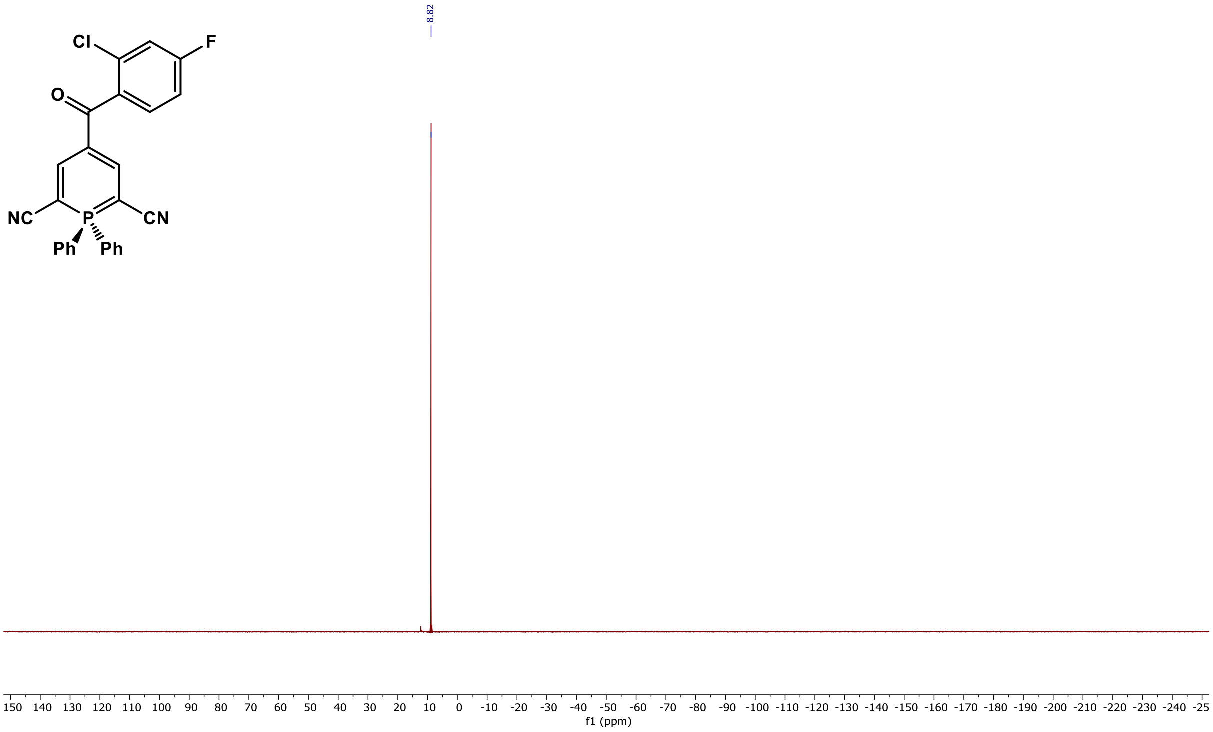

^{19}F NMR (471 MHz)

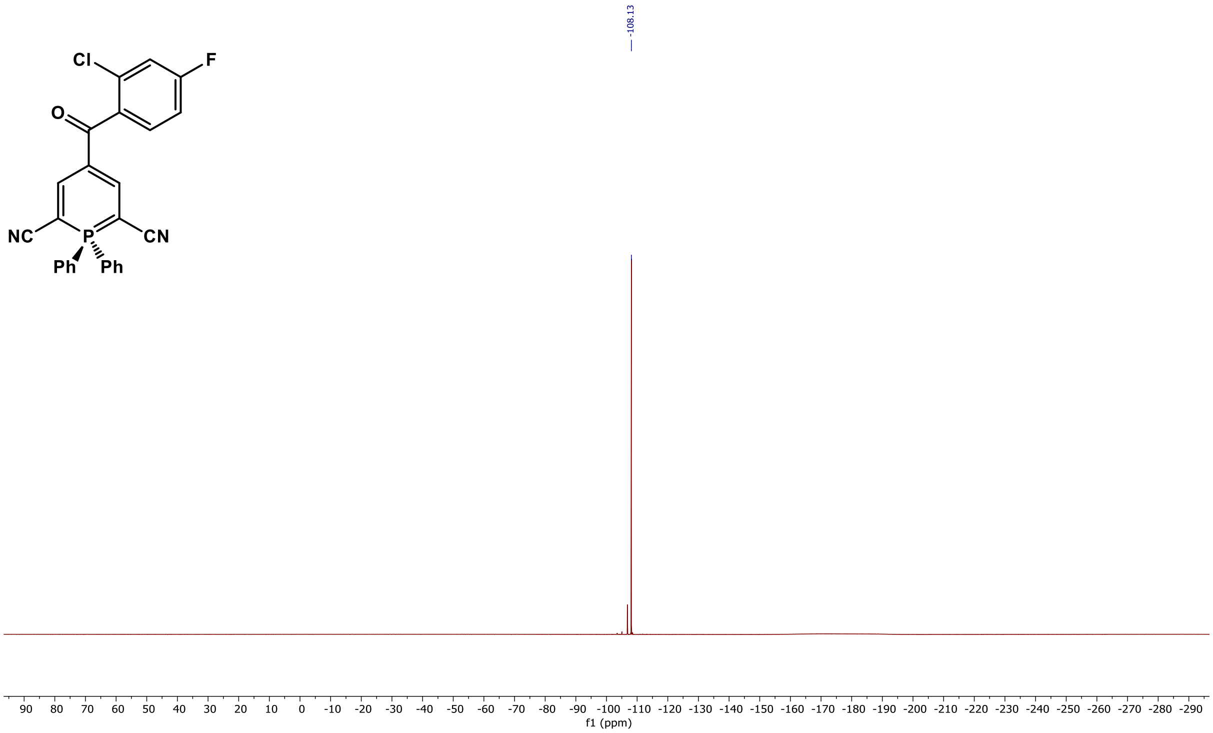

4-(5-Chlorothiophene-2-carbonyl)-1,1-diphenyl-1λ^5-phosphinine-2,6-dicarbonitrile (2h)

^{1}H NMR (500 MHz)

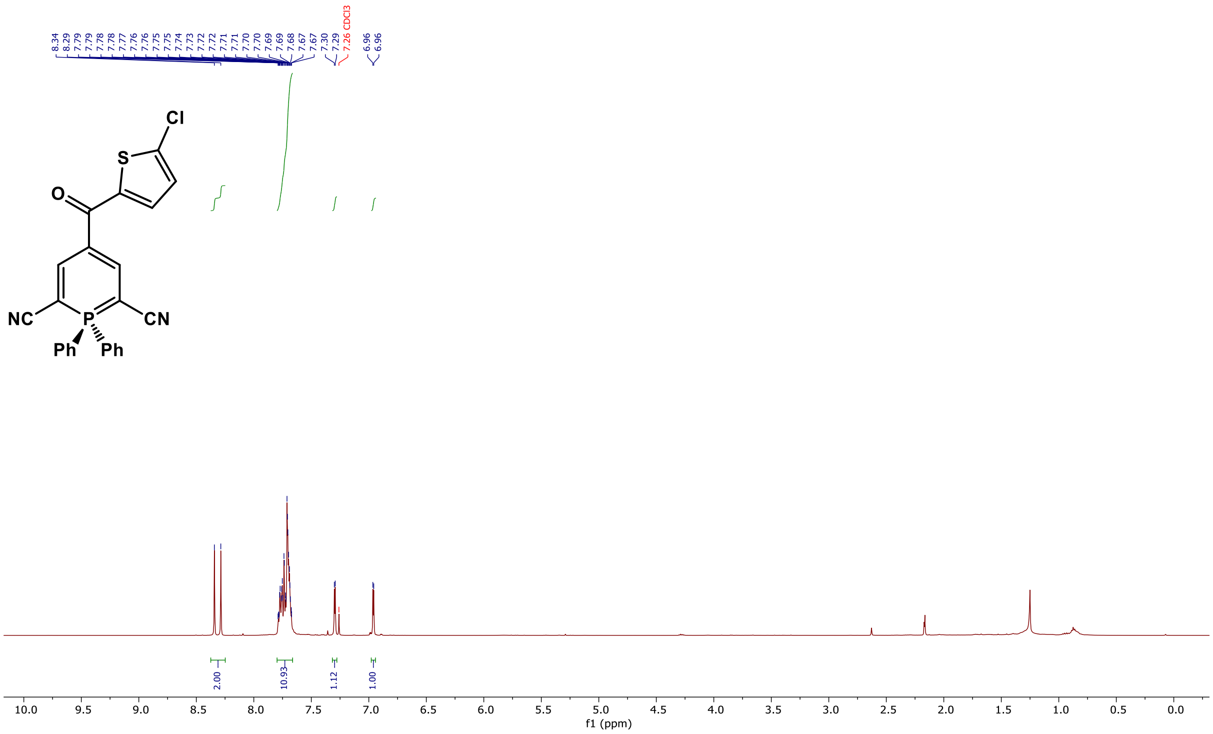

^{13}C NMR (126 MHz)

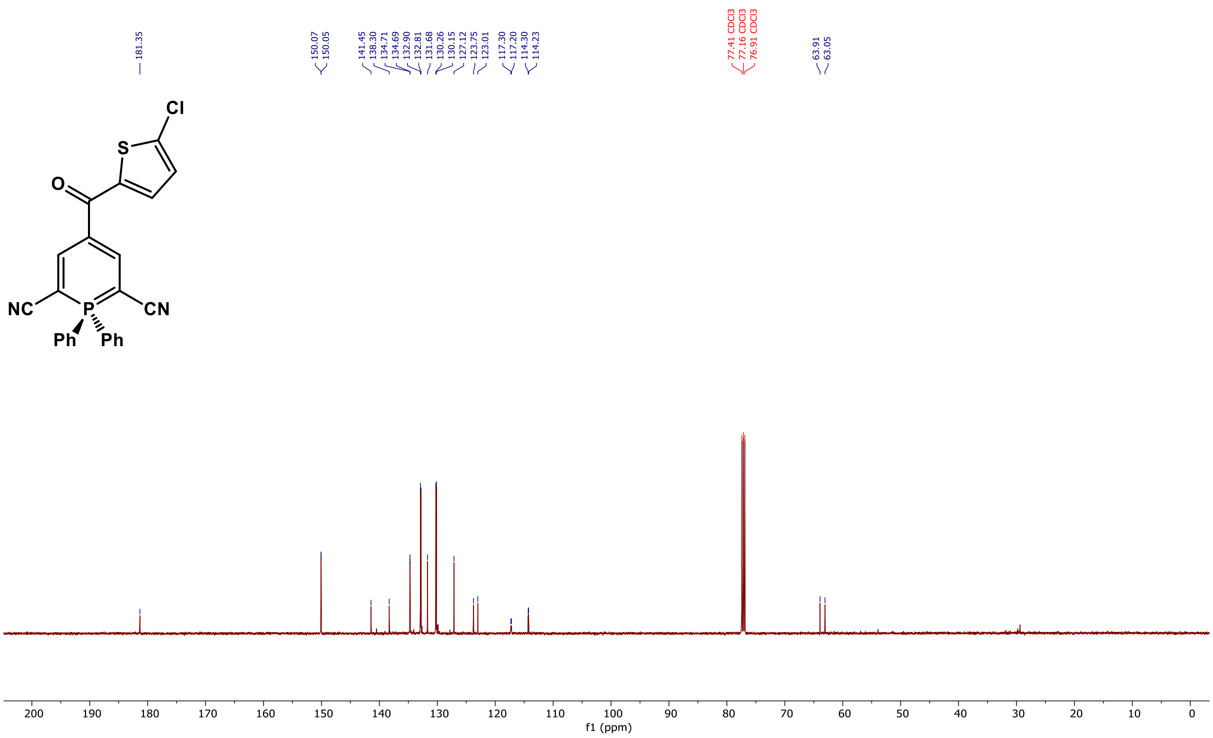

^{31}P NMR (202 MHz)

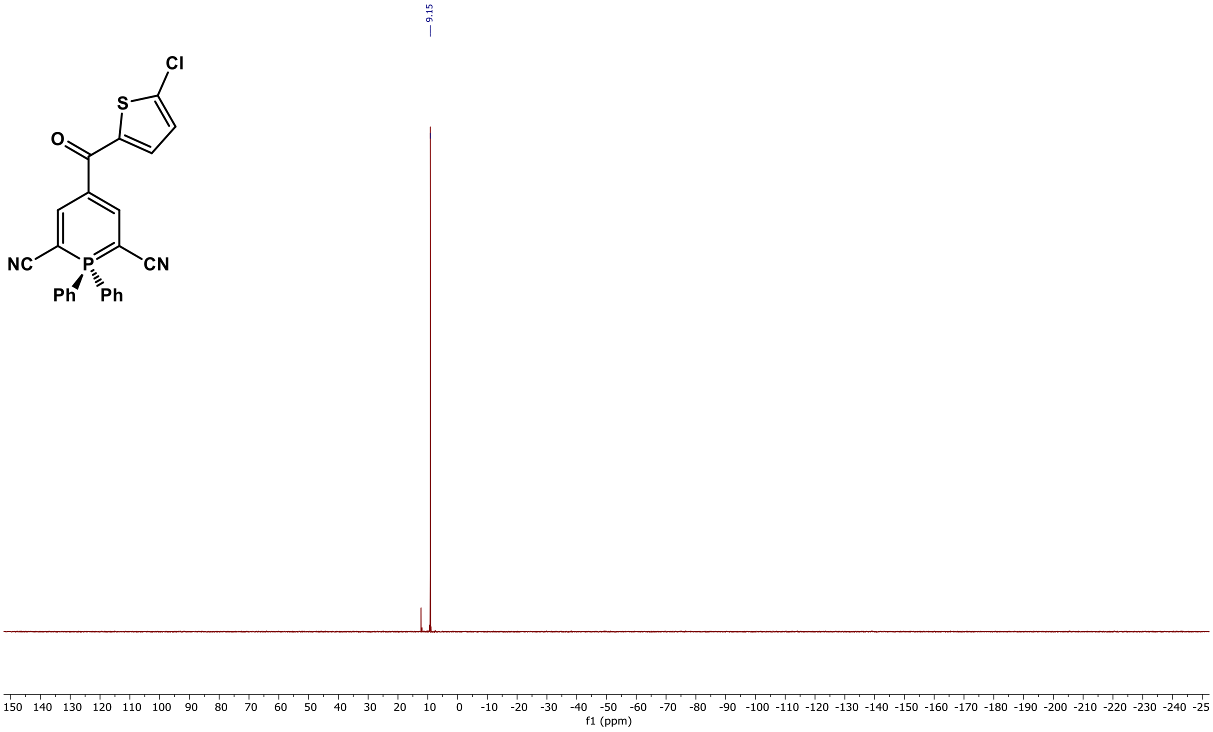

diethyl 4-benzoyl-1,1-diphenyl-1λ^5-phosphinine-2,6-dicarboxylate (5b)

^{1}H NMR (500 MHz)

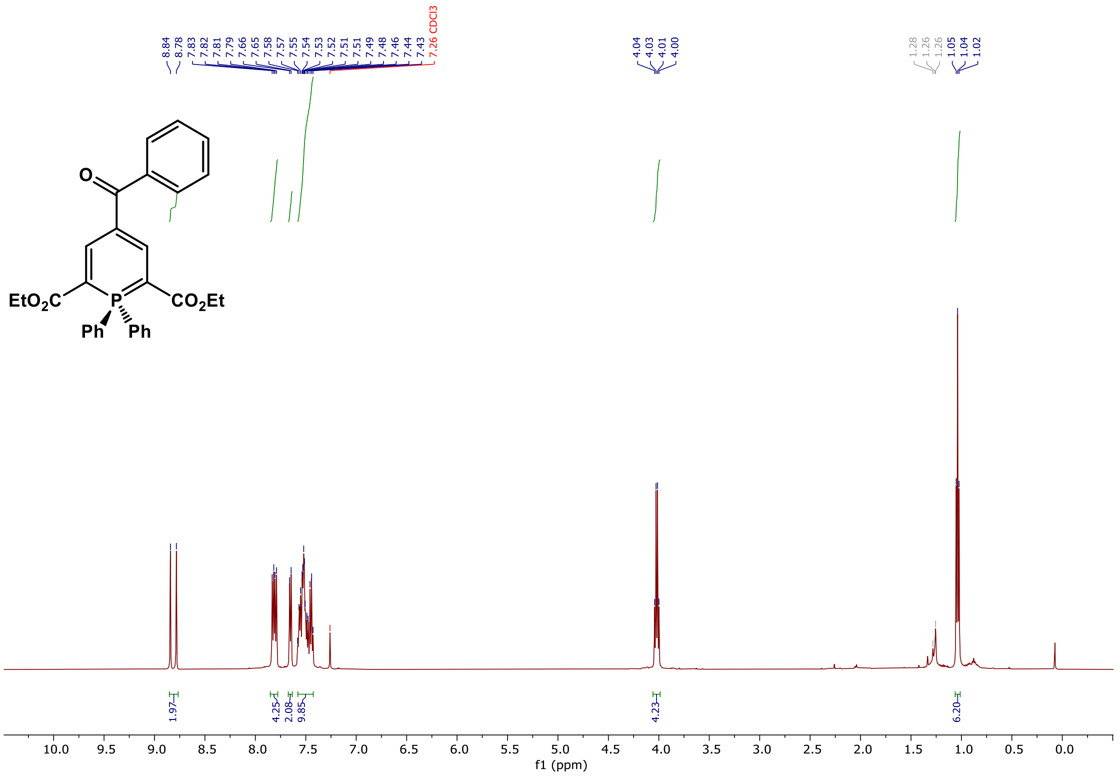

^{13}C NMR (126 MHz)

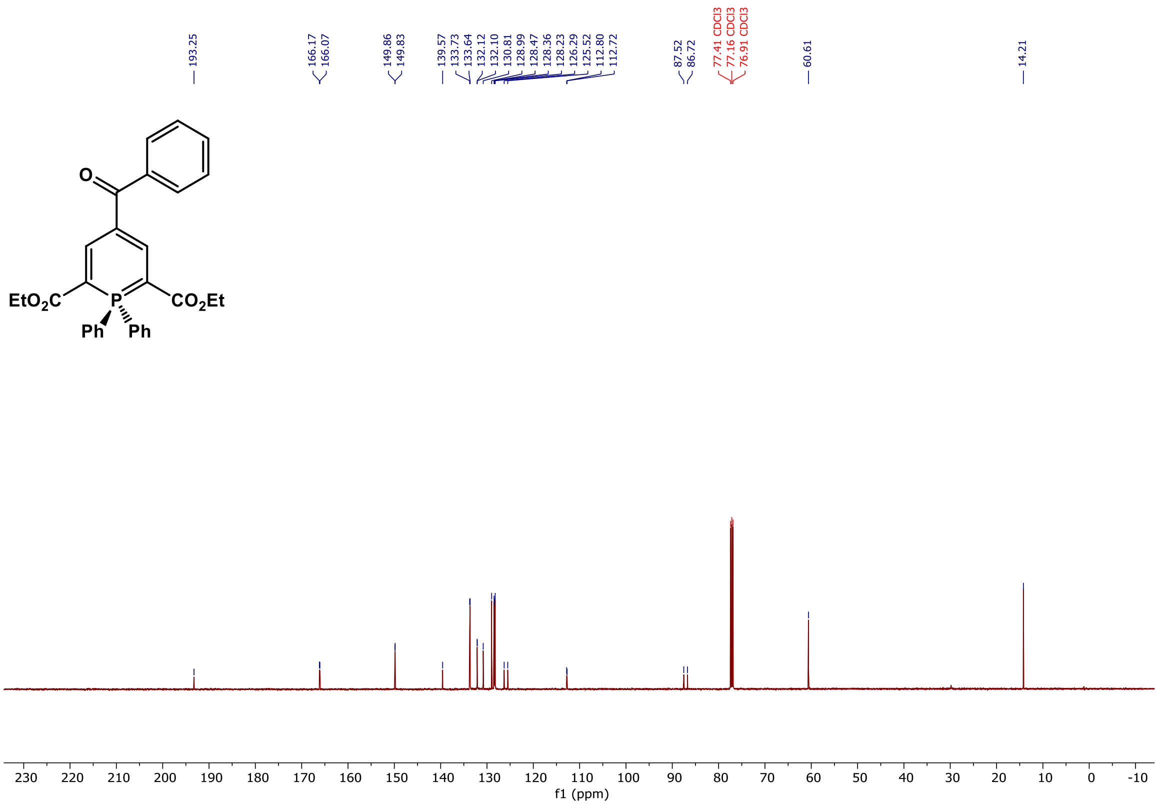

^{31}P NMR (202 MHz)

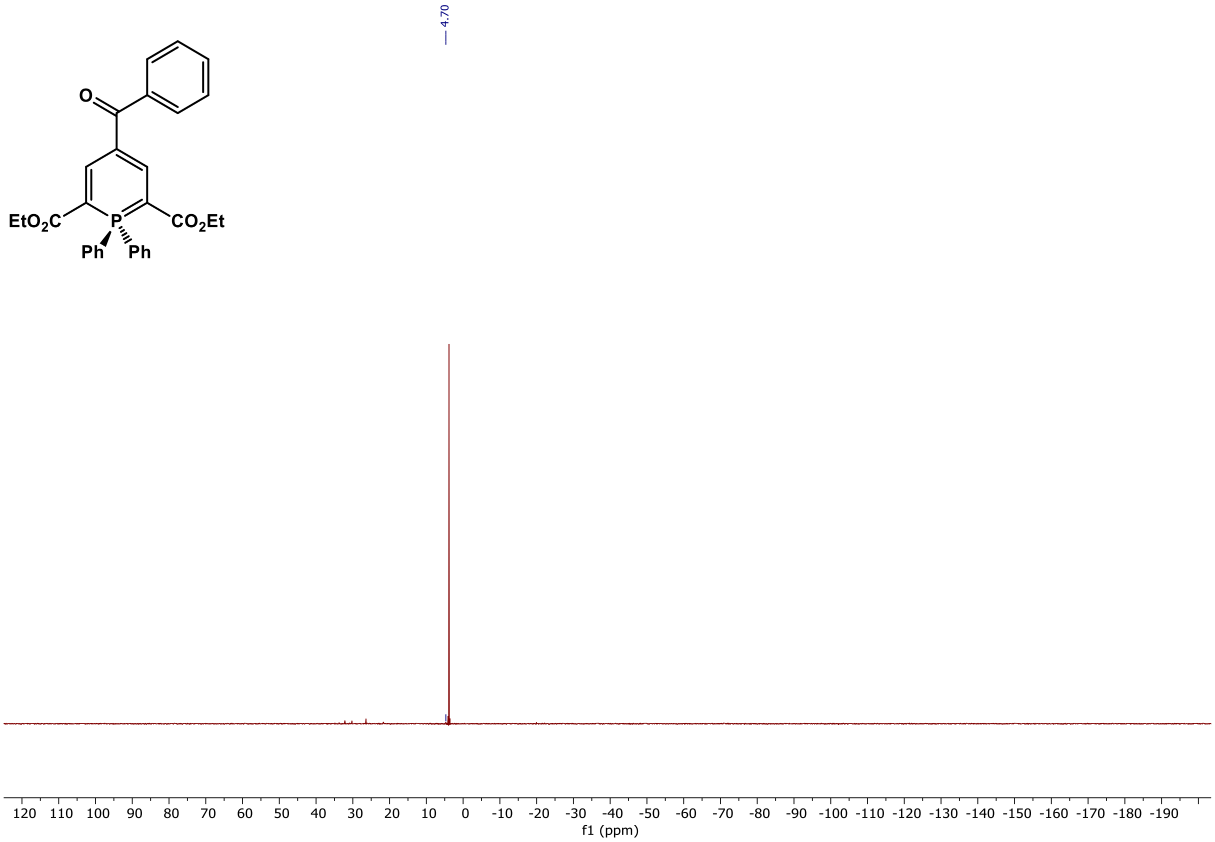

diethyl 4-(4-methoxybenzoyl)-1,1-diphenyl-1λ^5-phosphinine-2,6-dicarboxylate (5c)

^{1}H NMR (500 MHz)

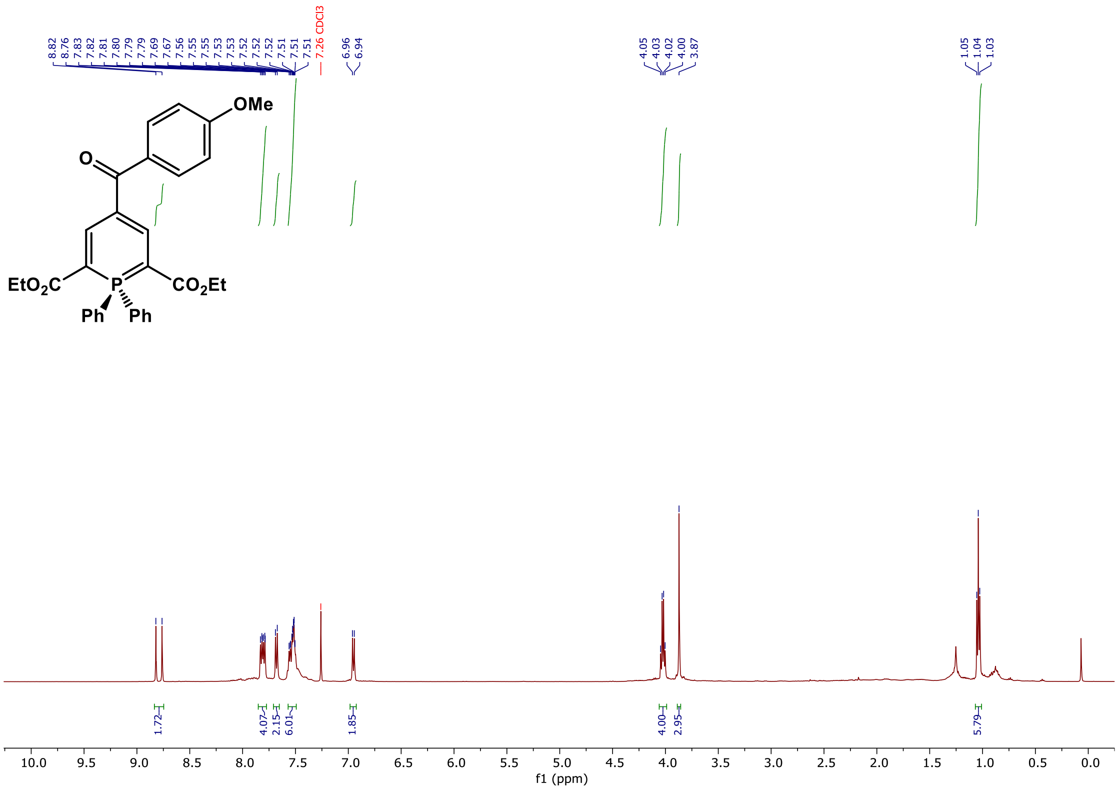

^{13}C NMR (126 MHz)

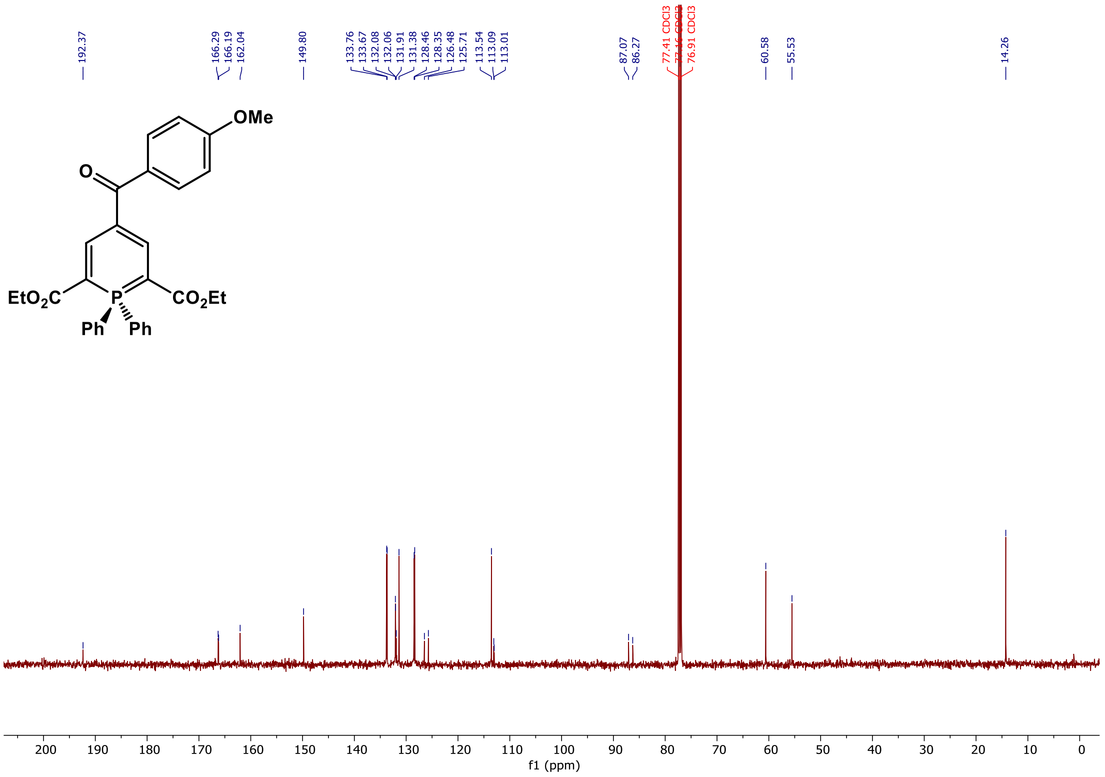

^{31}P NMR (202 MHz)

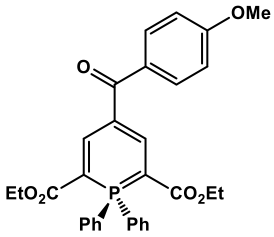

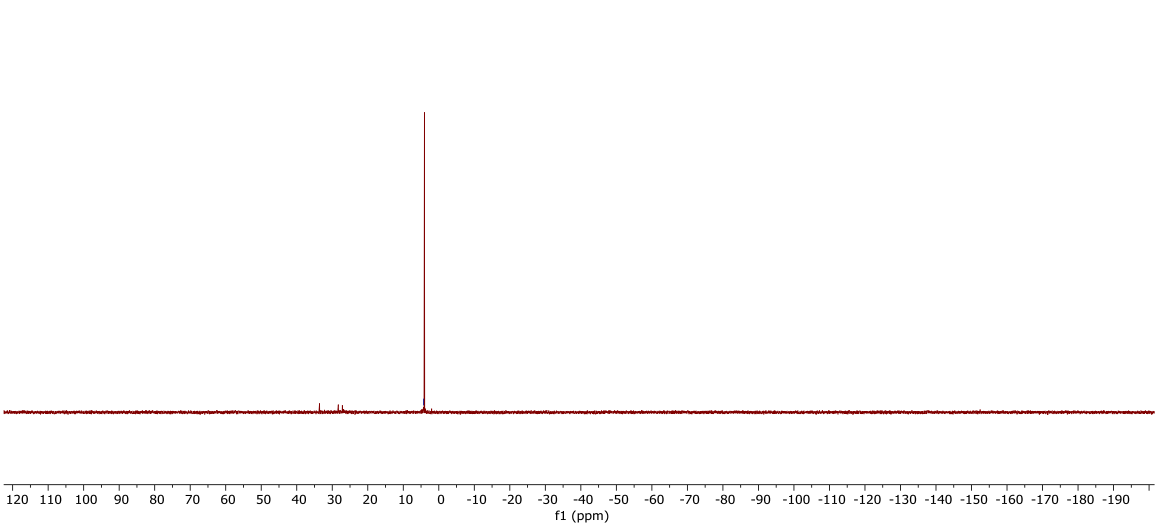

2,6-Dicyano-1,1-diphenyl-1λ[5]-phosphinine-4-carboxamide (2i)

^{1}H NMR (500 MHz)

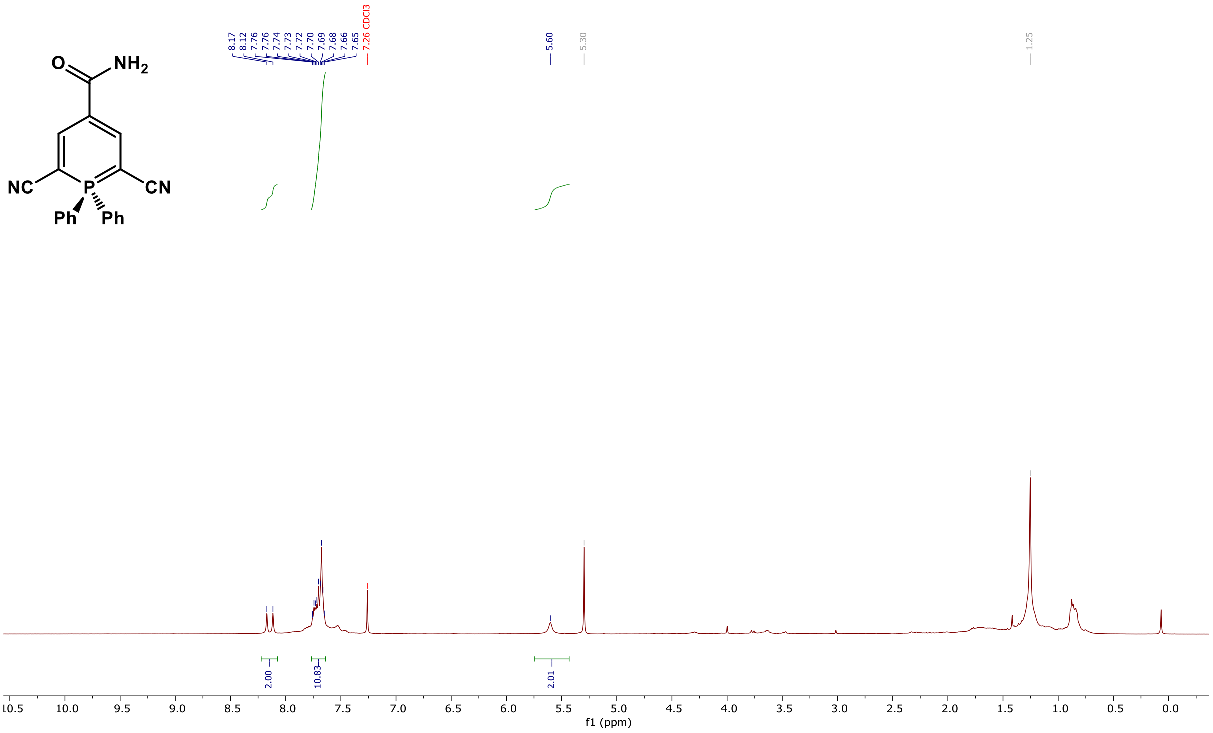

^{13}C NMR (126 MHz)

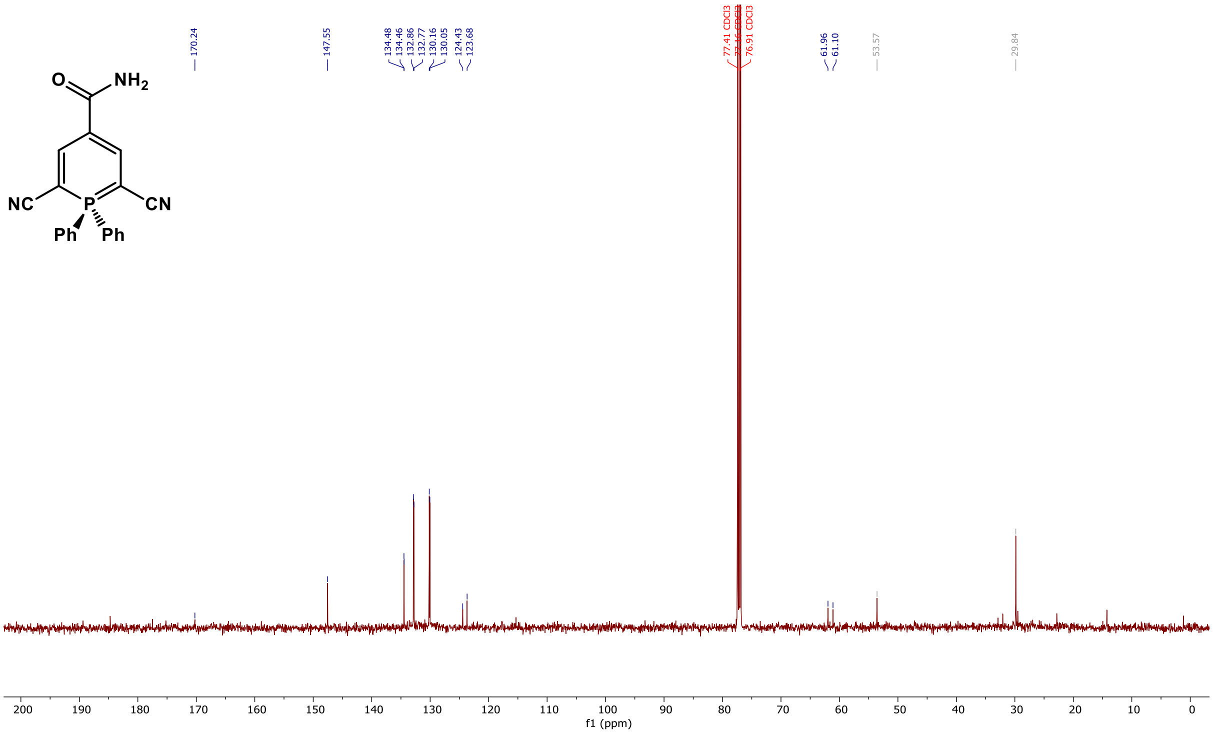

^{31}P NMR (202 MHz)

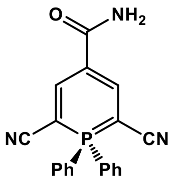

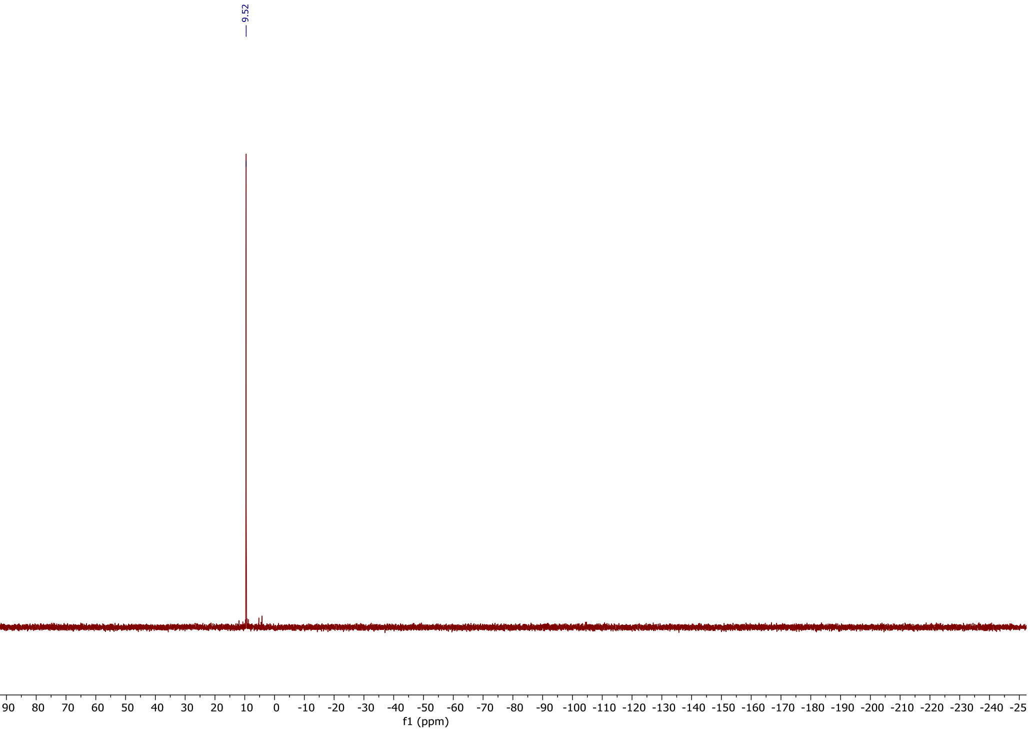

1,1-Diphenyl-1λ[5]-phosphinine-2,4,6-tricarbonitrile (2j)

^{1}H NMR (500 MHz)

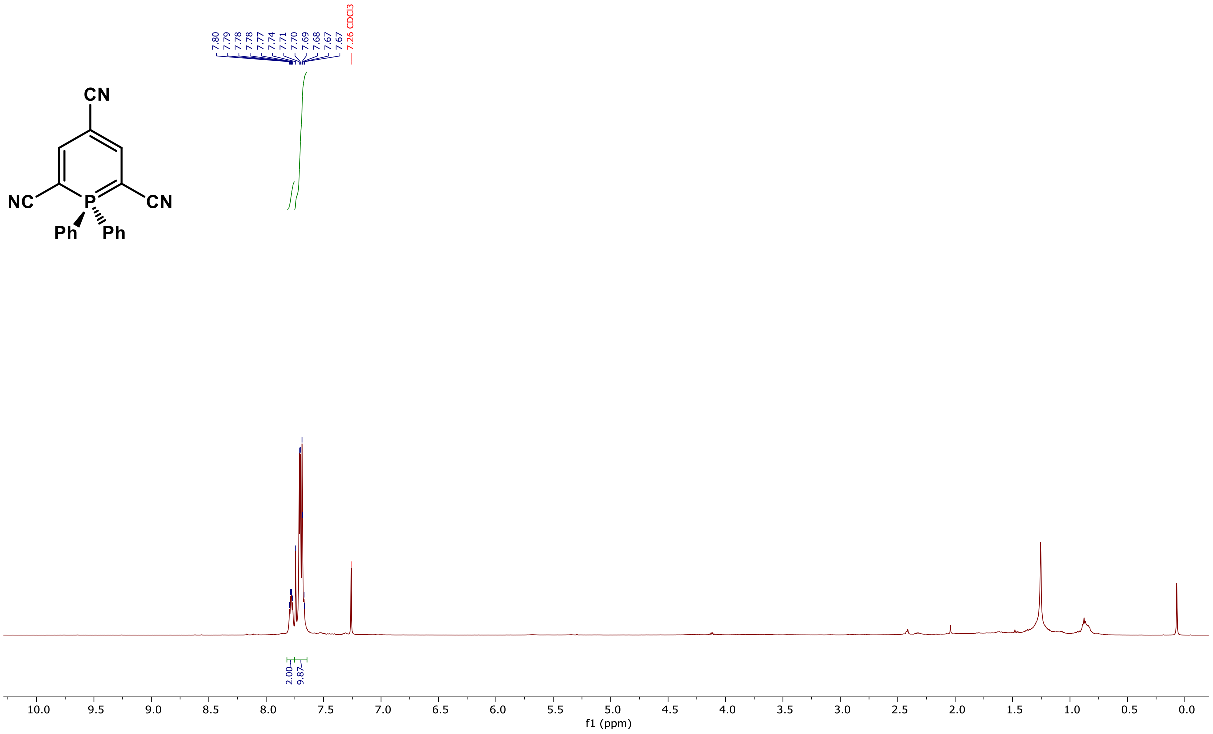

^{13}C NMR (126 MHz)

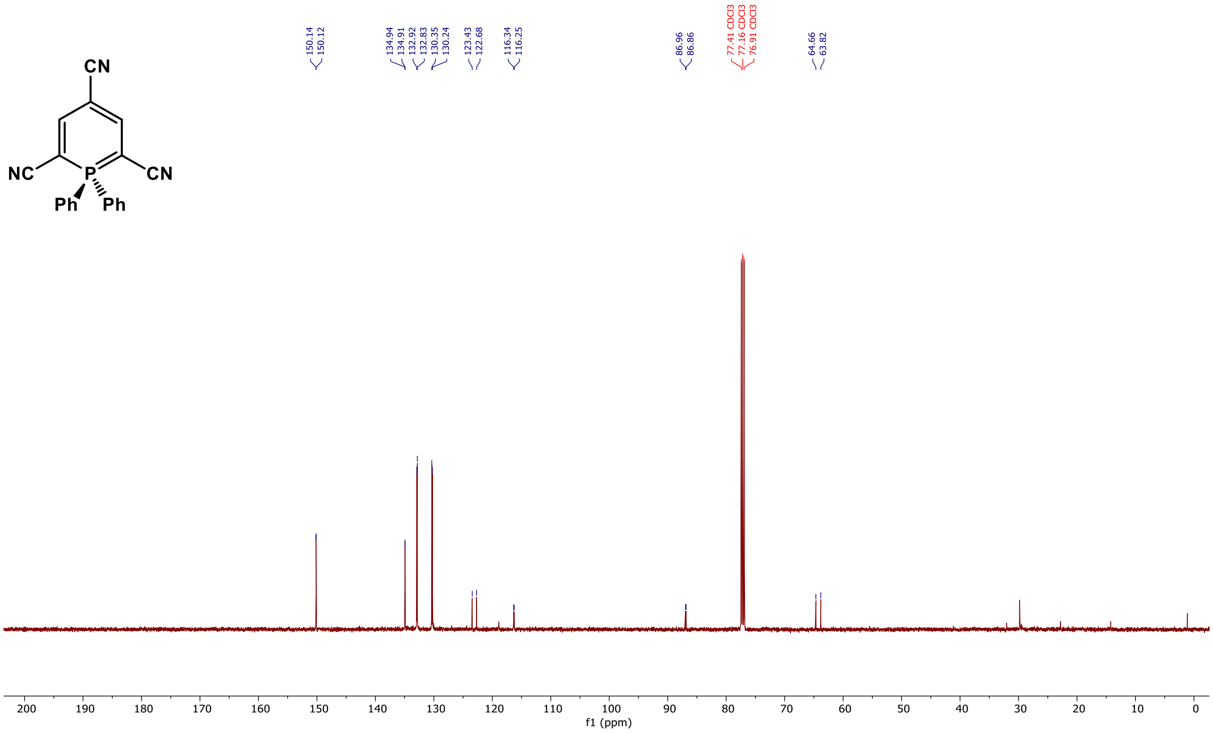

^{31}P NMR (202 MHz)

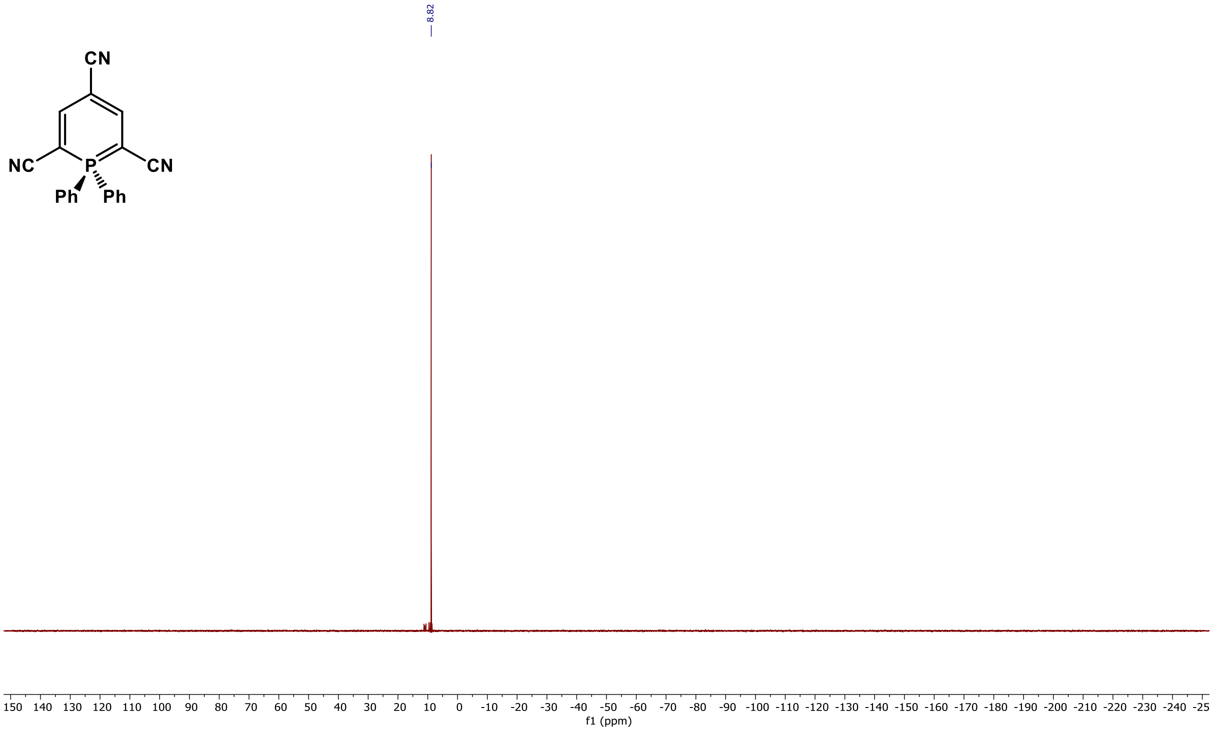

4-(Methylthio)-1,1-diphenyl-1λ^5-phosphinine-2,6-dicarbonitrile (2k)

^{1}H NMR (500 MHz)

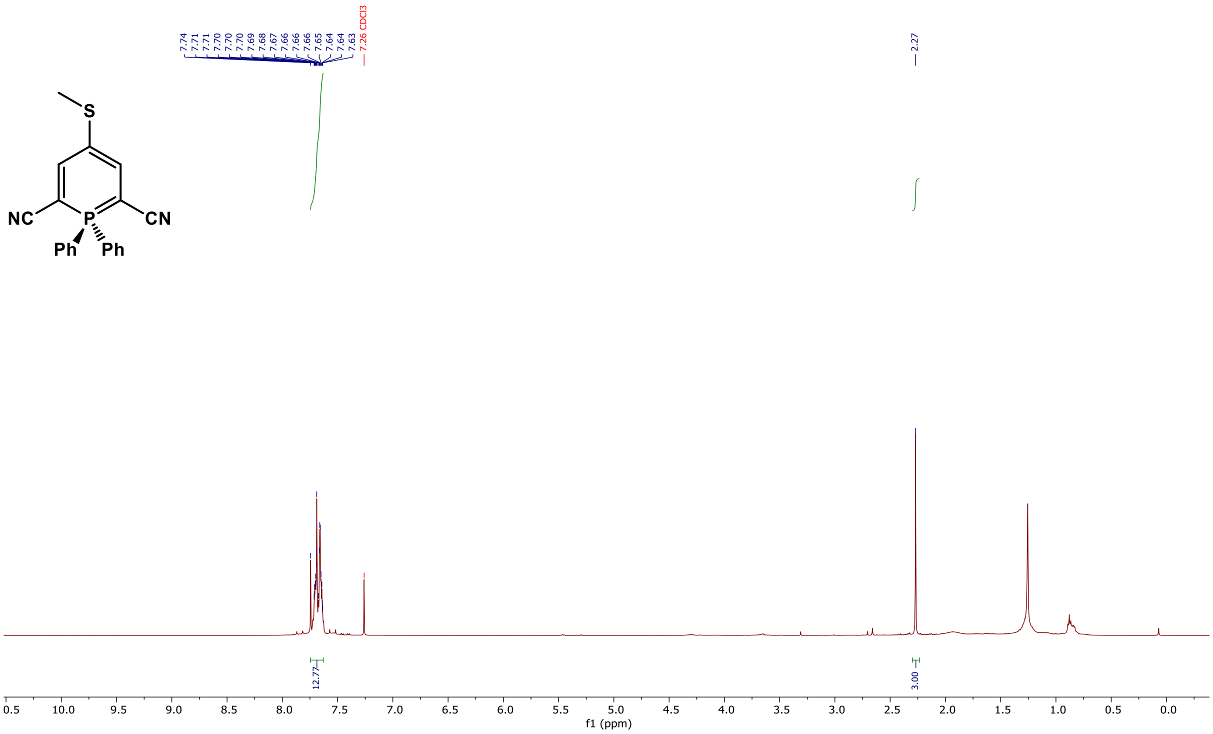

^{13}C NMR (126 MHz)

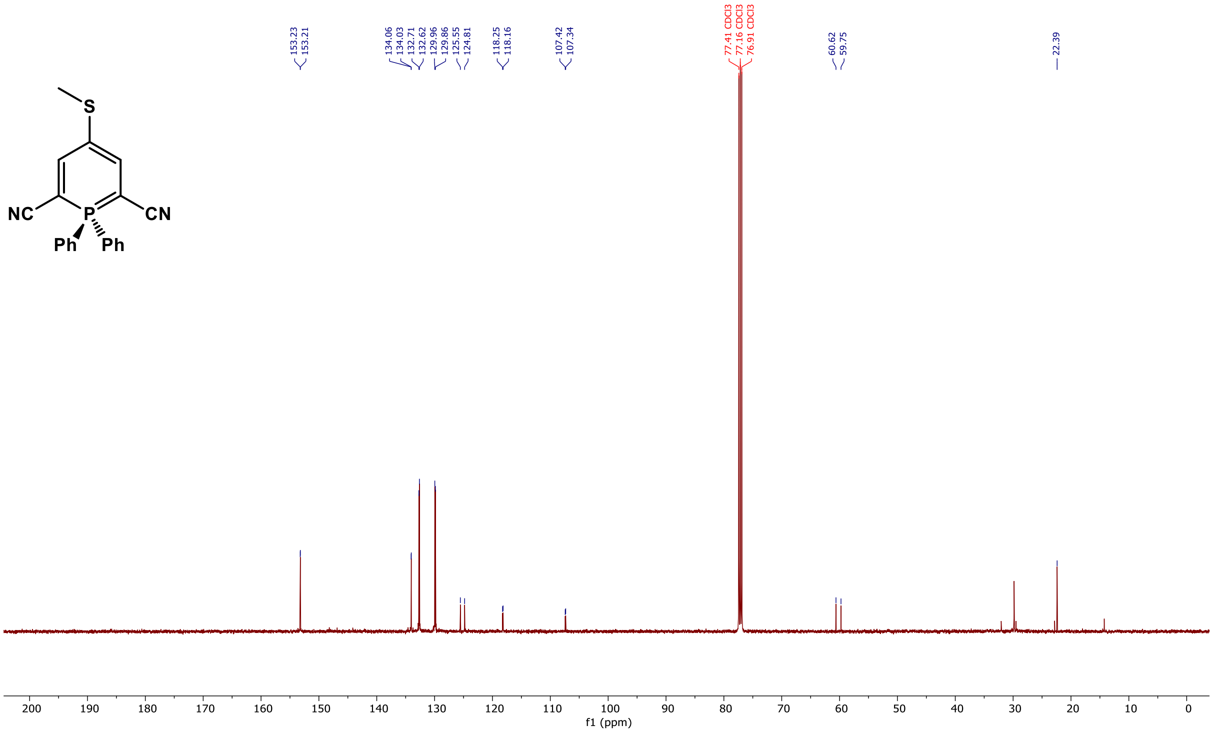

^{31}P NMR (202 MHz)

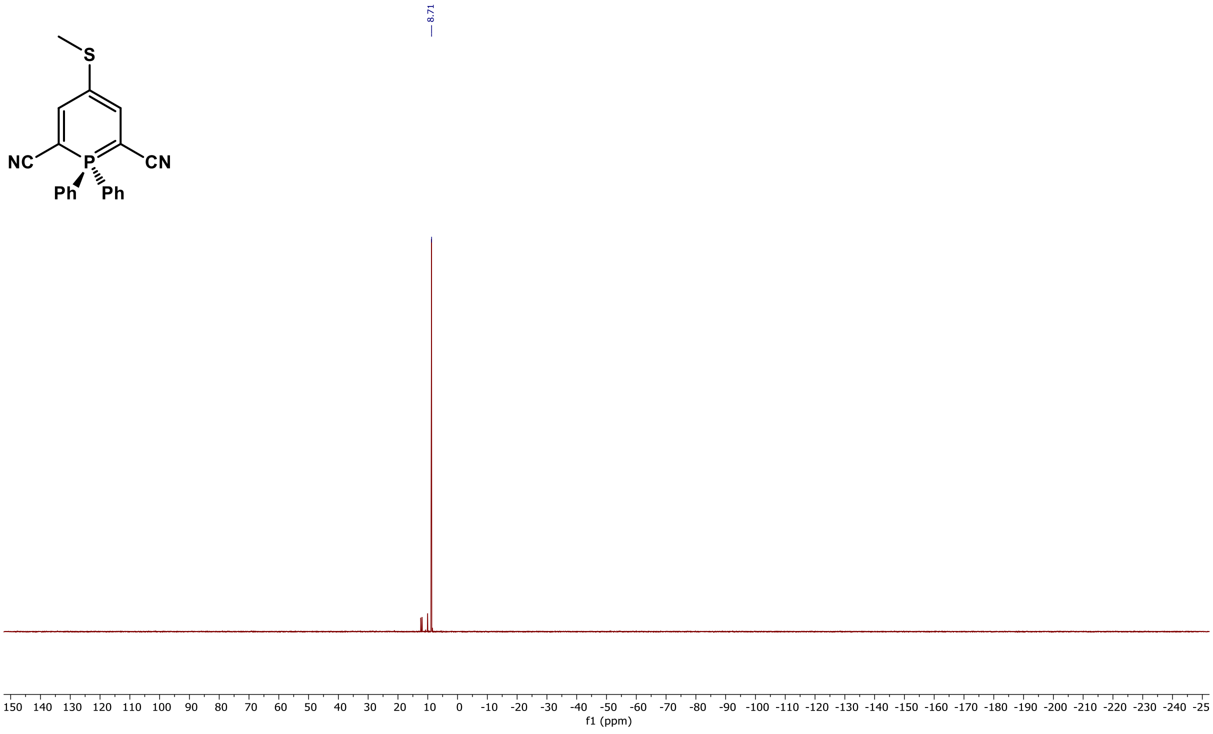

1,1-diphenyl-4-(3,4,5-trimethoxyphenyl)-1λ⁵-phosphinine-2,6-dicarbonitrile (3a)

^{1}H NMR (500 MHz)

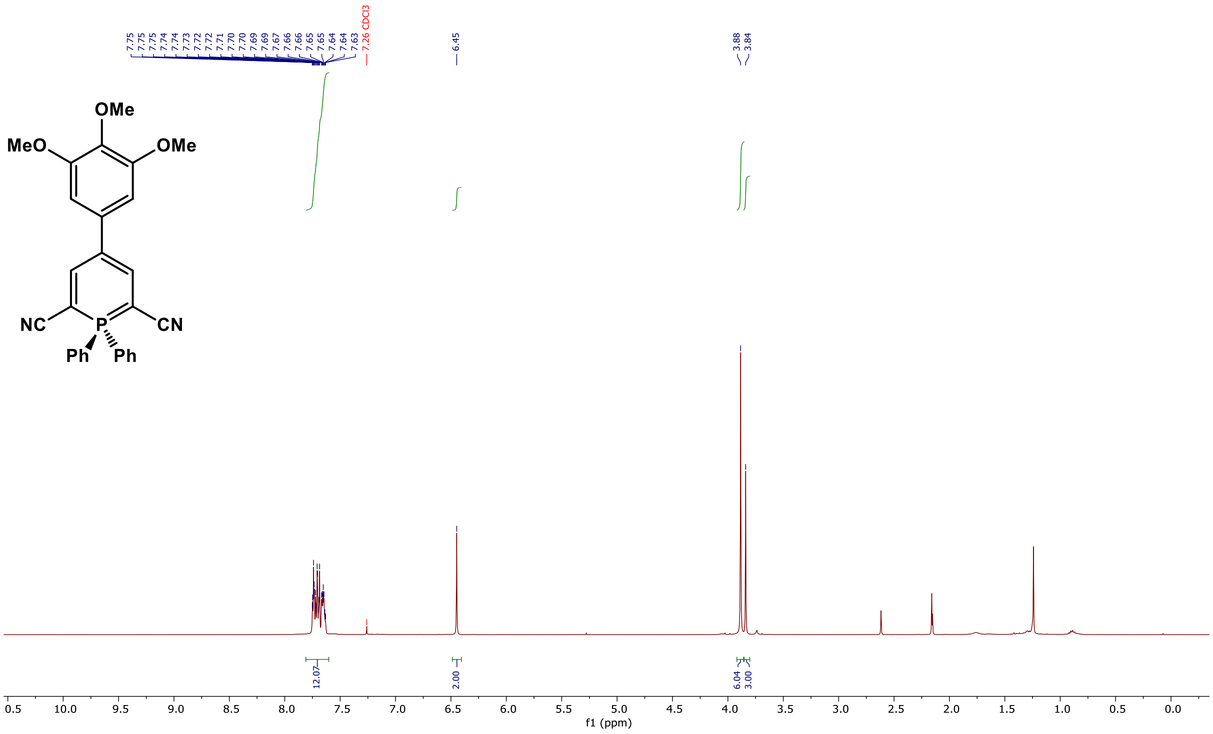

^{13}C NMR (126 MHz)

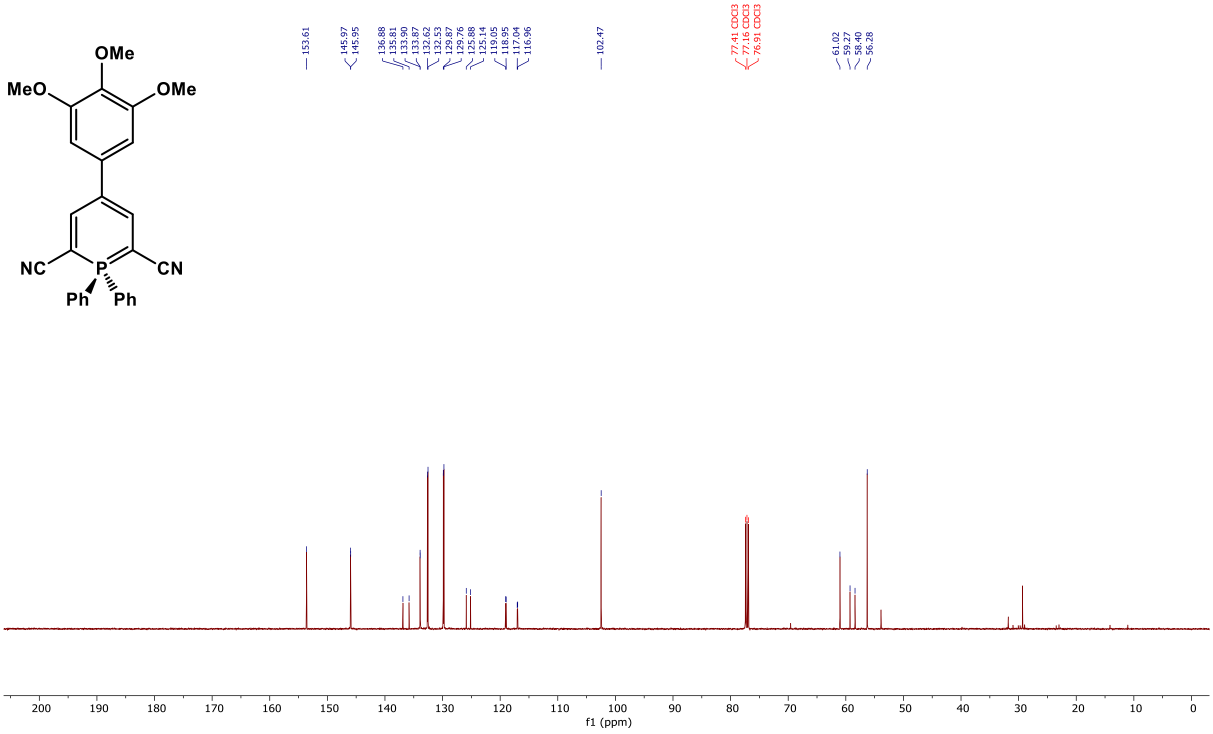

^{31}P NMR (202 MHz)

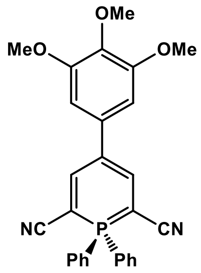

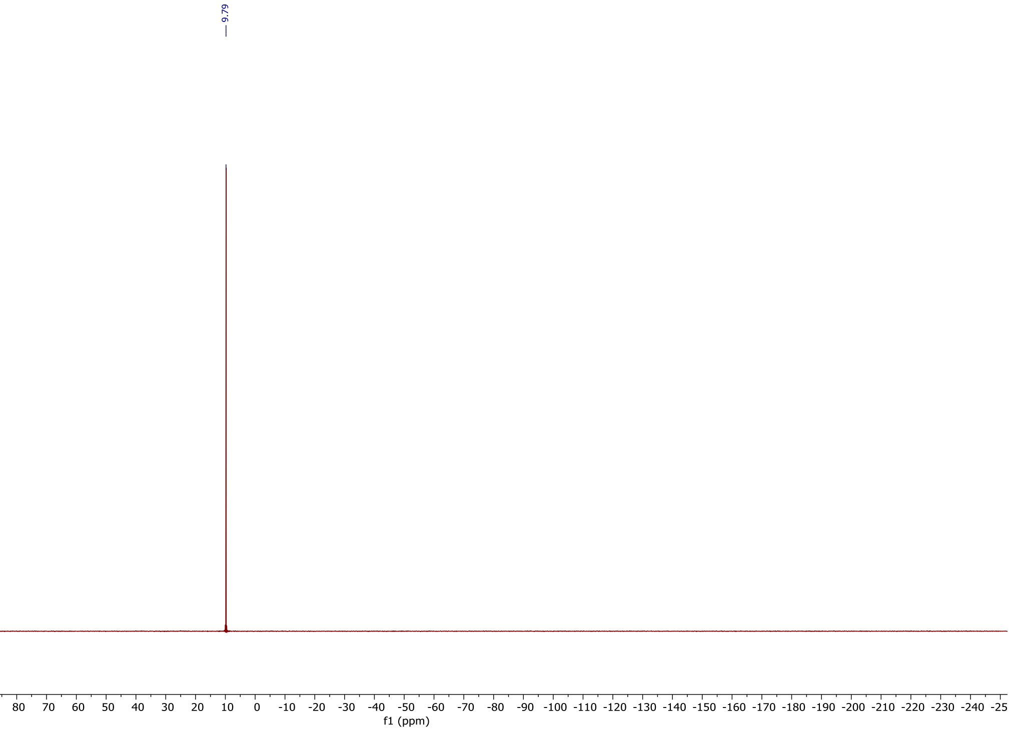

4-(4-(methylthio)phenyl)-1,1-diphenyl-1λ^5-phosphinine-2,6-dicarbonitrile (3b)

^{1}H NMR (500 MHz)

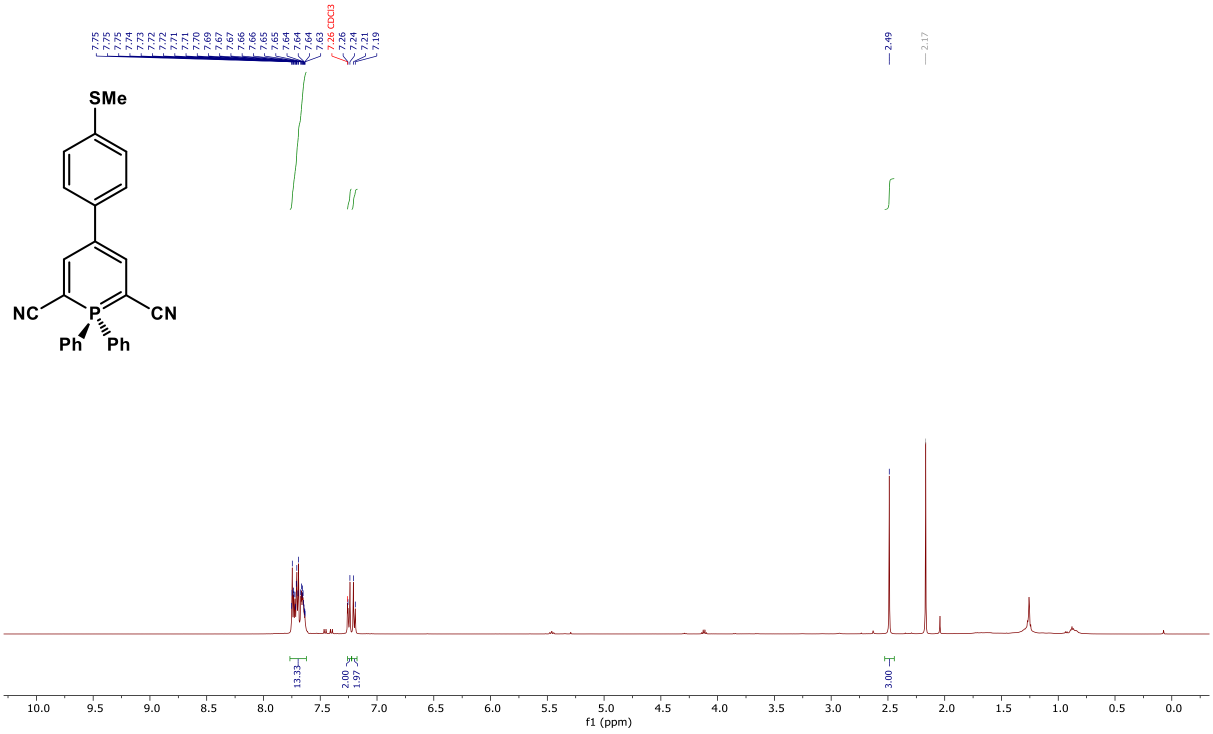

^{13}C NMR (126 MHz)

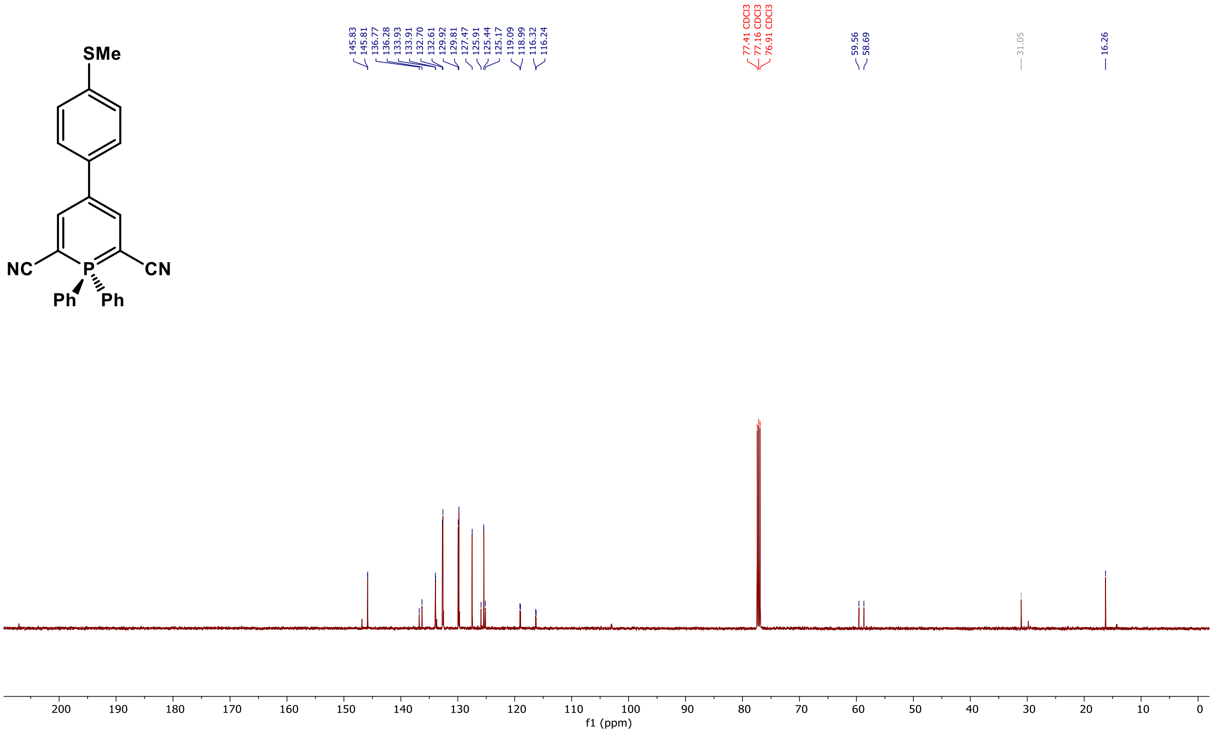

^{31}P NMR (202 MHz)

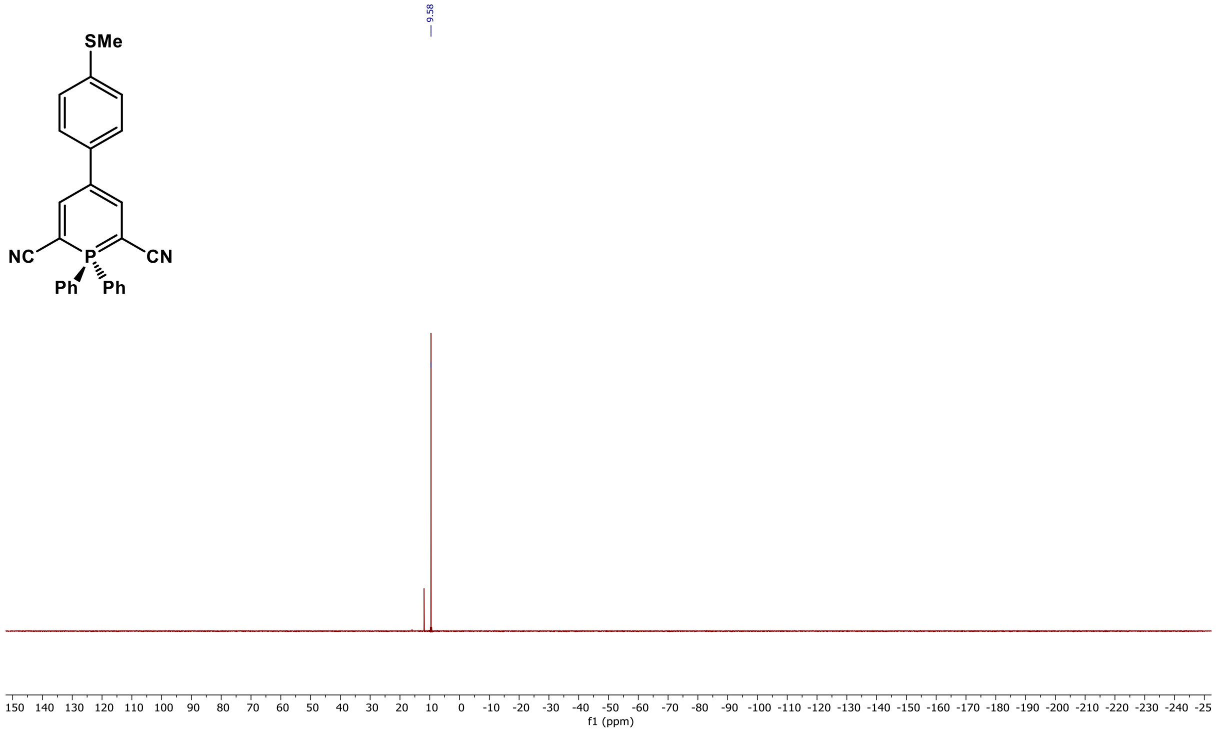

4-(3-nitrophenyl)-1,1-diphenyl-1λ^5-phosphinine-2,6-dicarbonitrile (3c)

^{1}H NMR (500 MHz)

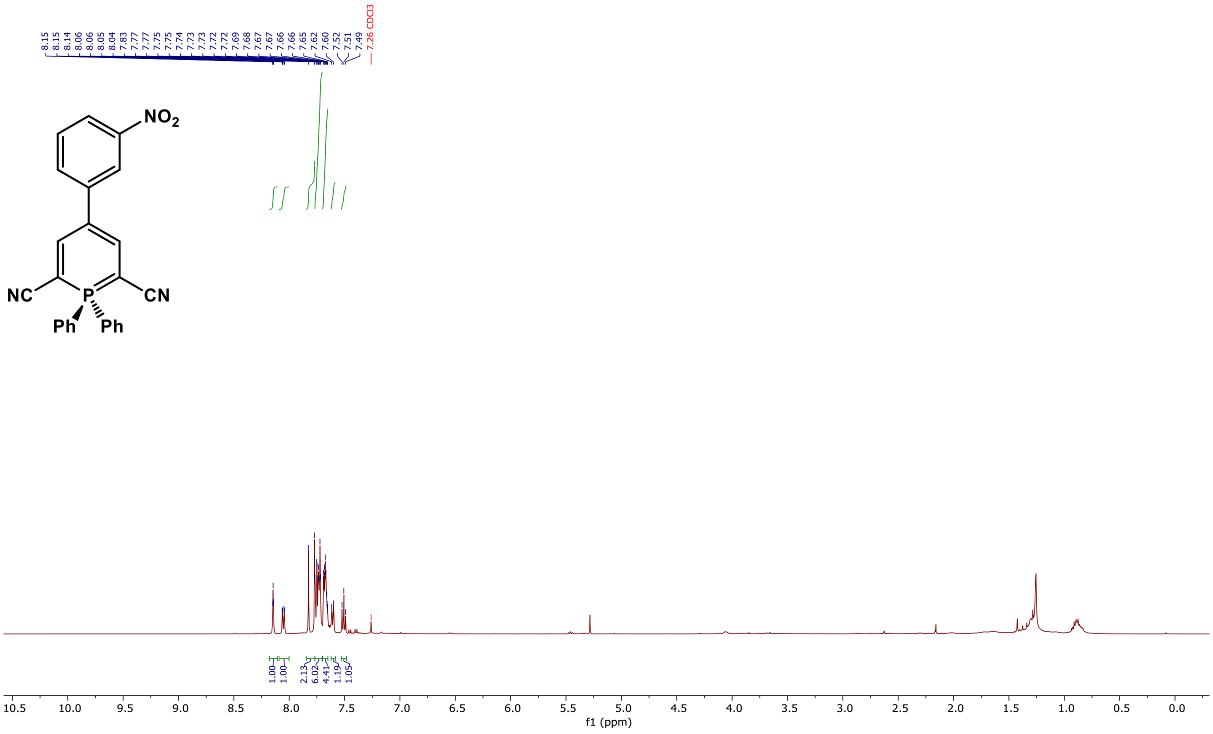

^{13}C NMR (126 MHz)

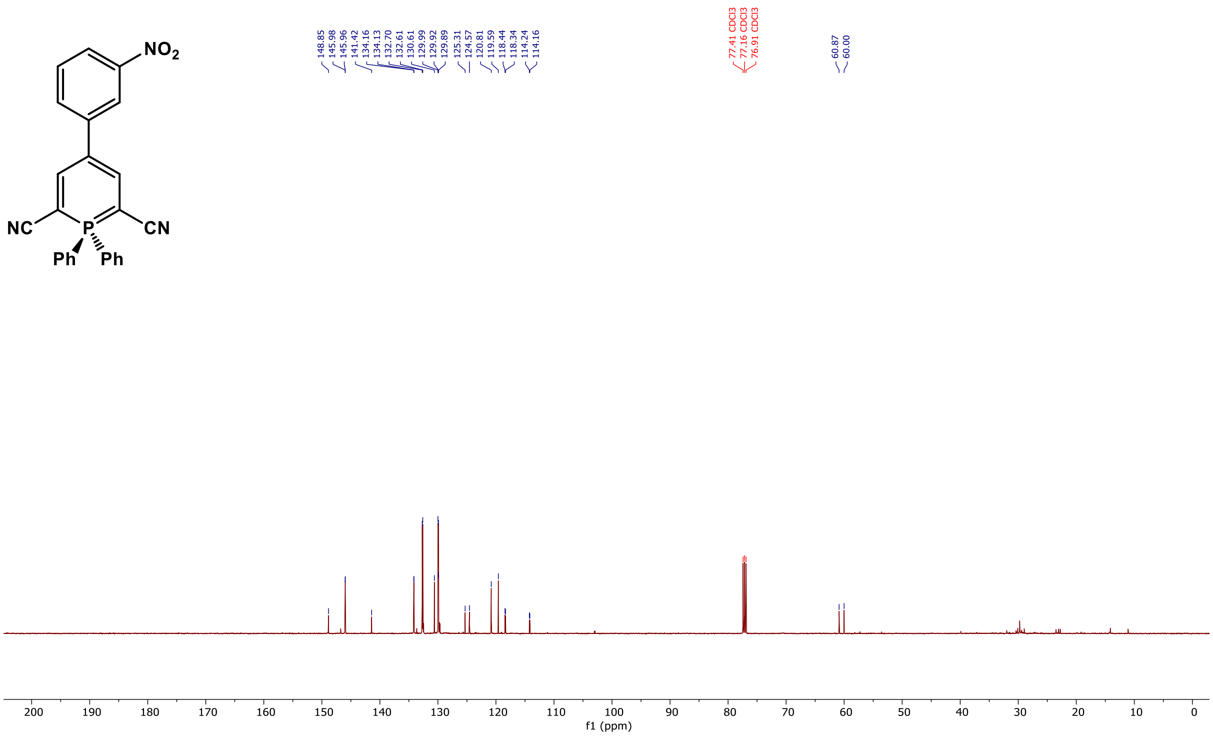

^{31}P NMR (202 MHz)

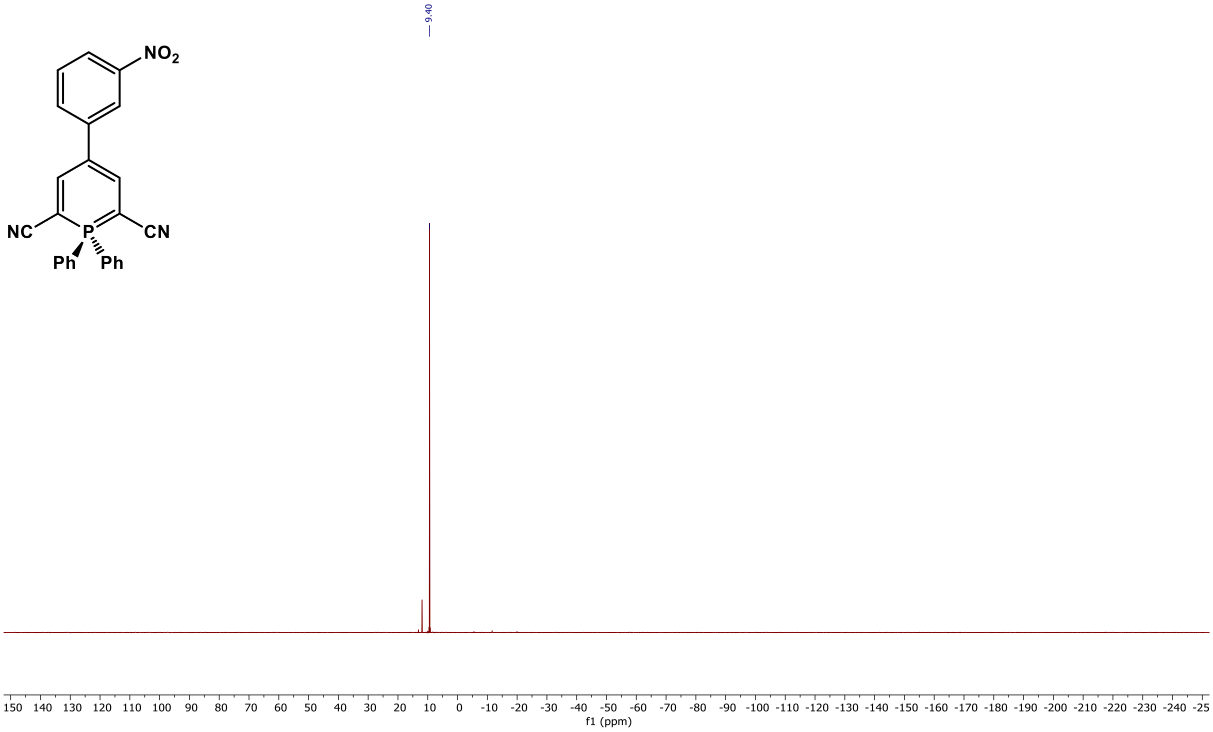

1,1-diphenyl-4-(3-(trifluoromethoxy)phenyl)-1λ^5-phosphinine-2,6-dicarbonitrile (3d)

^{1}H NMR (500 MHz)

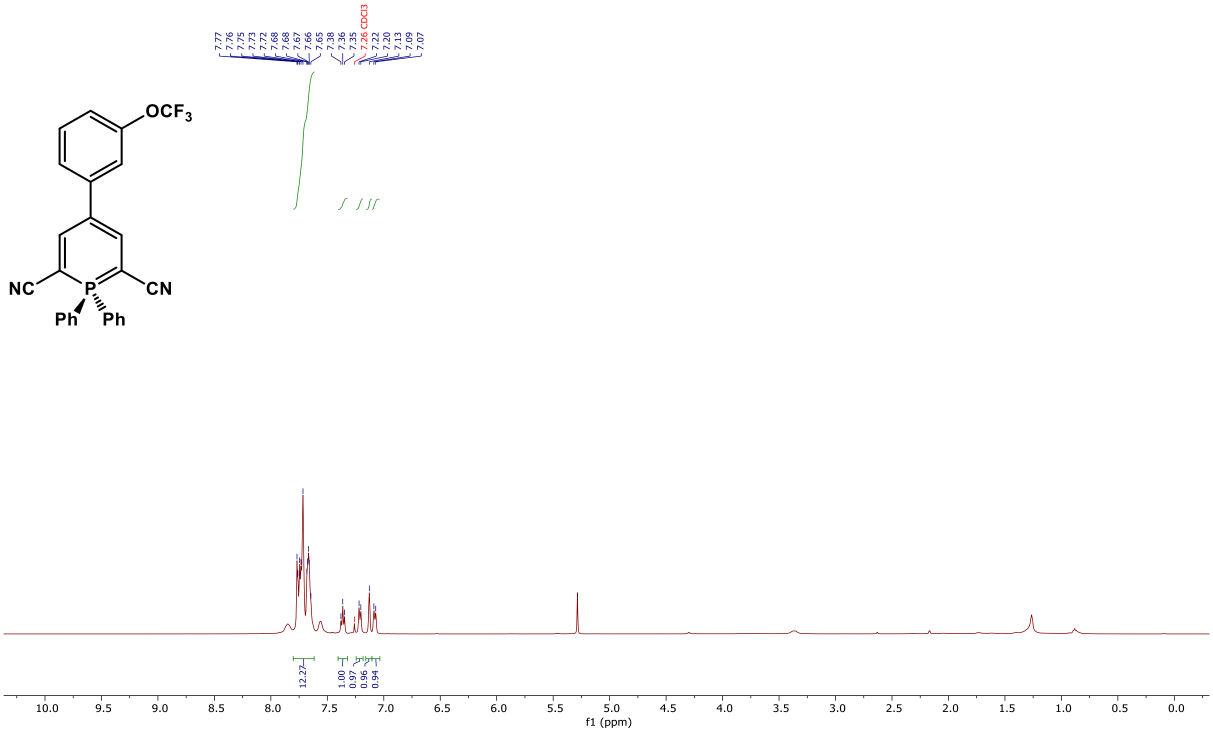

^{13}C NMR (126 MHz)

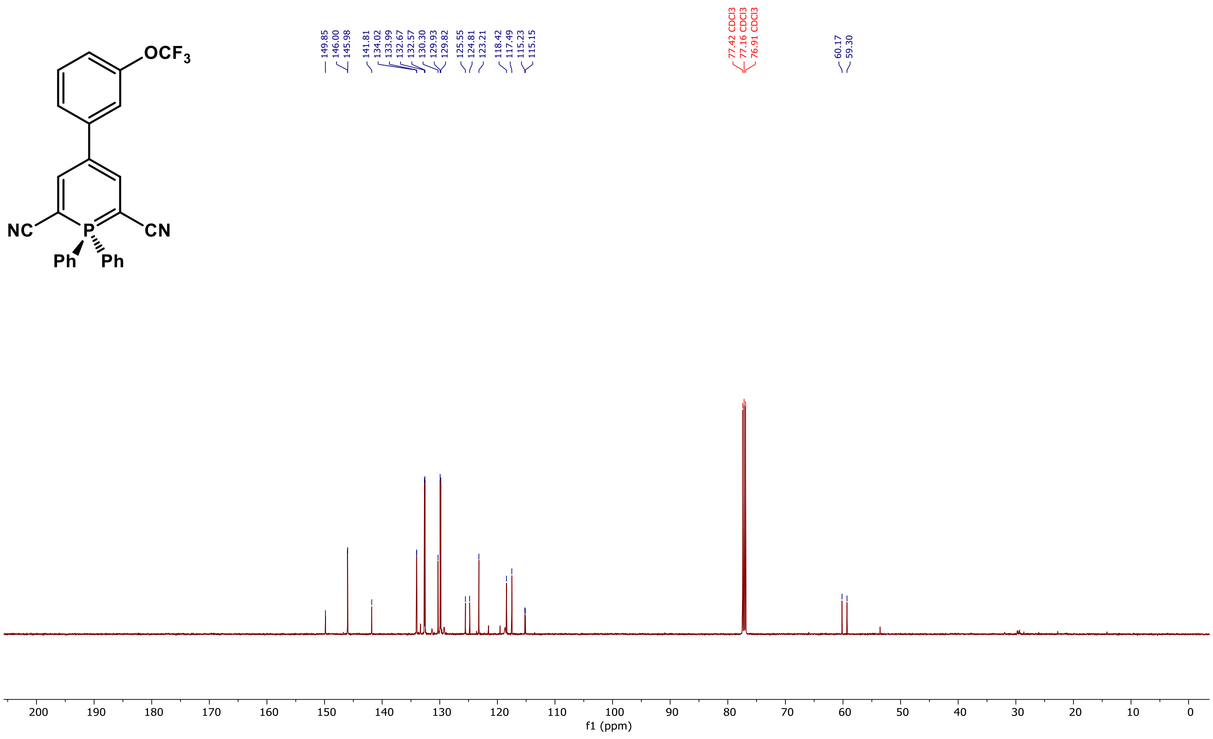

^{31}P NMR (202 MHz)

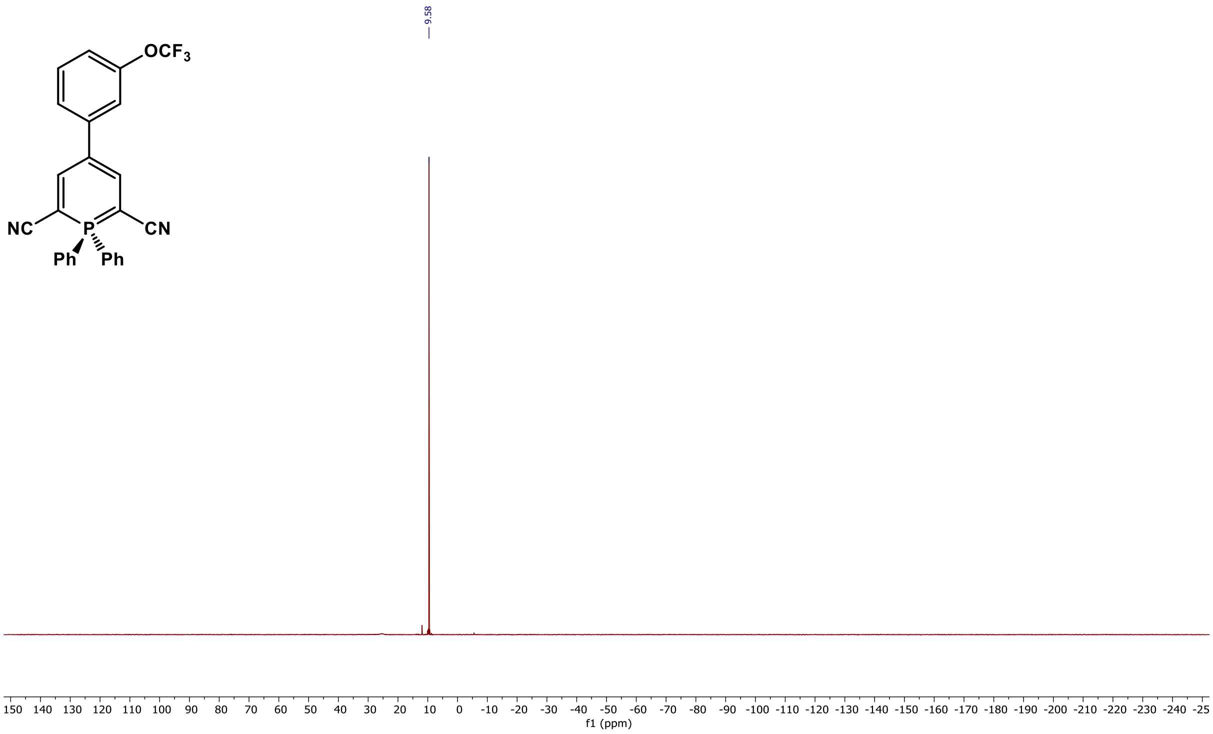

^{19}F NMR (471 MHz)

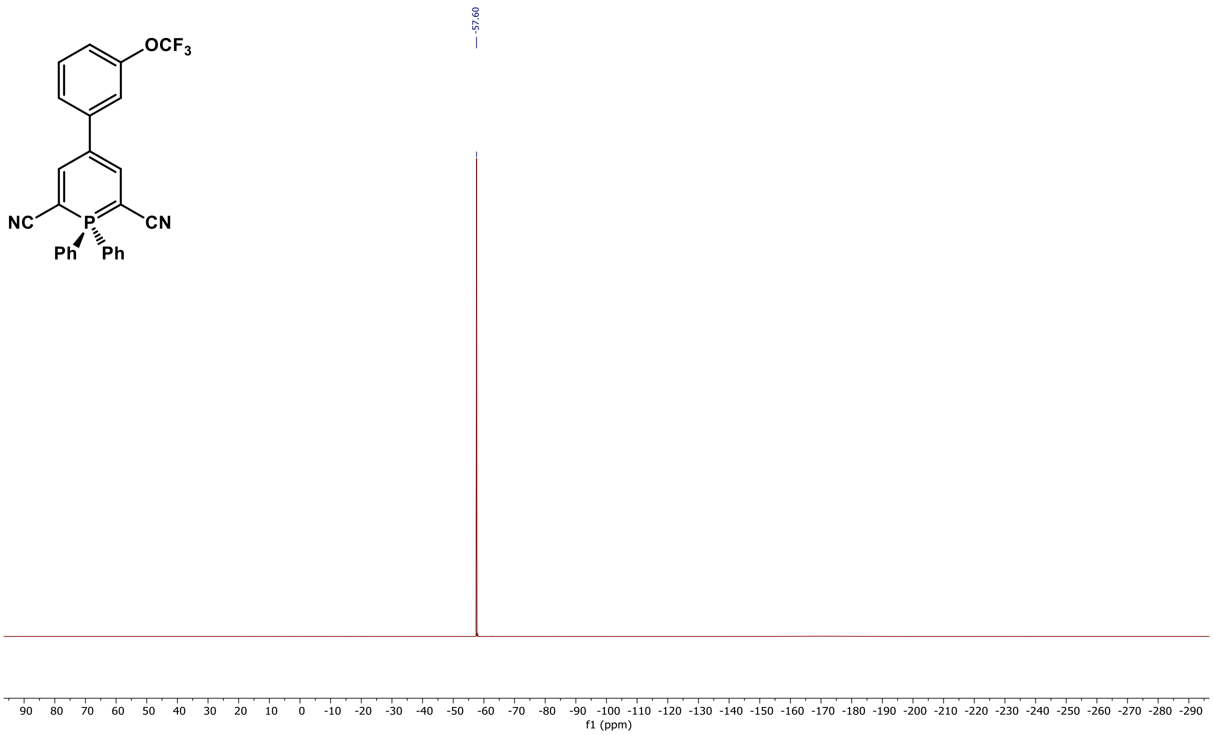

4-(2-chloro-4-fluorophenyl)-1,1-diphenyl-1λ^5-phosphinine-2,6-dicarbonitrile (3e)

^{1}H NMR (500 MHz)

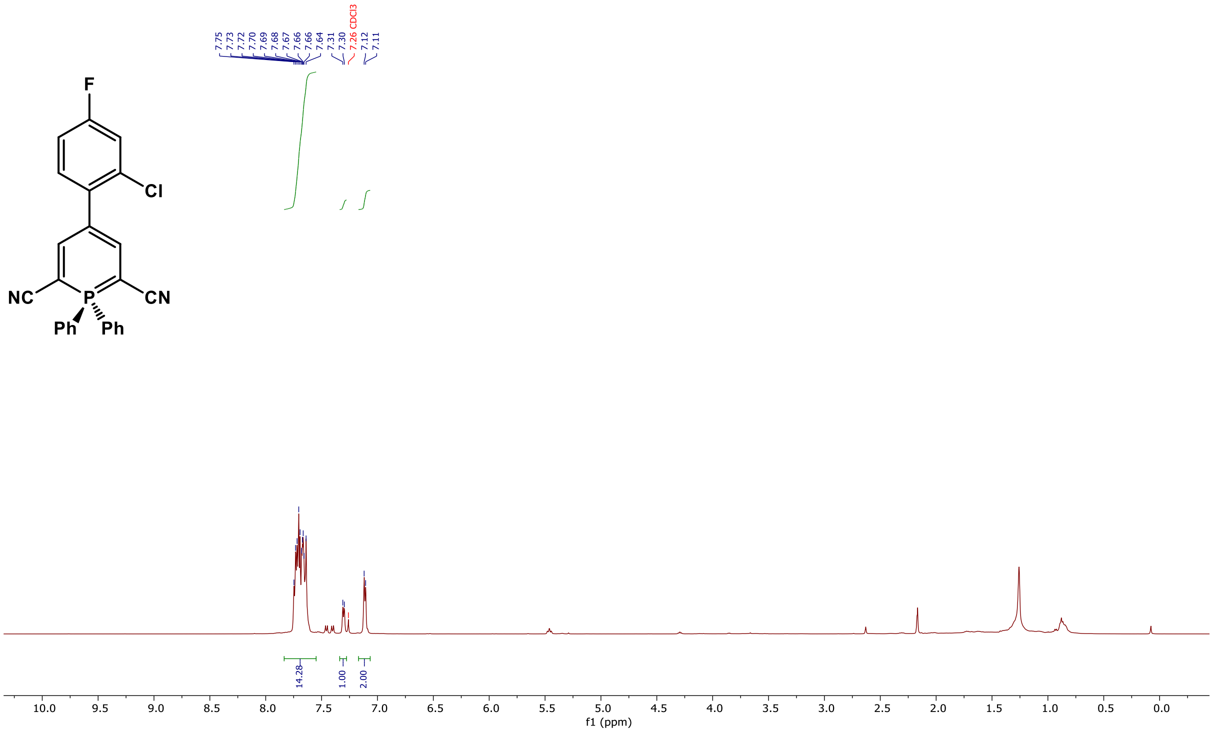

^{13}C NMR (126 MHz)

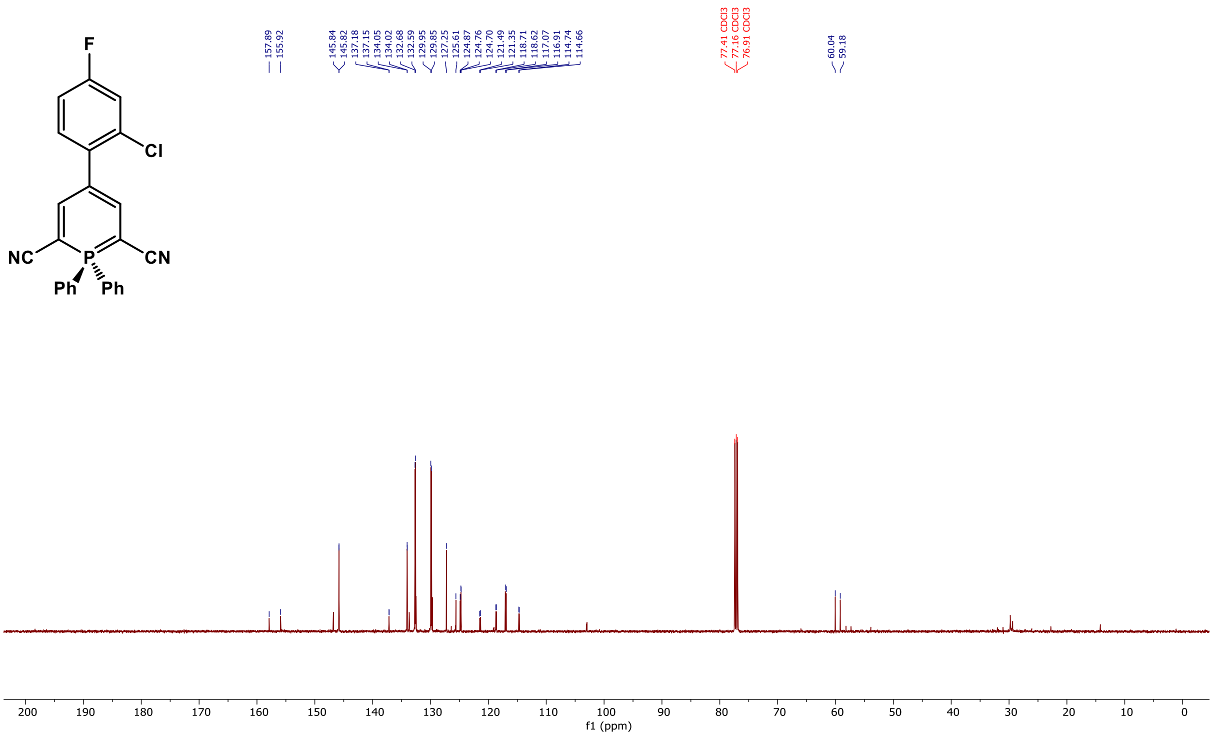

^{31}P NMR (202 MHz)

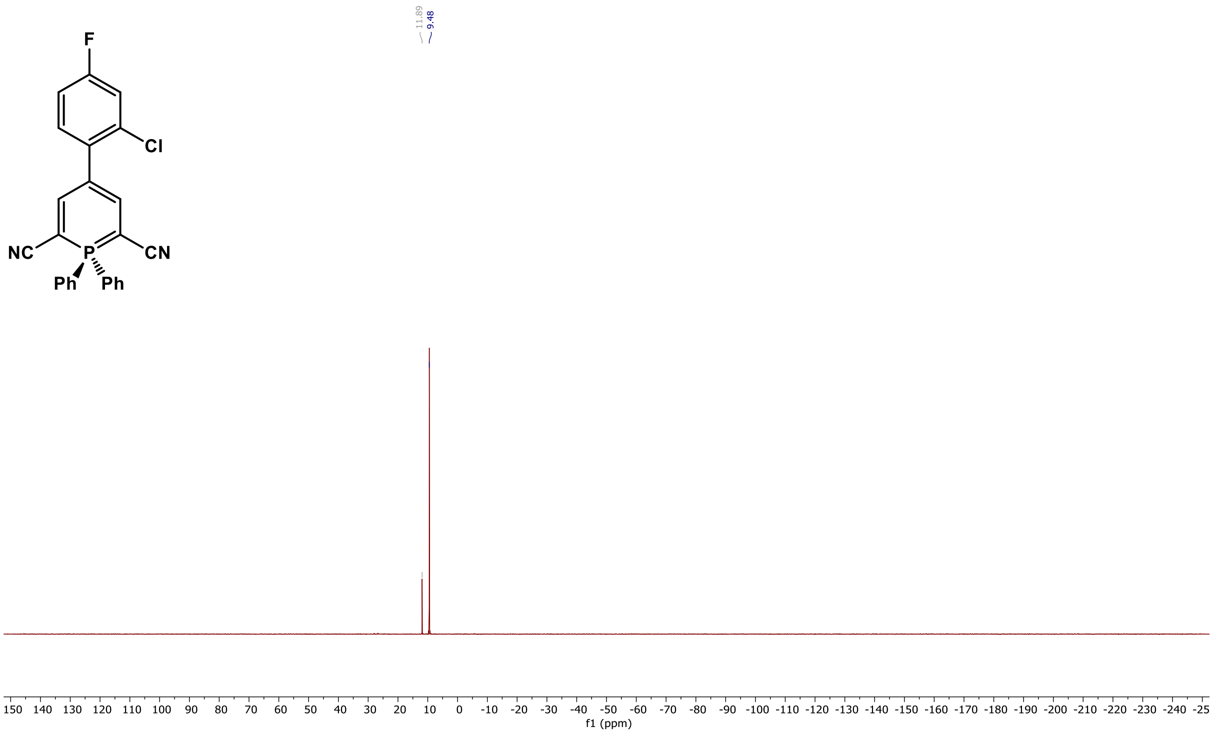

^{19}F NMR (471 MHz)

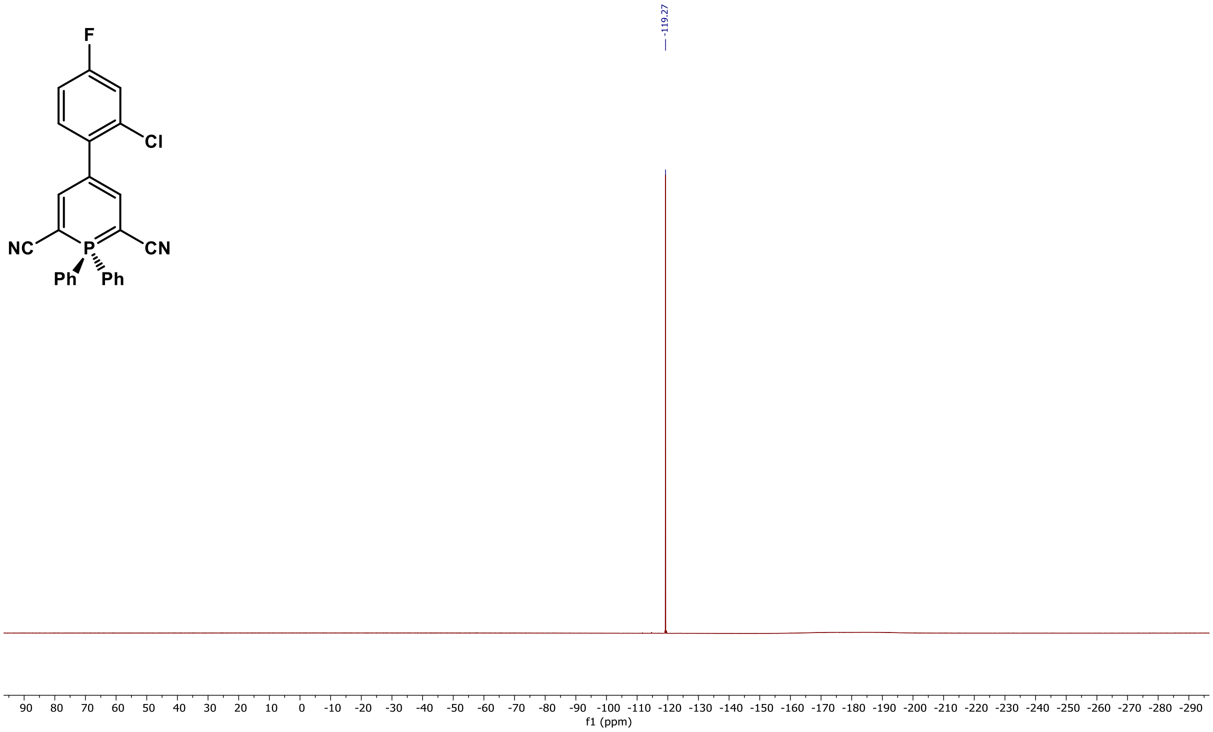

4-(4-acetylphenyl)-1,1-diphenyl-1λ^5-phosphinine-2,6-dicarbonitrile (3f)

^{1}H NMR (500 MHz)

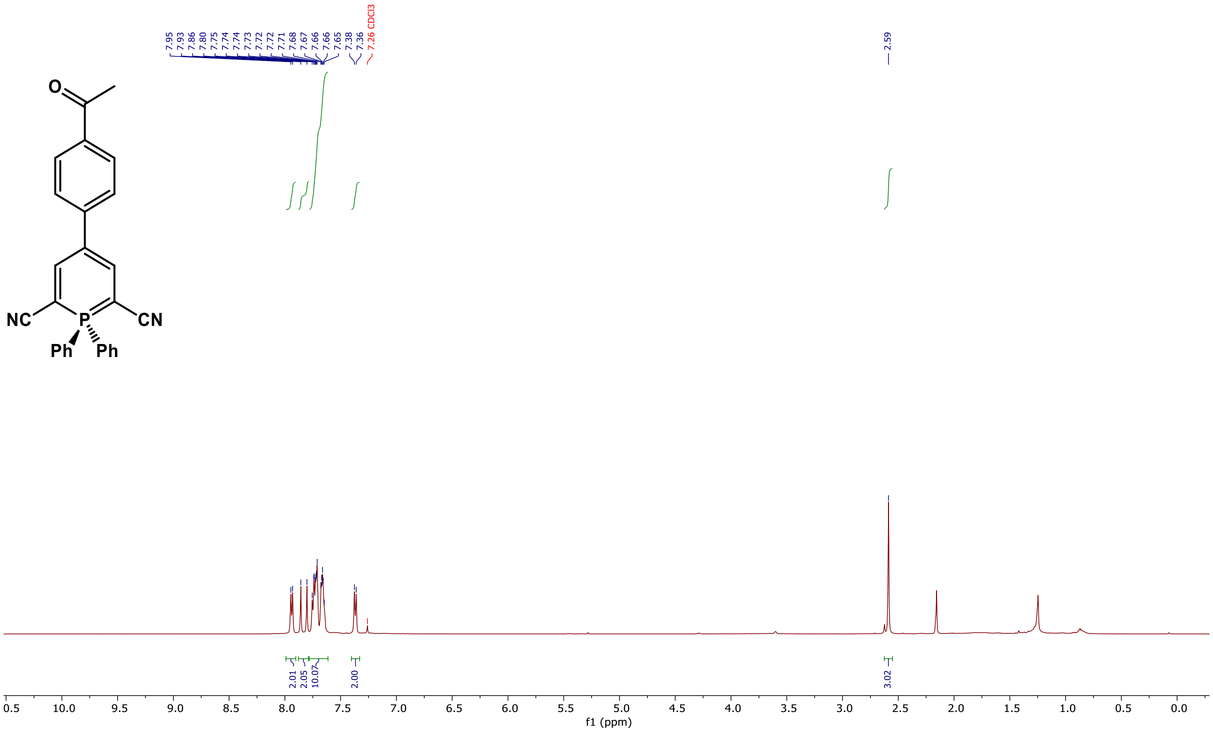

^{13}C NMR (126 MHz)

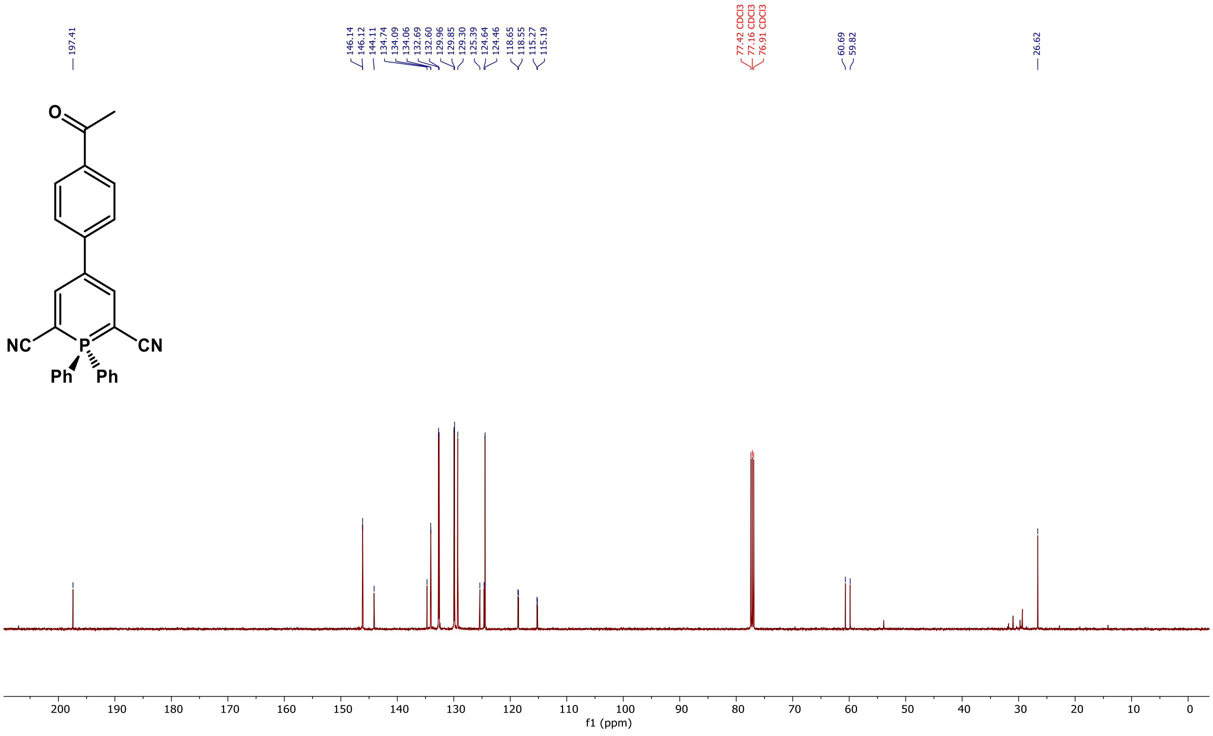

^{31}P NMR (202 MHz)

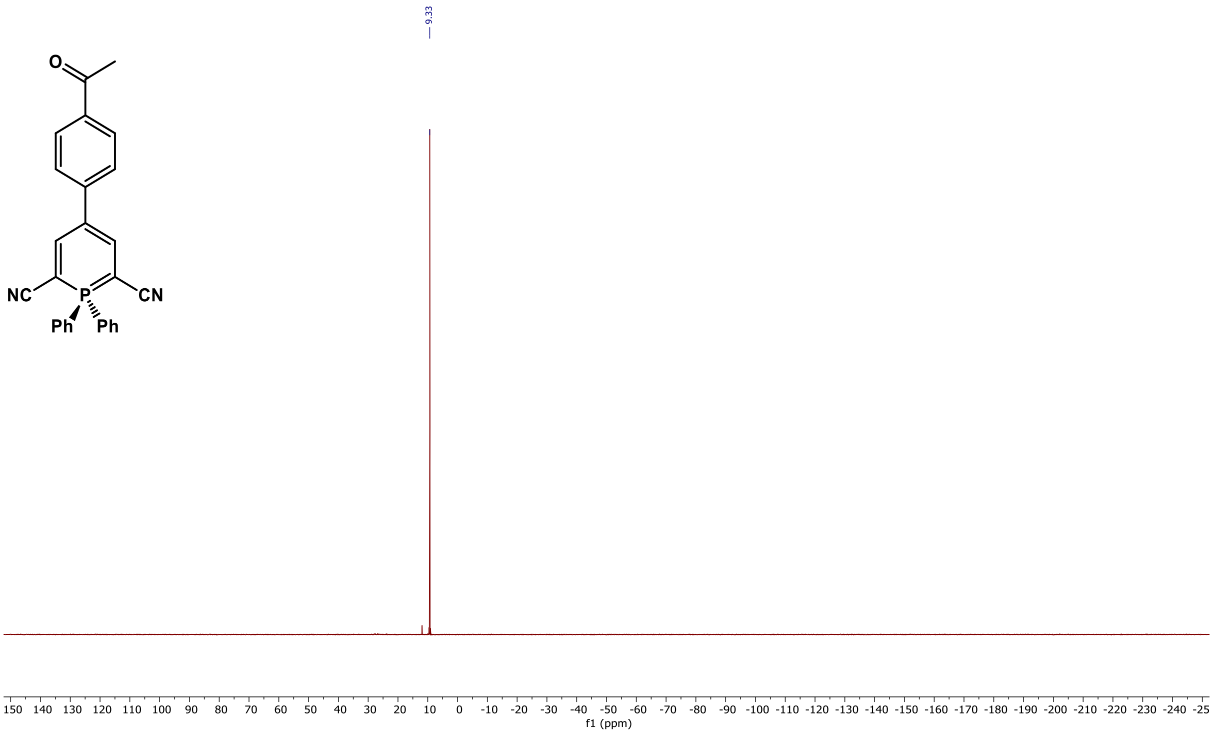

ethyl 4-(2,6-dicyano-1,1-diphenyl-1λ⁵-phosphinin-4-yl)benzoate (3g)

^{1}H NMR (500 MHz)

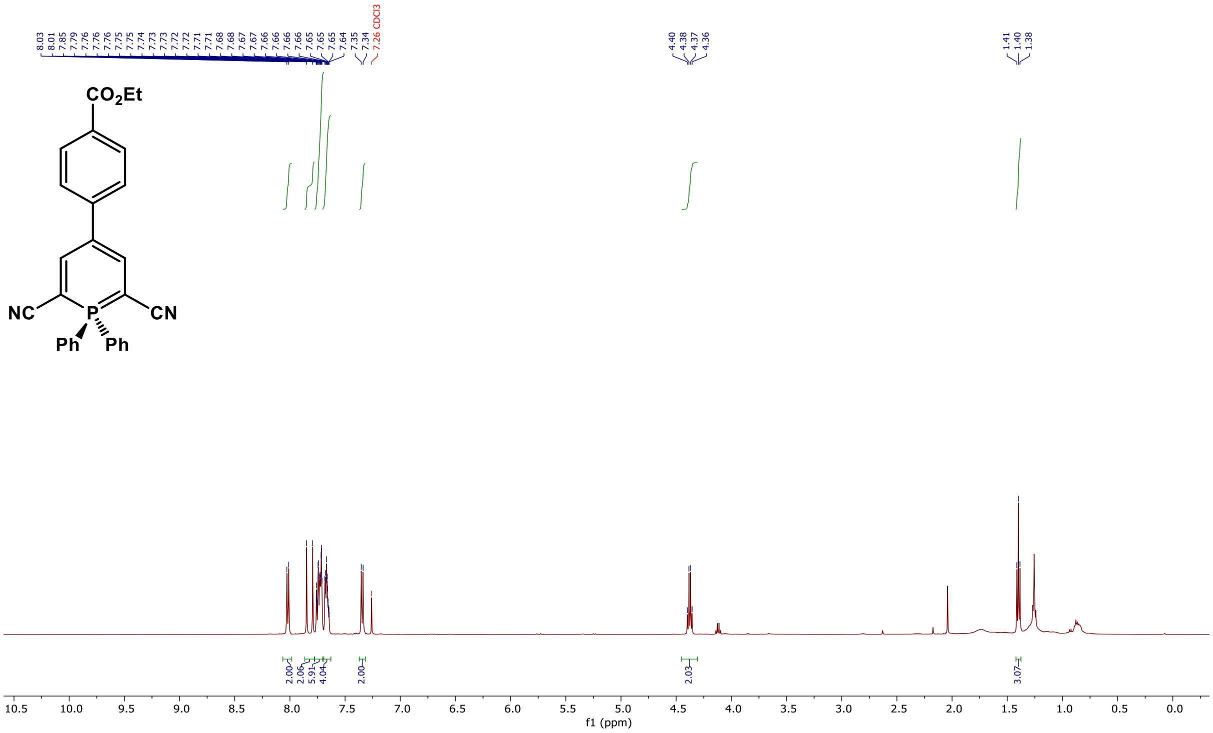

^{13}C NMR (126 MHz)

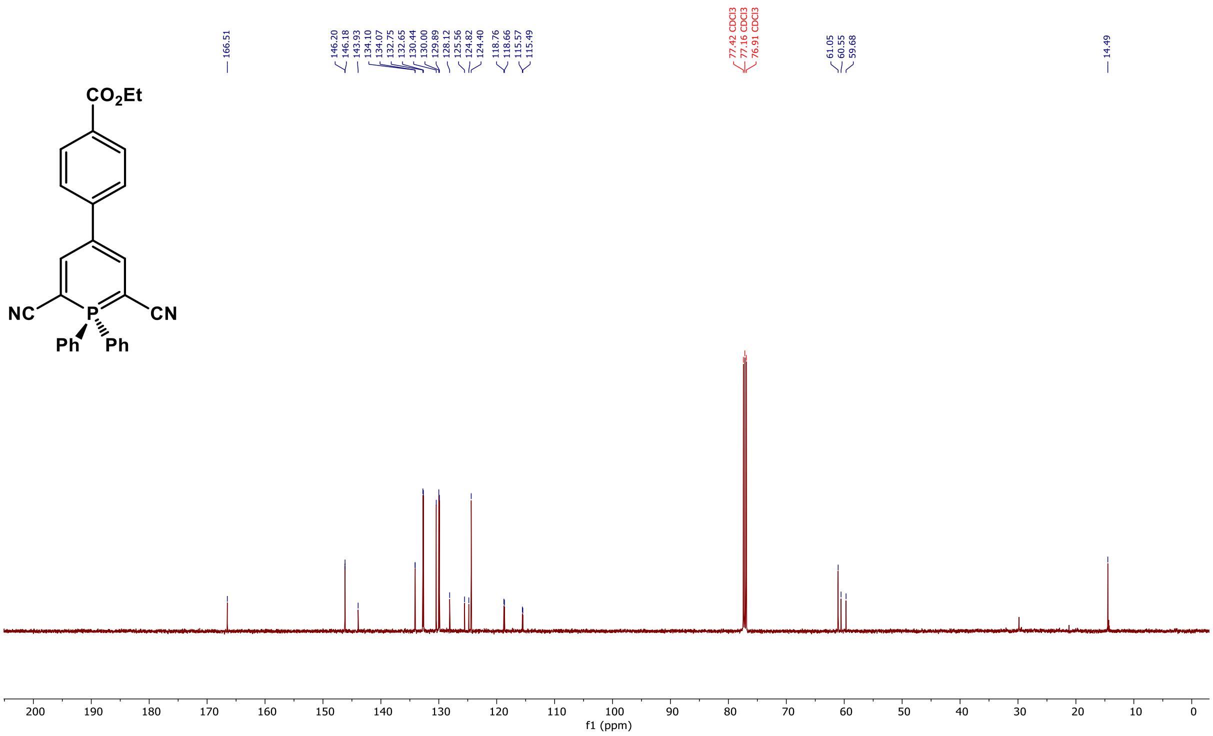

^{31}P NMR (202 MHz)

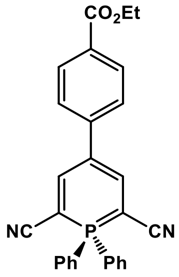

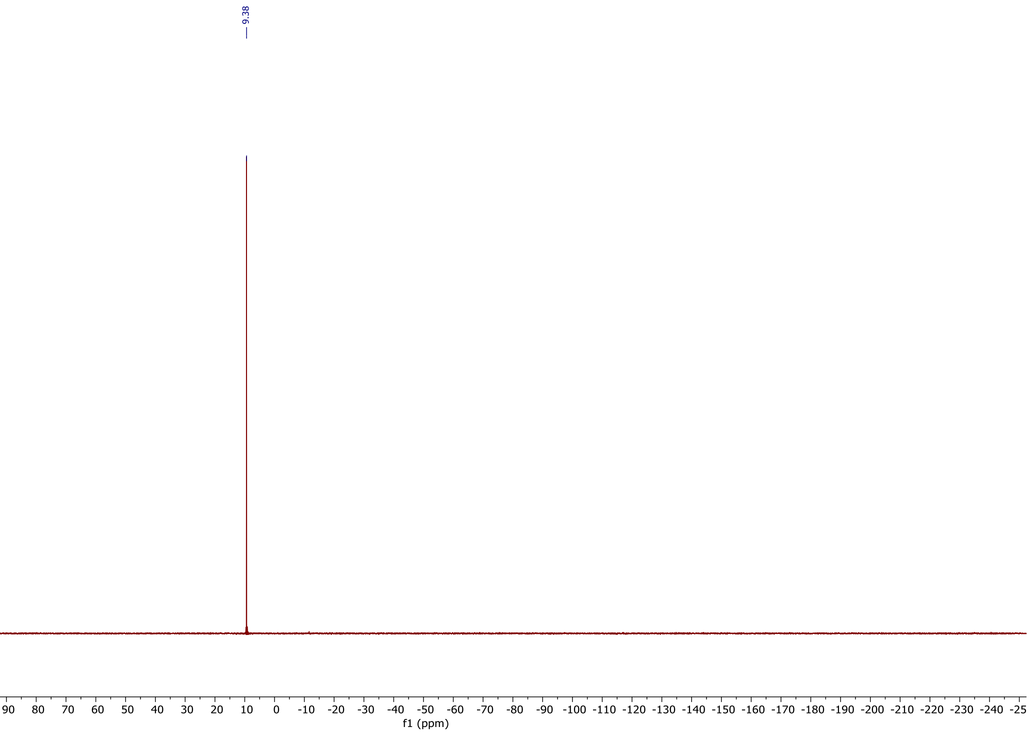

ethyl 2-(2,6-dicyano-1,1-diphenyl-1λ^5-phosphinin-4-yl)benzoate (3h)

^{1}H NMR (500 MHz)

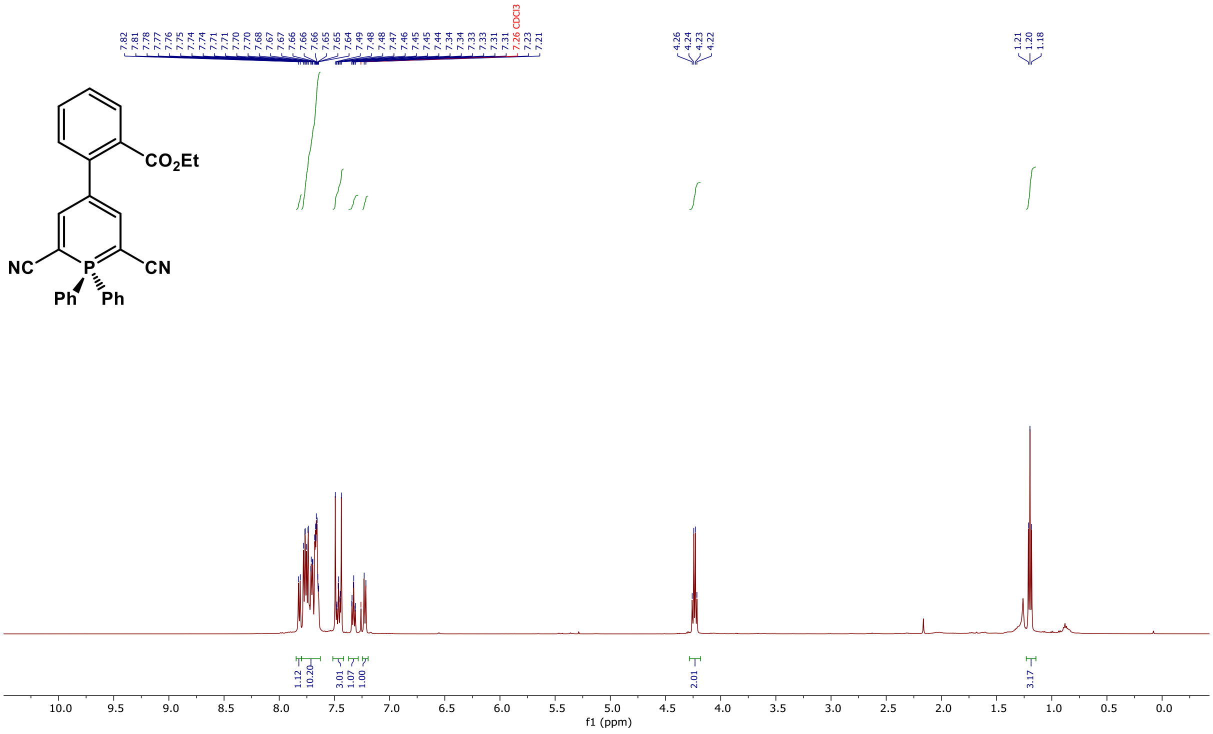

^{13}C NMR (126 MHz)

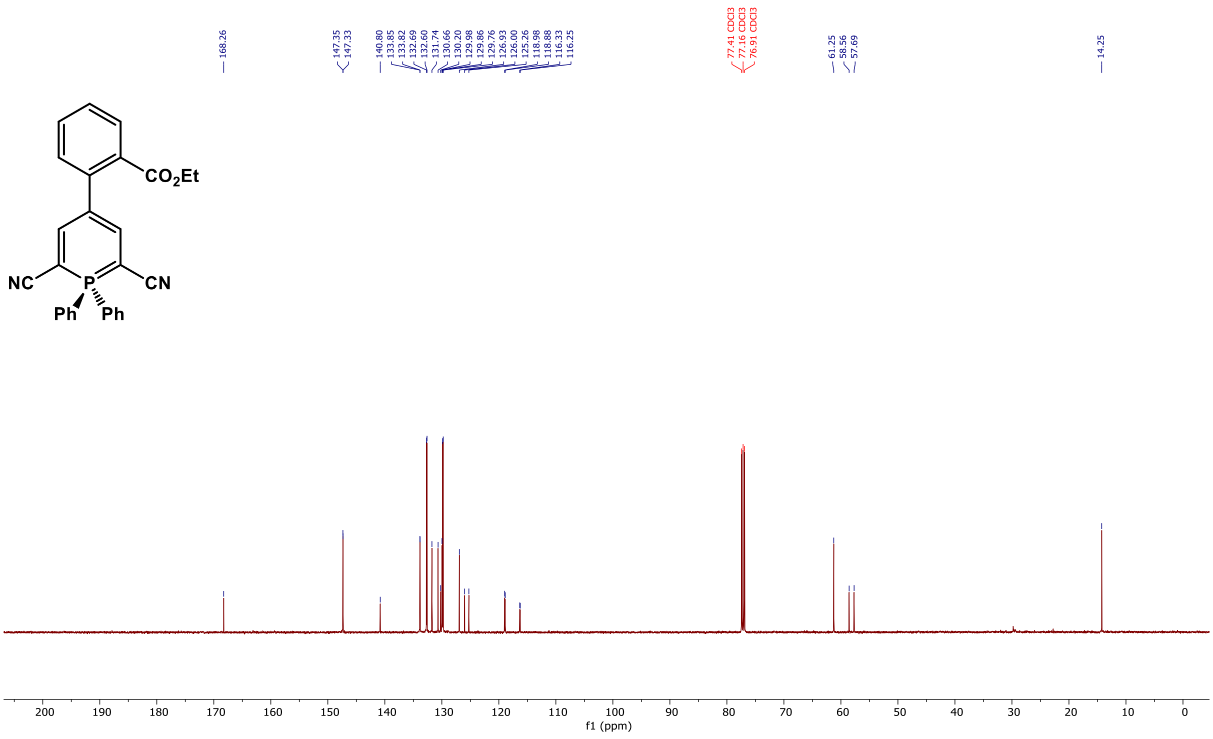

^{31}P NMR (202 MHz)

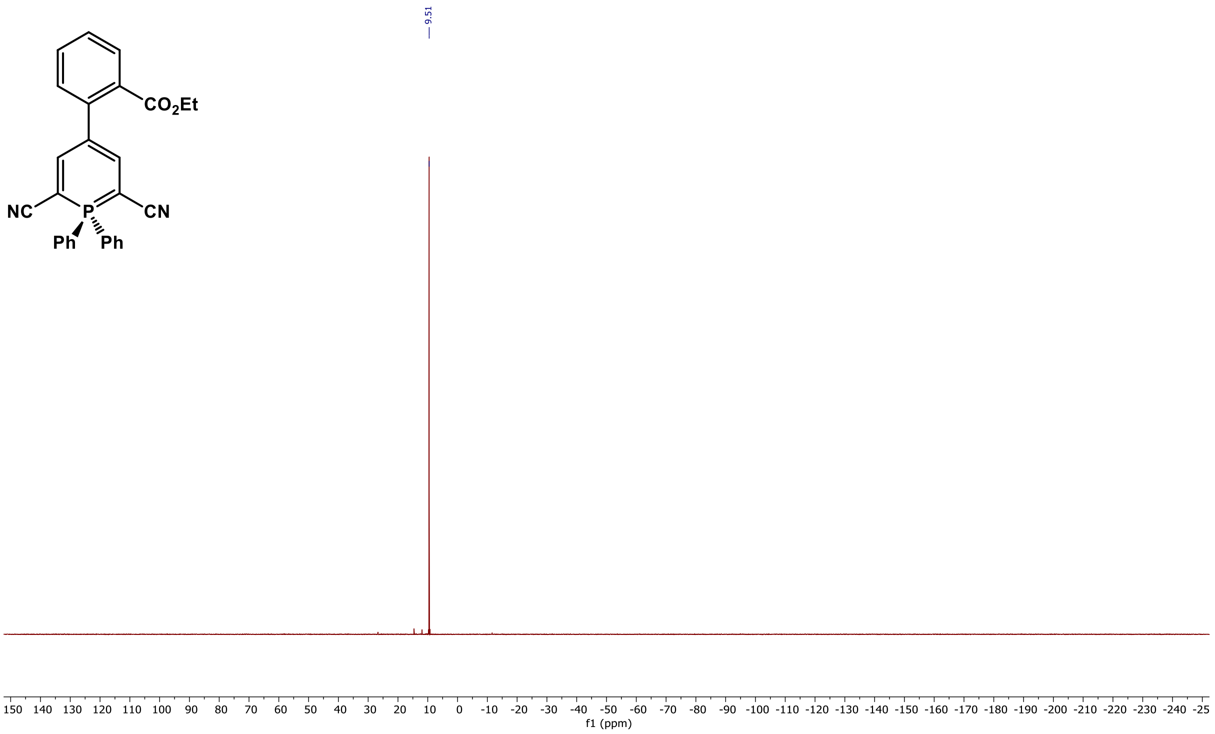

ethyl 3-(2,6-dicyano-1,1-diphenyl-1λ^5-phosphinin-4-yl)benzoate (3i)

^{1}H NMR (500 MHz)

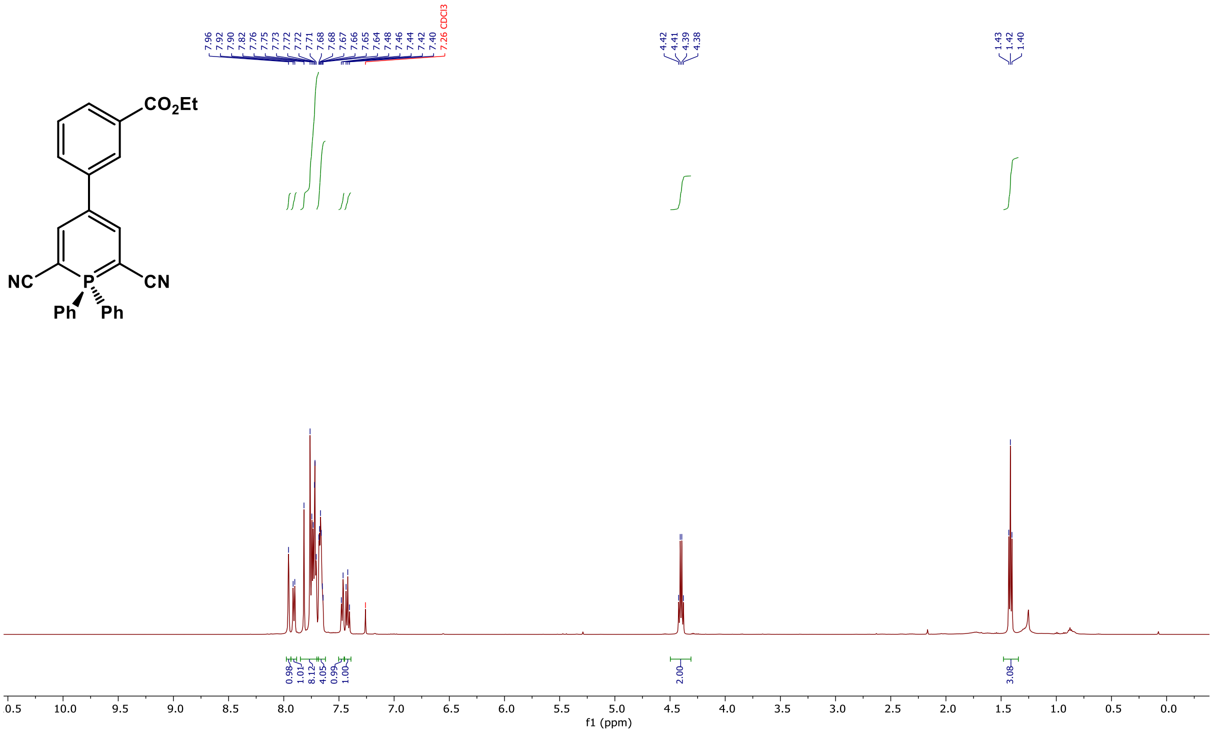

^{13}C NMR (126 MHz)

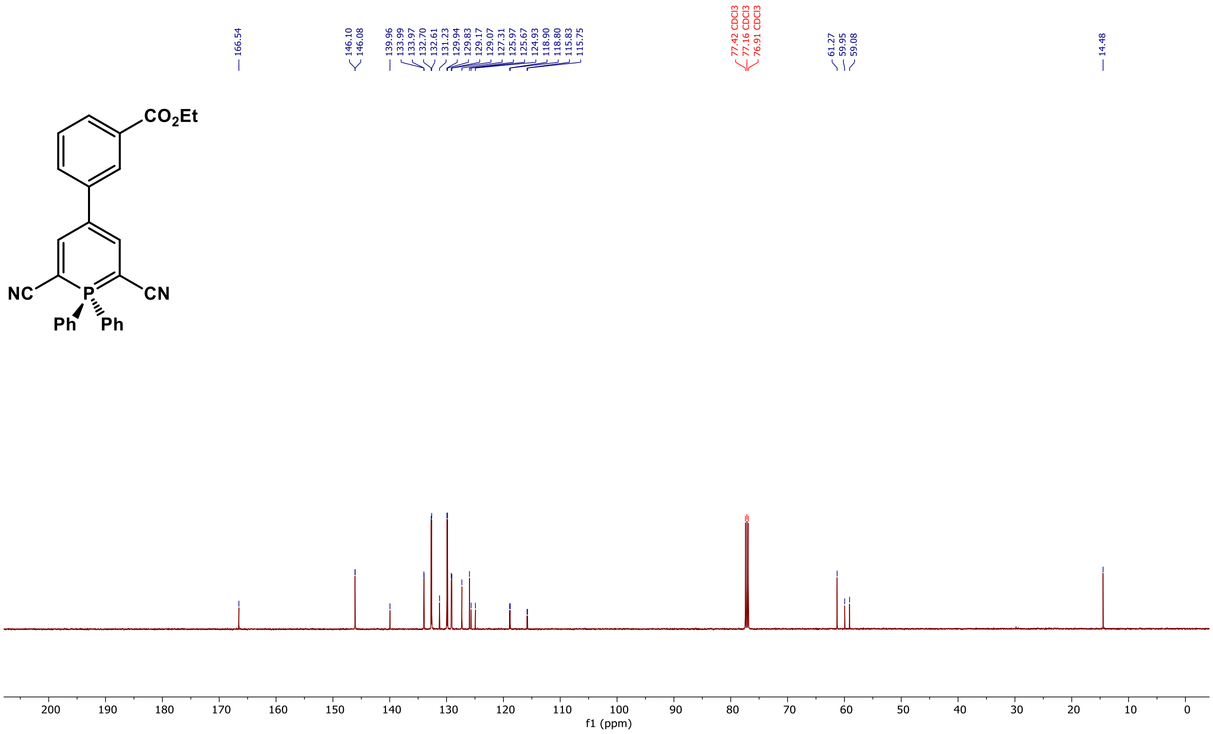

^{31}P NMR (202 MHz)

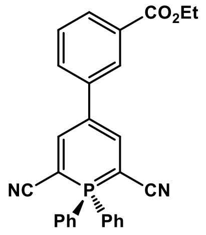

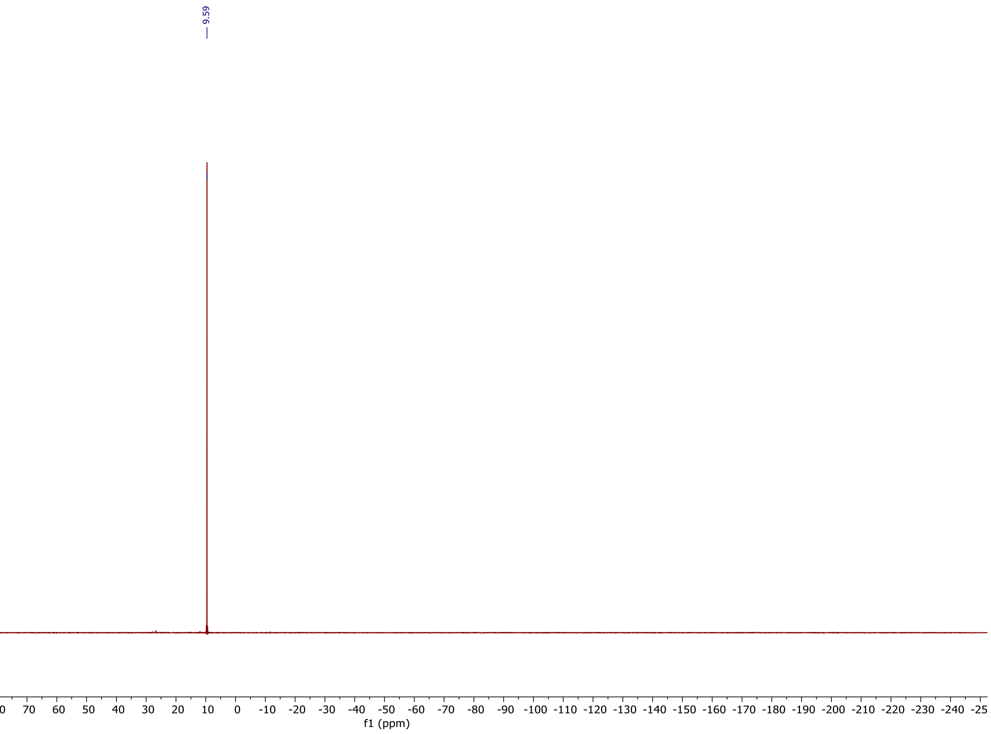

4-(benzo[*b*]thiophen-3-yl)-1,1-diphenyl-1λ^5-phosphinine-2,6-dicarbonitrile (3j)

^{1}H NMR (500 MHz)

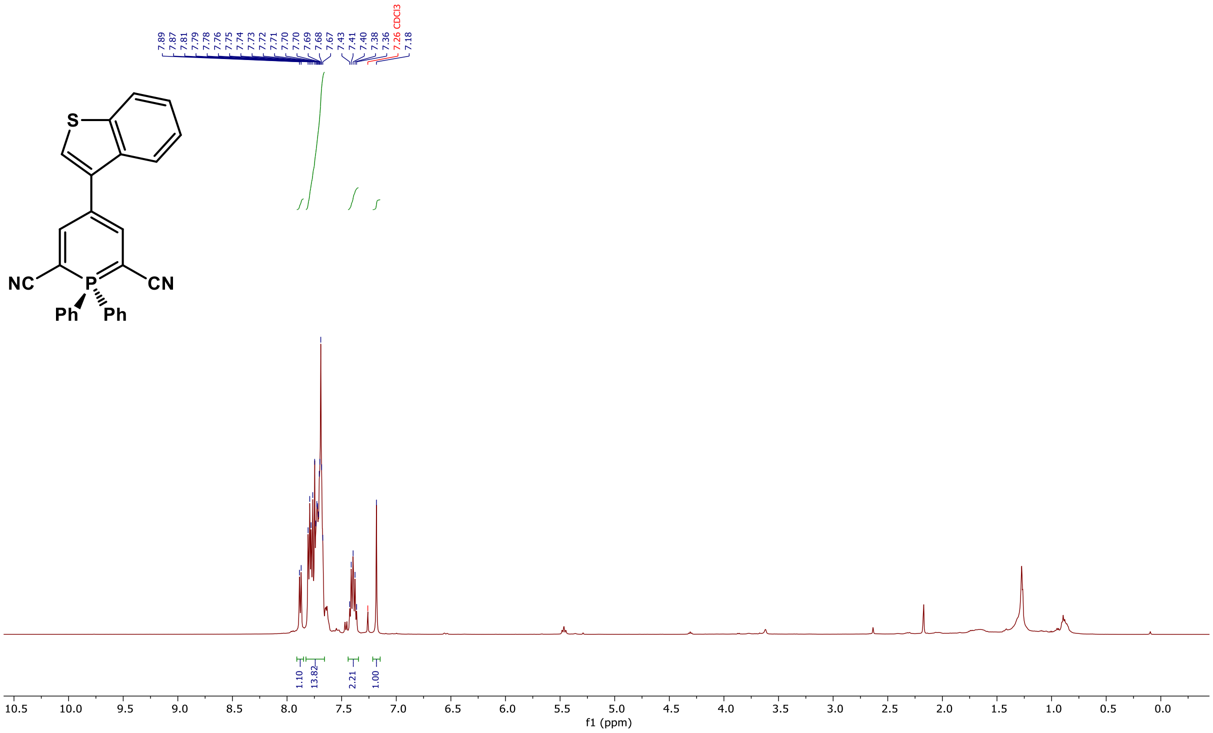

^{13}C NMR (126 MHz)

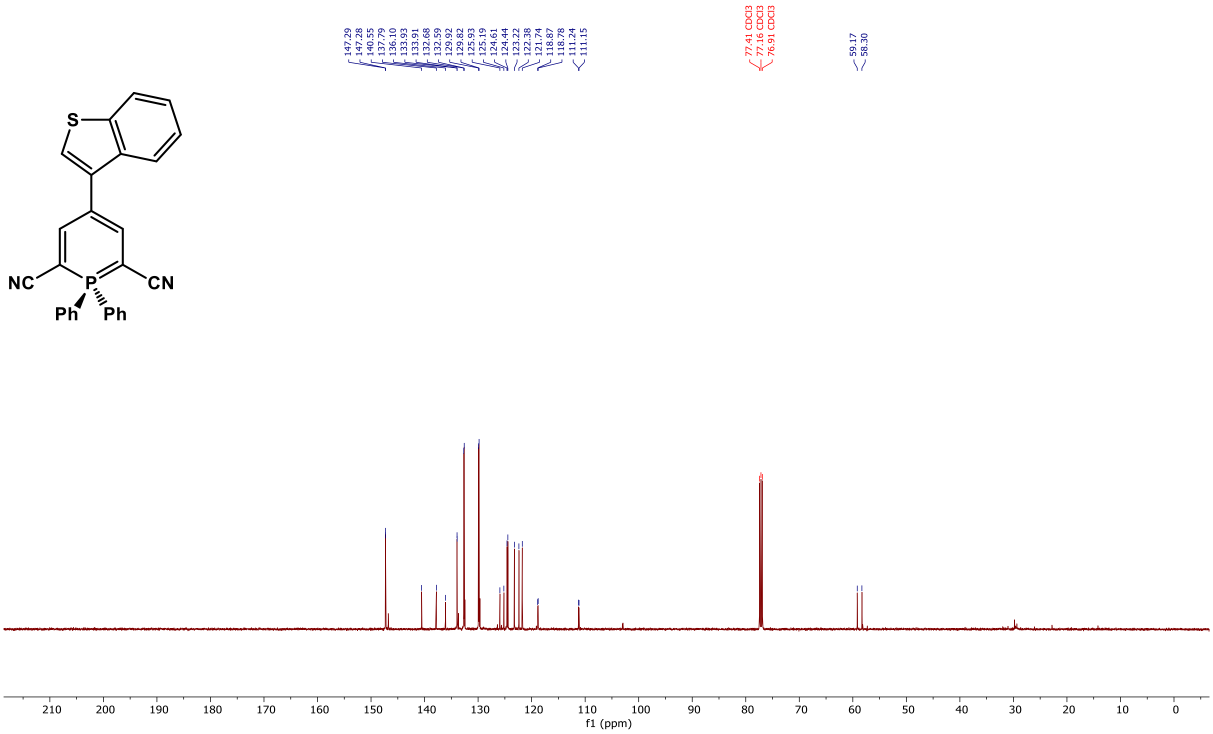

^{31}P NMR (202 MHz)

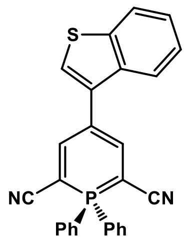

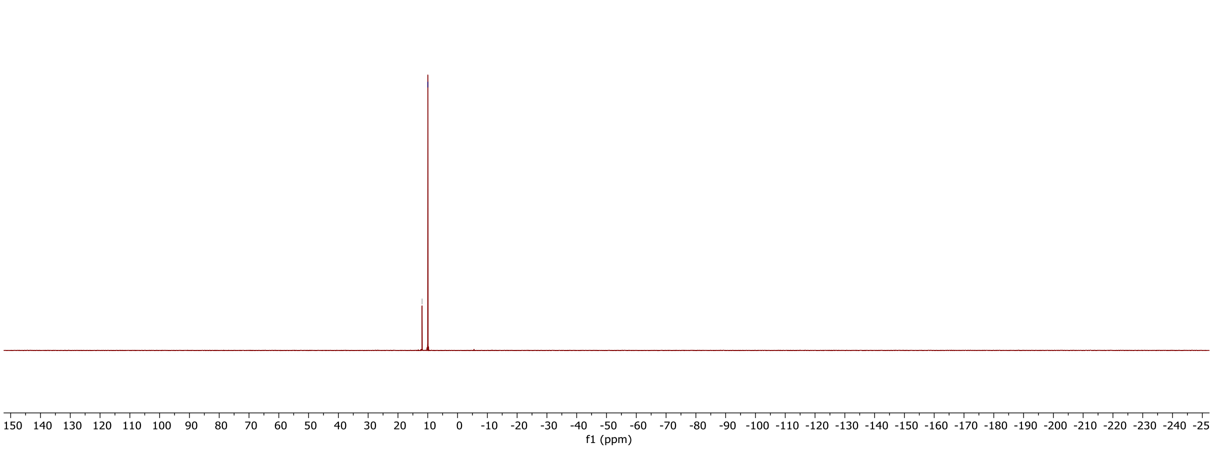

1,1-diphenyl-4-(9-phenyl-9*H*-carbazol-3-yl)-1λ^5-phosphinine-2,6-dicarbonitrile (3k)

^{1}H NMR (500 MHz)

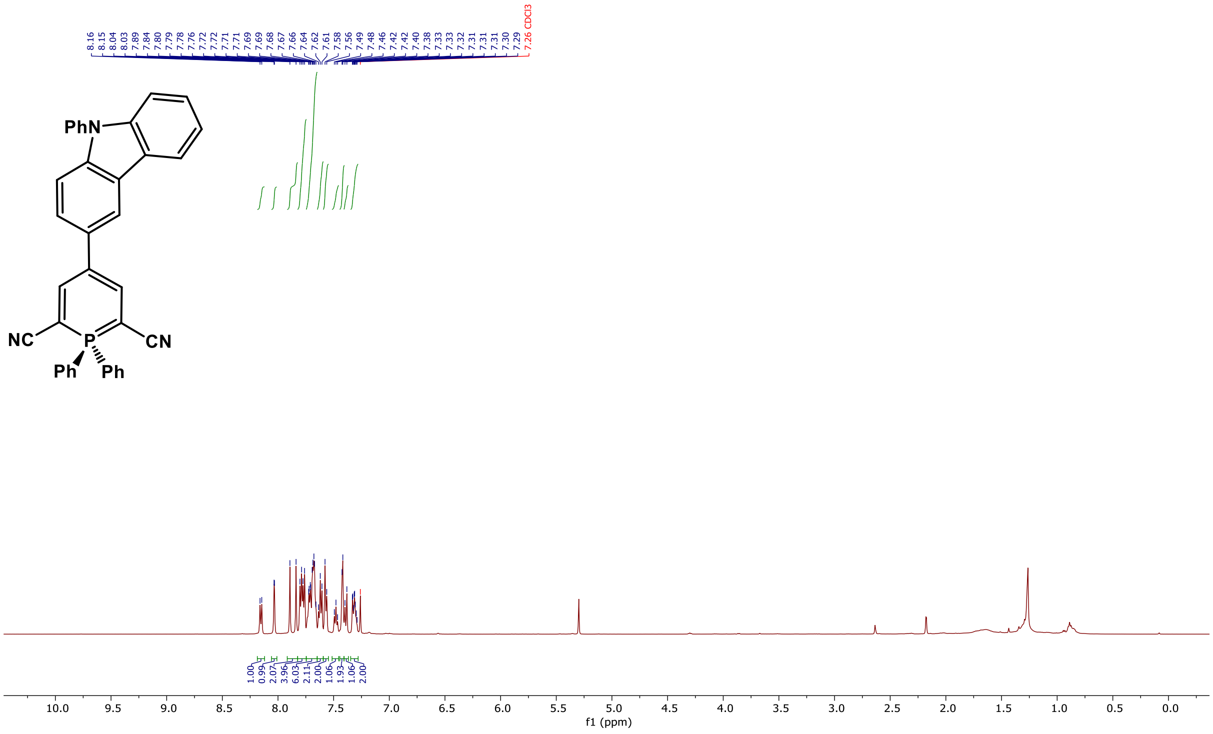

^{13}C NMR (126 MHz)

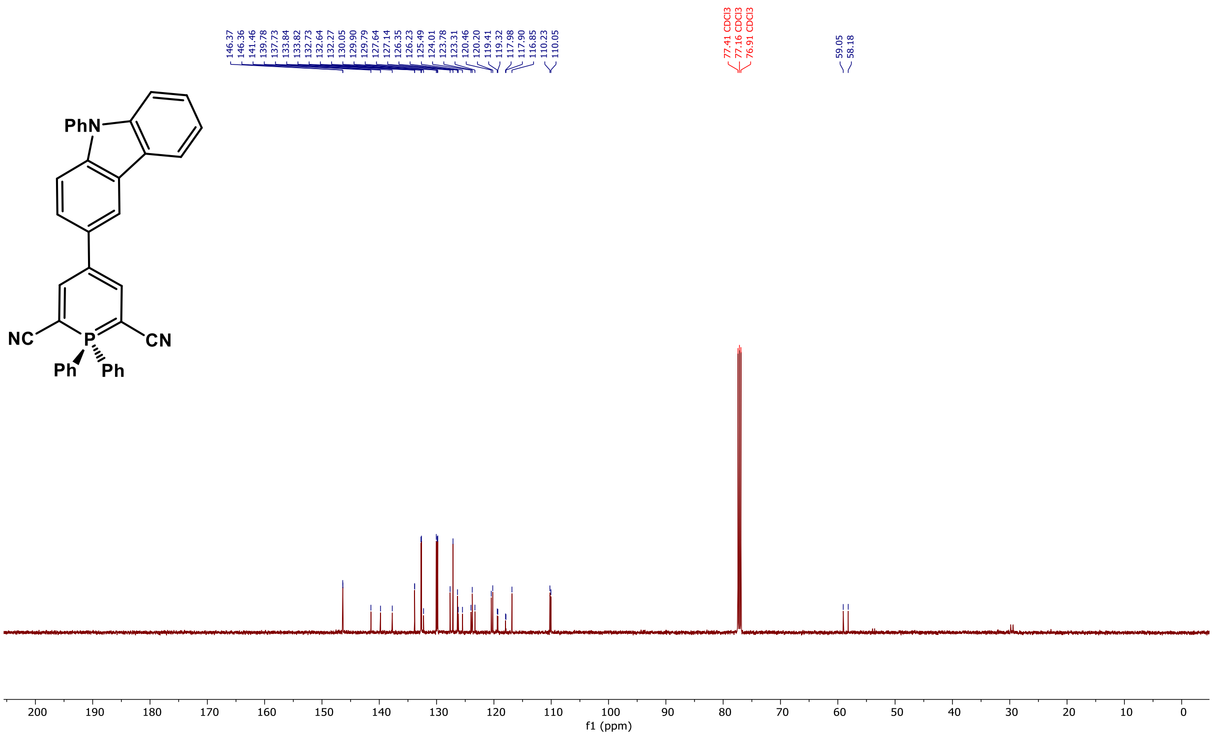

^{31}P NMR (202 MHz)

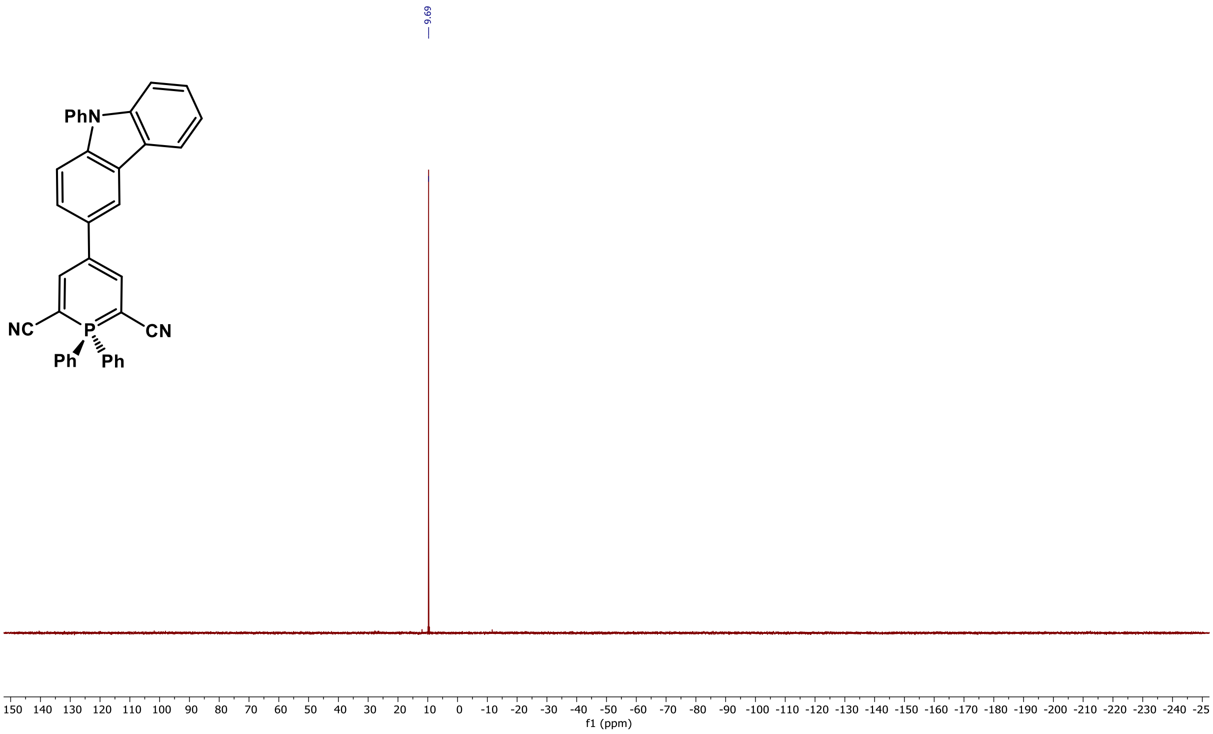

4-(dibenzo[*b*,*d*]thiophen-4-yl)-1,1-diphenyl-1λ^5-phosphinine-2,6-dicarbonitrile (3l)

^{1}H NMR (500 MHz)

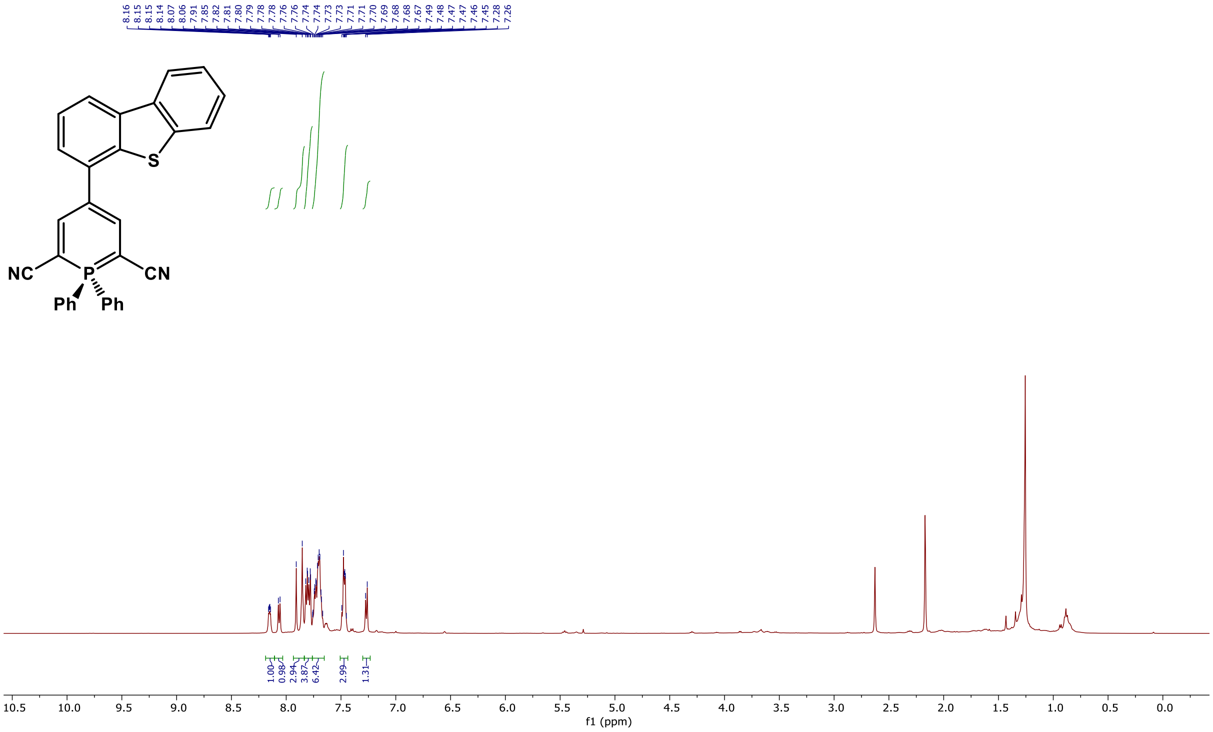

^{13}C NMR (126 MHz)

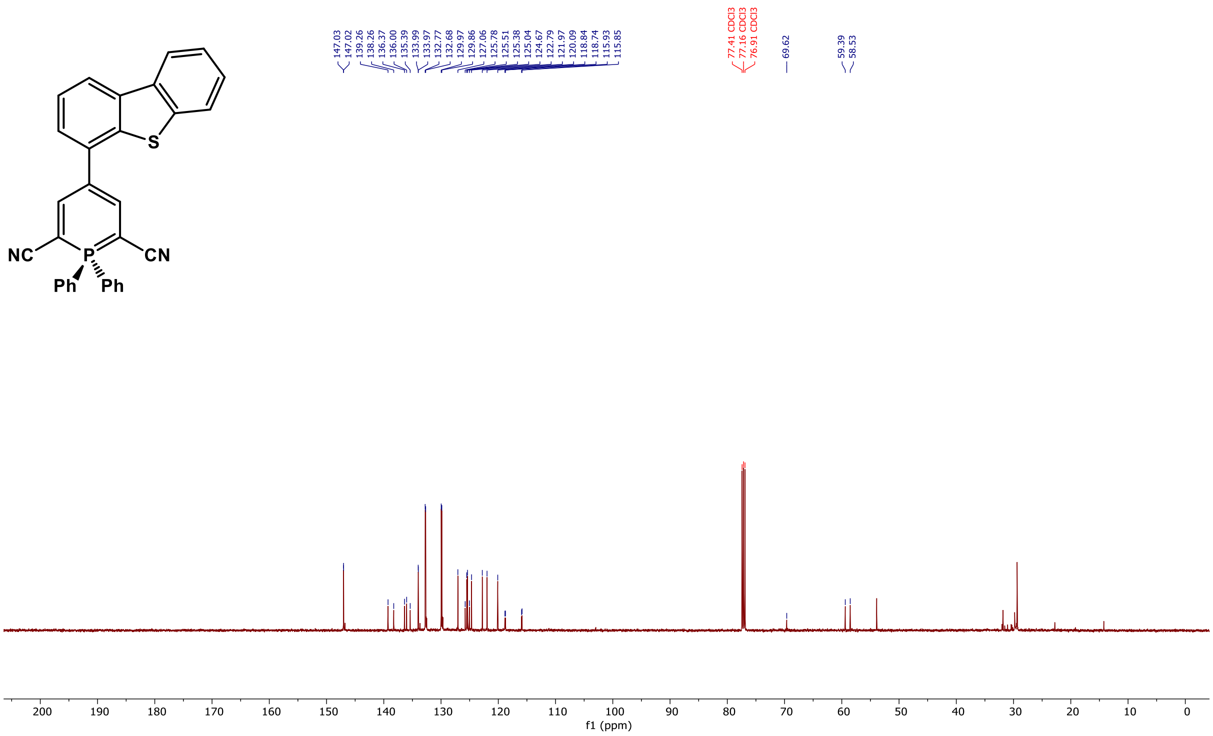

^{31}P NMR (202 MHz)

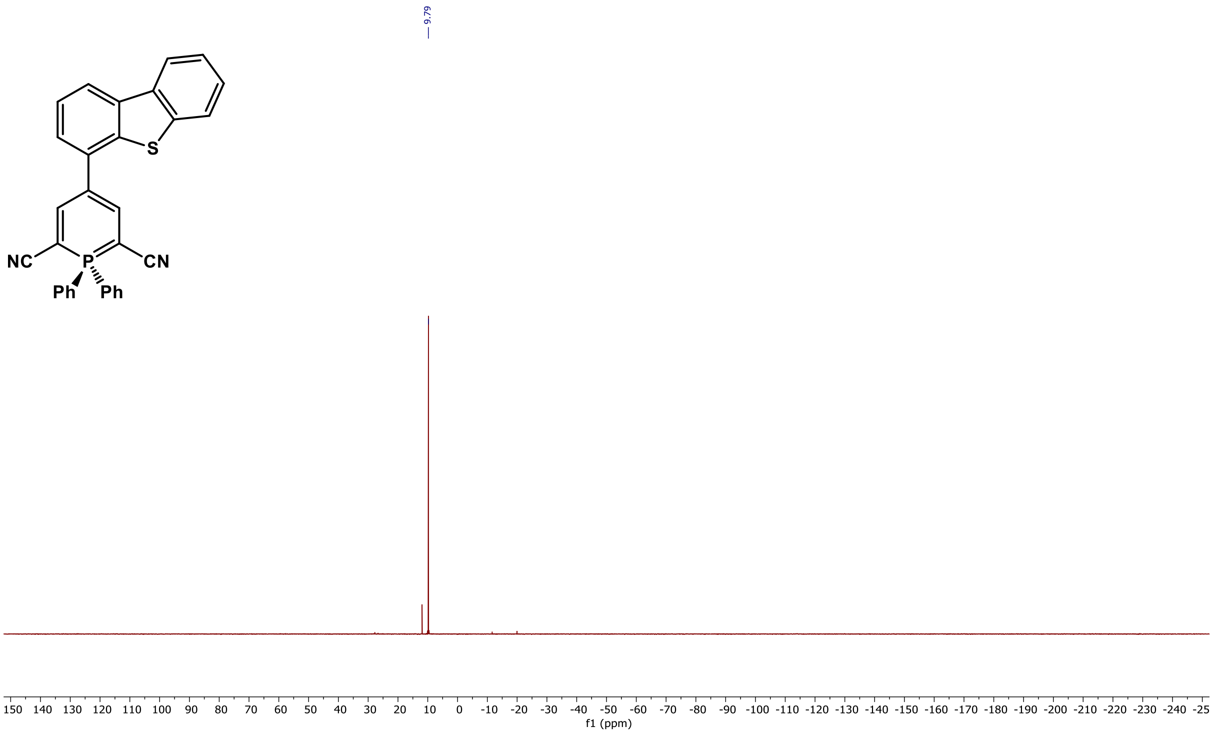

***tert*-butyl 3-(2,6-dicyano-1,1-diphenyl-1λ⁵-phosphinin-4-yl)-1*H*-pyrrolo[2,3-*c*]pyridine-1-carboxylate (3m)**

^{1}H NMR (500 MHz)

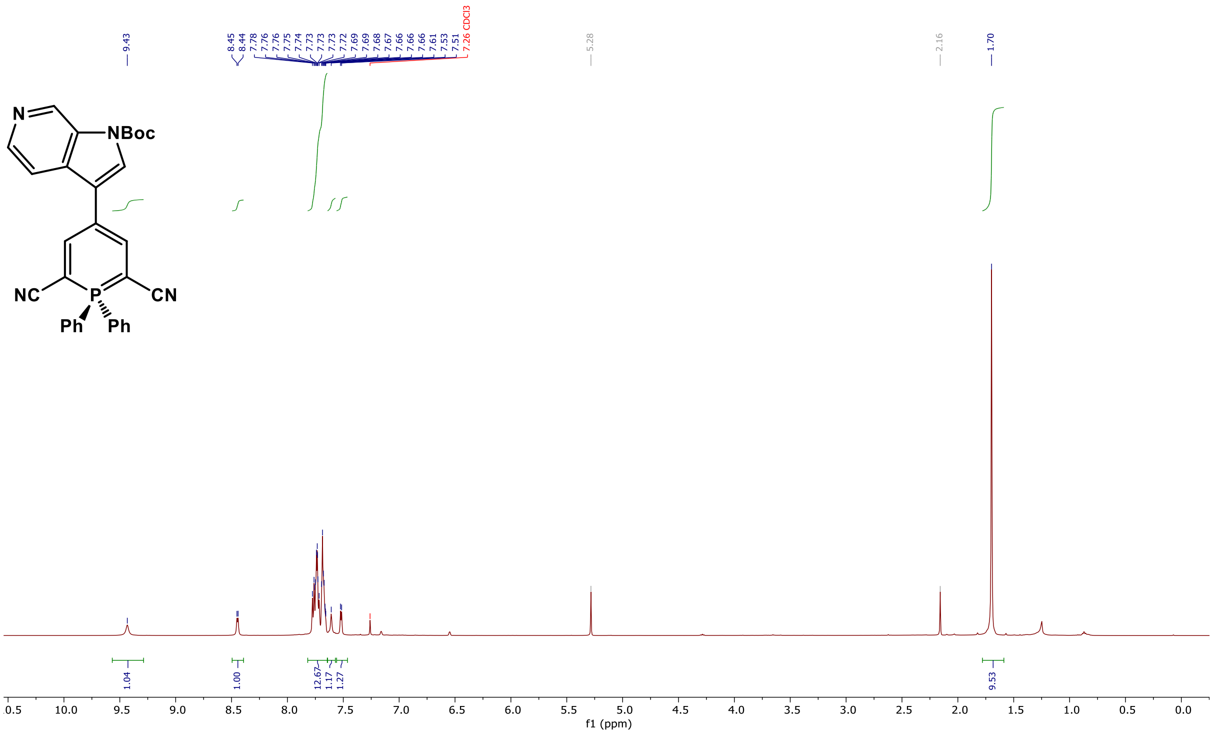

^{13}C NMR (126 MHz)

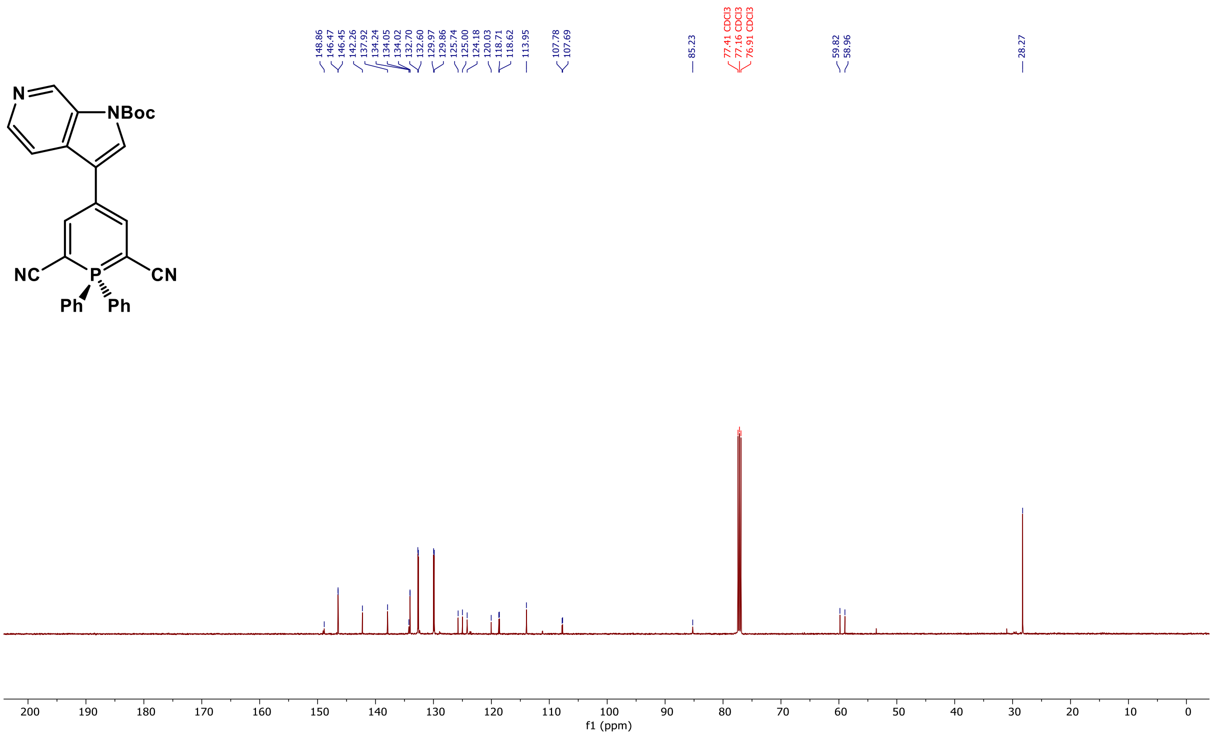

^{31}P NMR (202 MHz)

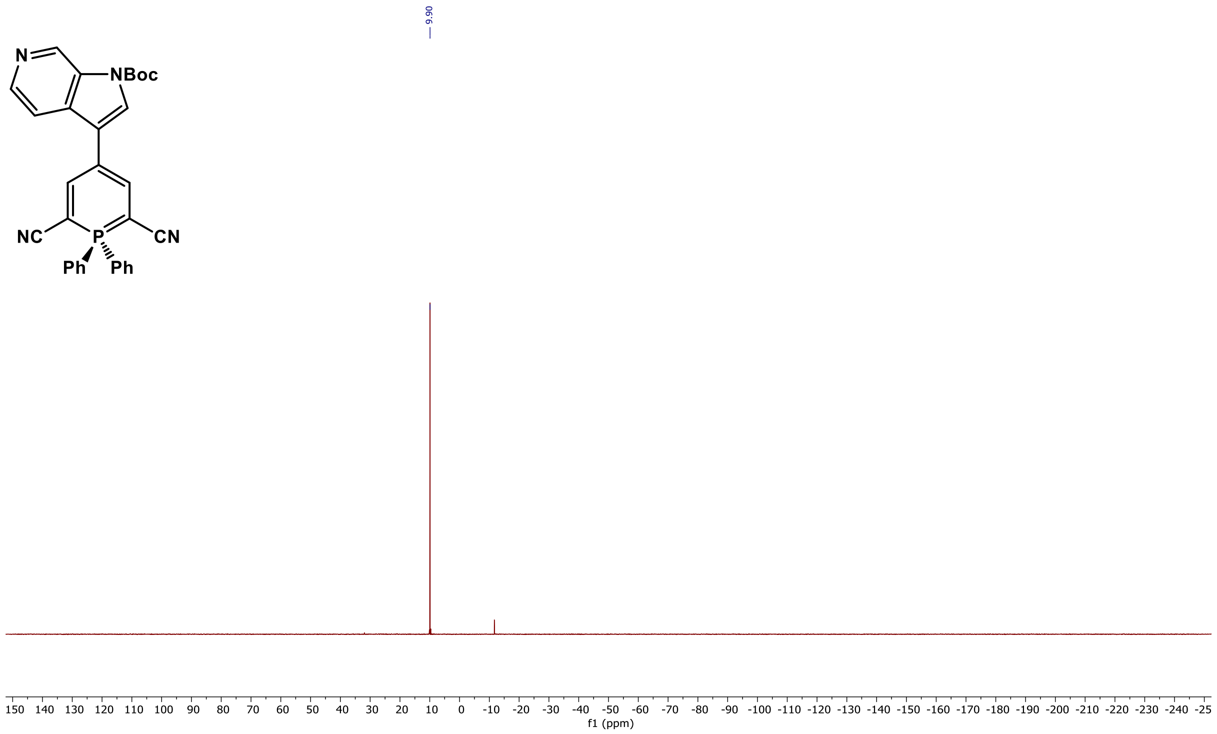

4-(2-bromothiazol-5-yl)-1,1-diphenyl-1λ^5-phosphinine-2,6-dicarbonitrile (3n)

^{1}H NMR (500 MHz)

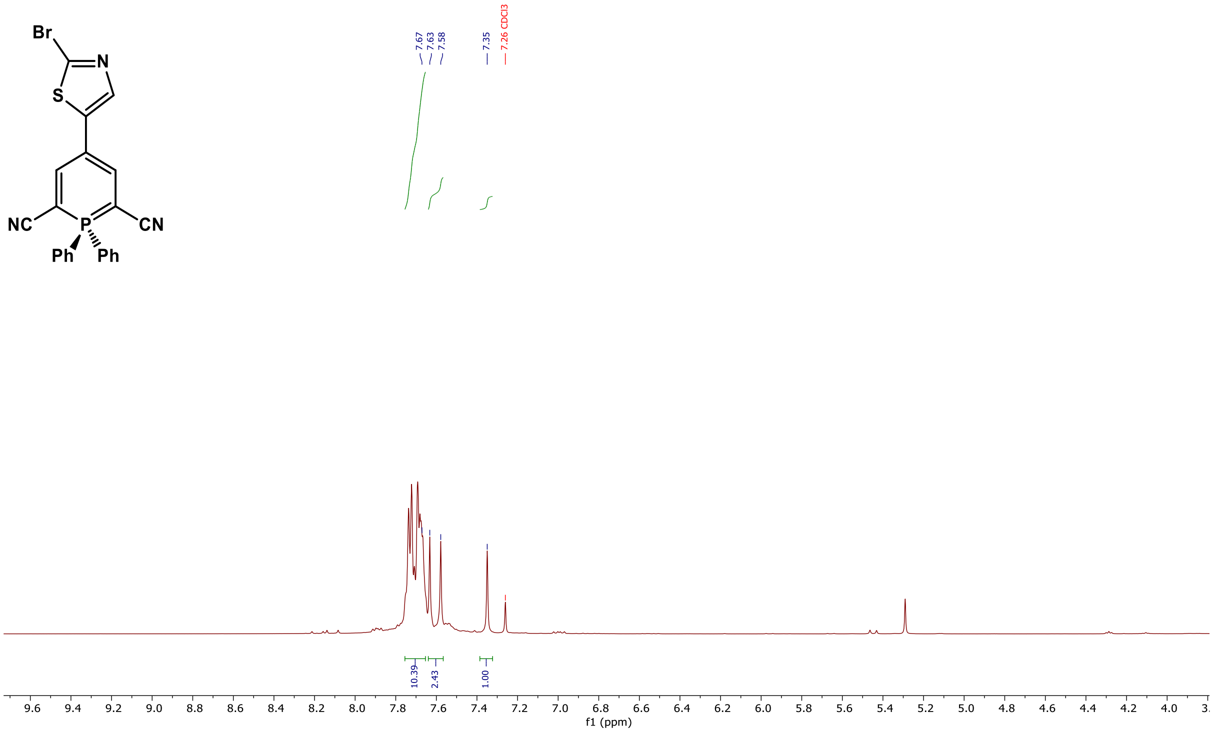

^{13}C NMR (126 MHz)

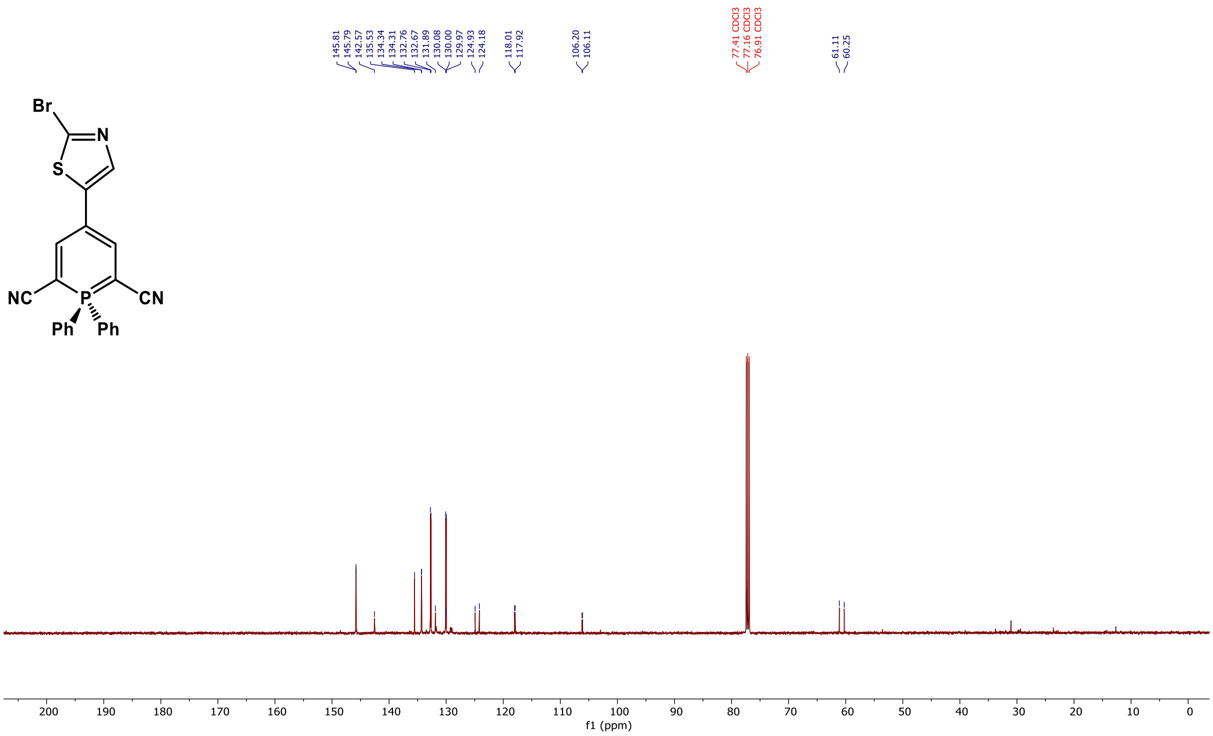

^{31}P NMR (202 MHz)

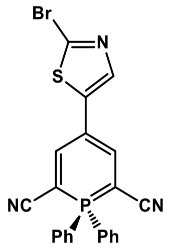

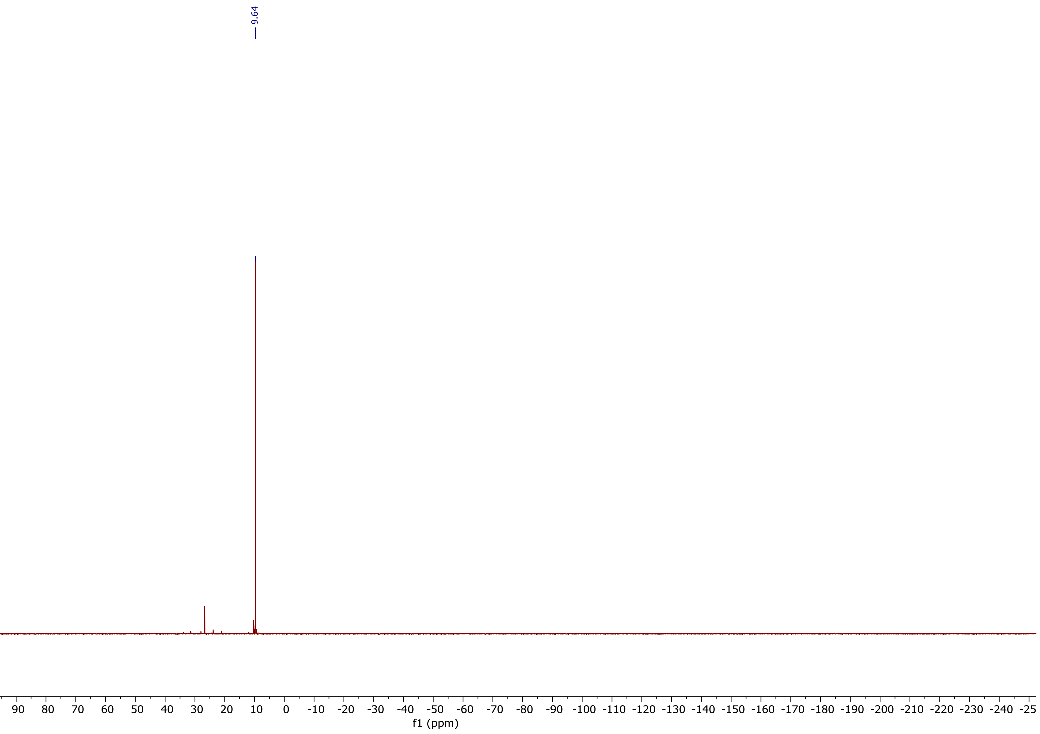

4-(5-cyano-1-tosyl-1*H*-indol-3-yl)-1,1-diphenyl-1λ^5-phosphinine-2,6-dicarbonitrile (3o)

^{1}H NMR (500 MHz)

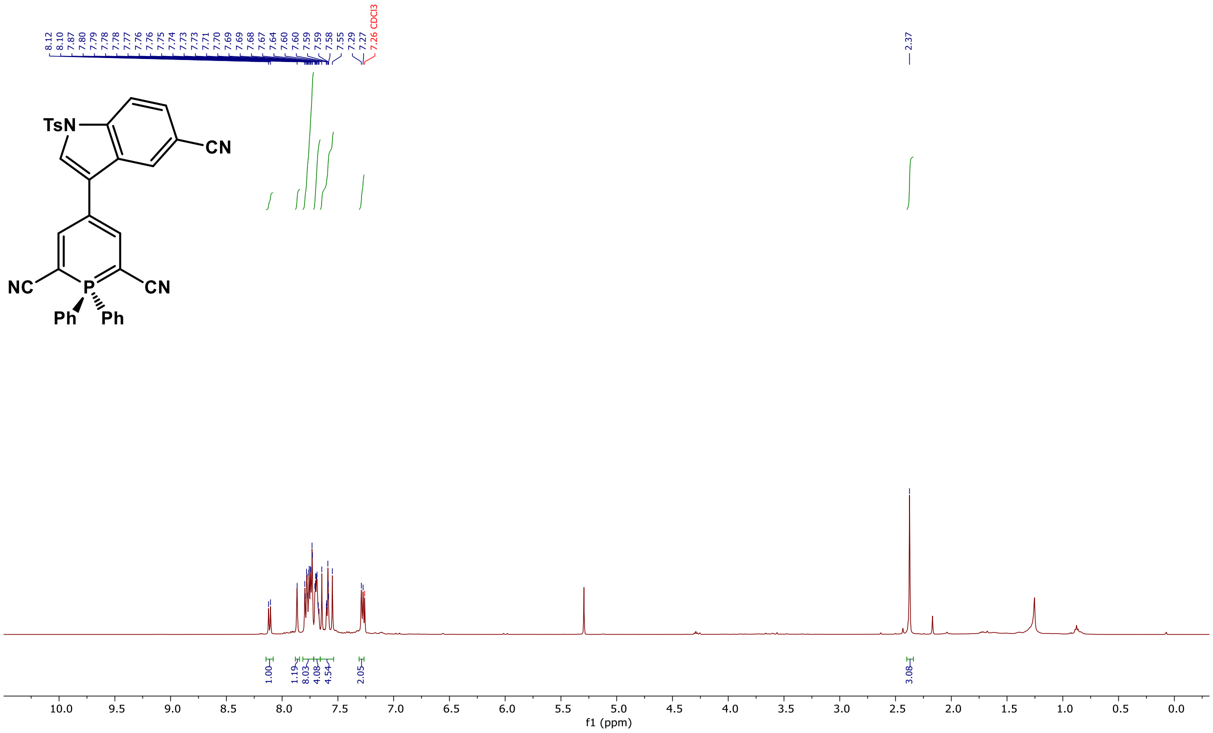

^{13}C NMR (126 MHz)

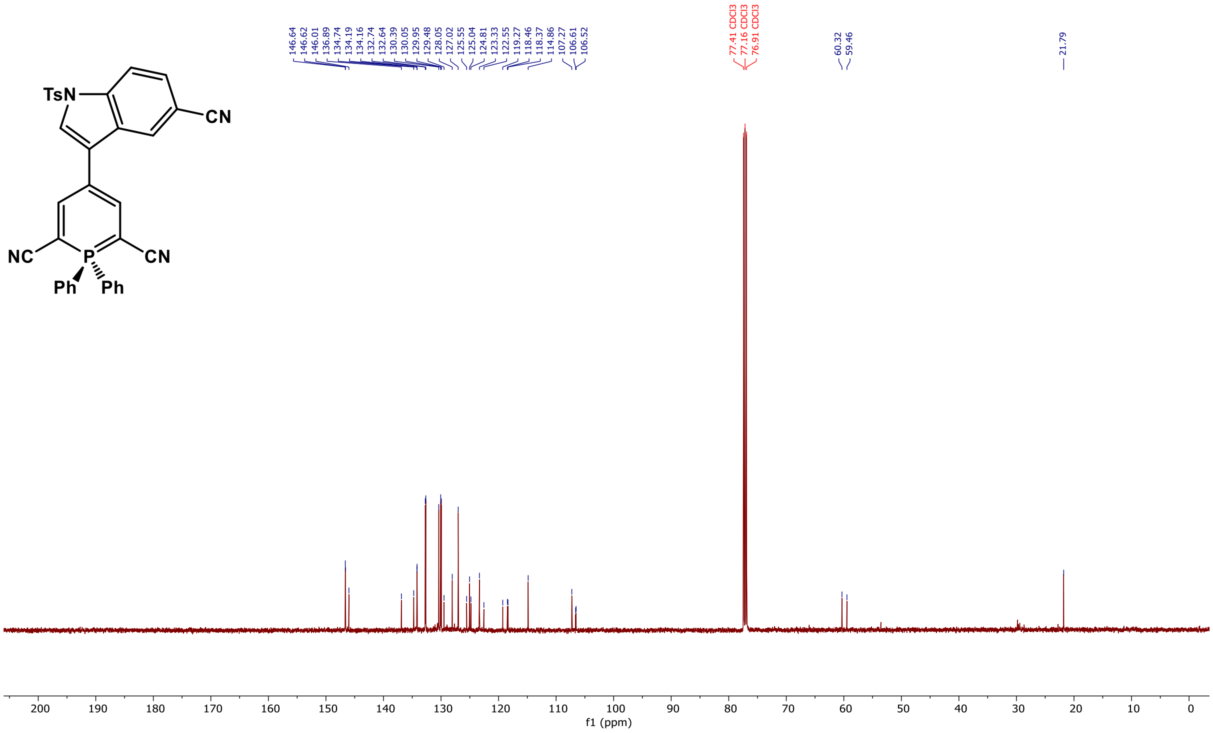

^{31}P NMR (202 MHz)

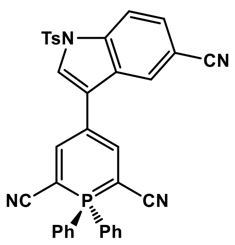

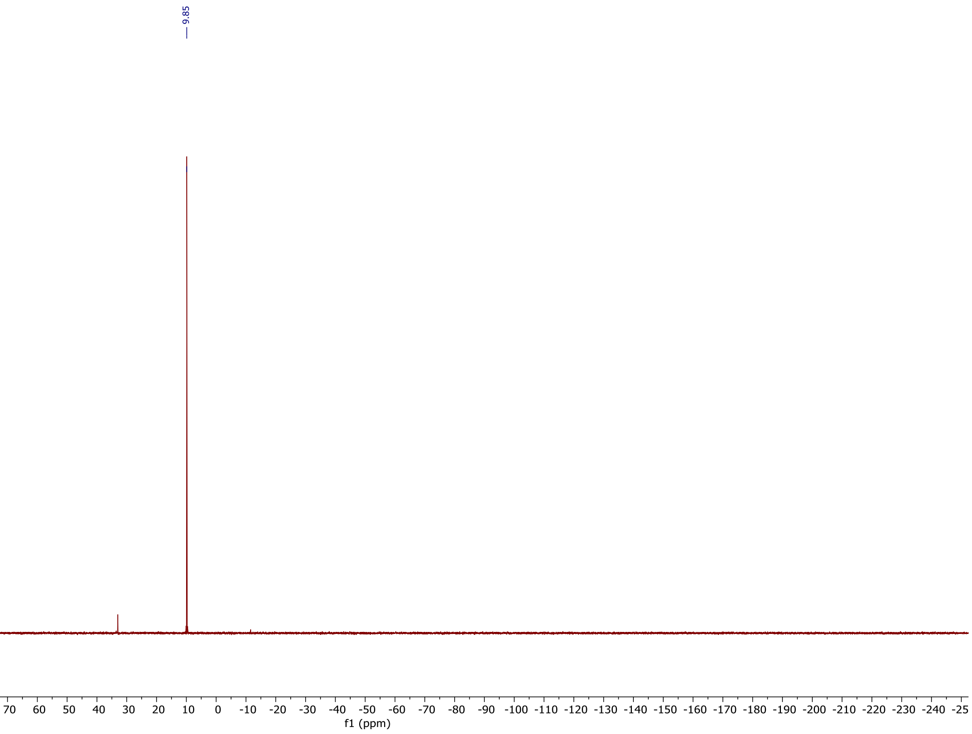

1,1-diphenyl-4-(thiophen-3-yl)-1λ^5-phosphinine-2,6-dicarbonitrile (3p)

^{1}H NMR (500 MHz)

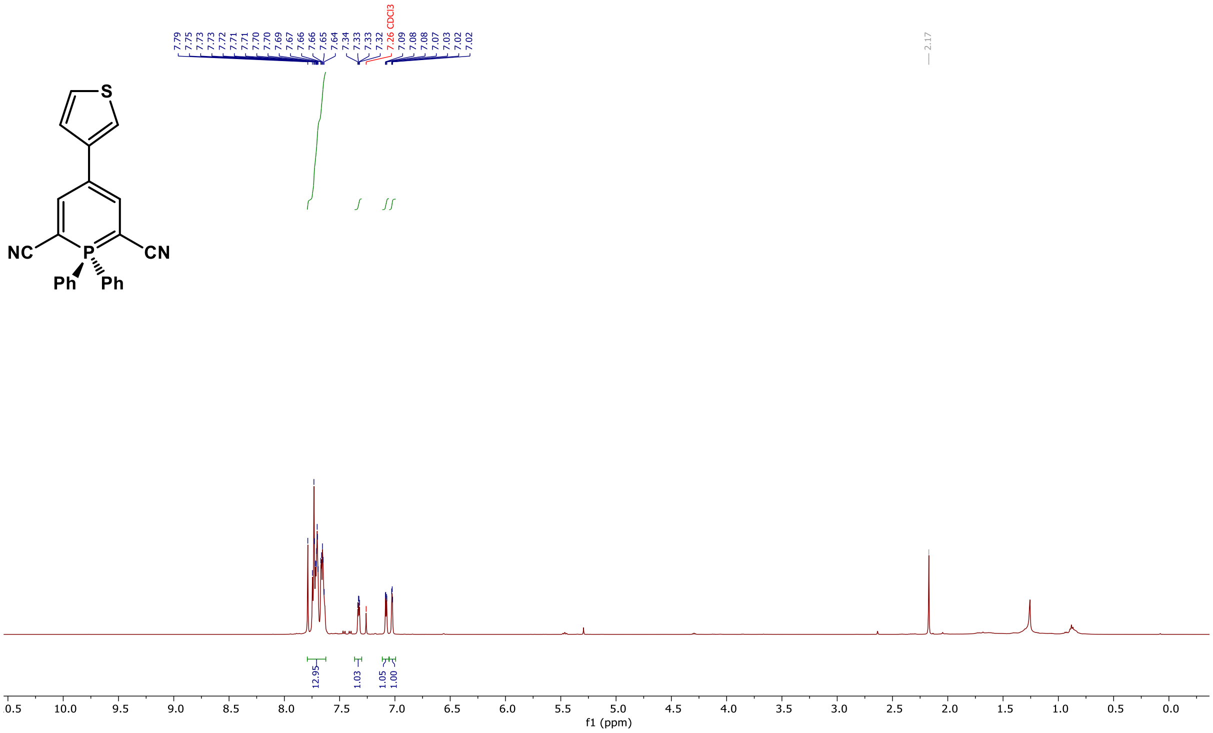

^{13}C NMR (126 MHz)

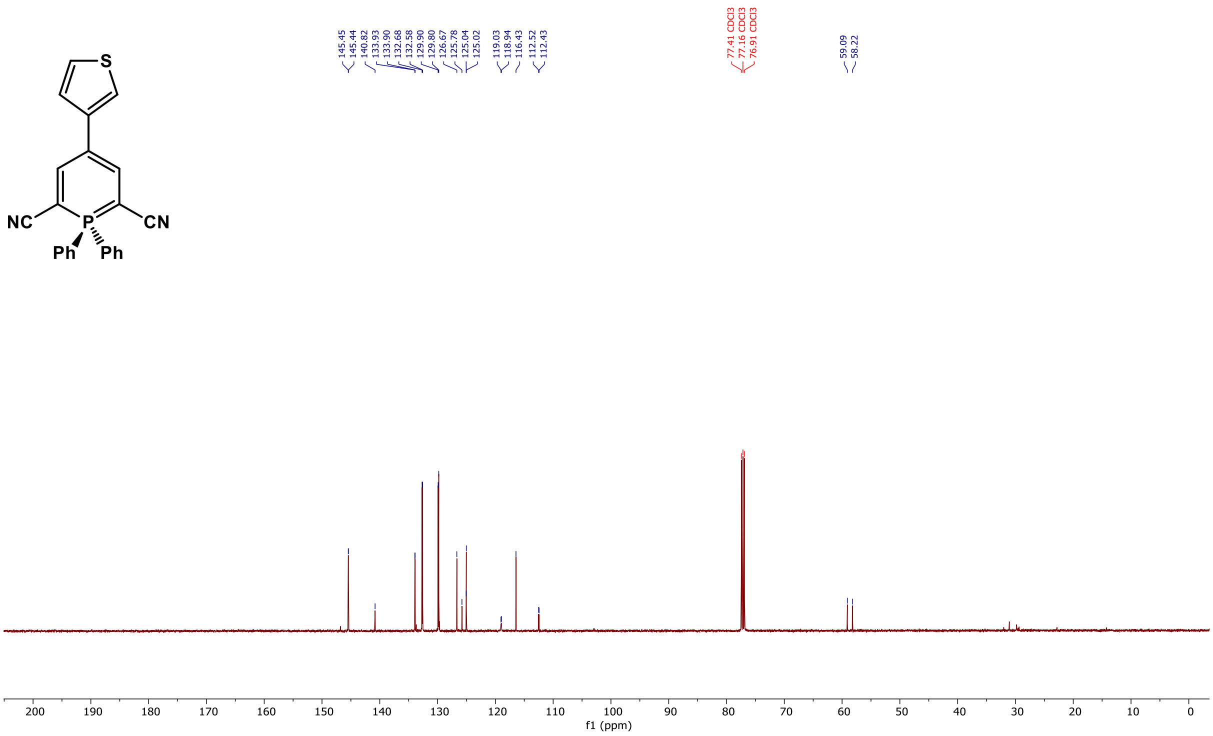

^{31}P NMR (202 MHz)

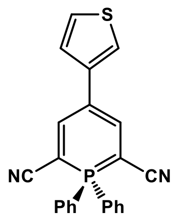

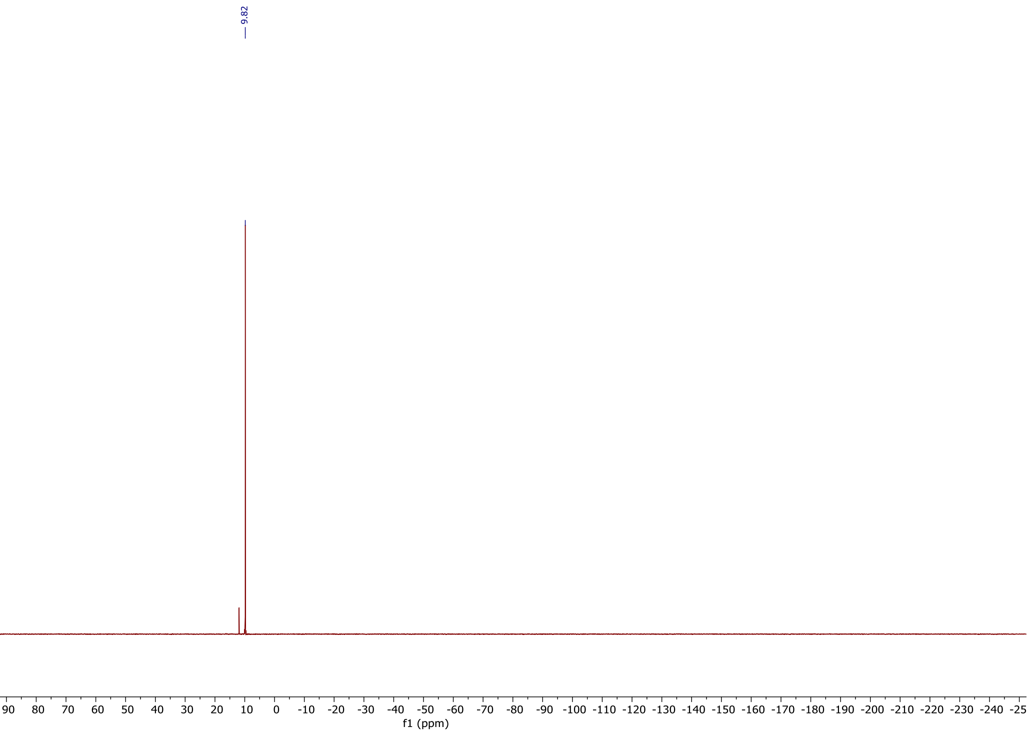

1,1-diphenyl-4-(pyrazin-2-yl)-1λ^5-phosphinine-2,6-dicarbonitrile (3q)

^{1}H NMR (500 MHz)

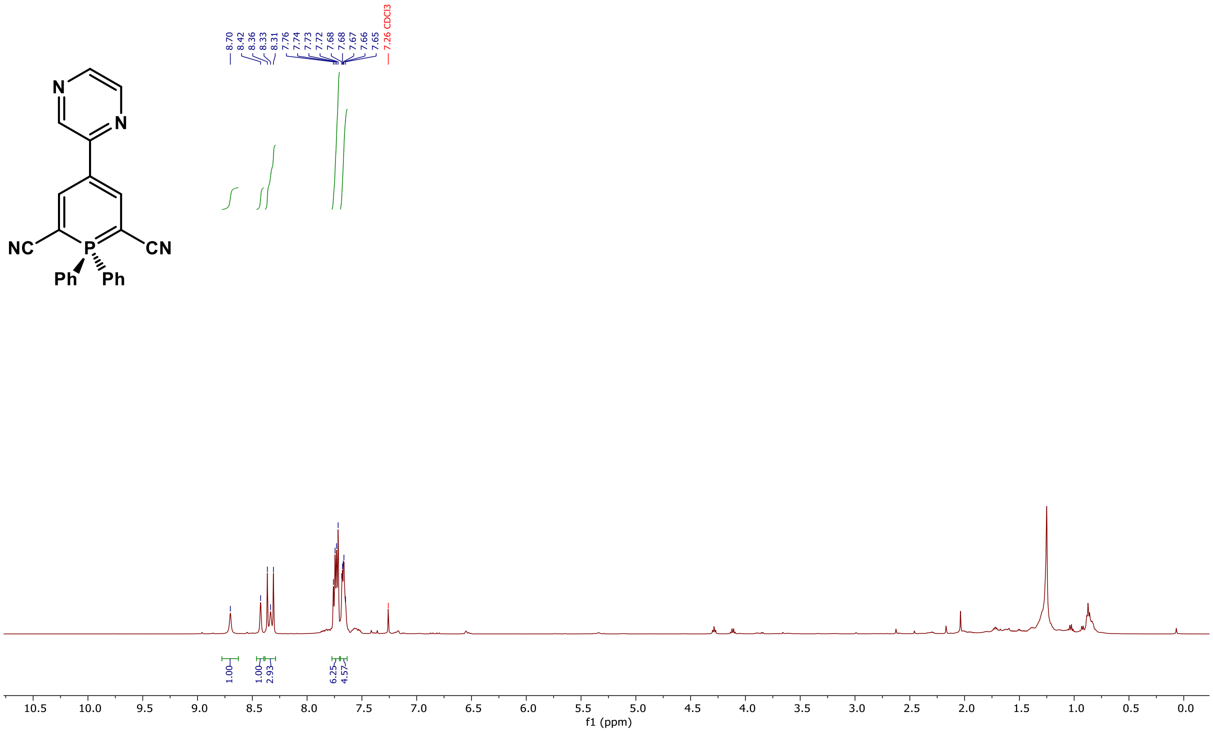

^{13}C NMR (126 MHz)

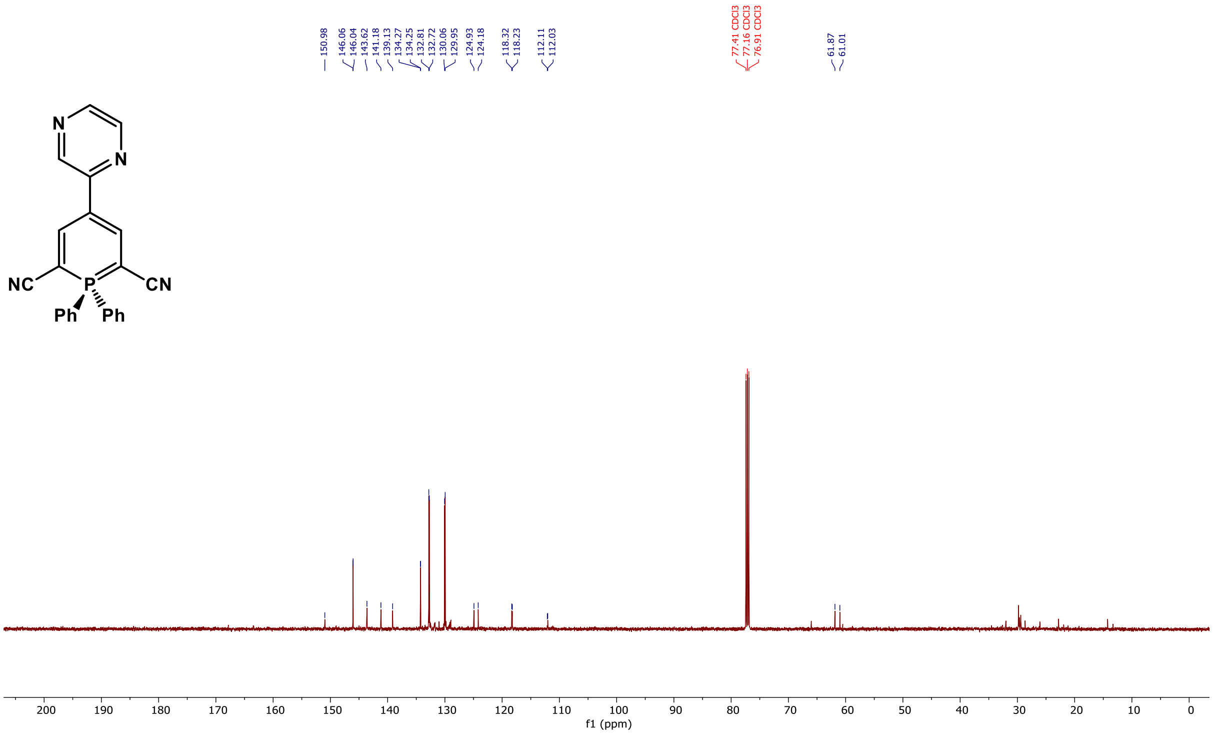

^{31}P NMR (202 MHz)

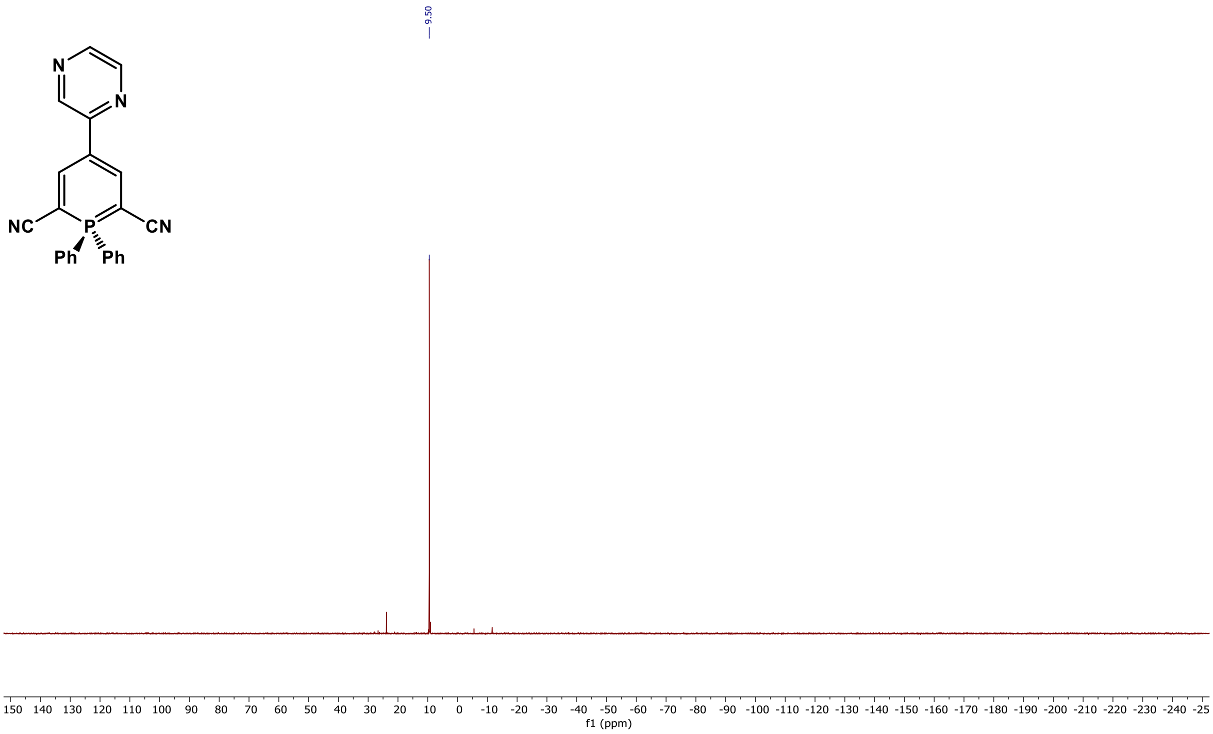

1,1-diphenyl-4-(quinolin-6-yl)-1λ^5-phosphinine-2,6-dicarbonitrile (3r)

^{1}H NMR (500 MHz)

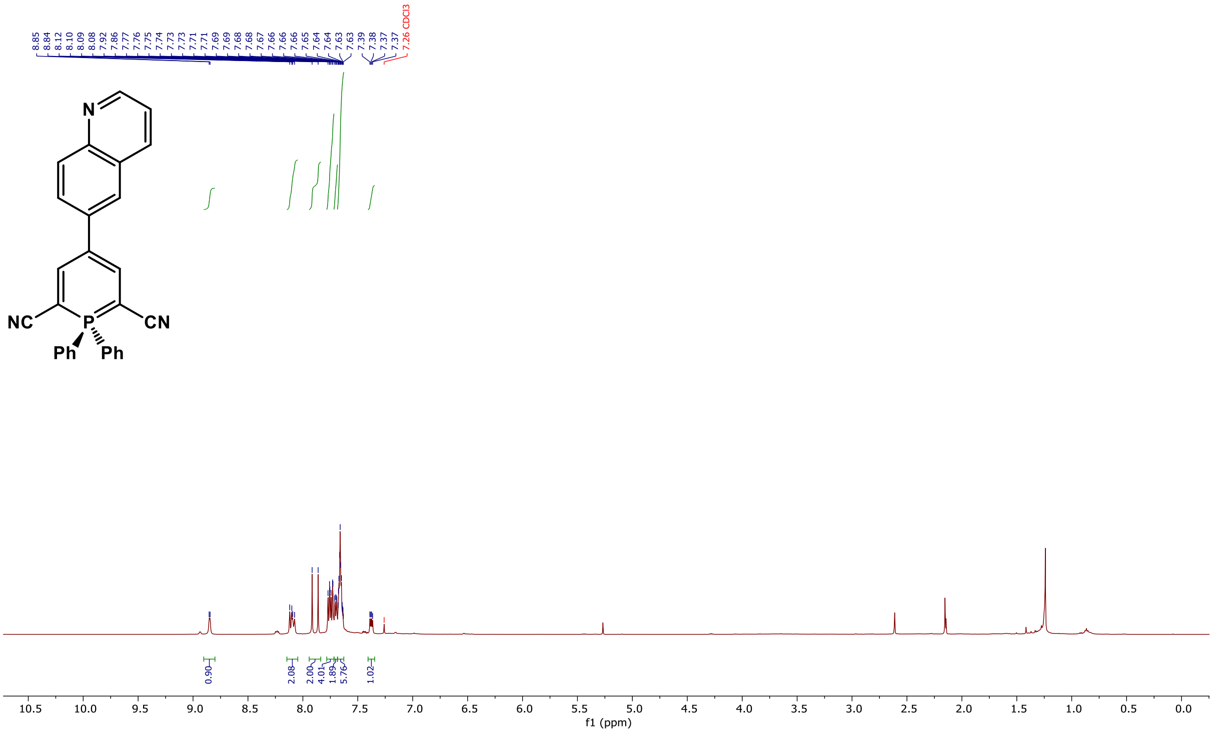

^{13}C NMR (126 MHz)

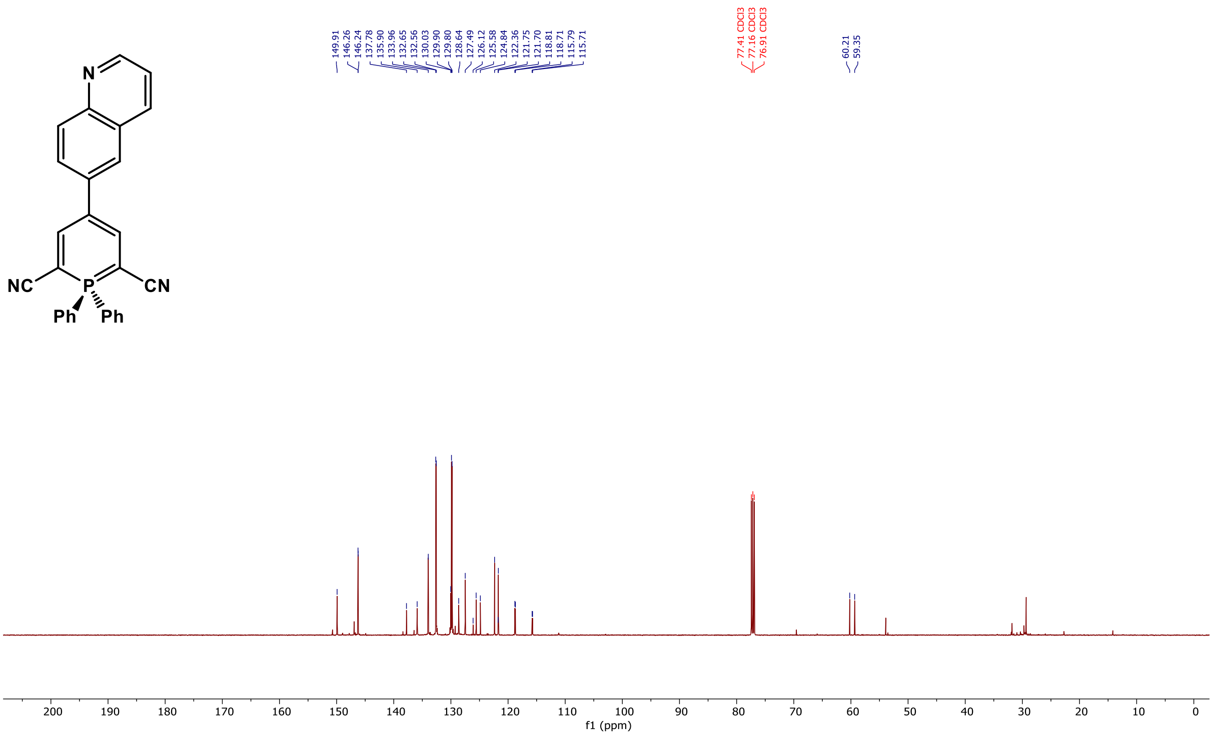

^{31}P NMR (202 MHz)

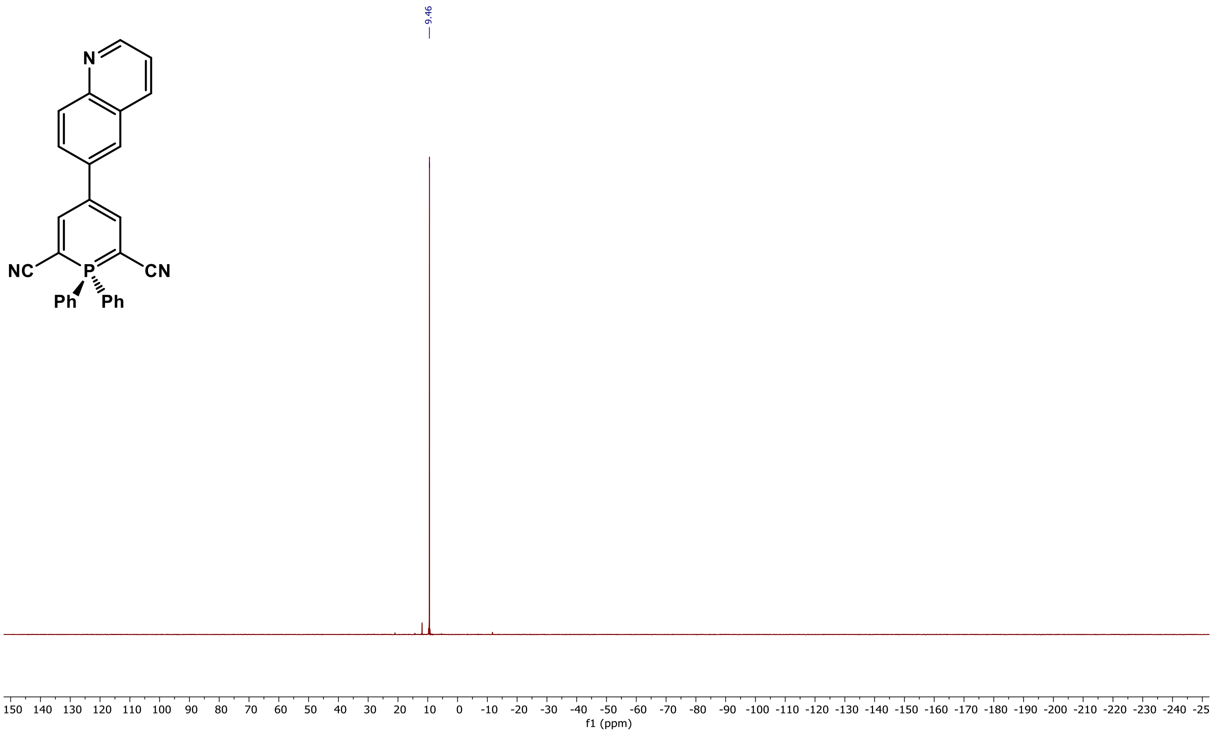

1,1,1',1'-tetraphenyl-1λ^{5},1'λ^{5}-[4,4'-biphosphinine]-2,2',6,6'-tetracarbonitrile (3s)

^{1}H NMR (500 MHz)

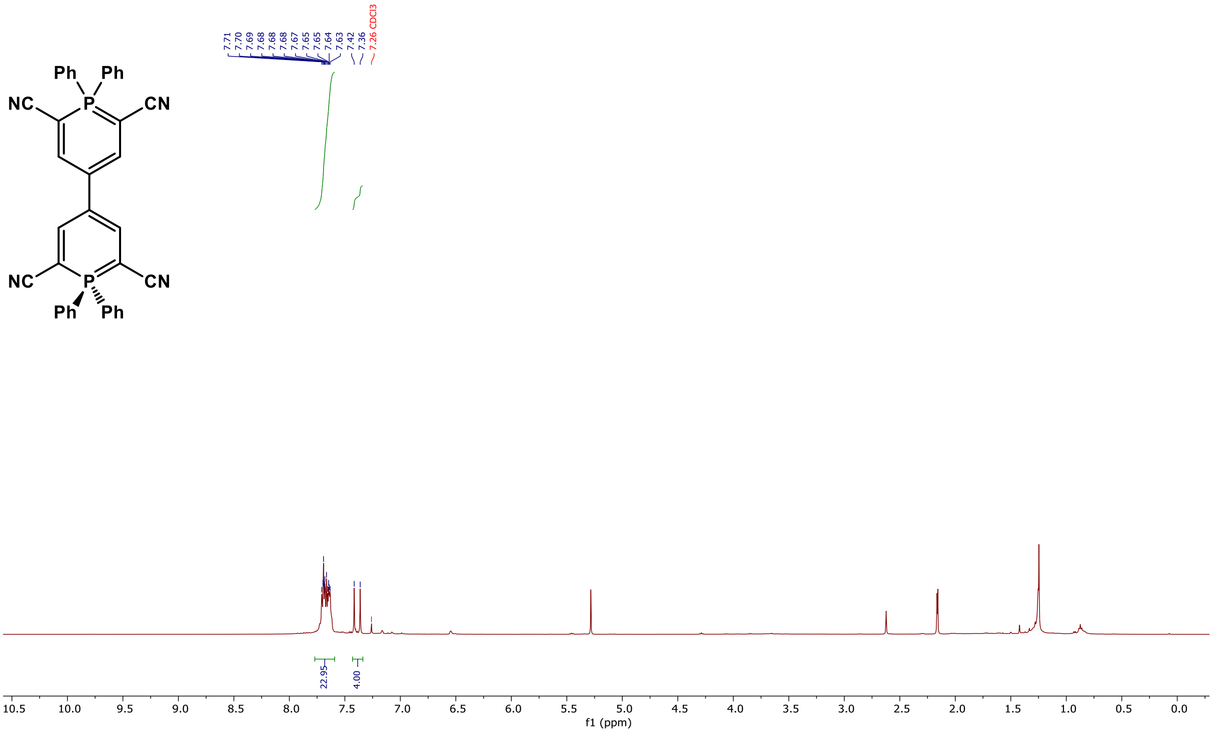

^{13}C NMR (126 MHz)

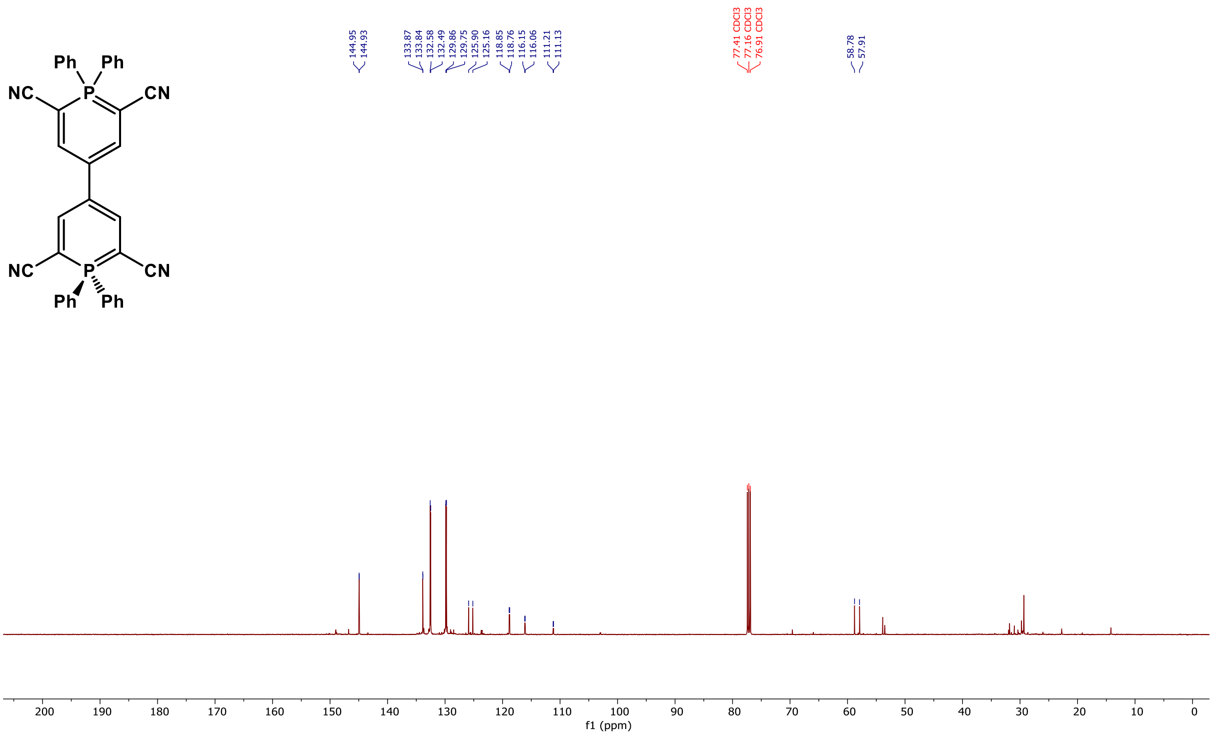

^{31}P NMR (202 MHz)

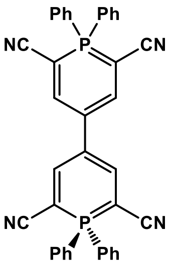

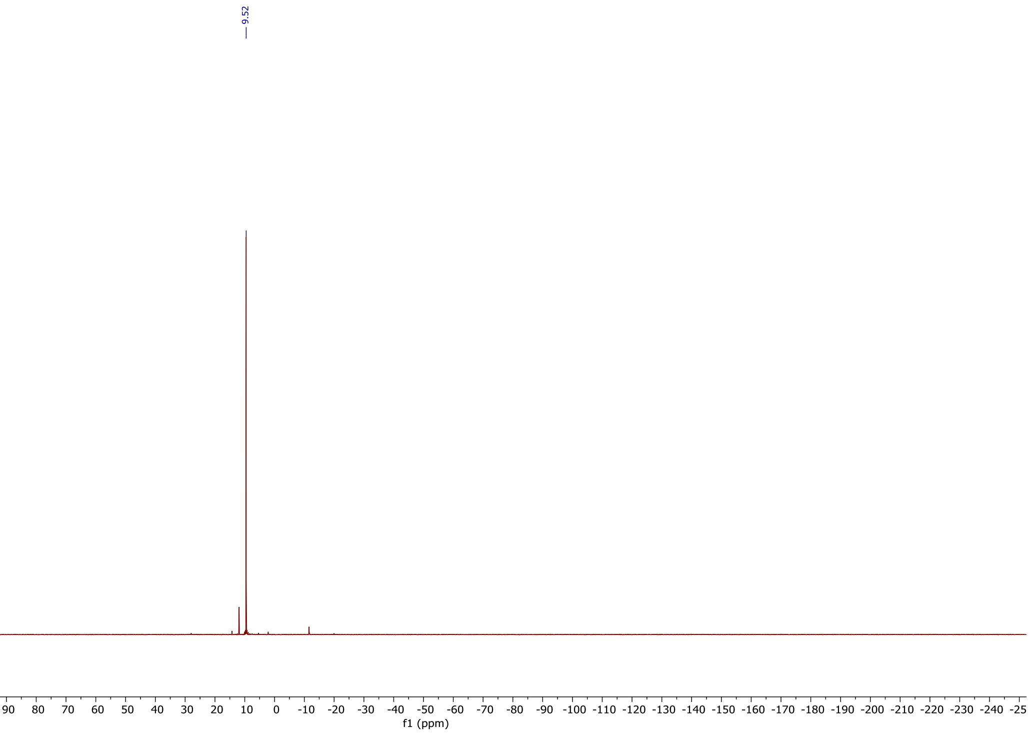

diethyl 2',6'-dicyano-1,1,1',1'-tetraphenyl-1λ^{5},1'λ^{5}-[4,4'-biphosphinine]-2,6-dicarboxylate (3t)

^{1}H NMR (500 MHz)

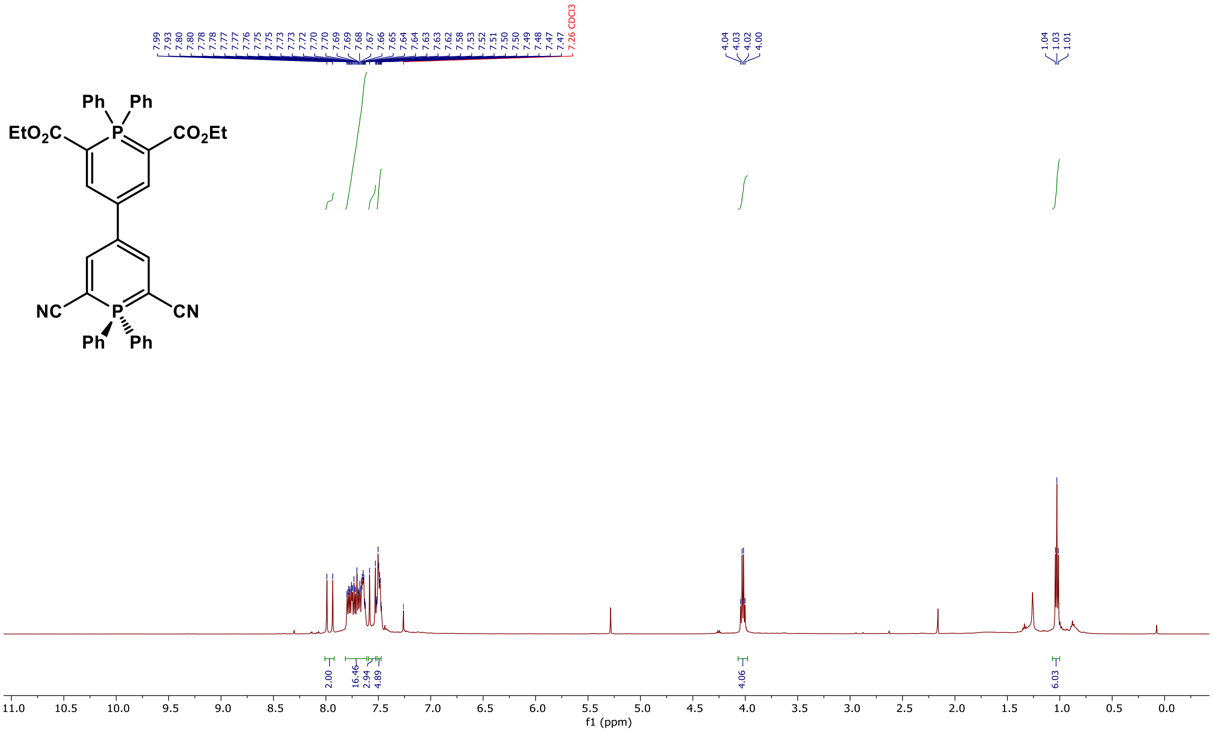

^{13}C NMR (126 MHz)

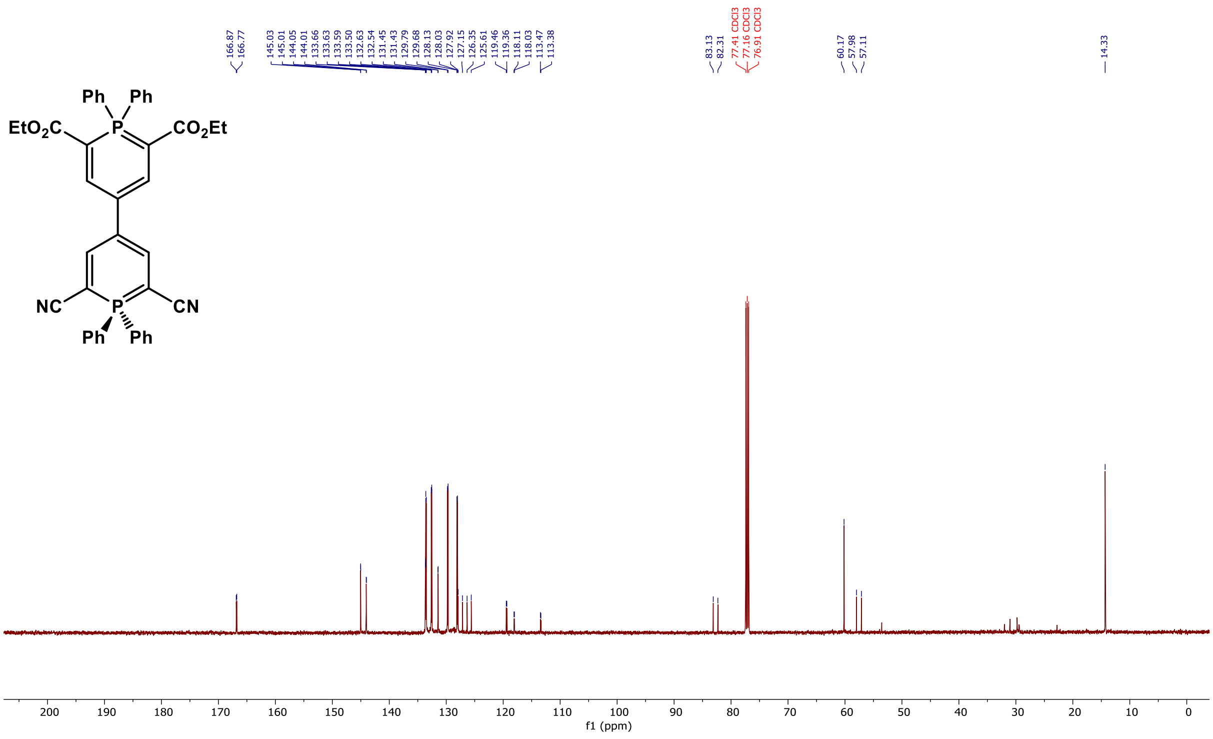

^{31}P NMR (202 MHz)

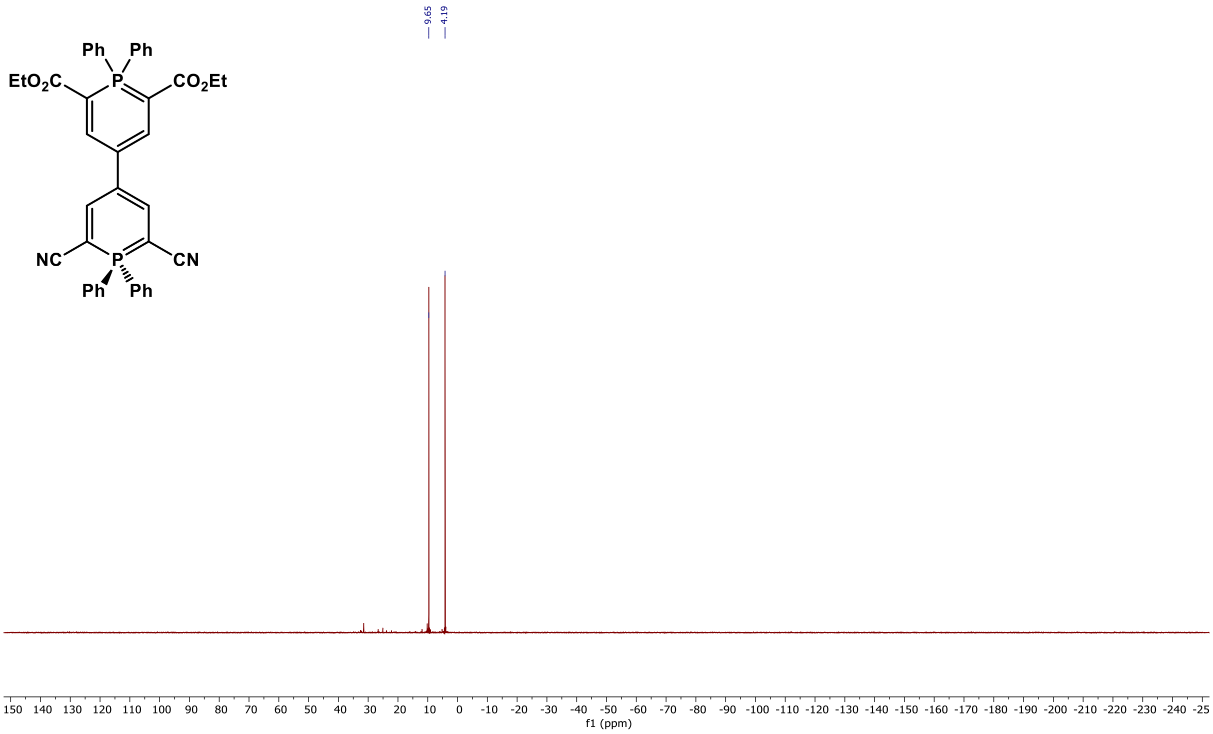

4-(3-methyl-2,4-dioxo-7-phenyl-3-azabicyclo[3.2.0]hept-6-en-6-yl)-1,1-diphenyl-1λ^5-phosphinine-2,6-dicarbonitrile (3u)

^{1}H NMR (500 MHz)

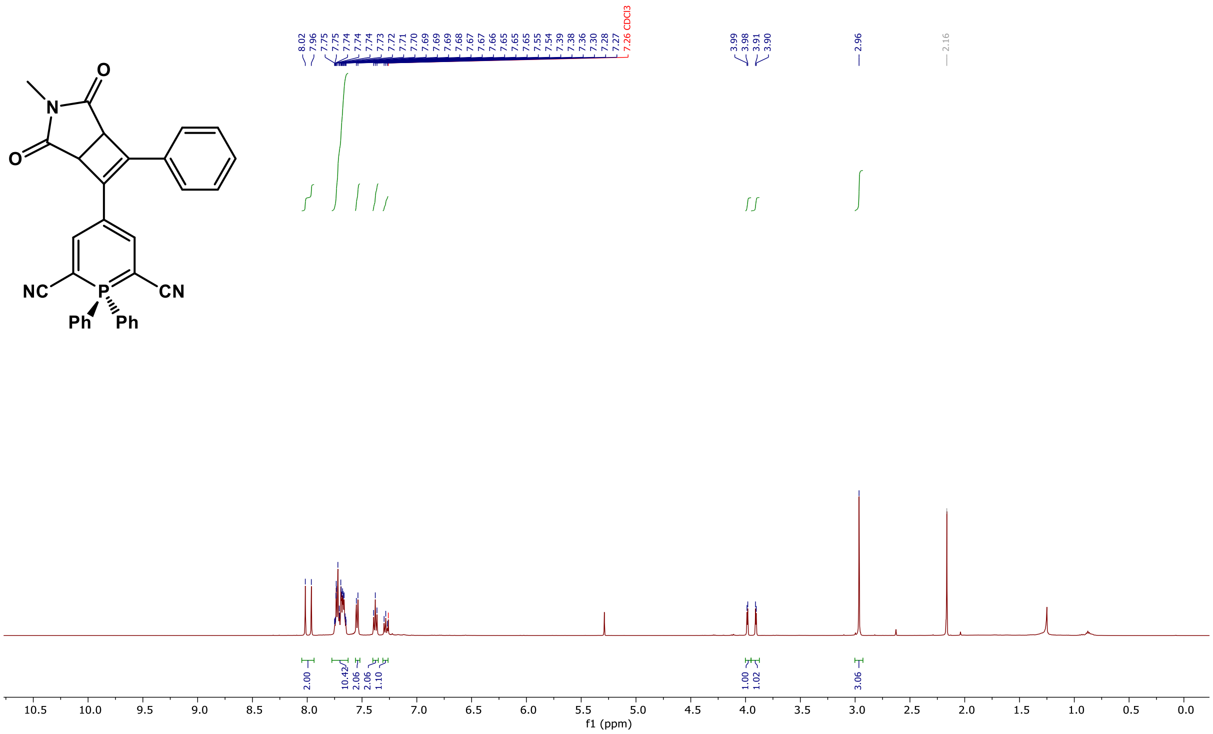

^{13}C NMR (126 MHz)

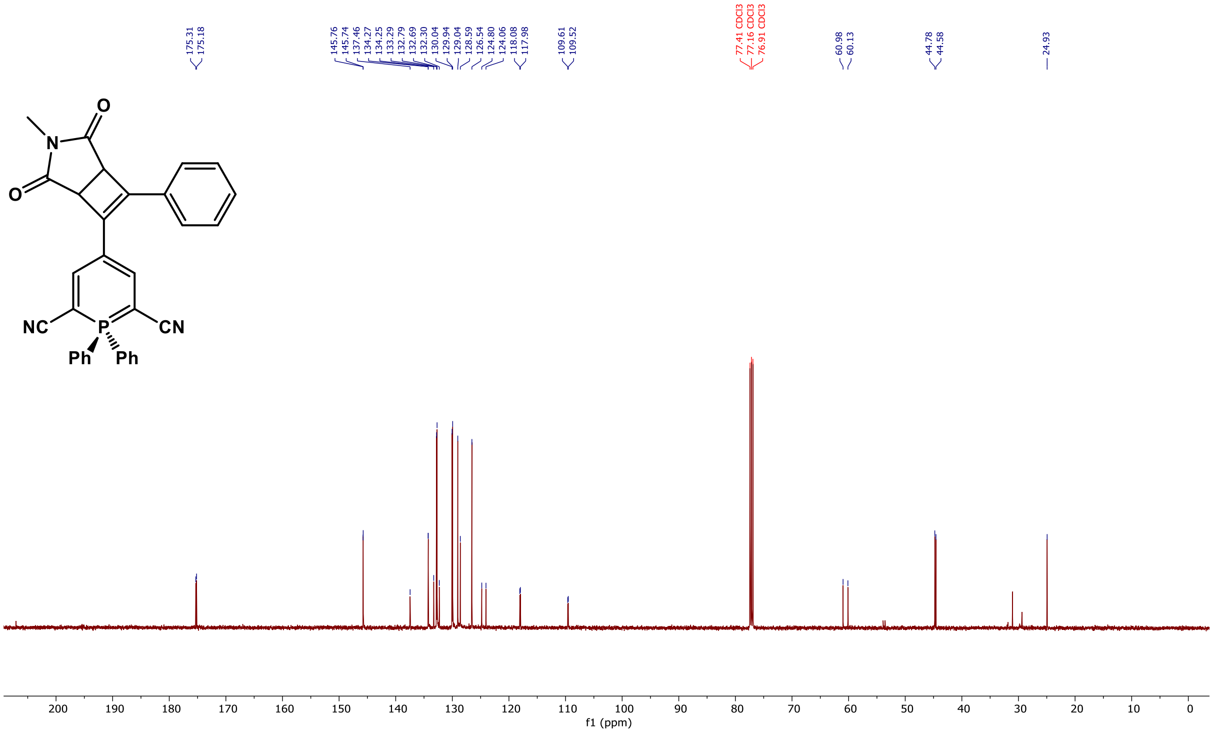

^{31}P NMR (202 MHz)

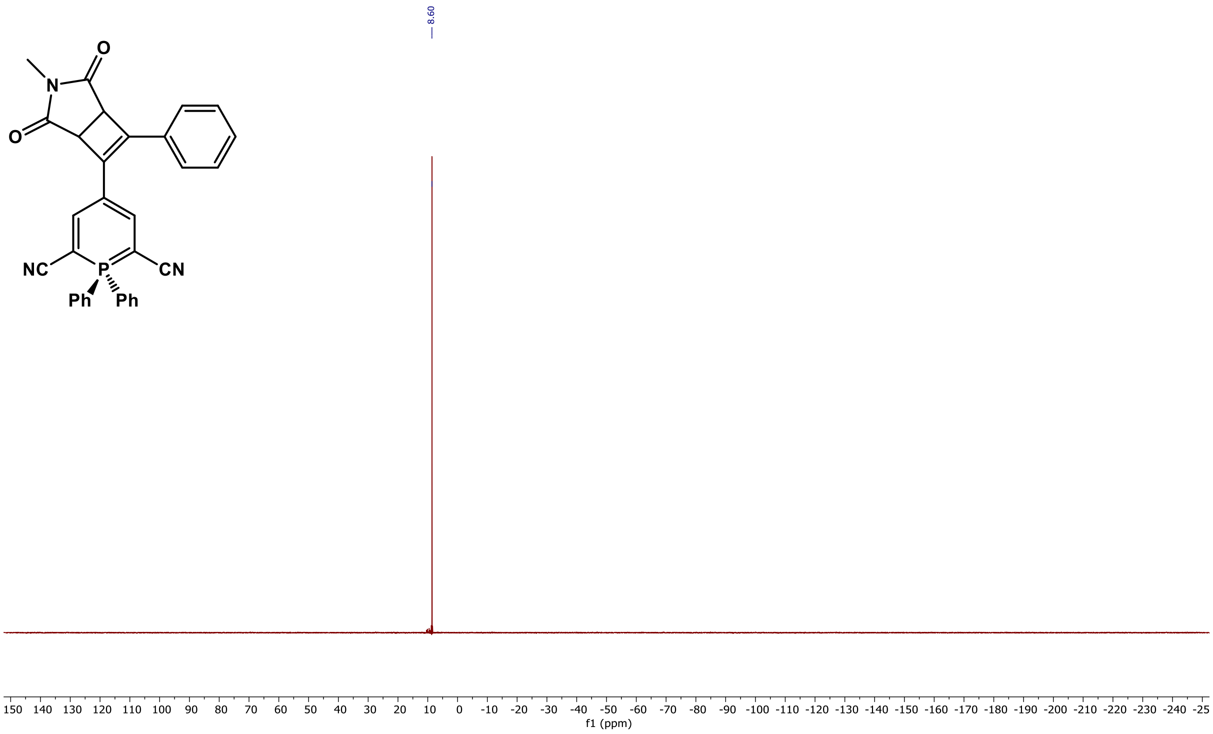

methyl (*E*)-3-(2,6-dicyano-1,1-diphenyl-1λ^5-phosphinin-4-yl)acrylate (3v)

^{1}H NMR (500 MHz)

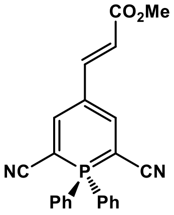

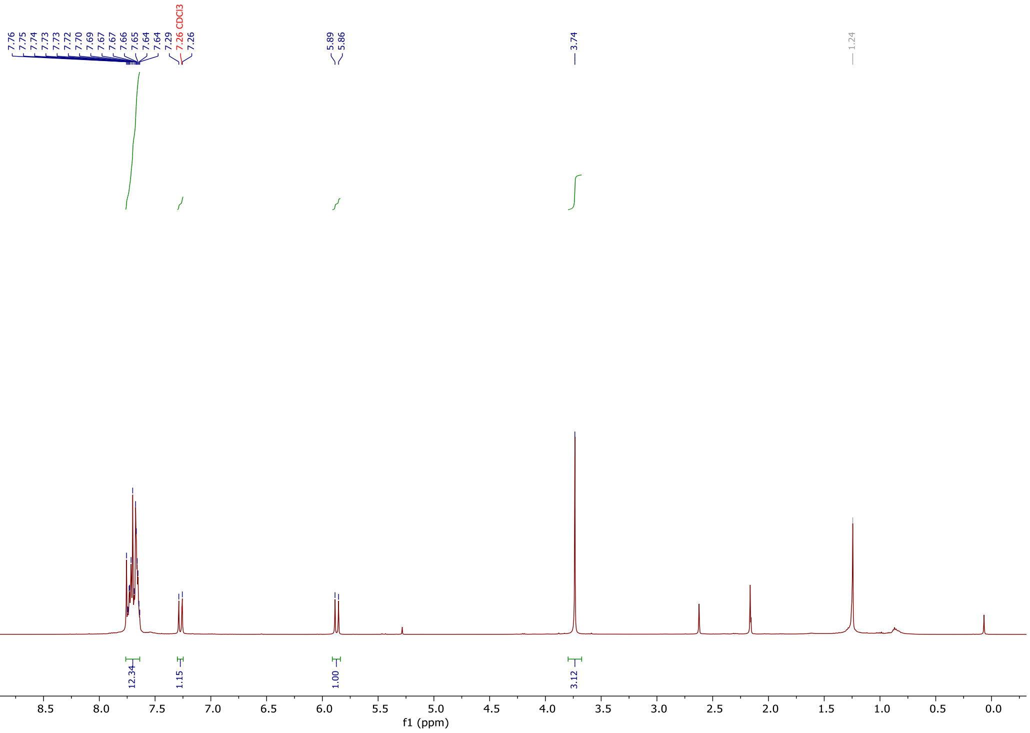

^{13}C NMR (126 MHz)

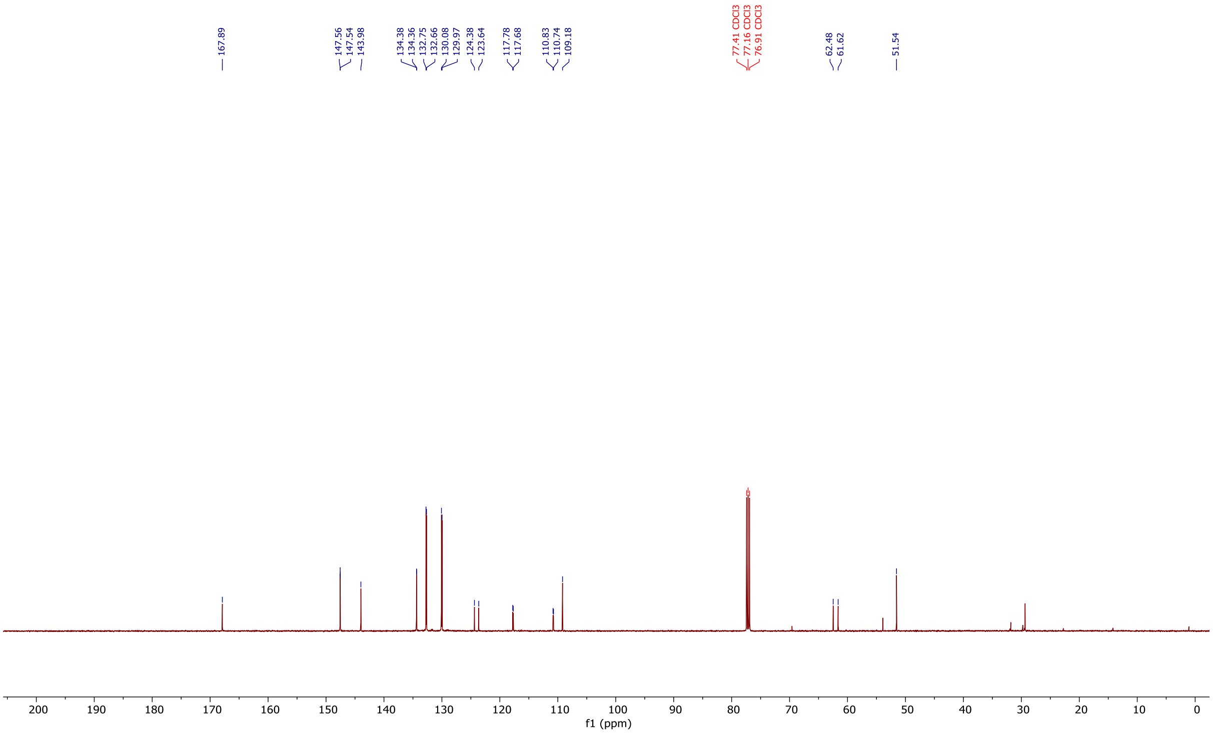

^{31}P NMR (202 MHz)

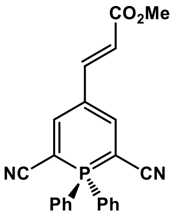

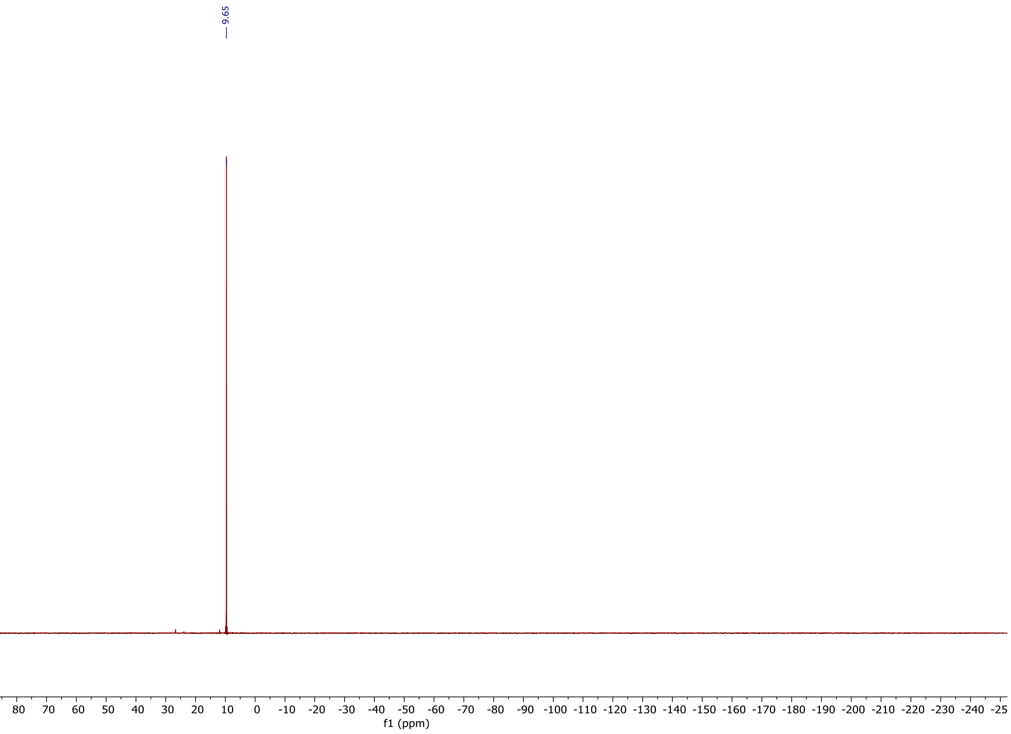

tert-butyl 4-(2,6-dicyano-1,1-diphenyl-1λ^5-phosphinin-4-yl)-3,6-dihydropyridine-1(2_H_)-carboxylate (3w)

^{1}H NMR (500 MHz)

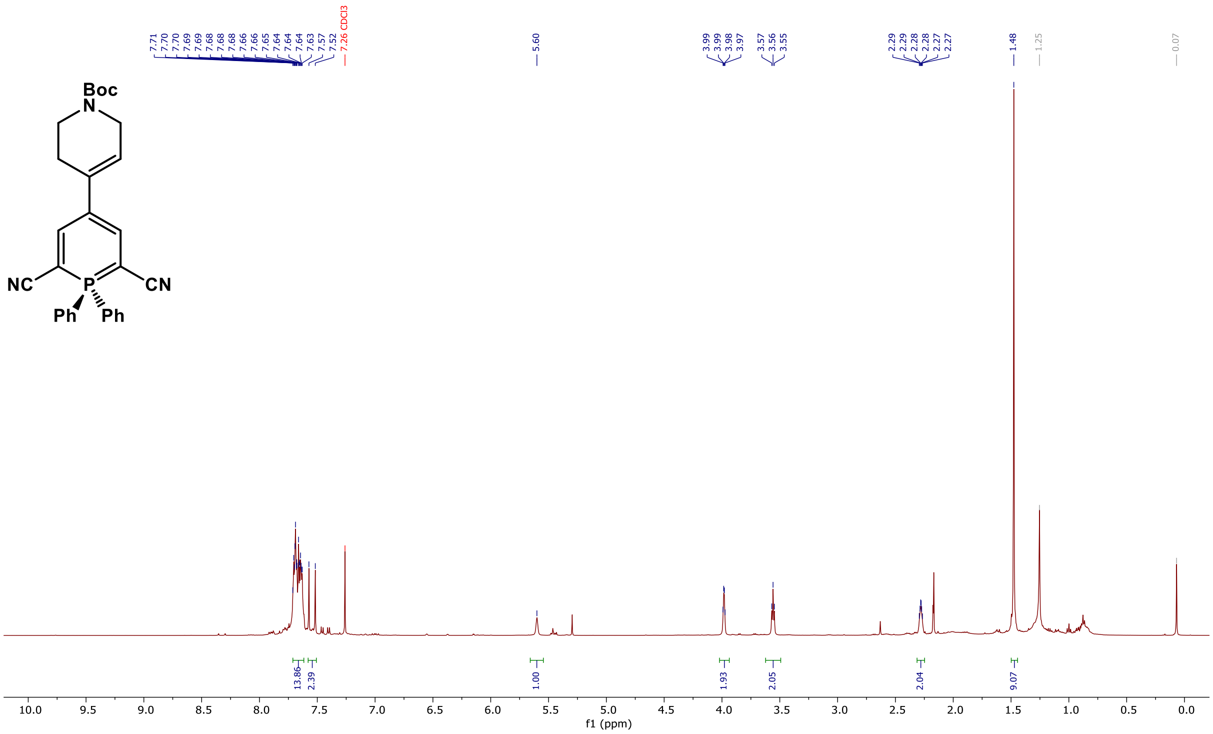

^{13}C NMR (126 MHz)

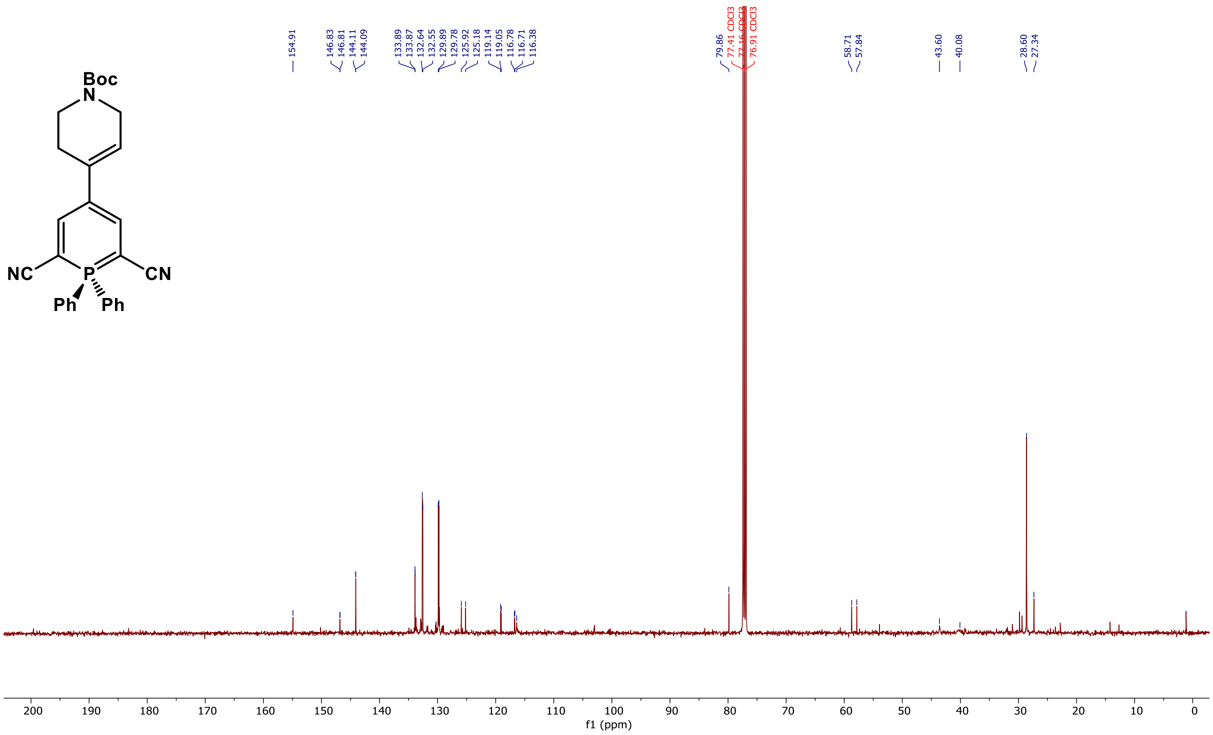

^{31}P NMR (202 MHz)

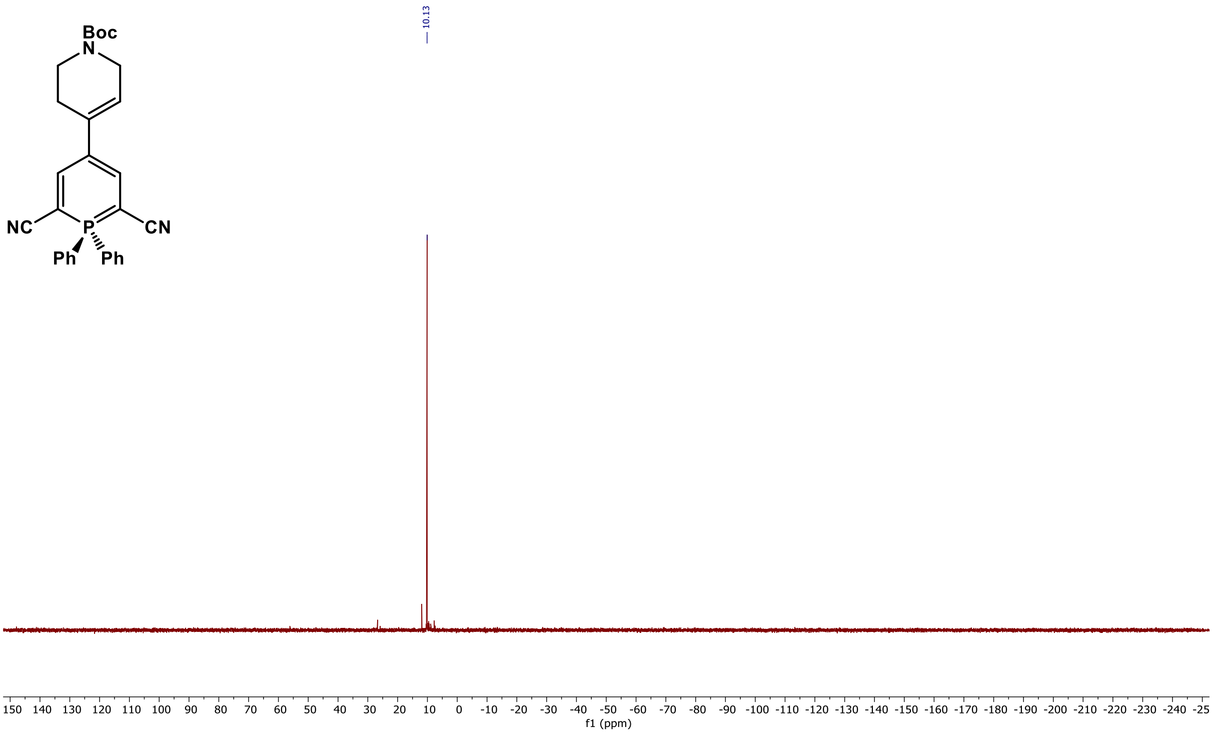

ethyl 6-(4-(2,6-dicyano-1,1-diphenyl-1λ[5]-phosphinin-4-yl)phenyl)-1-(4-methoxyphenyl)-7-oxo-4,5,6,7-tetrahydro-1*H*-pyrazolo[3,4-*c*]pyridine-3-carboxylate (3x)

^{1}H NMR (500 MHz)

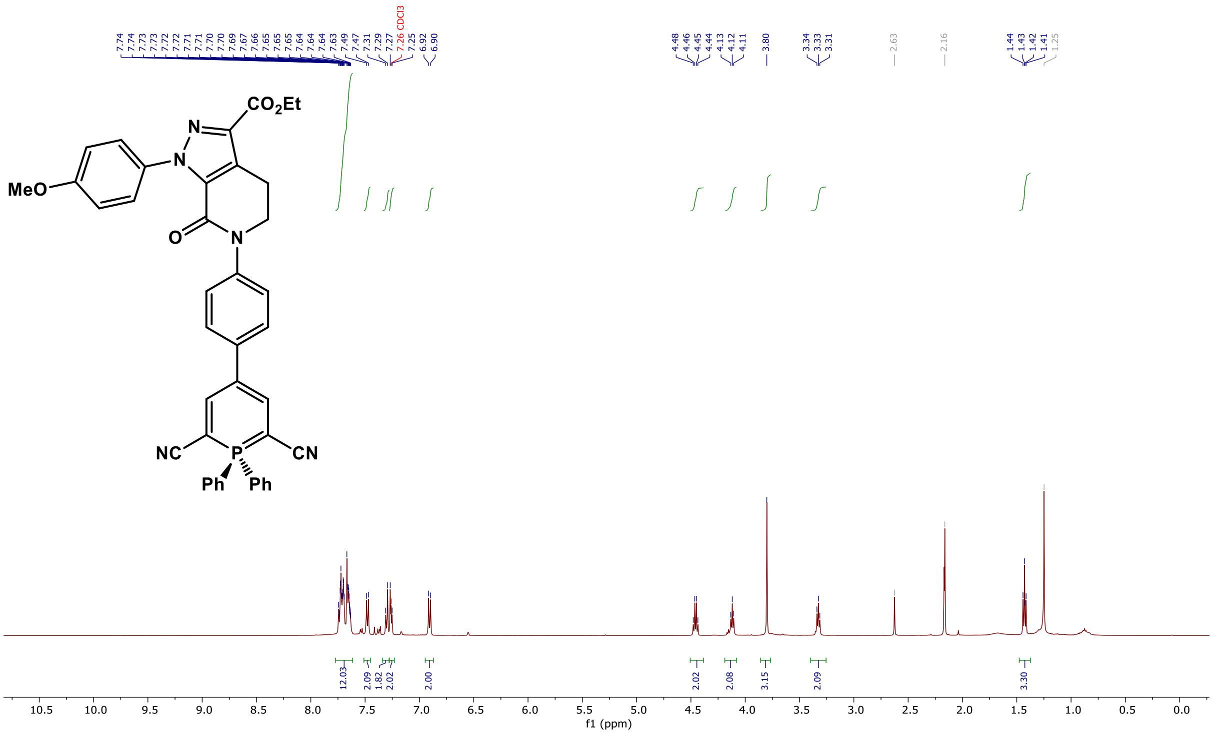

^{13}C NMR (126 MHz)

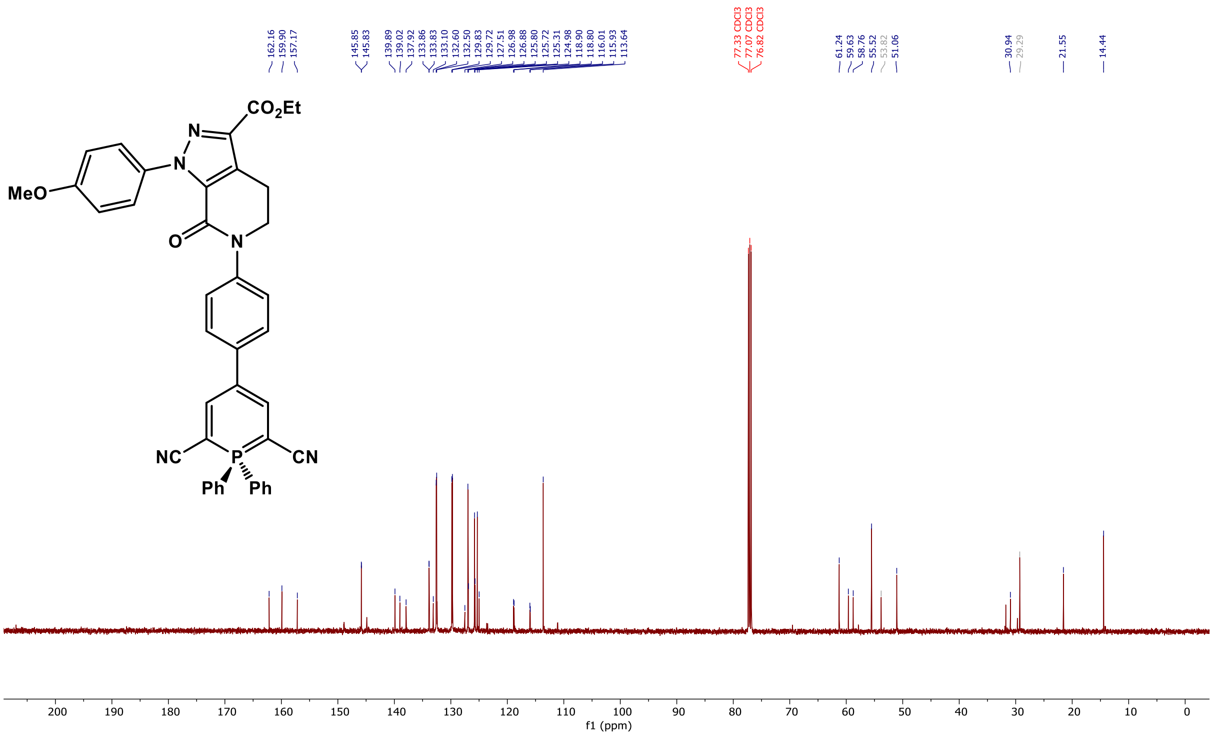

^{31}P NMR (202 MHz)

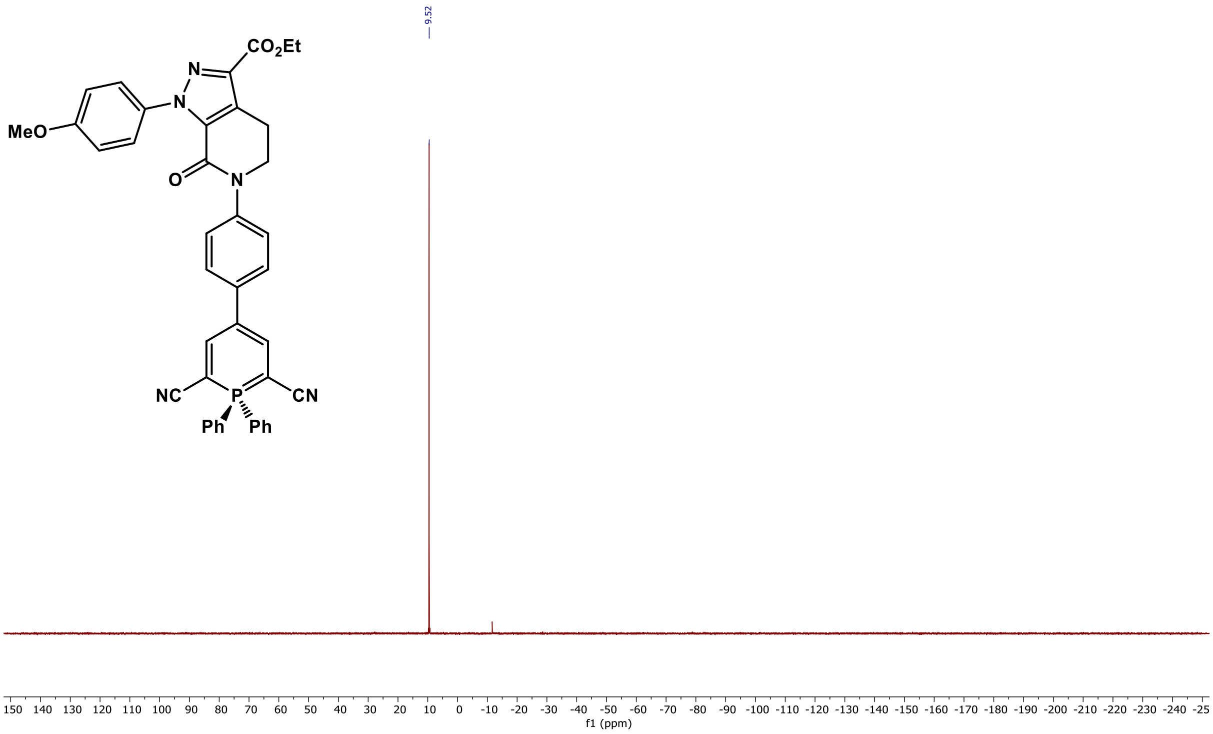

4-(1-(2-fluoro-6-(trifluoromethyl)benzyl)-3,6-dimethyl-2,4-dioxo-1,2,3,4-tetrahydropyrimidin-5-yl)-1,1-diphenyl-1λ^5-phosphinine-2,6-dicarbonitrile (3y)

^{1}H NMR (500 MHz)

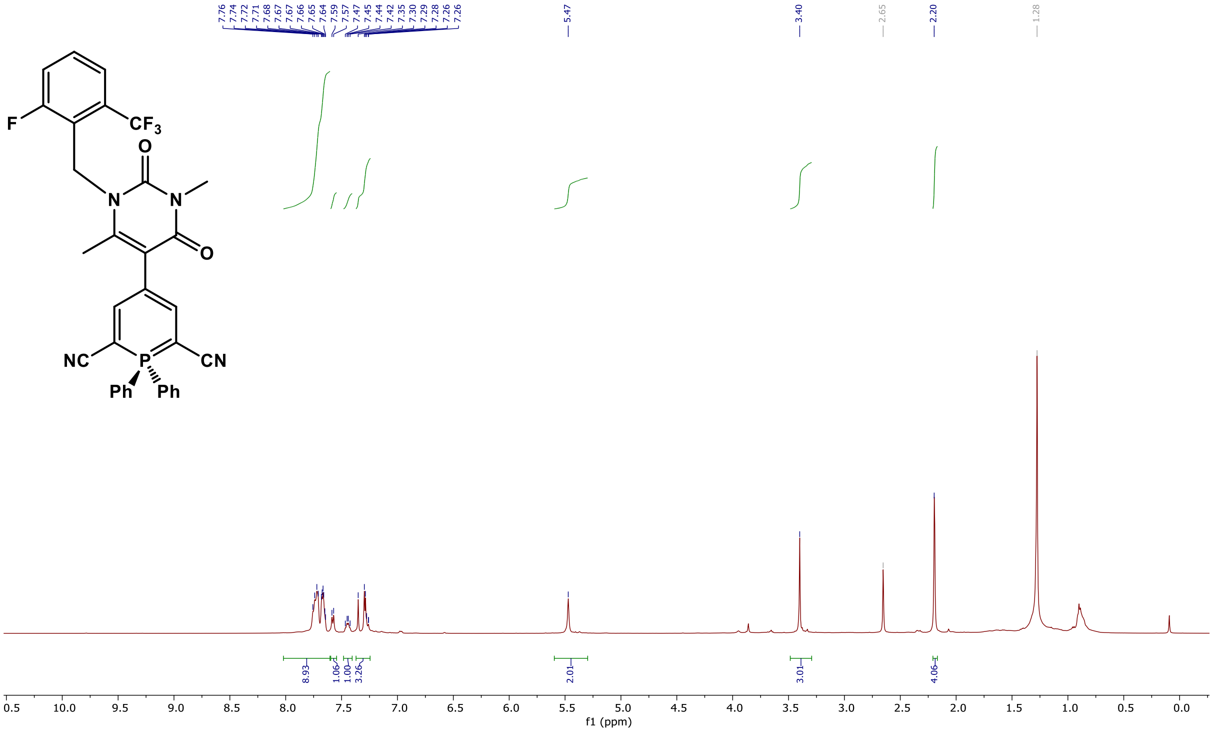

^{13}C NMR (126 MHz)

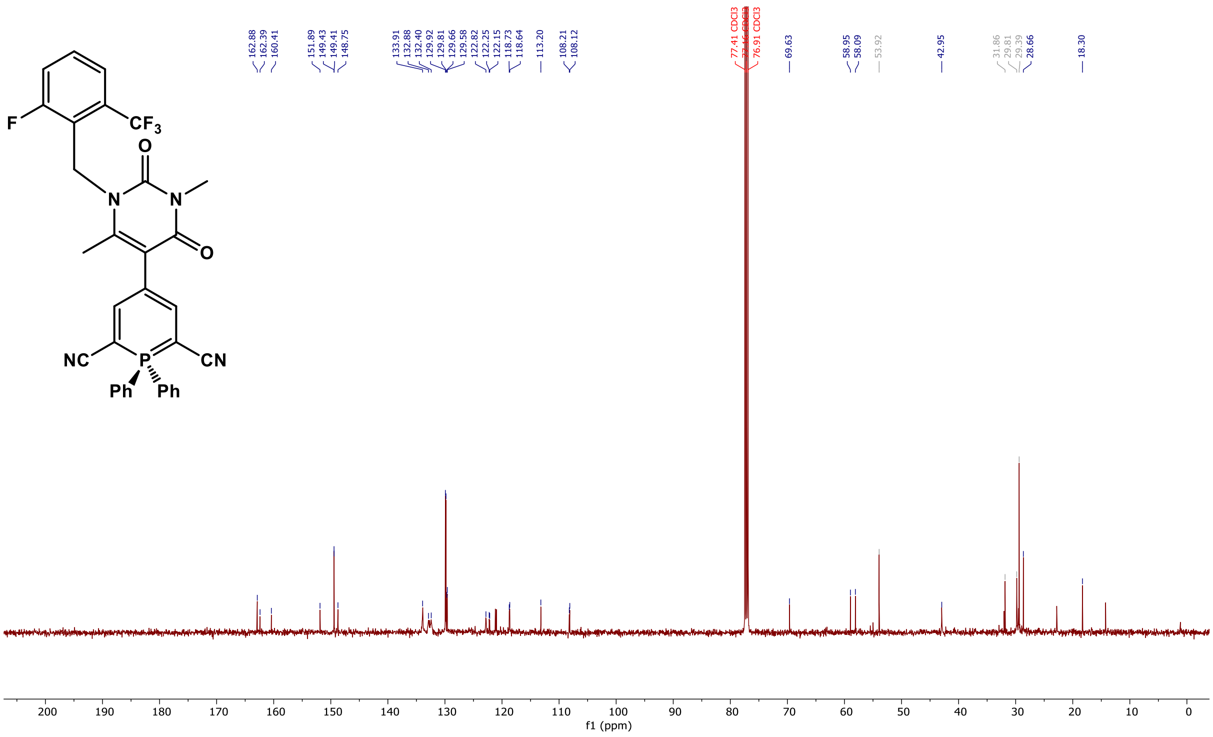

^{31}P NMR (202 MHz)

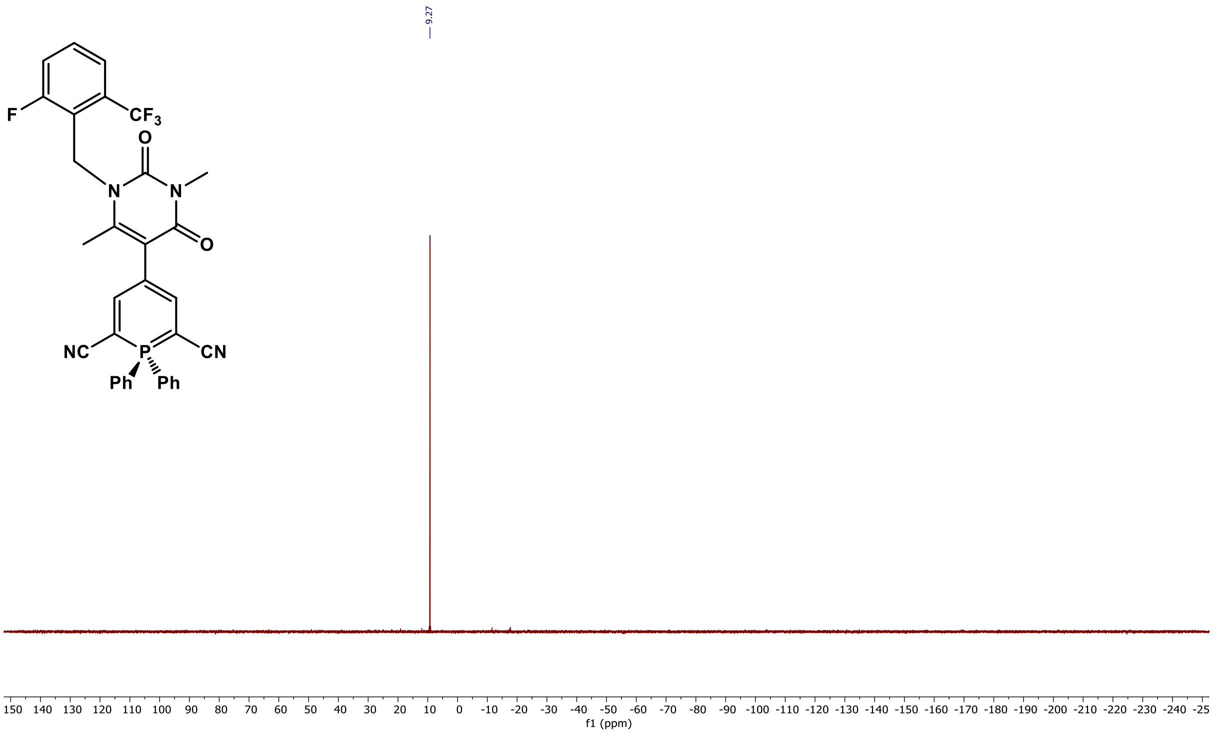

^{19}F NMR (471 MHz)

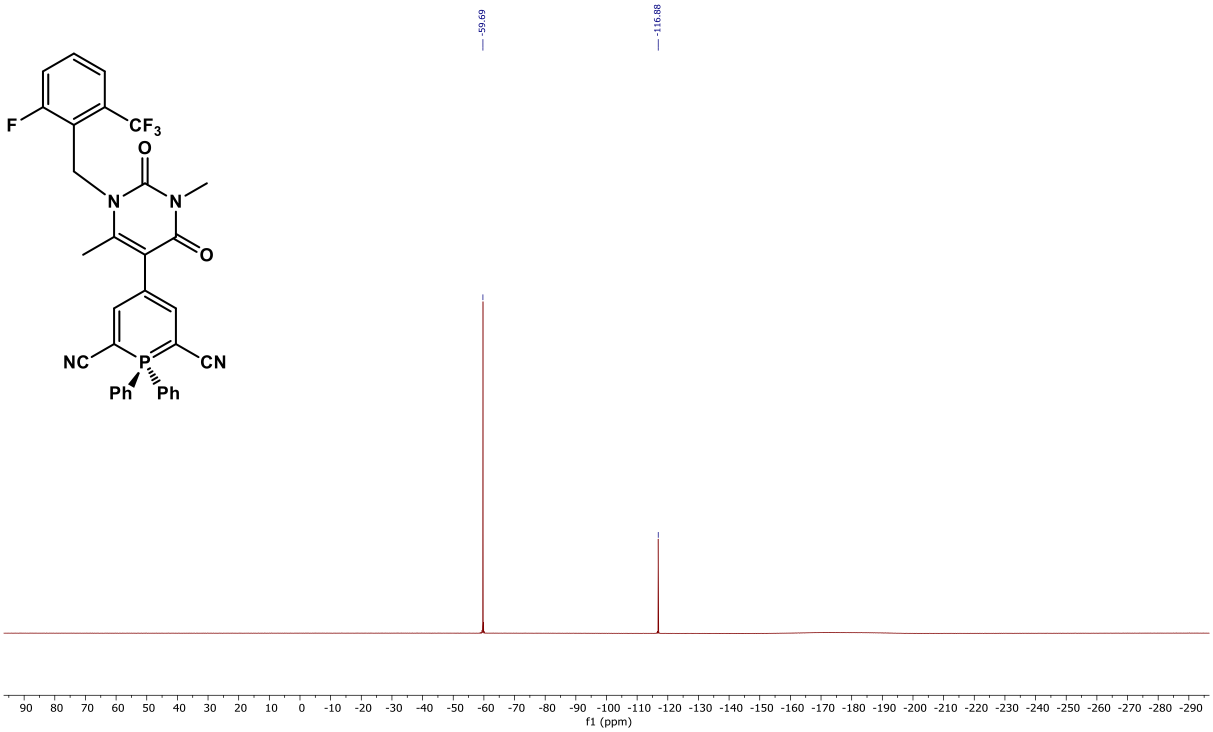

4-(1-methyl-2,6-dioxo-3-((2*R*,4*S*,5*R*)-4-((triisopropylsilyl)oxy)-5-(((triisopropylsilyl)oxy)methyl)tetrahydrofuran-2-yl)-1,2,3,6-tetrahydropyrimidin-4-yl)-1,1-diphenyl-1λ^5-phosphinine-2,6-dicarbonitrile (3z)

^{1}H NMR (500 MHz)

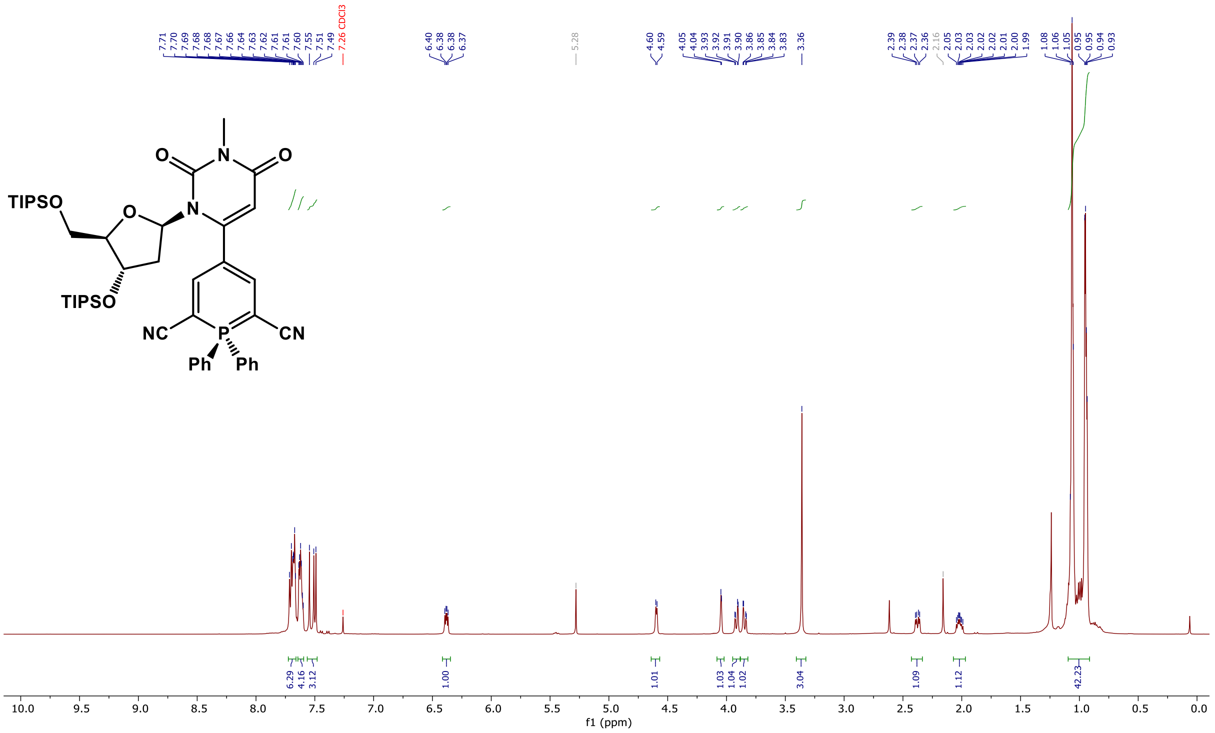

^{13}C NMR (126 MHz)

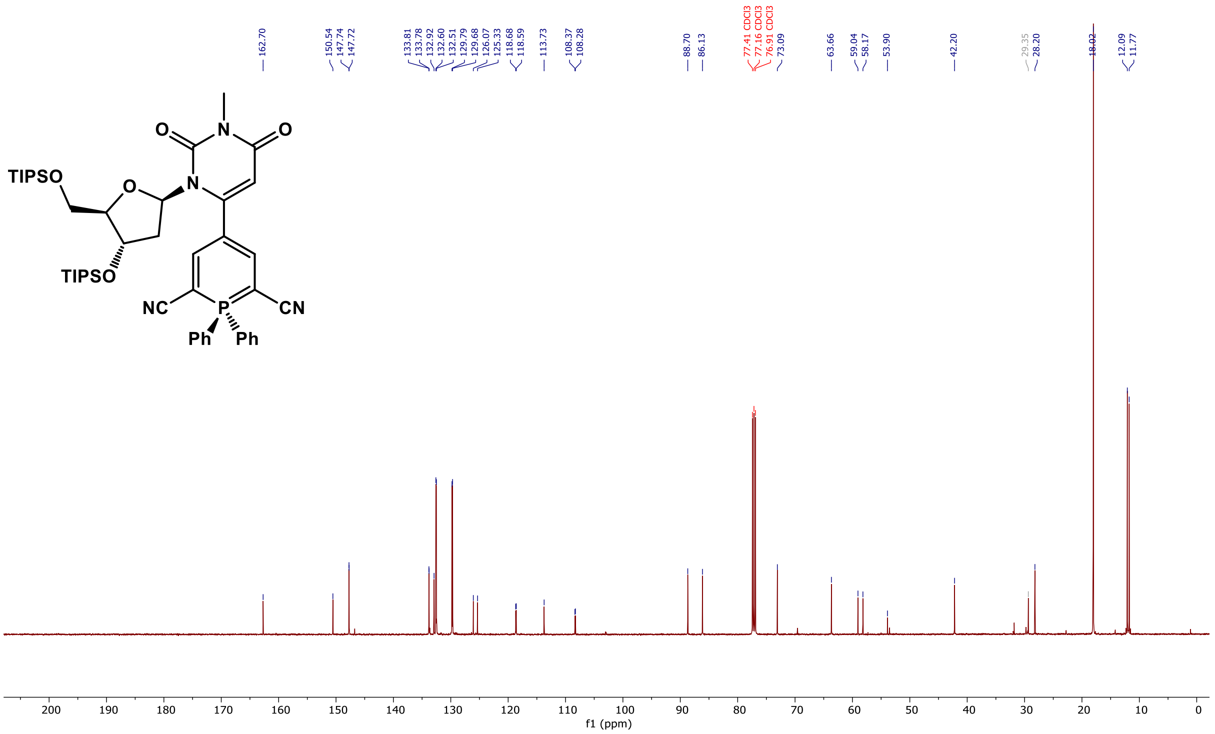

^{31}P NMR (202 MHz)

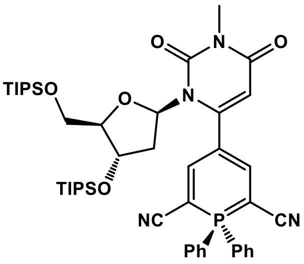

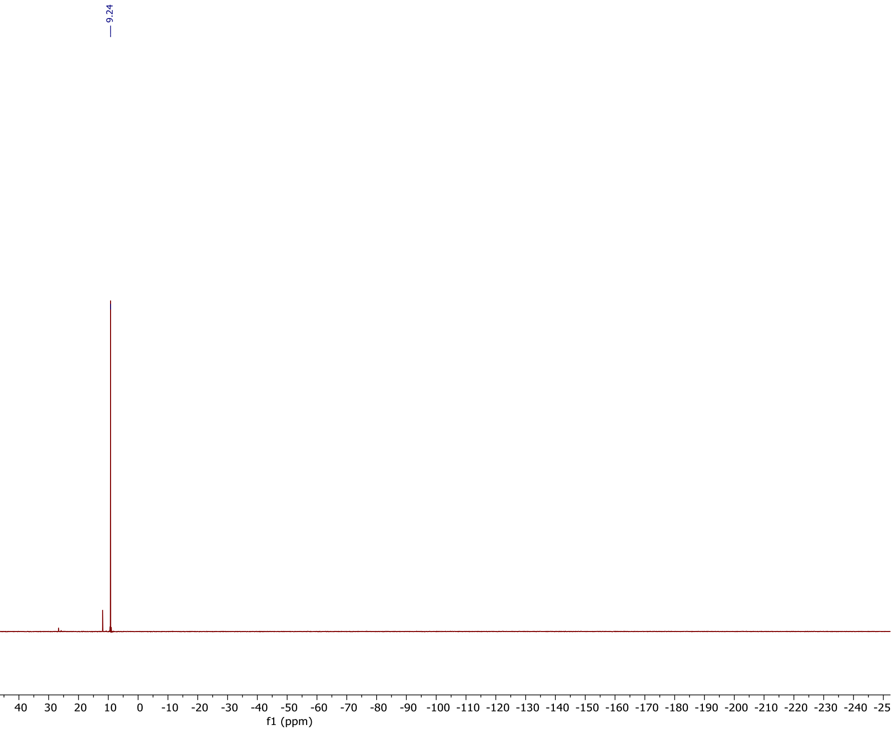

4-((2*R*,3*R*,4*R*)-3,4-bis(benzyloxy)-2-((benzyloxy)methyl)-3,4-dihydro-2*H*-pyran-5-yl)-1,1-diphenyl-1λ[5]-phosphinine-2,6-dicarbonitrile (3aa)

^{1}H NMR (500 MHz)

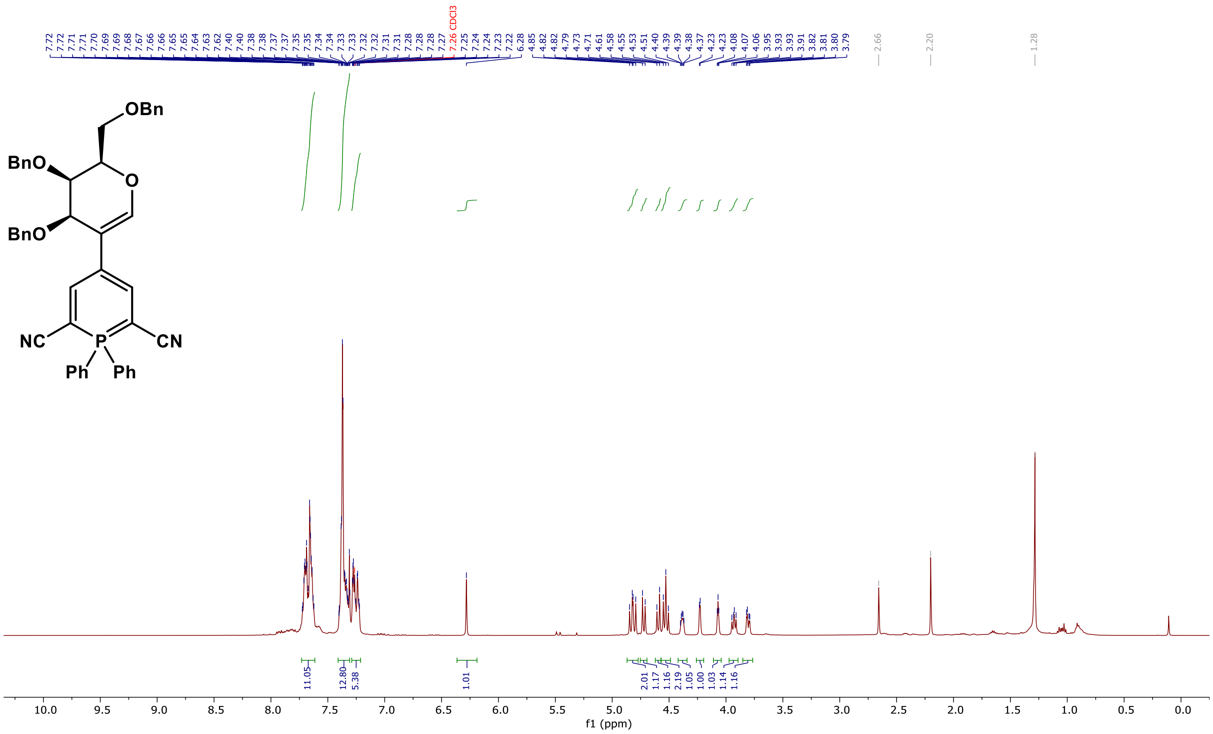

^{13}C NMR (126 MHz)

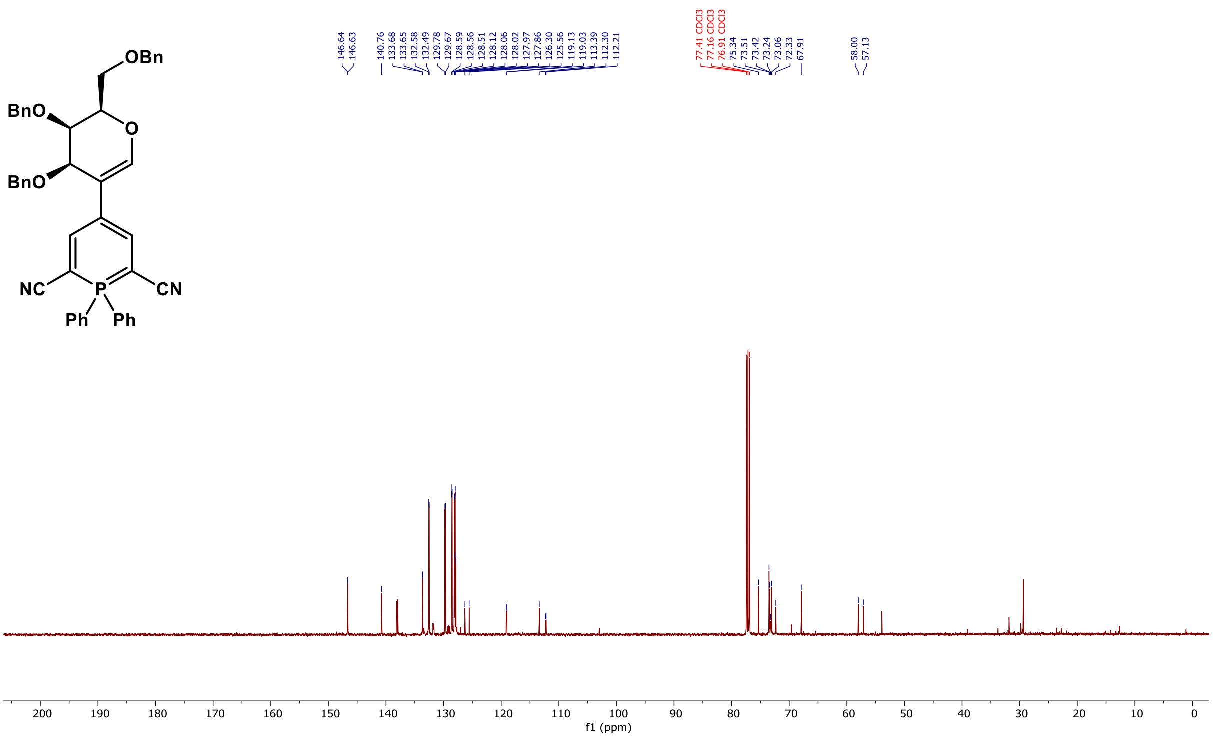

^{31}P NMR (202 MHz)

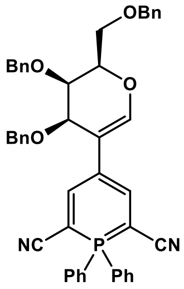

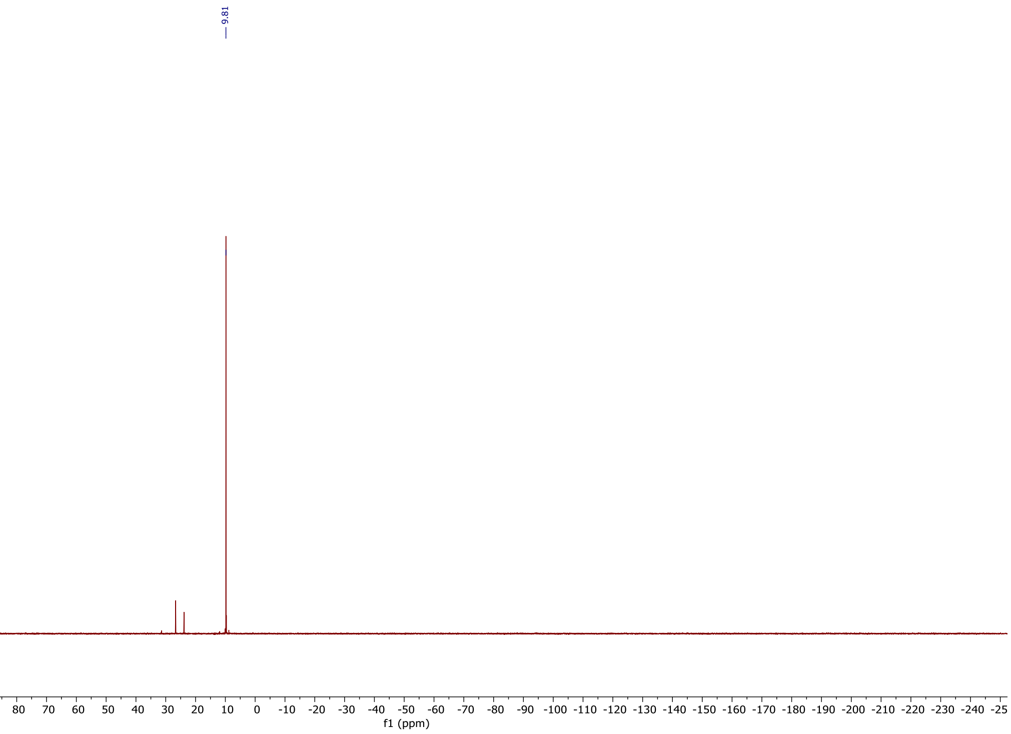

diethyl 1,1-diphenyl-4-(3,4,5-trimethoxyphenyl)-1λ^5-phosphinine-2,6-dicarboxylate (5d)

^{1}H NMR (500 MHz)

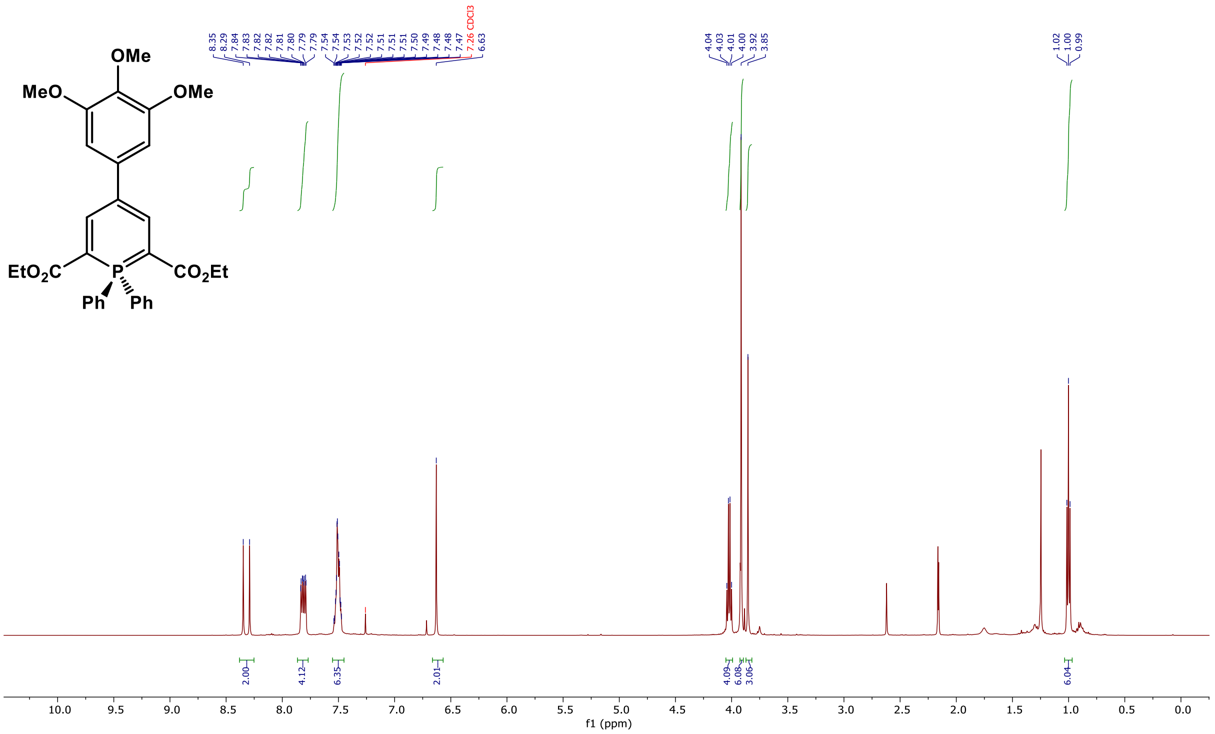

^{13}C NMR (126 MHz)

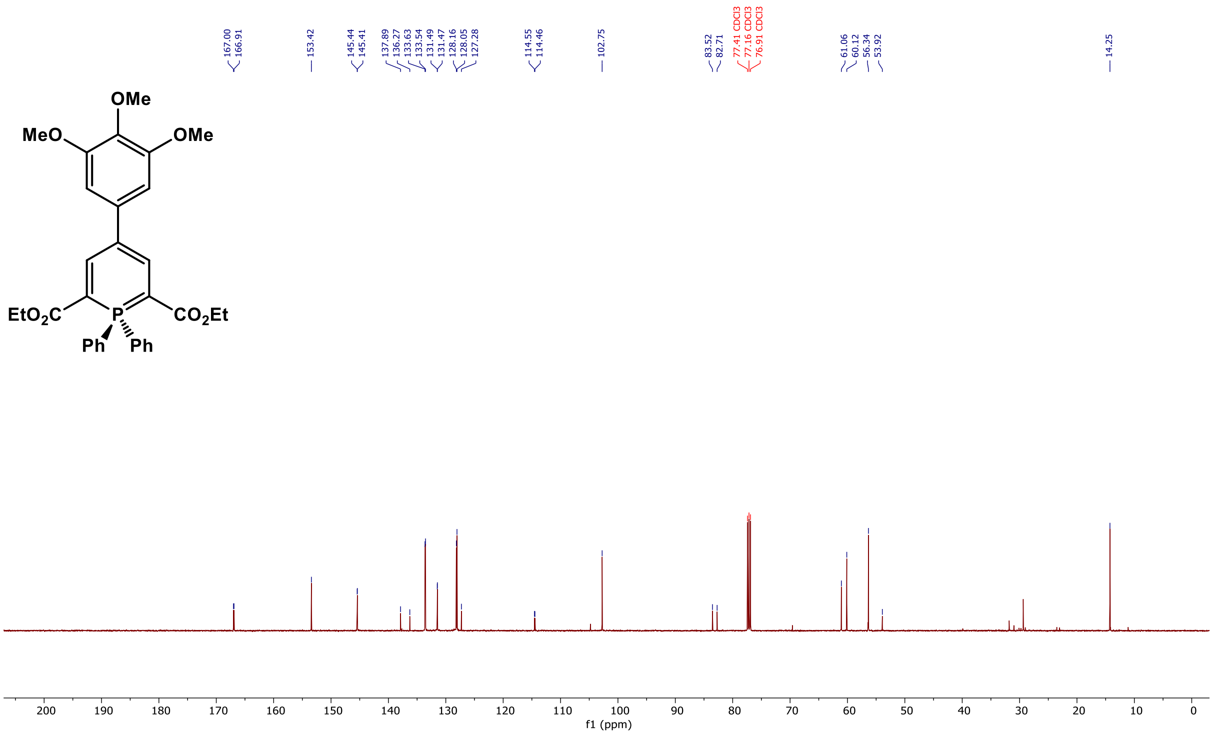

^{31}P NMR (202 MHz)

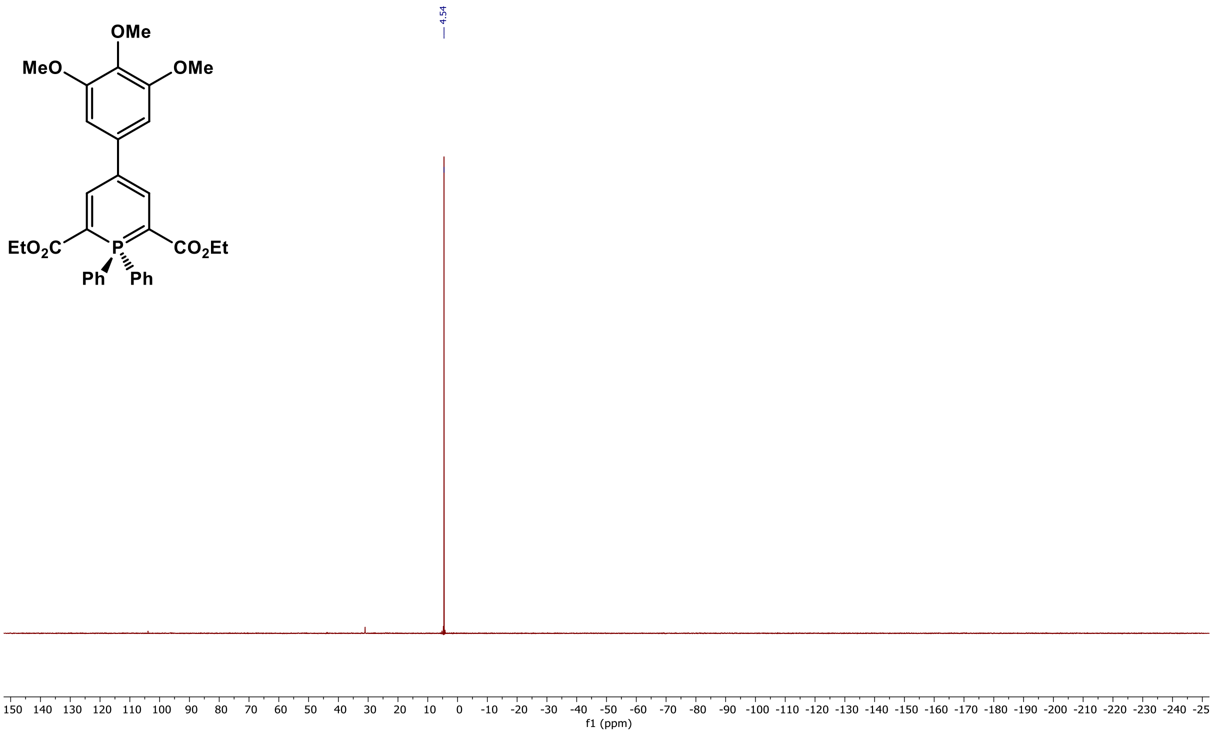

diethyl 4-(2,3-dihydrobenzo[*b*][1,4]dioxin-6-yl)-1,1-diphenyl-1λ^5-phosphinine-2,6-dicarboxylate (5e)

^{1}H NMR (500 MHz)

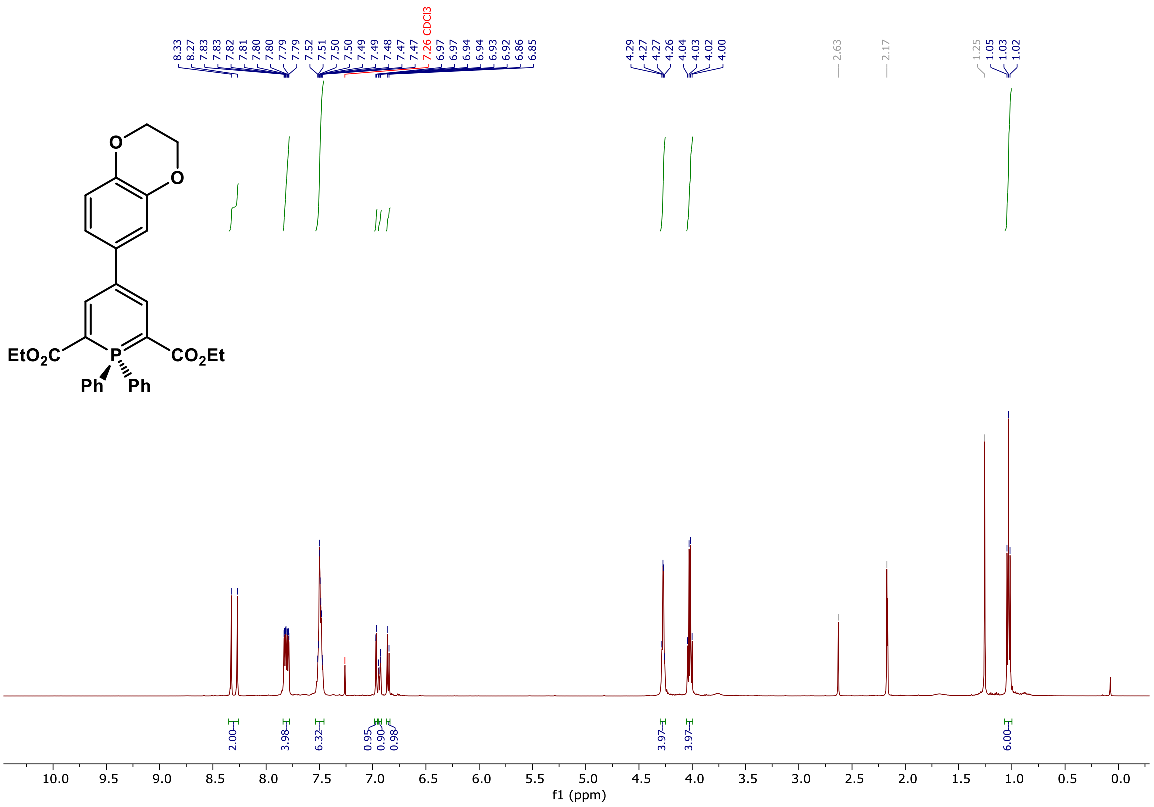

^{13}C NMR (126 MHz)

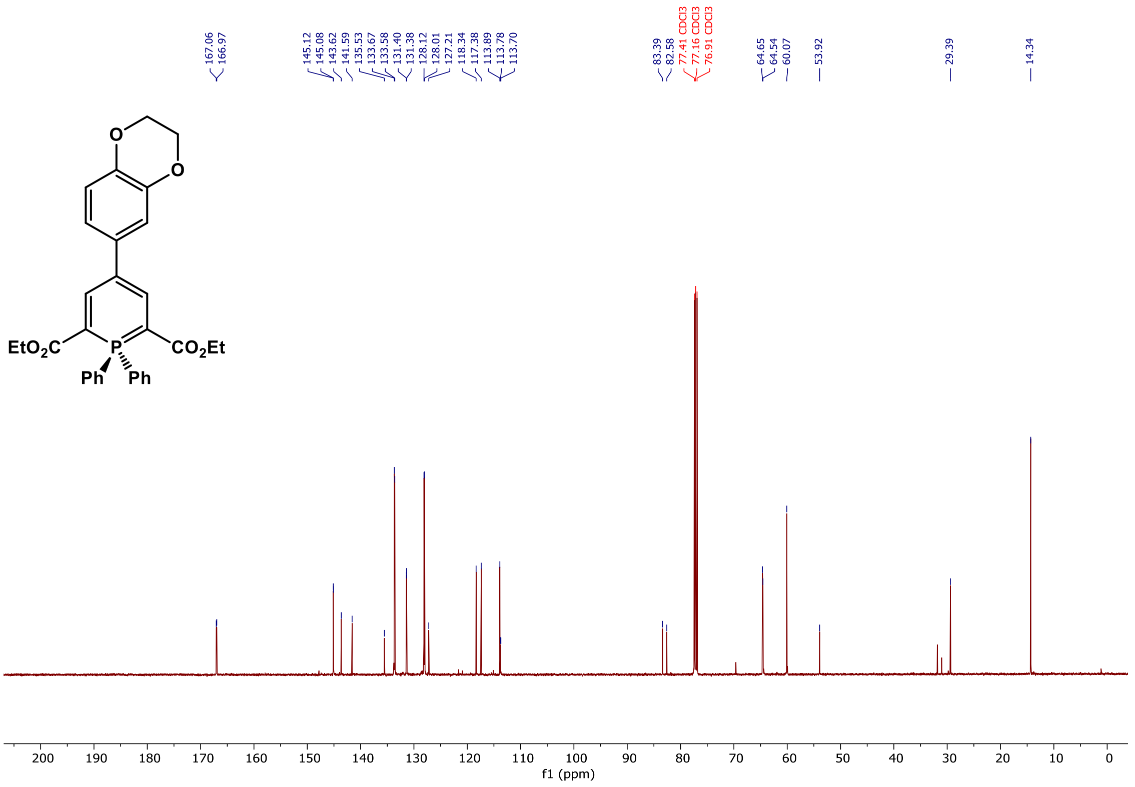

^{31}P NMR (202 MHz)

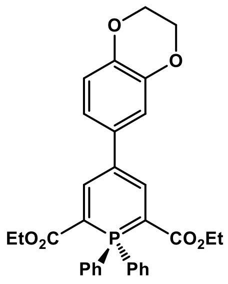

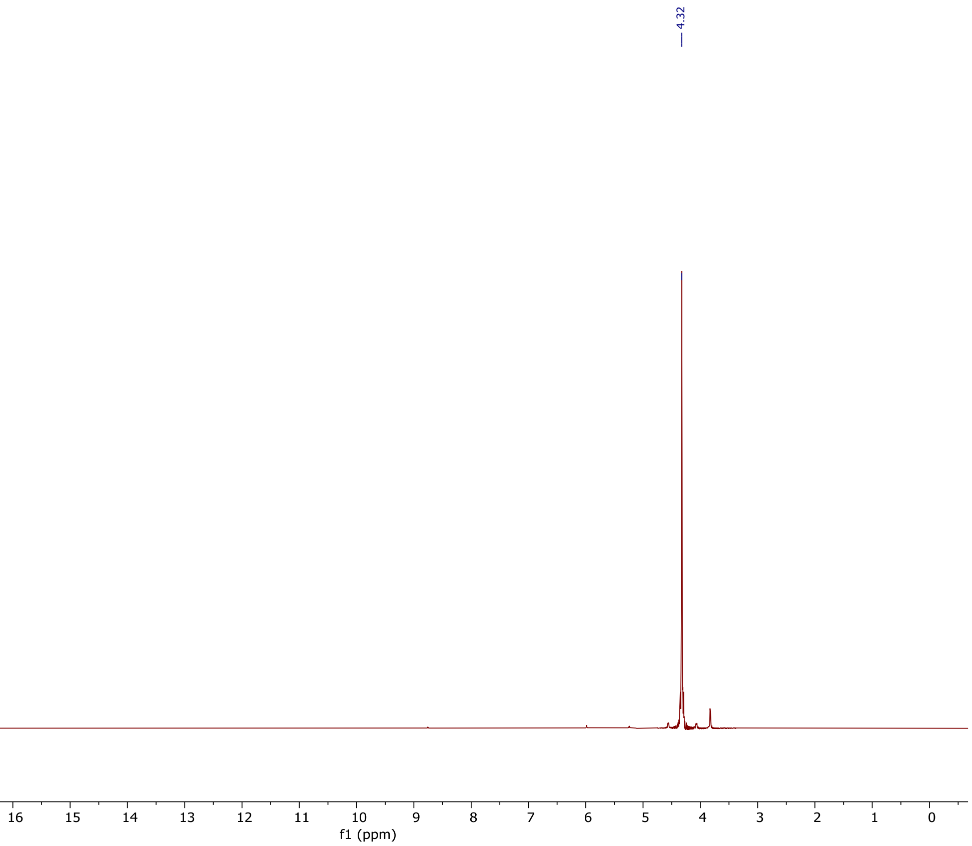

diethyl 1,1-diphenyl-4-(3-(trifluoromethoxy)phenyl)-1λ[5]-phosphinine-2,6-dicarboxylate (5f)

^{1}H NMR (500 MHz)

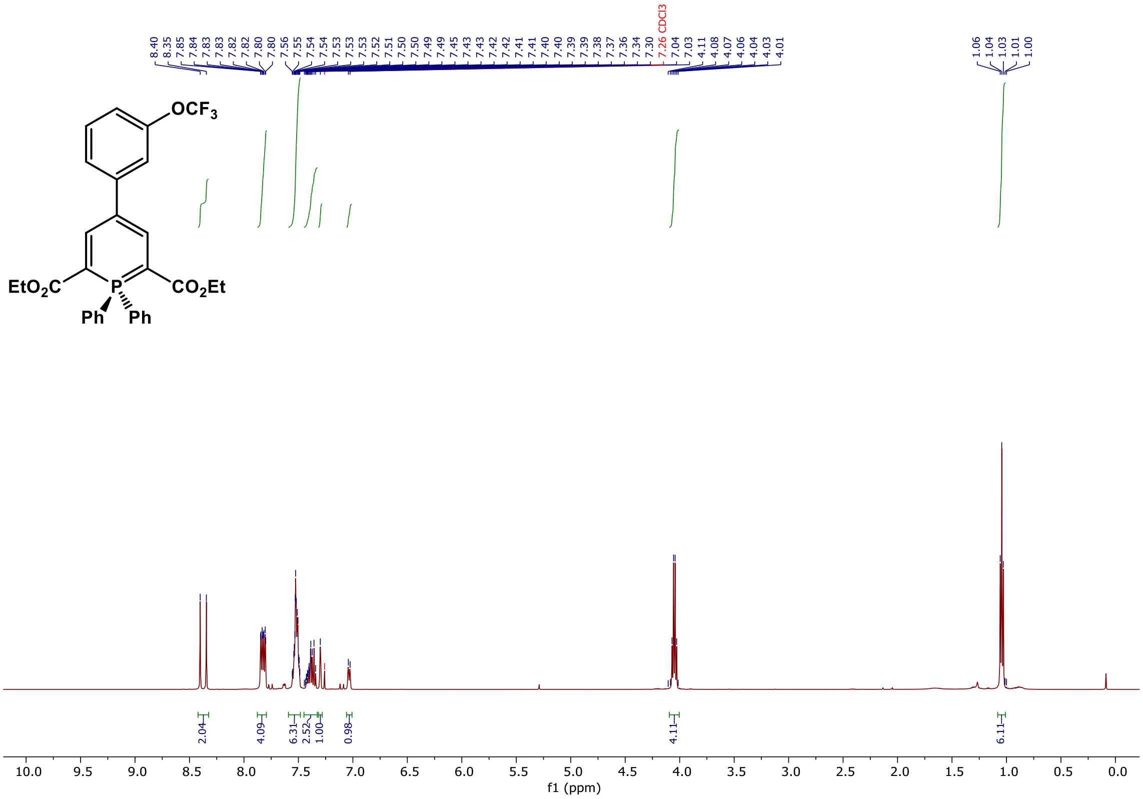

^{13}C NMR (126 MHz)

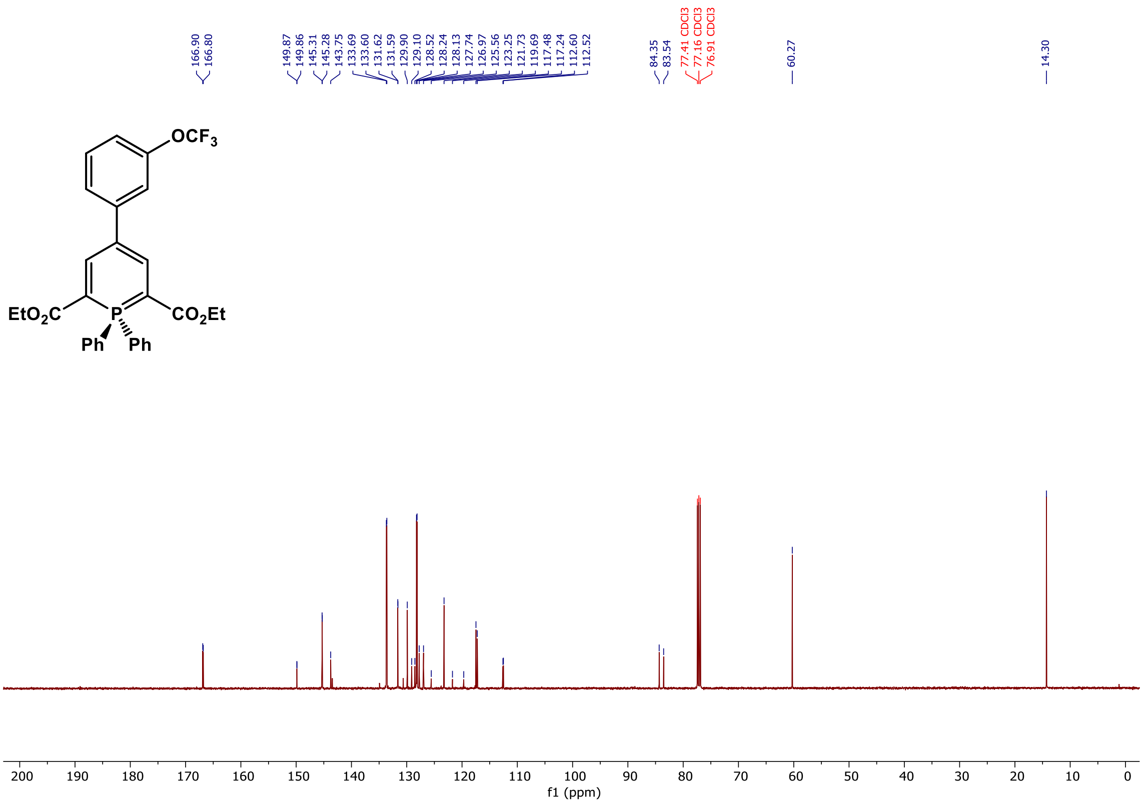

^{31}P NMR (202 MHz)

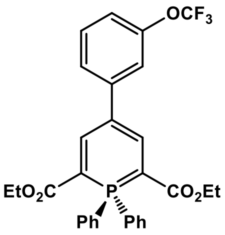

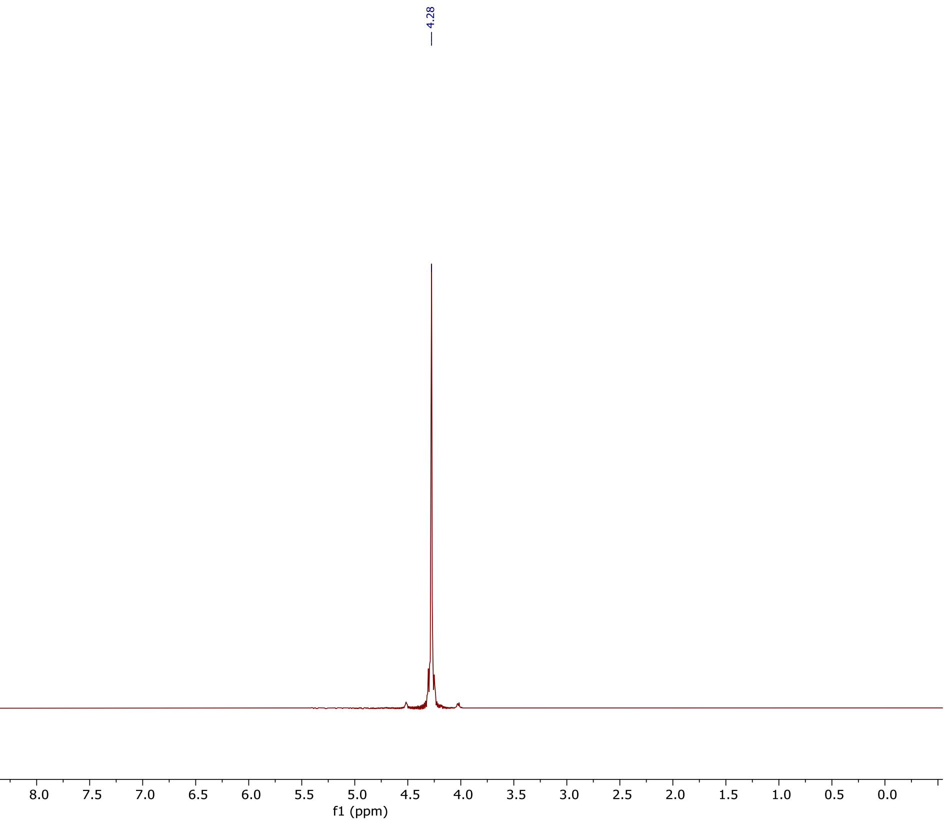

^{19}F NMR (471 MHz)

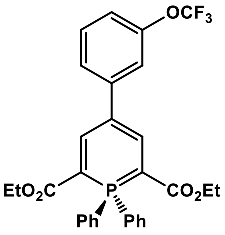

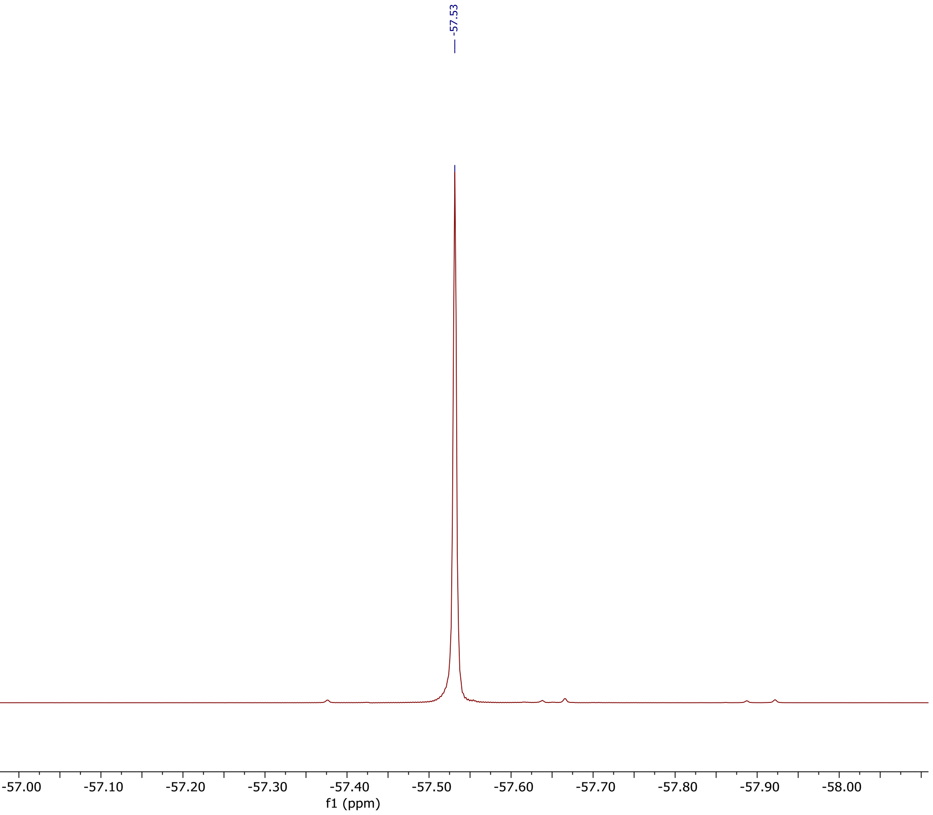

diethyl 4-(4-(difluoromethoxy)phenyl)-1,1-diphenyl-1λ^5-phosphinine-2,6-dicarboxylate (5g)

^{1}H NMR (500 MHz)

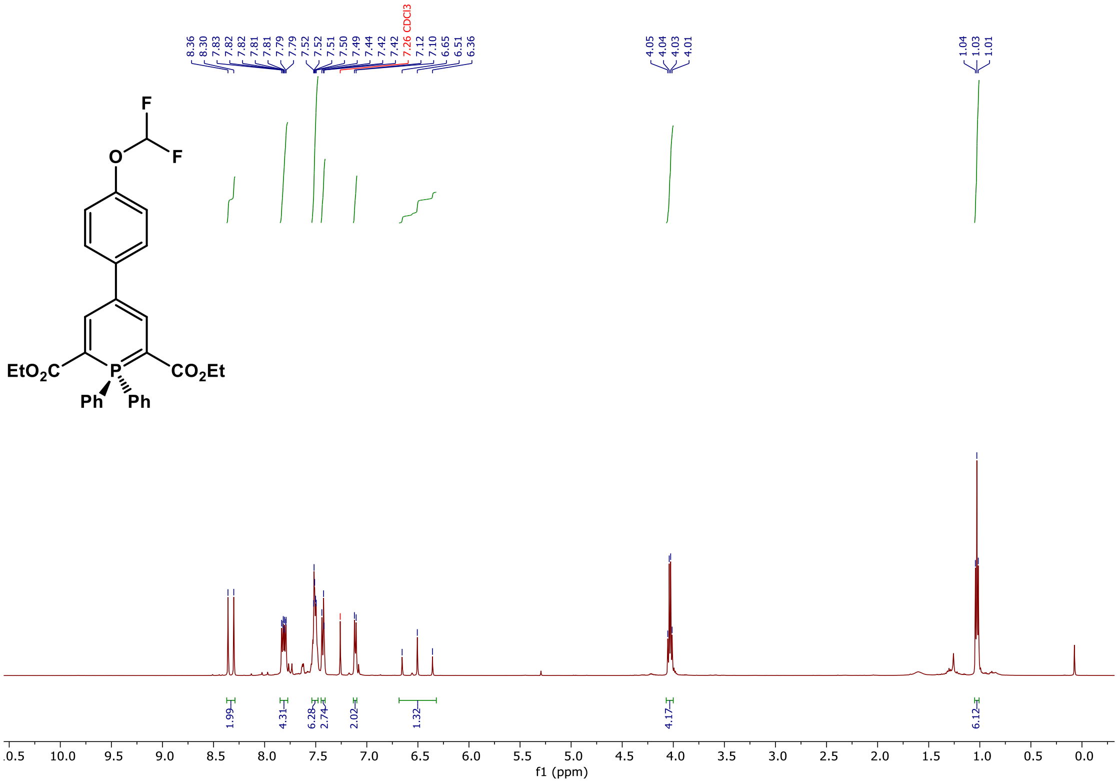

^{13}C NMR (126 MHz)

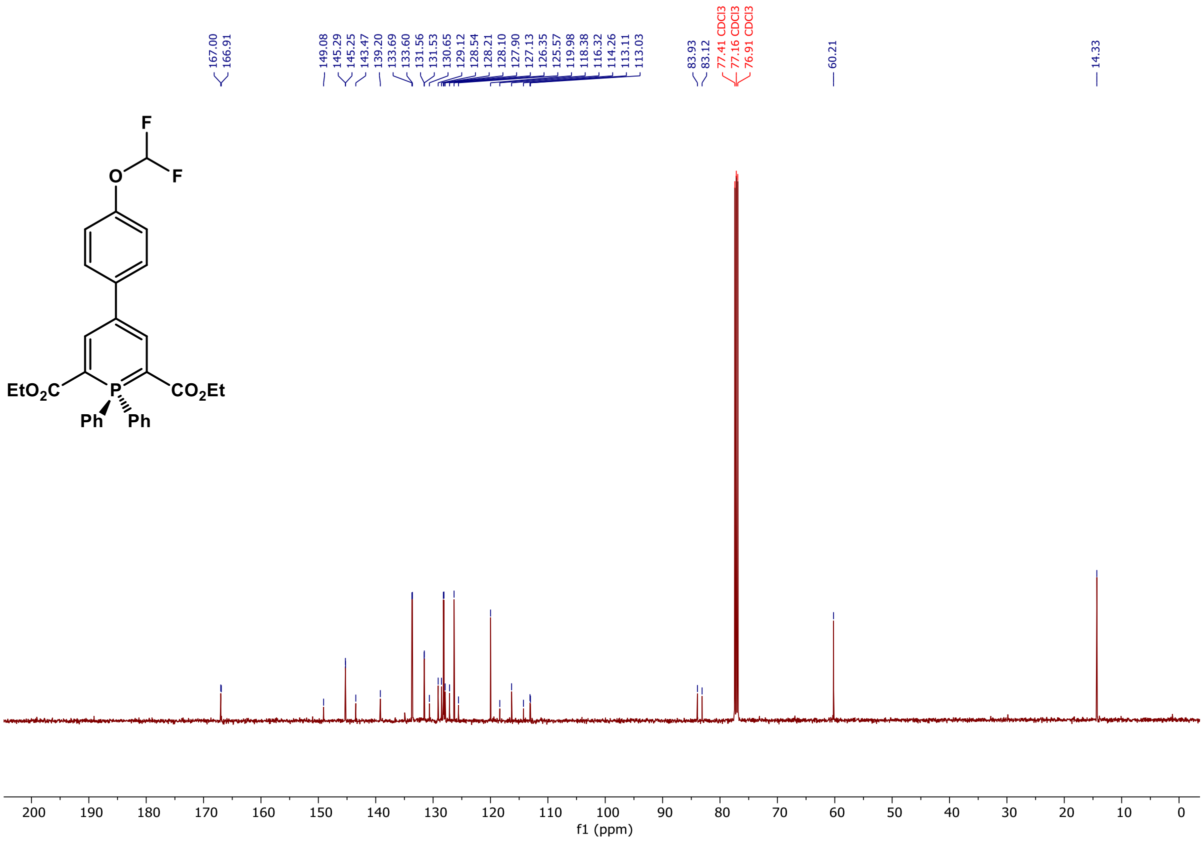

^{31}P NMR (202 MHz)

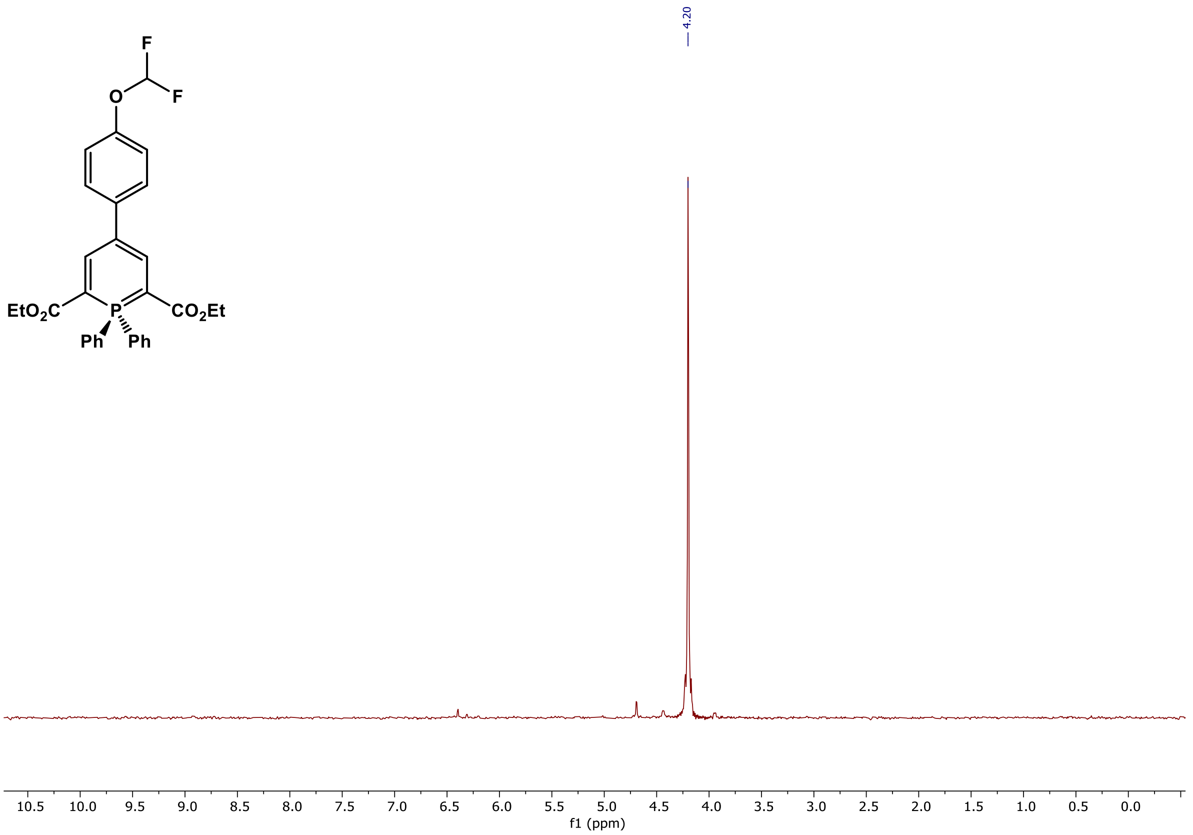

^{19}F NMR (471 MHz)

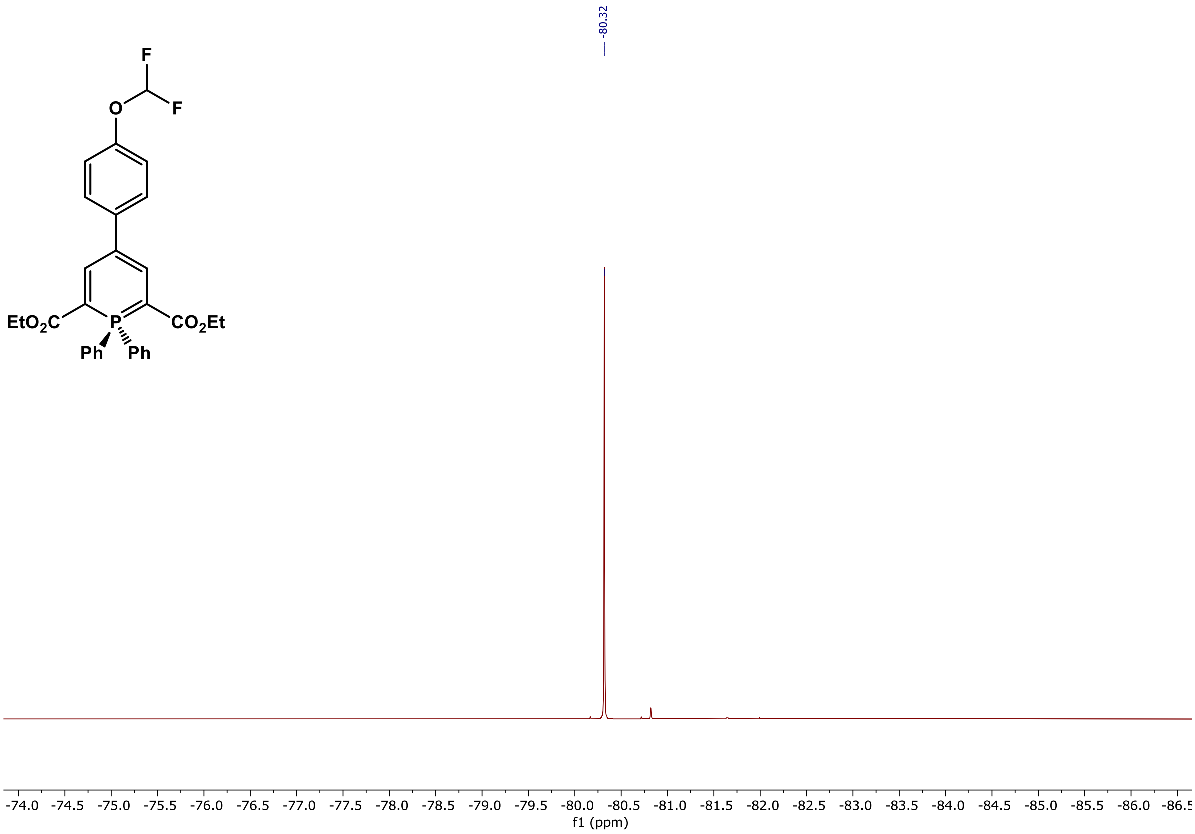

diethyl 1,1-diphenyl-4-(9-phenyl-9H-carbazol-3-yl)-1λ[5]-phosphinine-2,6-dicarboxylate (5h)

^{1}H NMR (500 MHz)

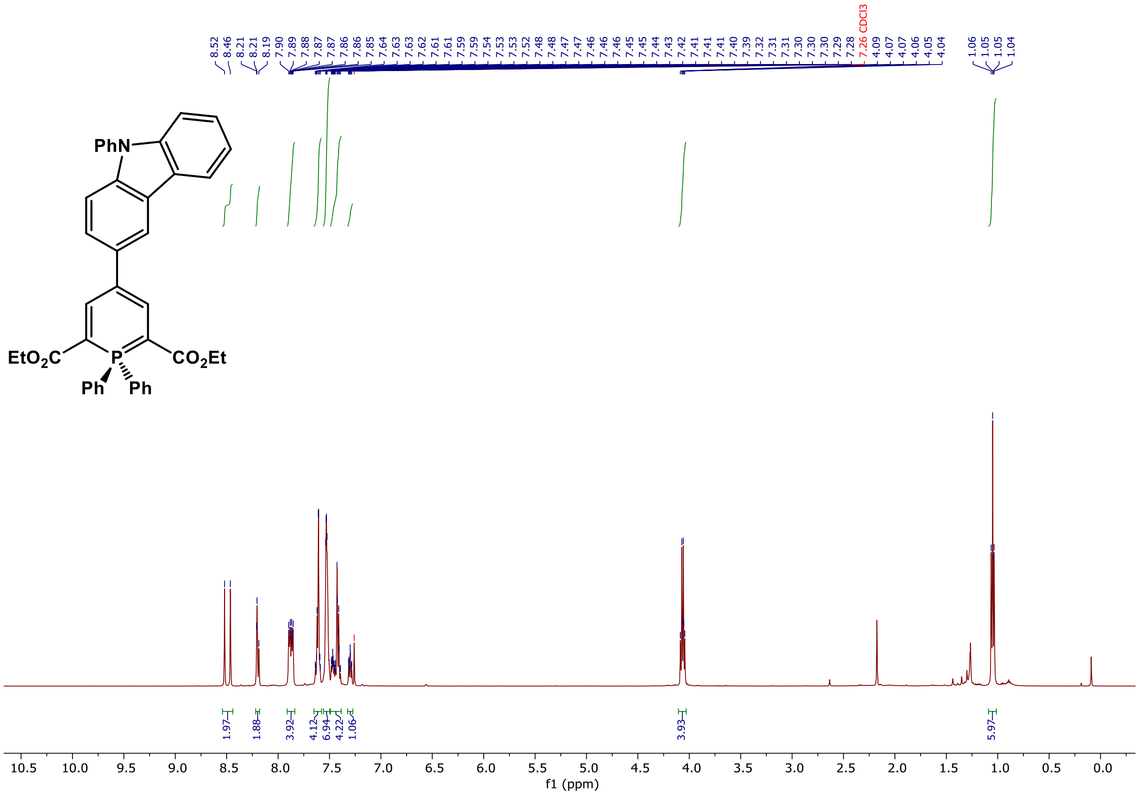

^{13}C NMR (126 MHz)

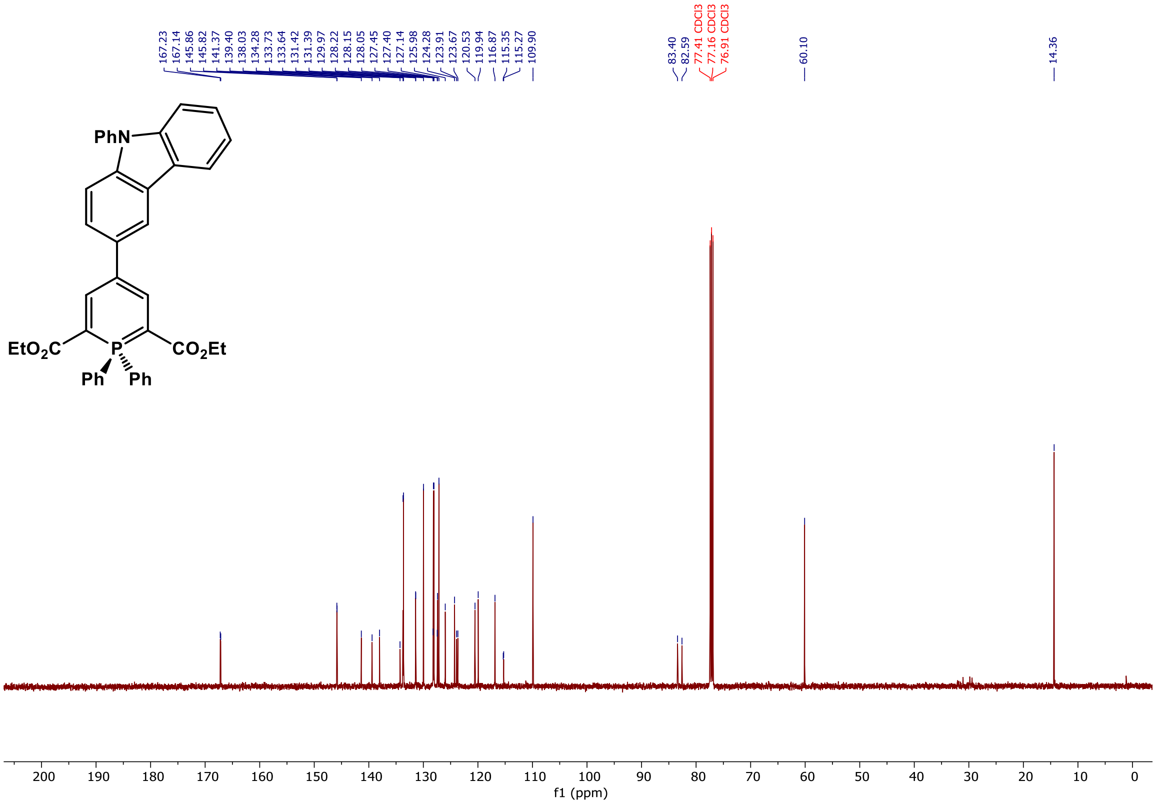

^{31}P NMR (202 MHz)

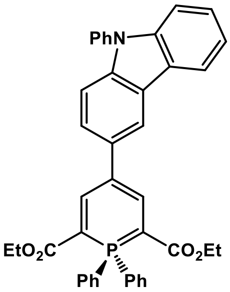

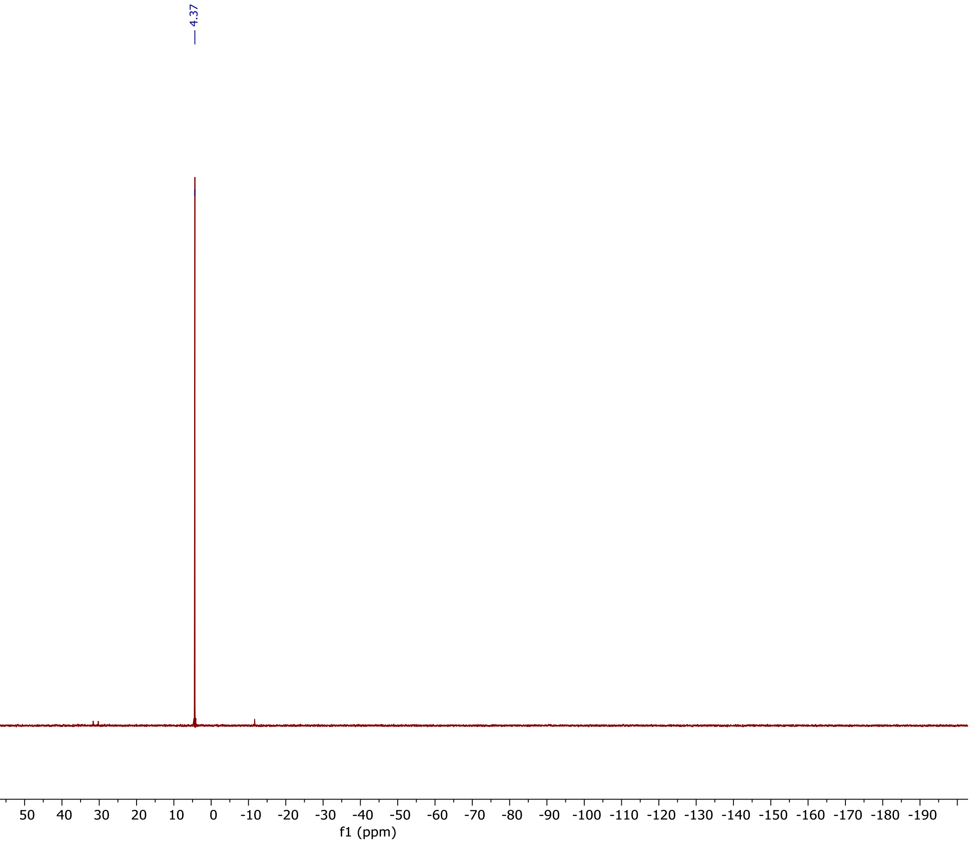

diethyl 4-(1-(*tert*-butoxycarbonyl)-1*H*-pyrrolo[2,3-*c*]pyridin-3-yl)-1,1-diphenyl-1λ^5-phosphinine-2,6-dicarboxylate (5i)

^{1}H NMR (500 MHz)

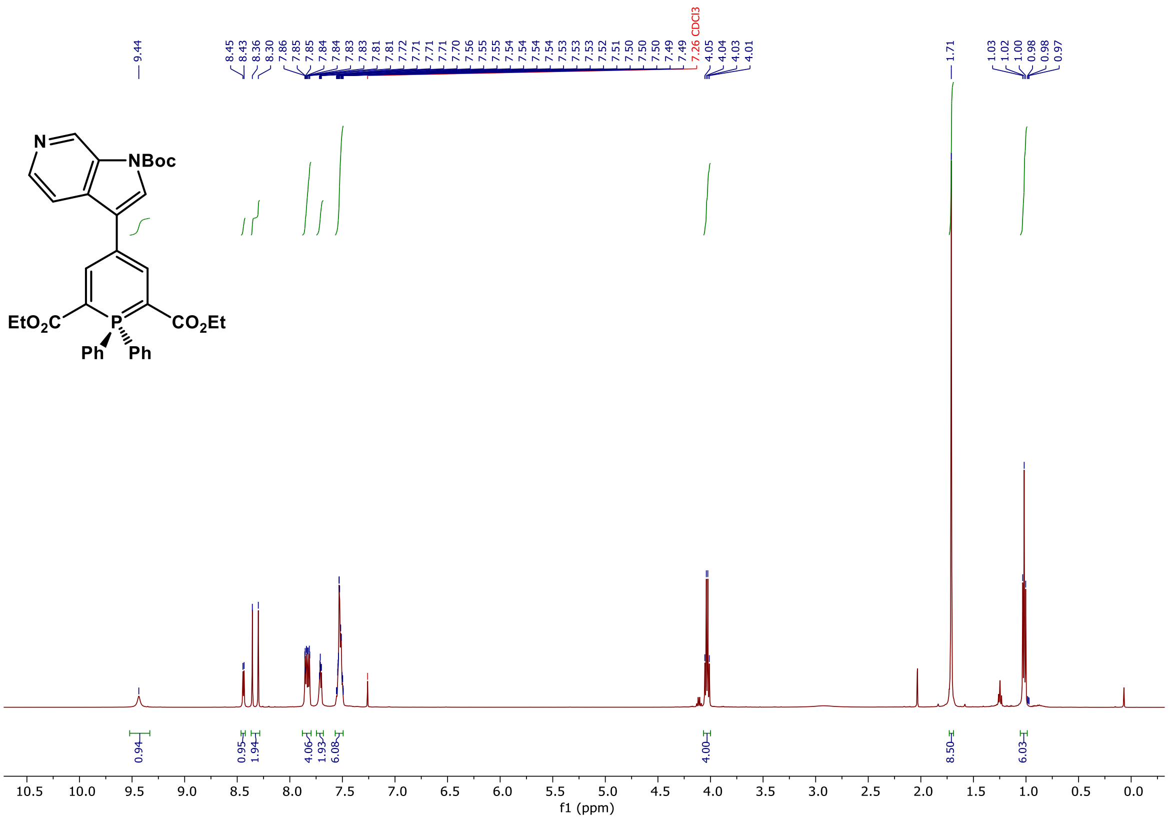

^{13}C NMR (126 MHz)

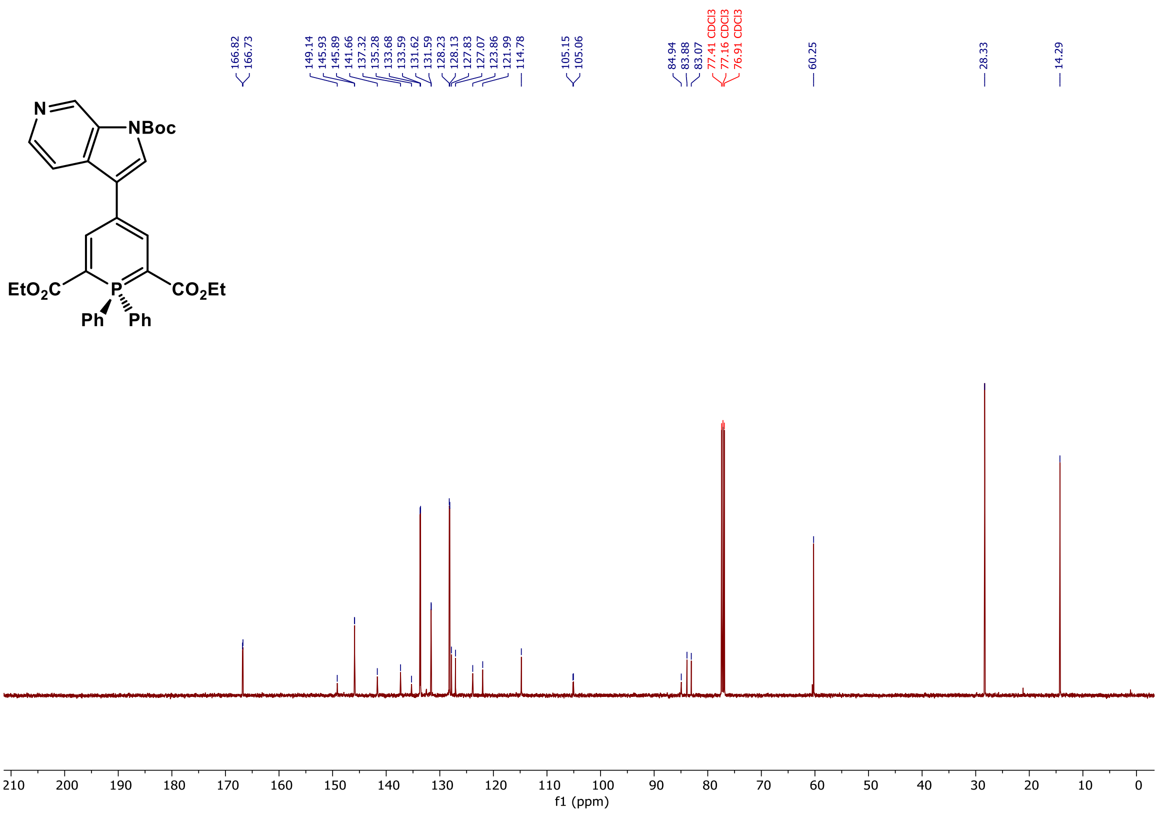

^{31}P NMR (202 MHz)

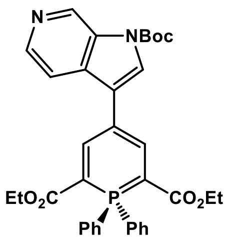

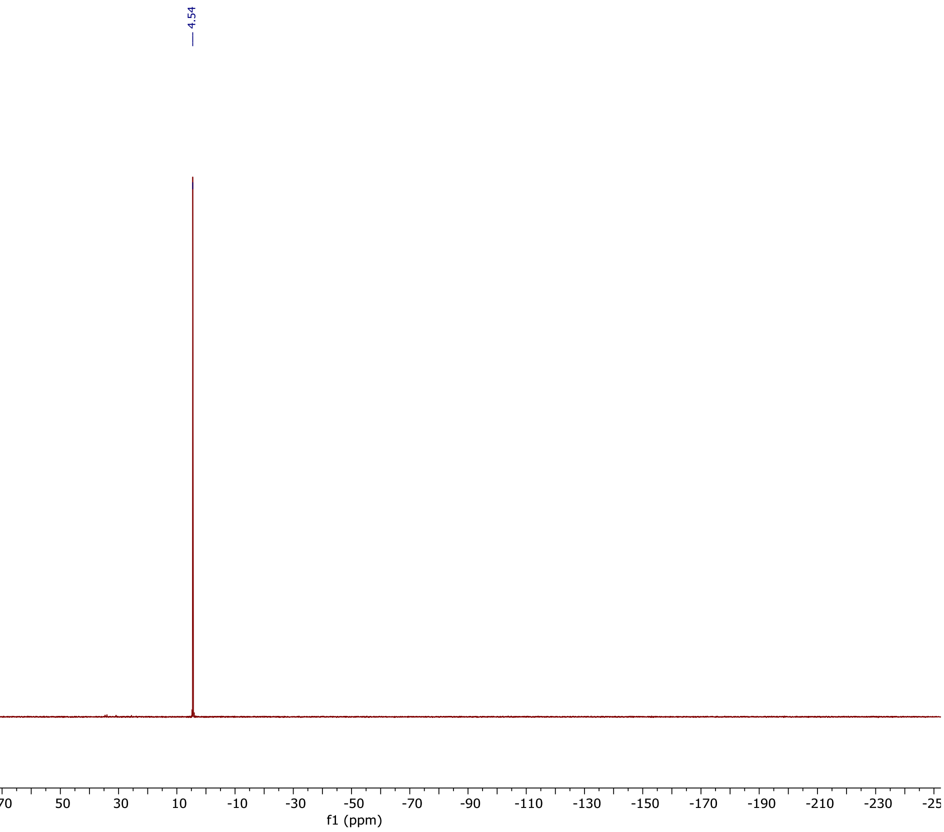

diethyl 4-(3,5-bis(trifluoromethyl)phenyl)-1,1-diphenyl-1λ^5-phosphinine-2,6-dicarboxylate (5j)

^{1}H NMR (500 MHz)

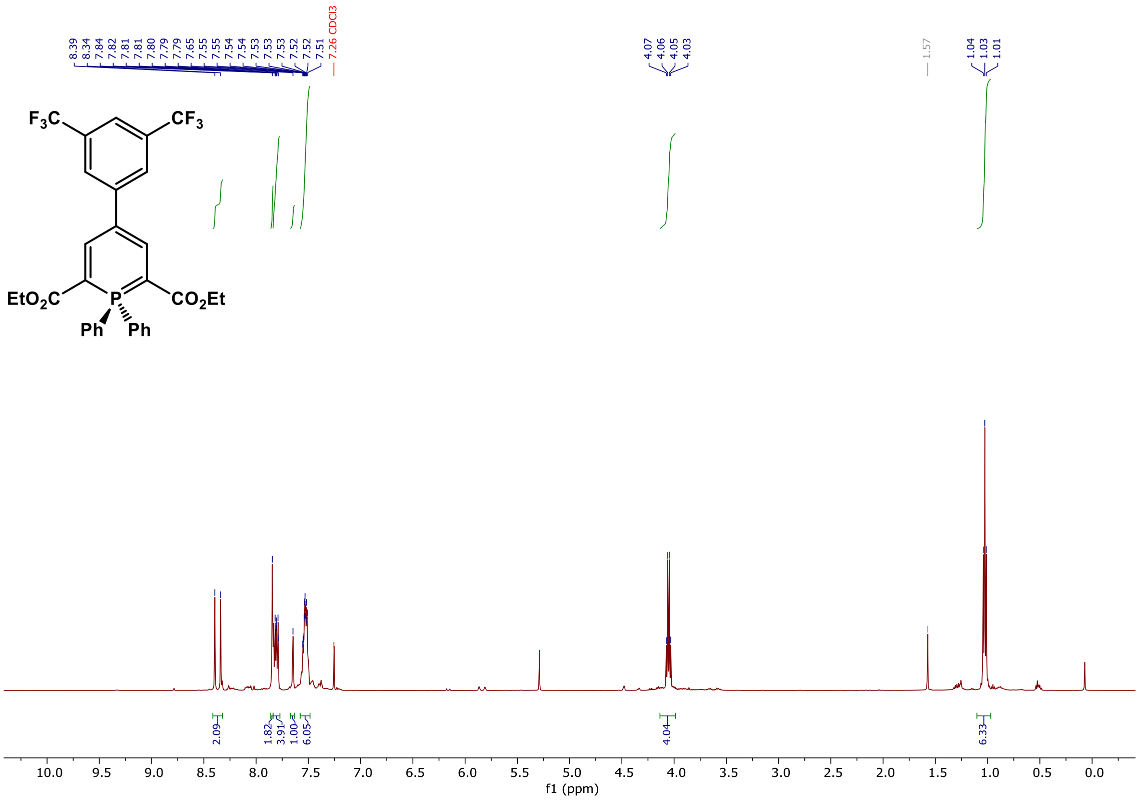

^{13}C NMR (126 MHz)

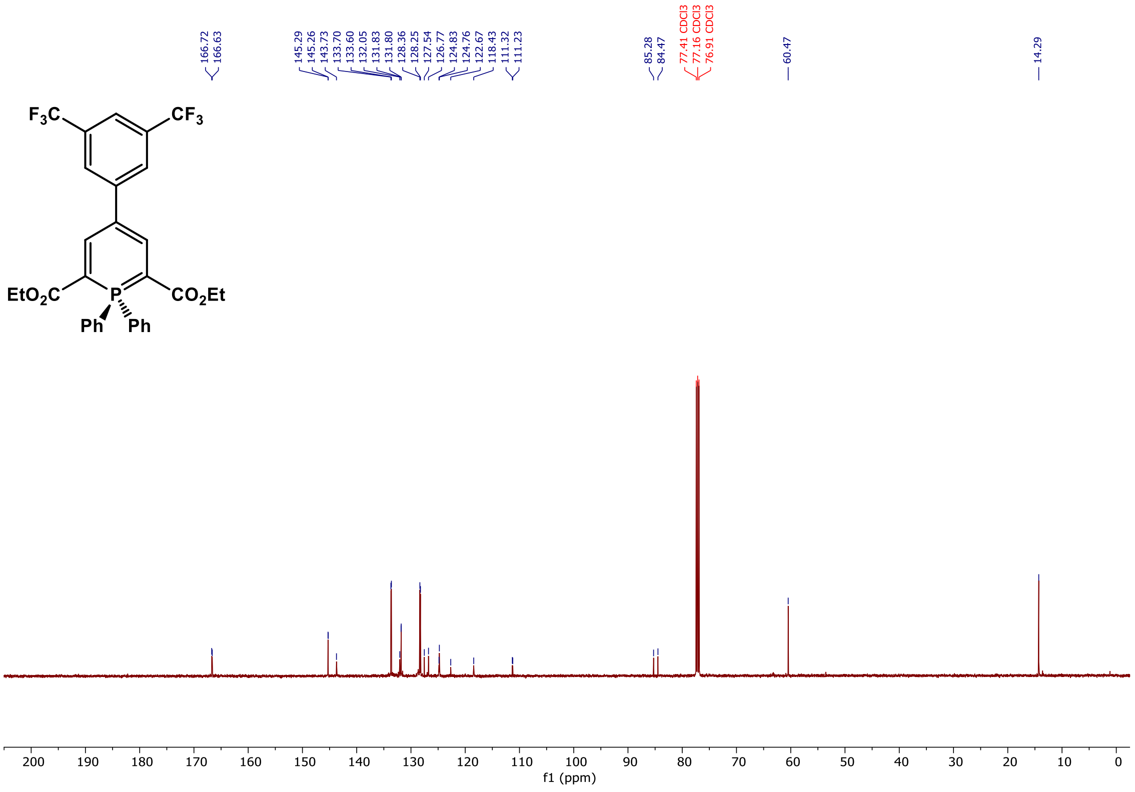

^{31}P NMR (202 MHz)

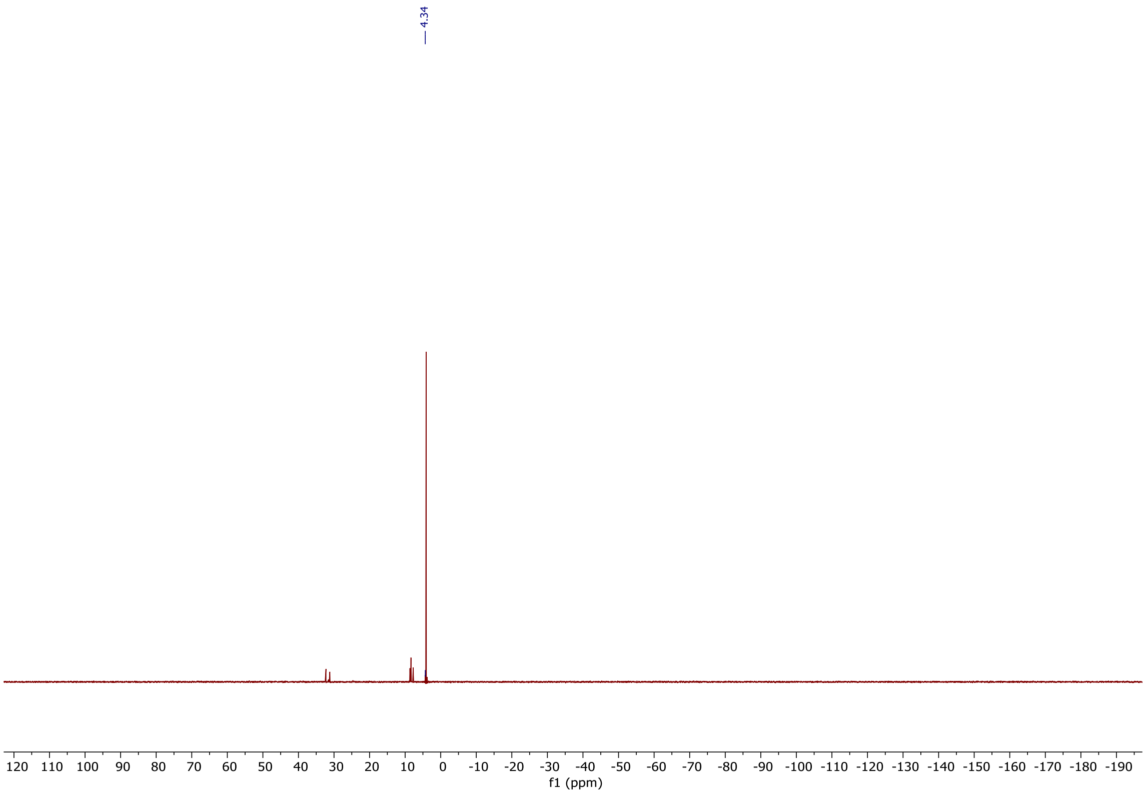

^{19}F NMR (471 MHz)

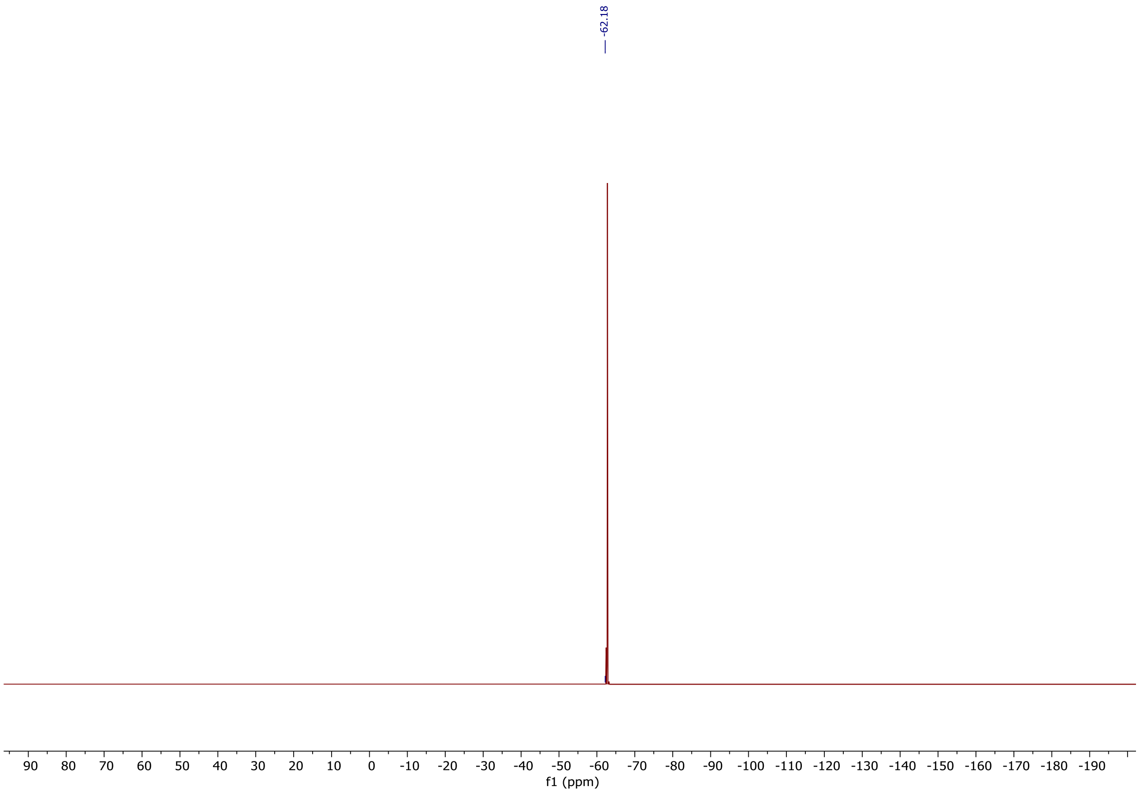

diethyl 1,1-diphenyl-4-(quinolin-6-yl)-1λ^5-phosphinine-2,6-dicarboxylate (5k)

^{1}H NMR (500 MHz)

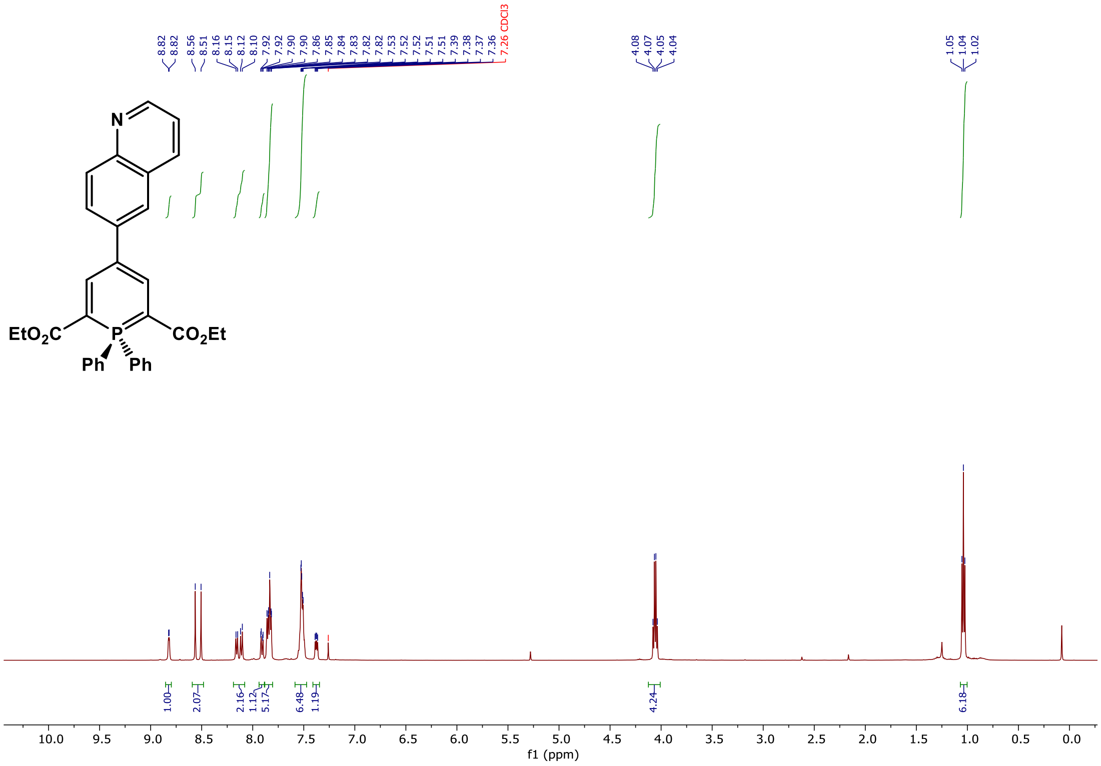

^{13}C NMR (126 MHz)

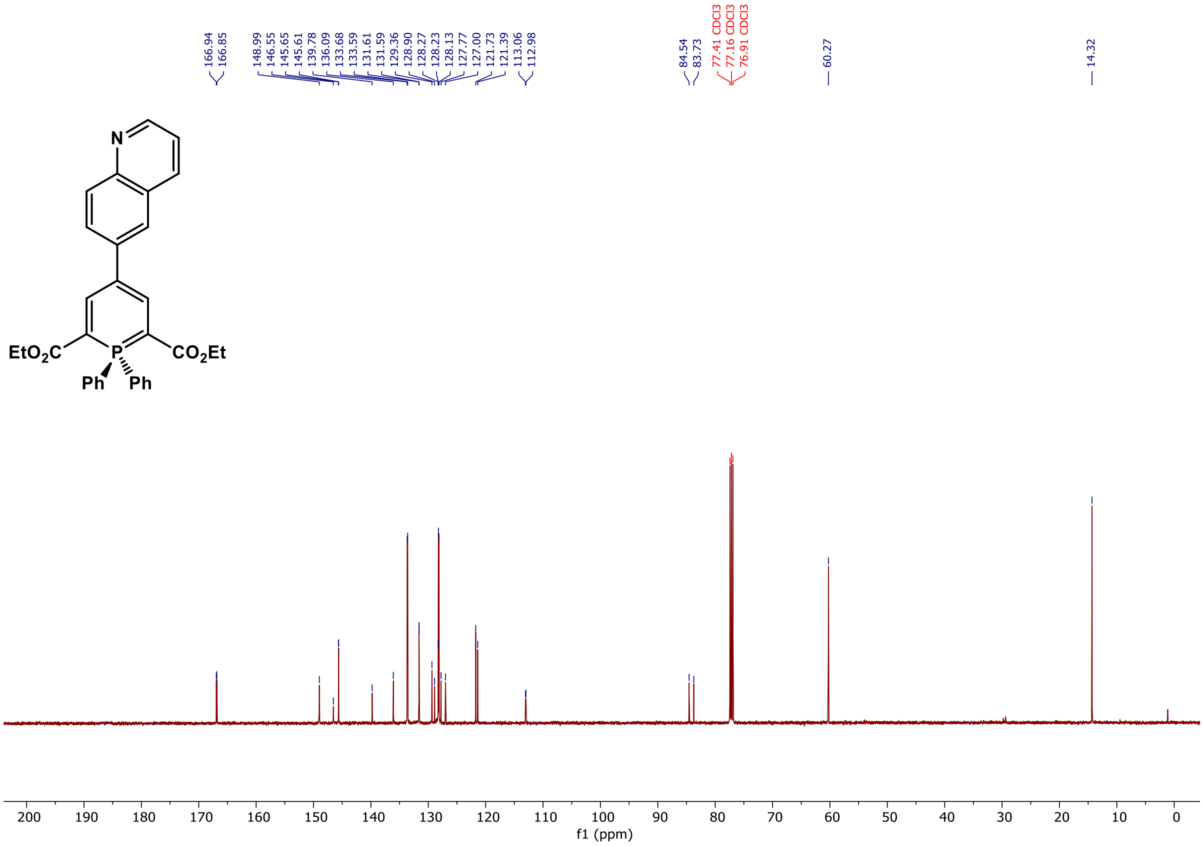

^{31}P NMR (202 MHz)

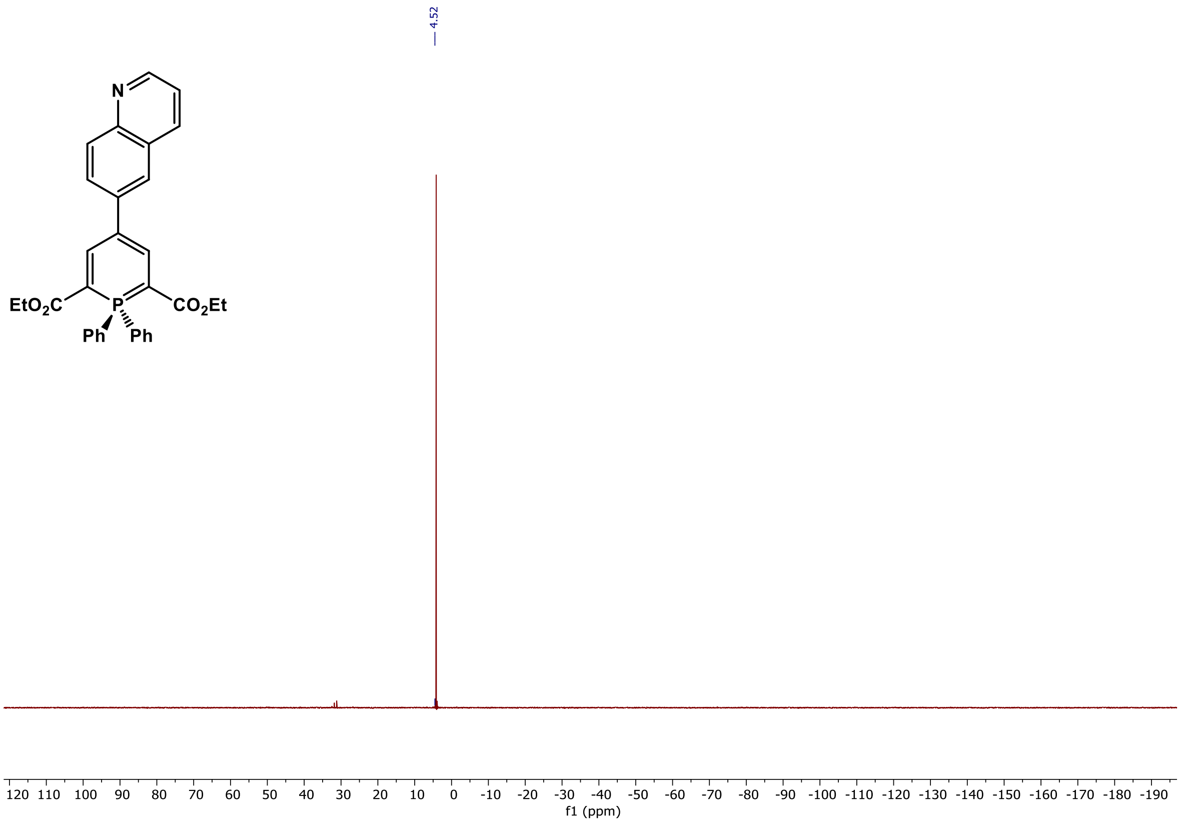

I/Mg Exchange

4-formyl-1,1-diphenyl-1λ^5-phosphinine-2,6-dicarbonitrile (4a)

^{1}H NMR (500 MHz)

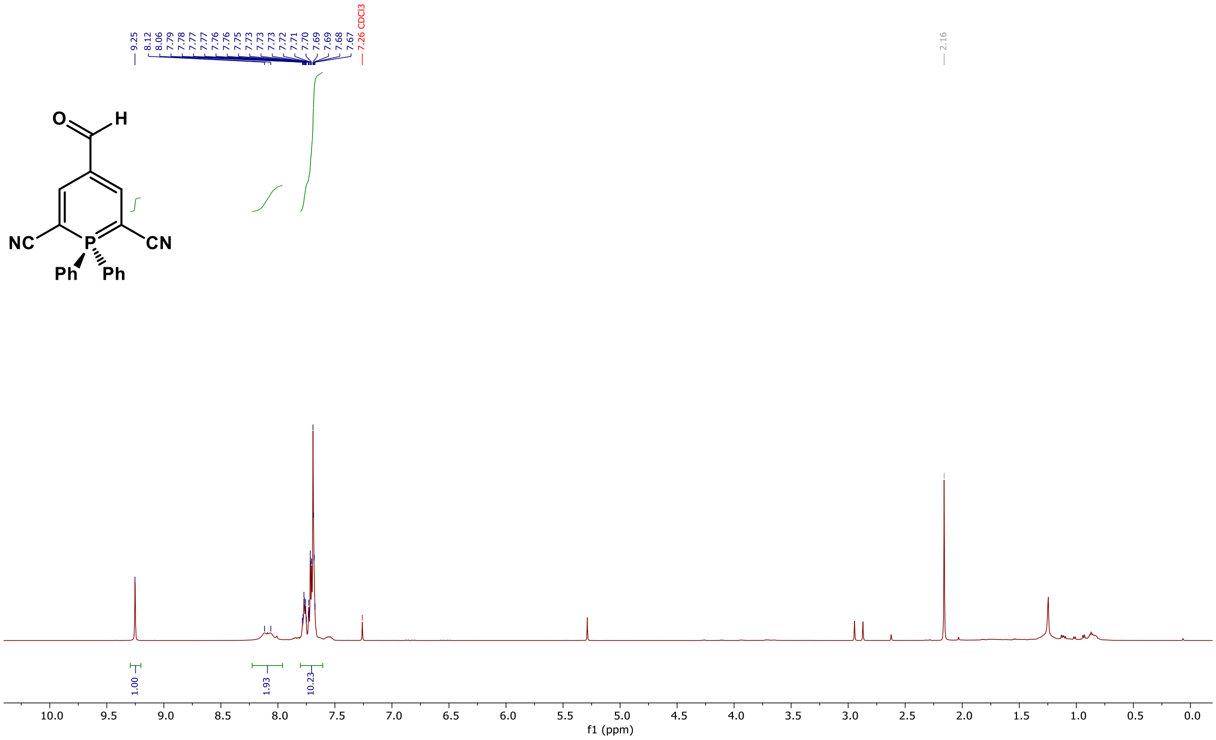

^{13}C NMR (126 MHz)

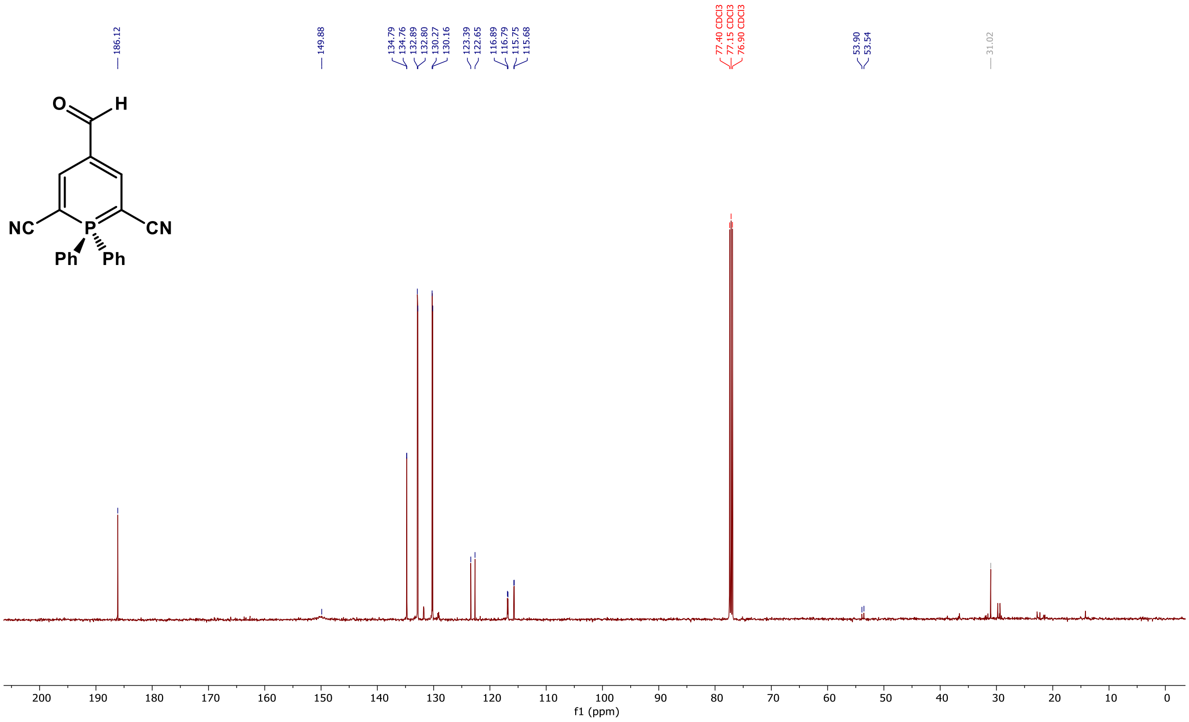

^{31}P NMR (202 MHz)

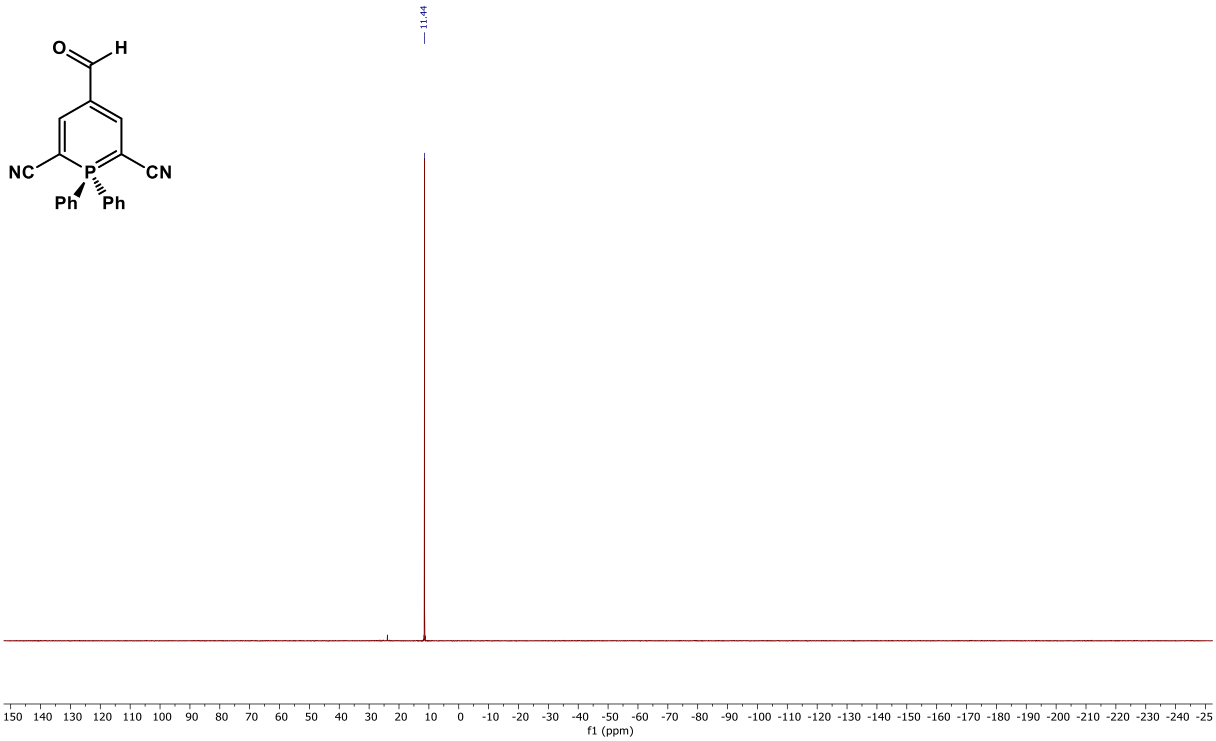

2,6-dicyano-1,1-diphenyl-1λ^5-phosphinine -4-carboxylic acid (4b)

^{1}H NMR (500 MHz)

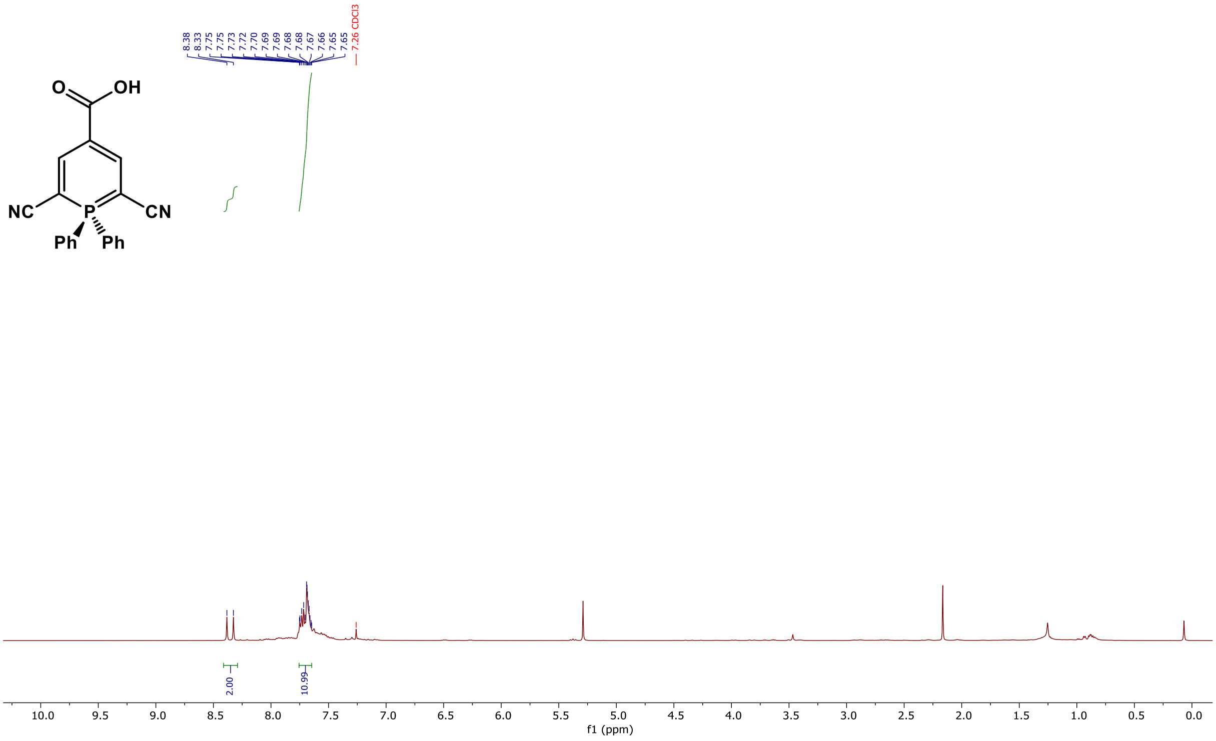

^{13}C NMR (126 MHz)

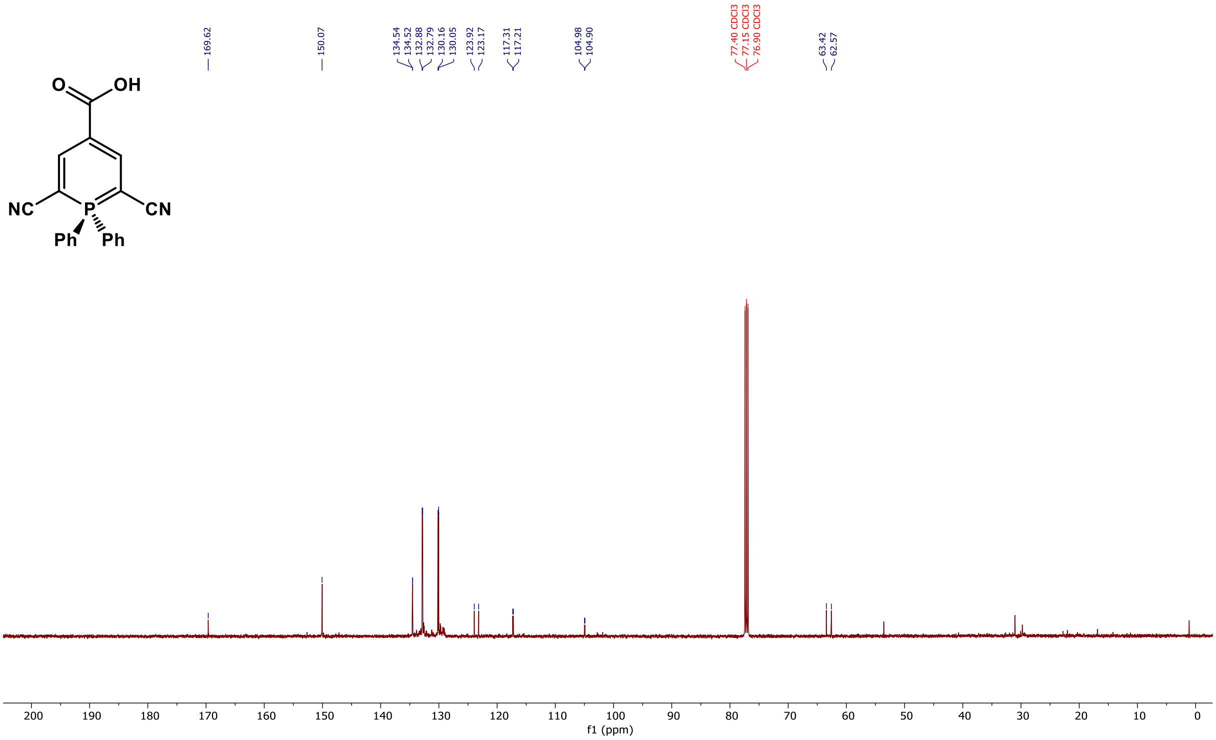

^{31}P NMR (202 MHz)

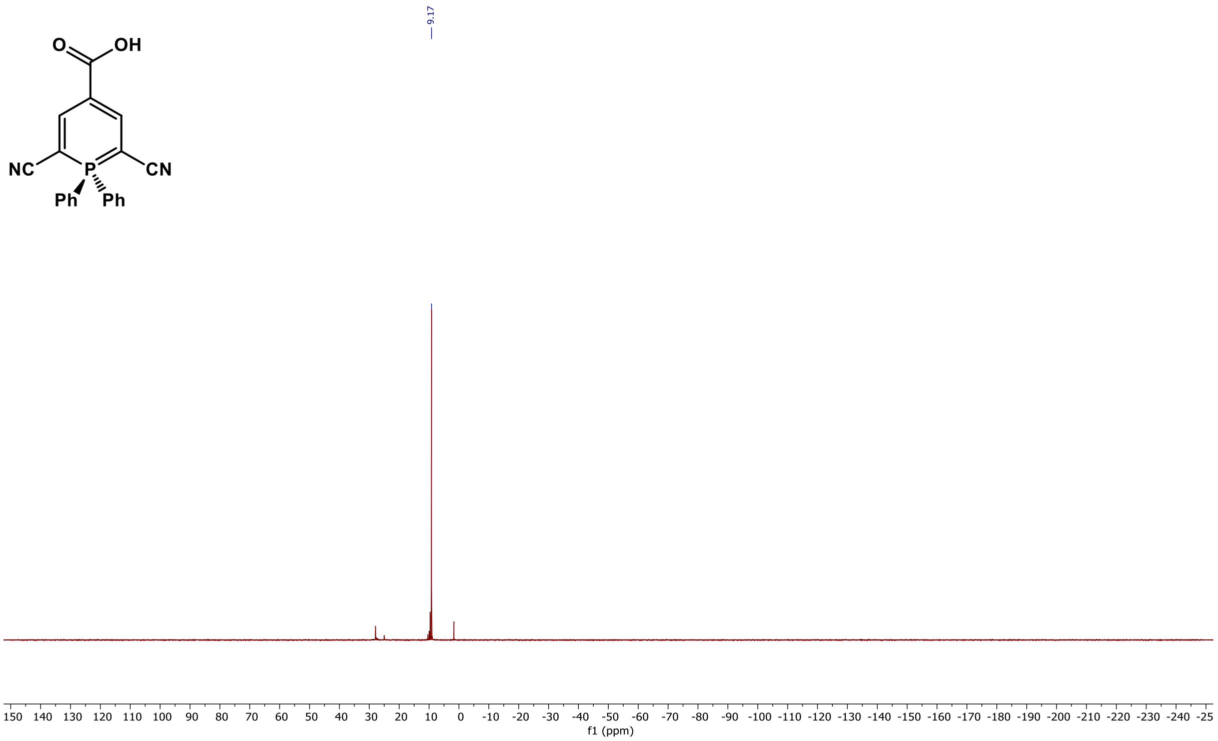

4-((2,4-dichlorophenyl)(hydroxy)methyl)-1,1-diphenyl-1λ^5-phosphinine -2,6-dicarbonitrile (4c)

^{1}H NMR (500 MHz)

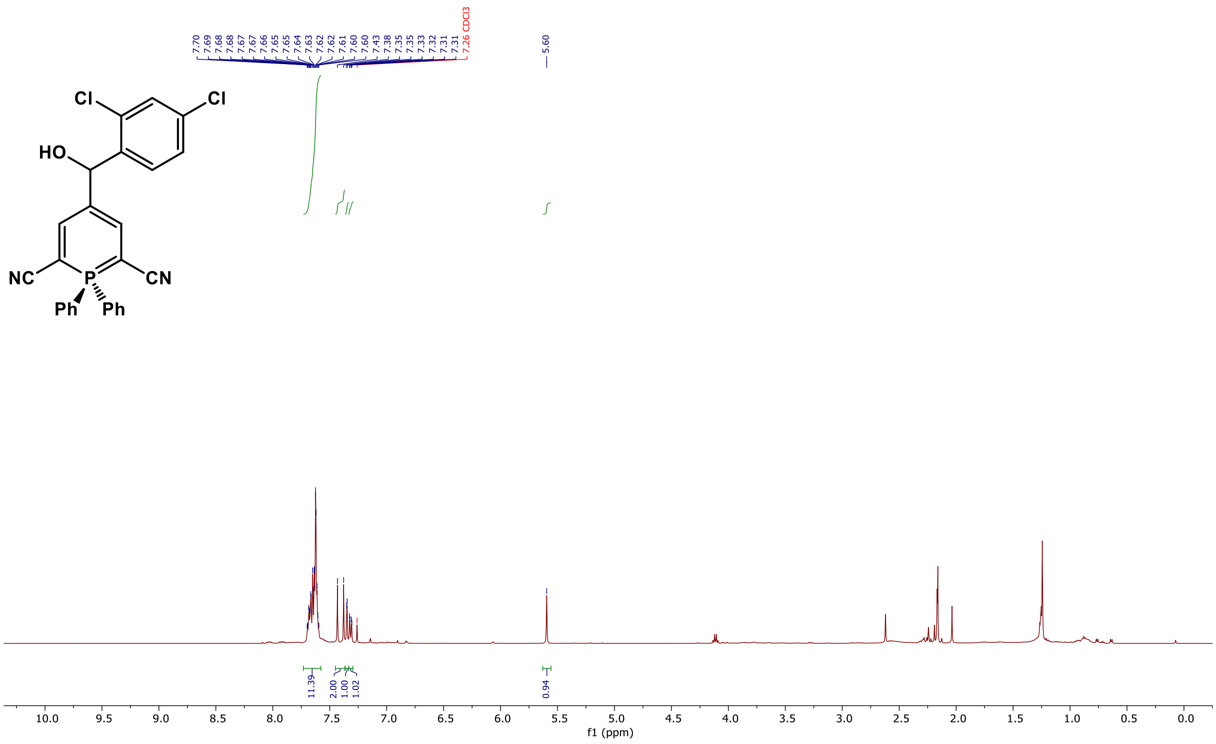

^{13}C NMR (126 MHz)

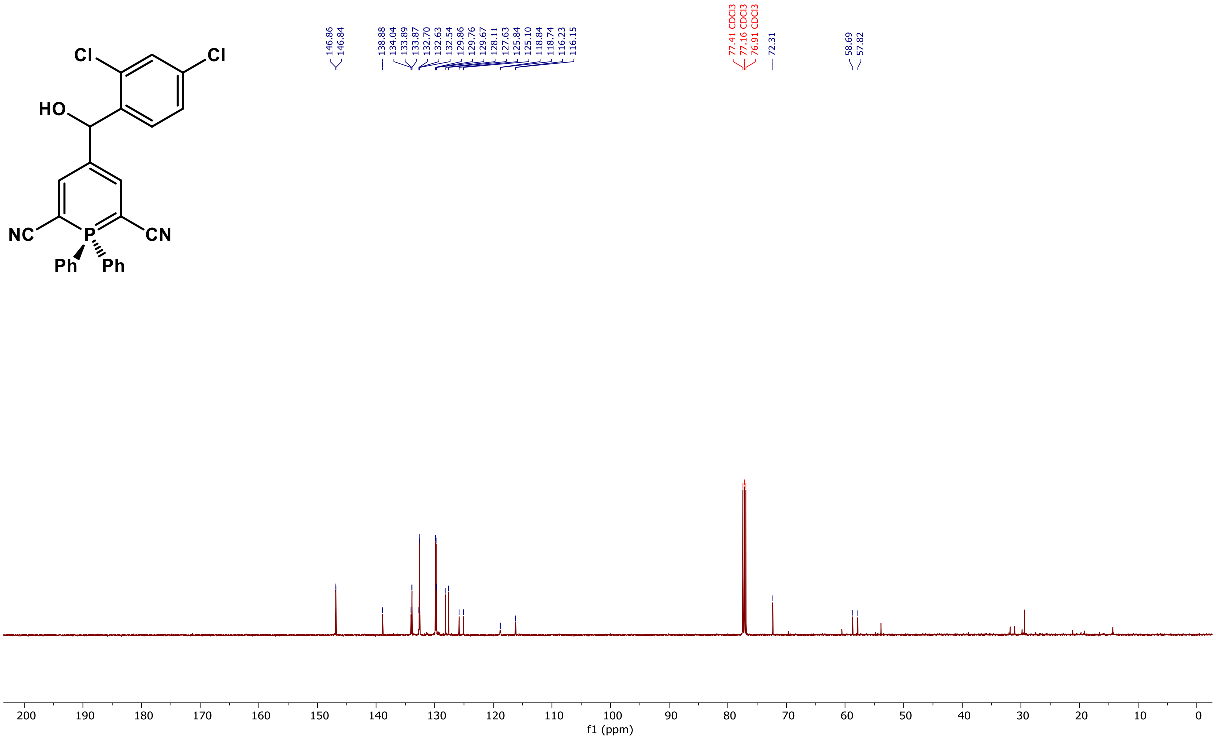

^{31}P NMR (202 MHz)

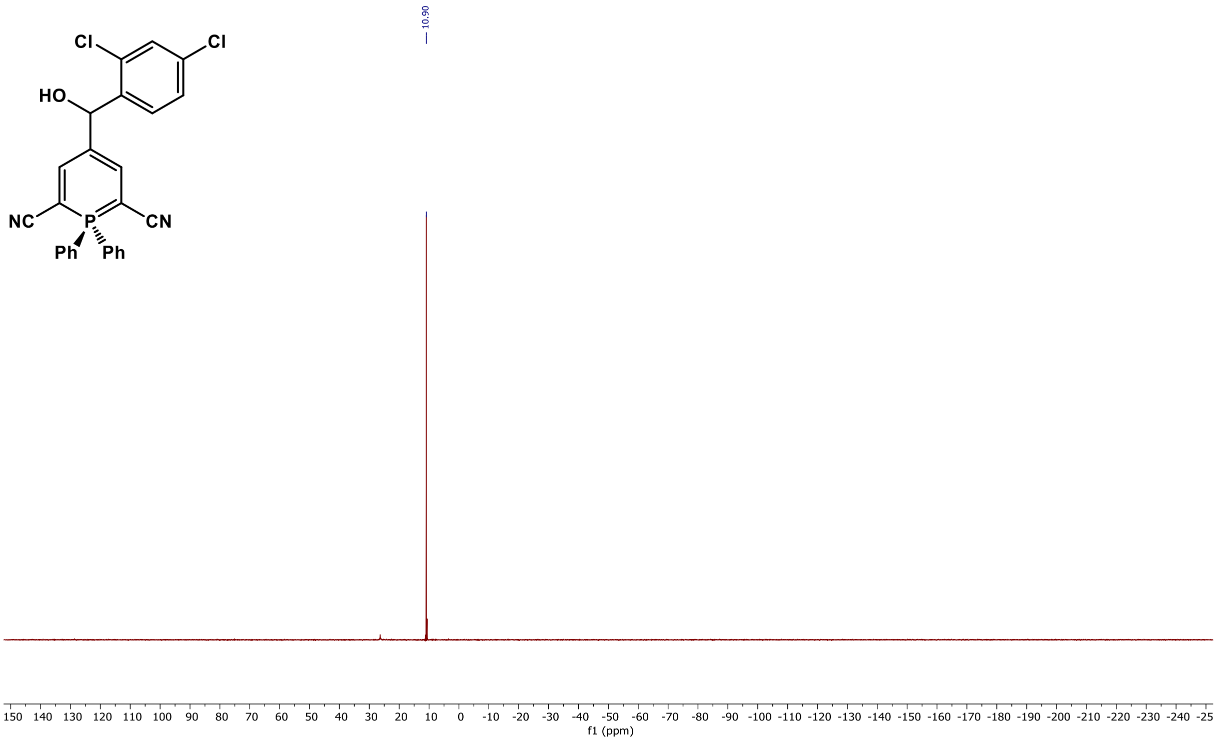

1,1-diphenyl-4-(4,4,5,5-tetramethyl-1,3,2-dioxaborolan-2-yl)-1λ^5-phosphinine-2,6-dicarbonitrile (4d)

^{1}H NMR (500 MHz)

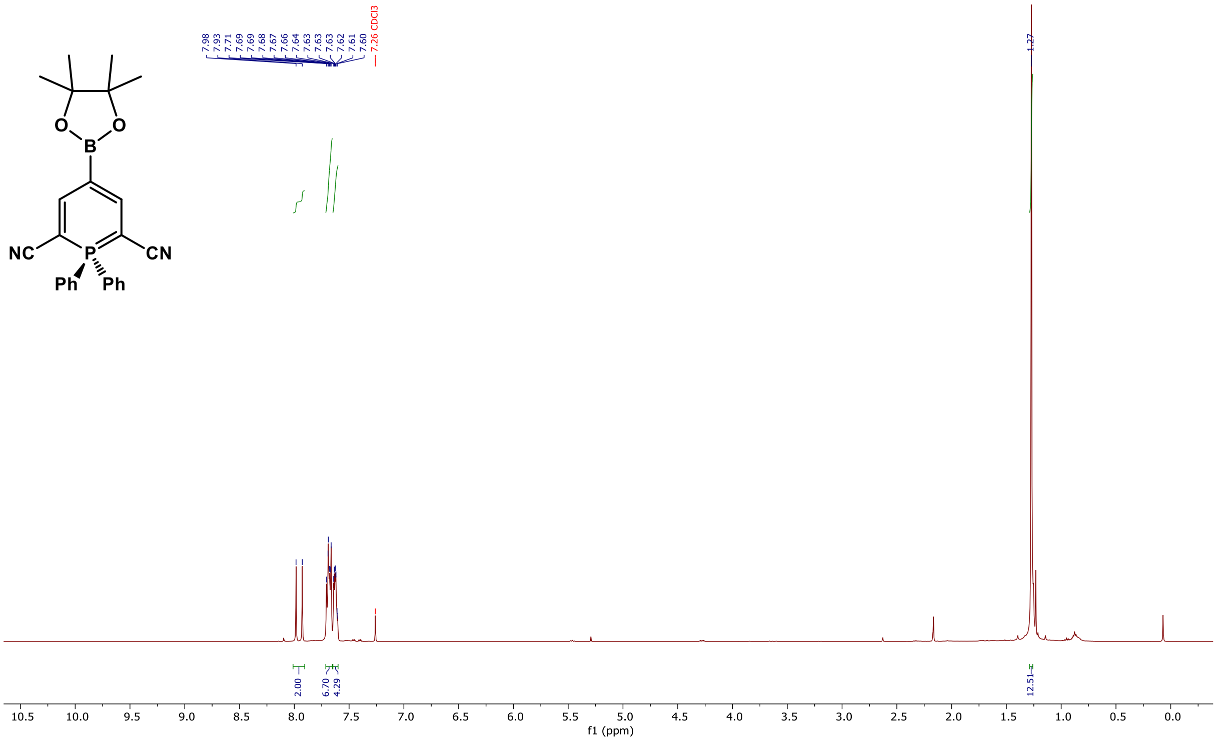

^{13}C NMR (126 MHz)

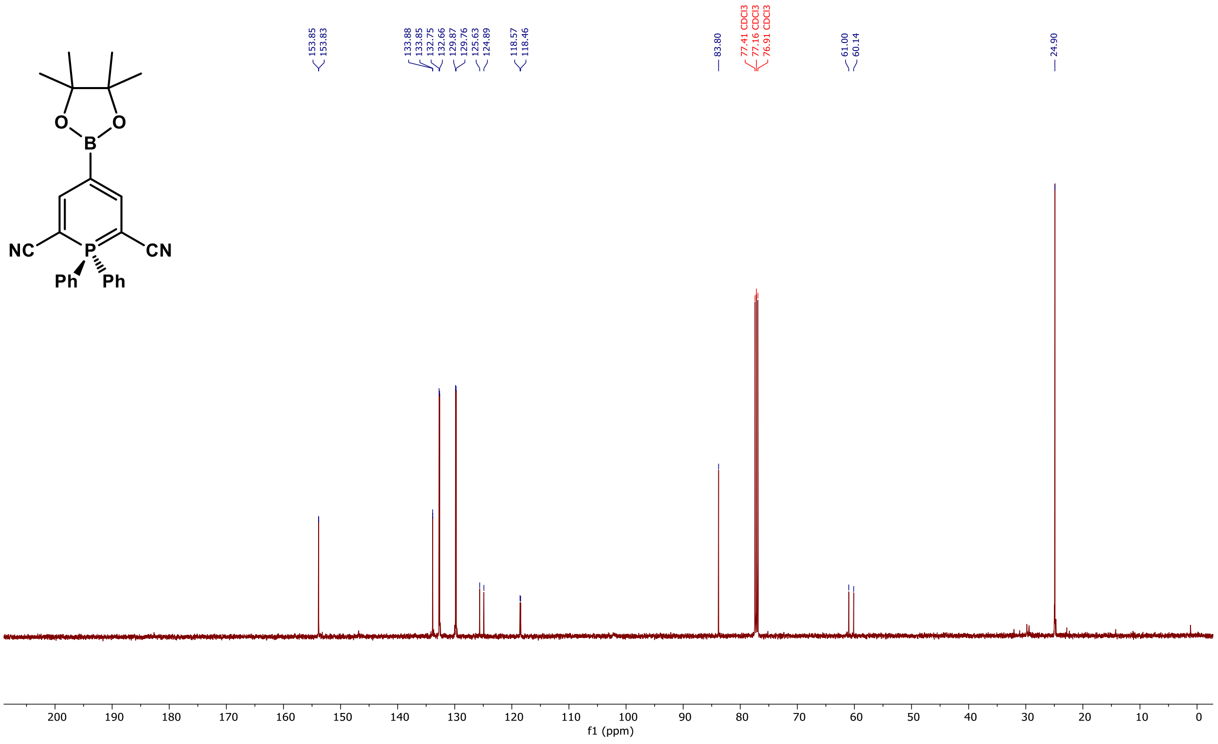

^{31}P NMR (202 MHz)

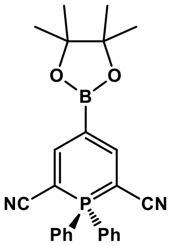

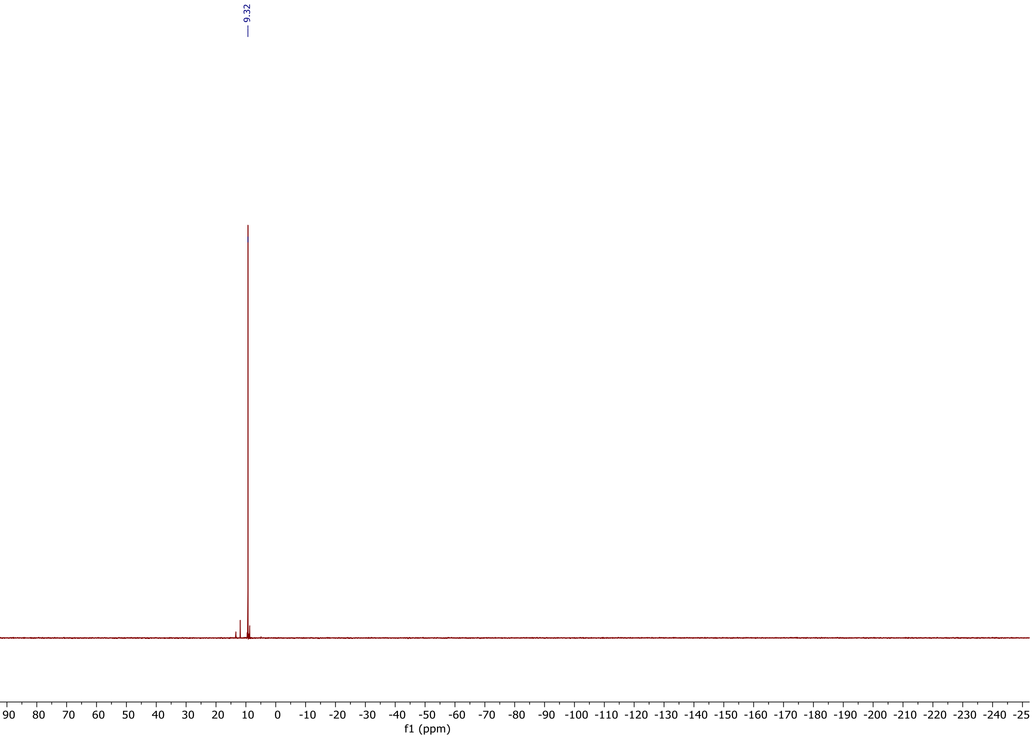

diethyl 4-formyl-1,1-diphenyl-1λ^5-phosphinine-2,6-dicarboxylate (6a)

^{1}H NMR (500 MHz)

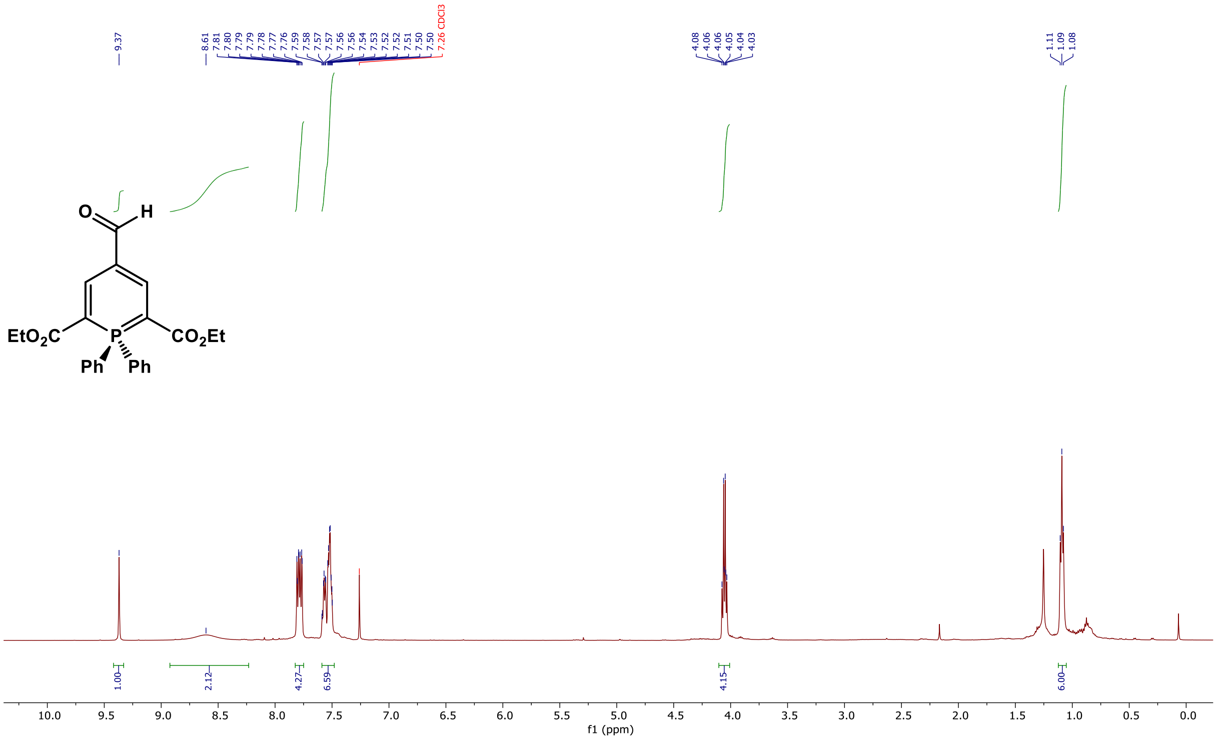

^{13}C NMR (126 MHz)

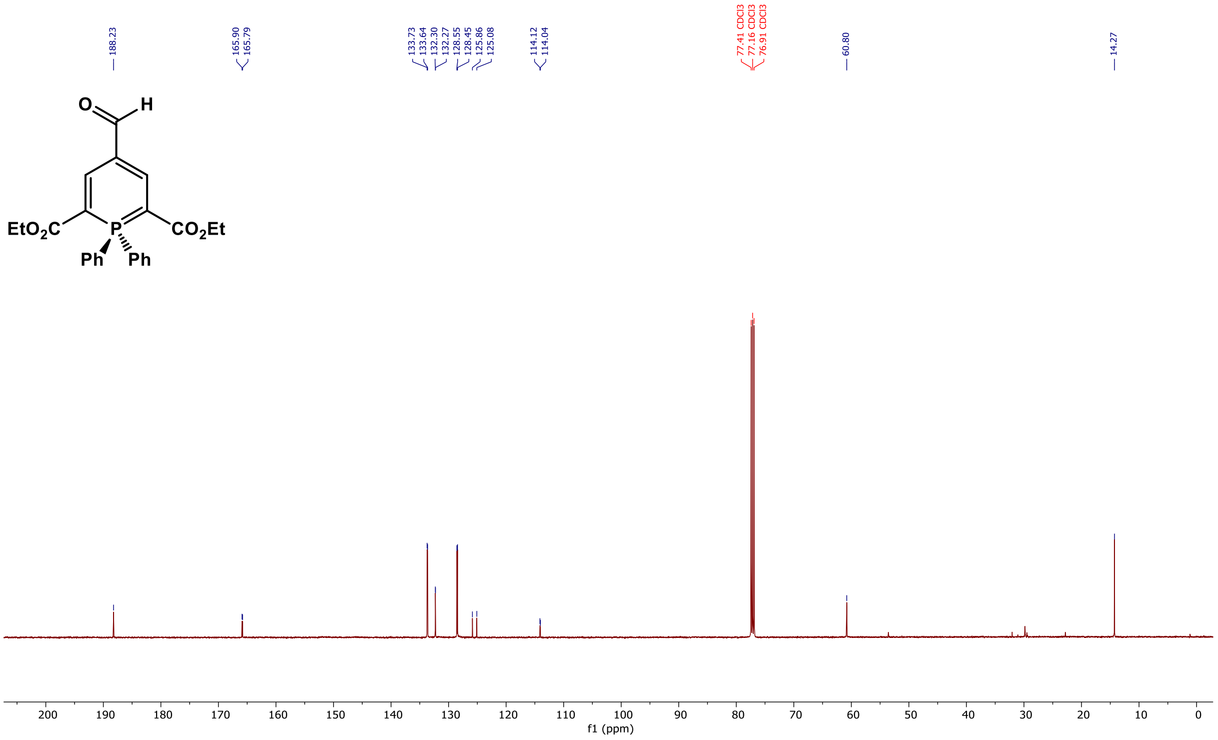

^{31}P NMR (202 MHz)

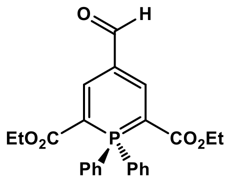

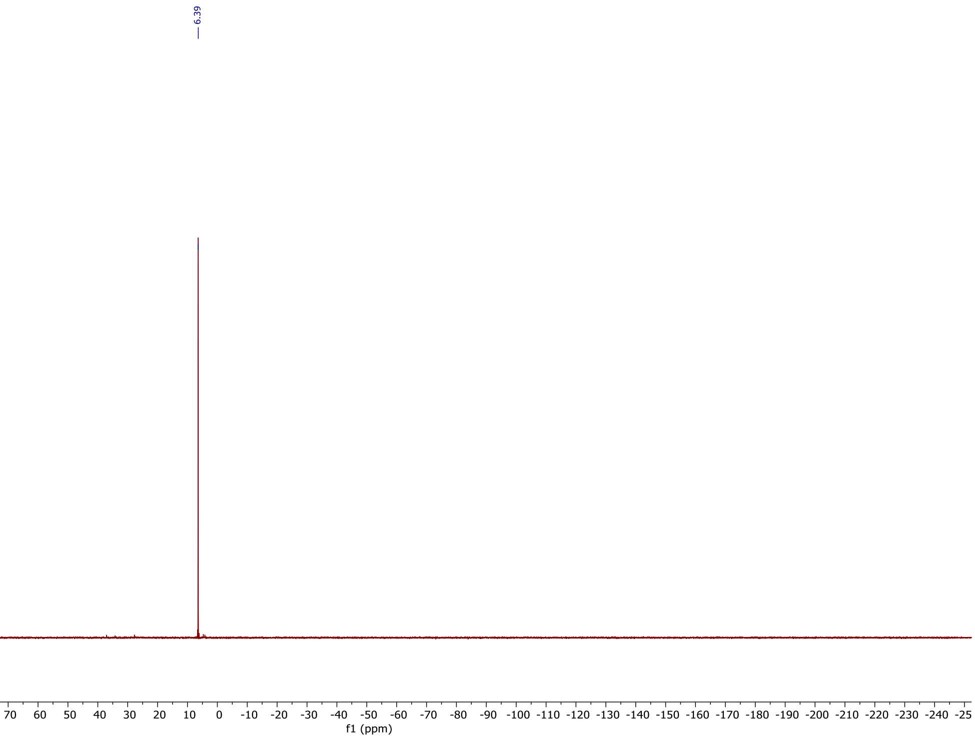

2,6-bis(ethoxycarbonyl)-1,1-diphenyl-1λ[5]-phosphinine-4-carboxylic acid (6b)

^{1}H NMR (500 MHz)

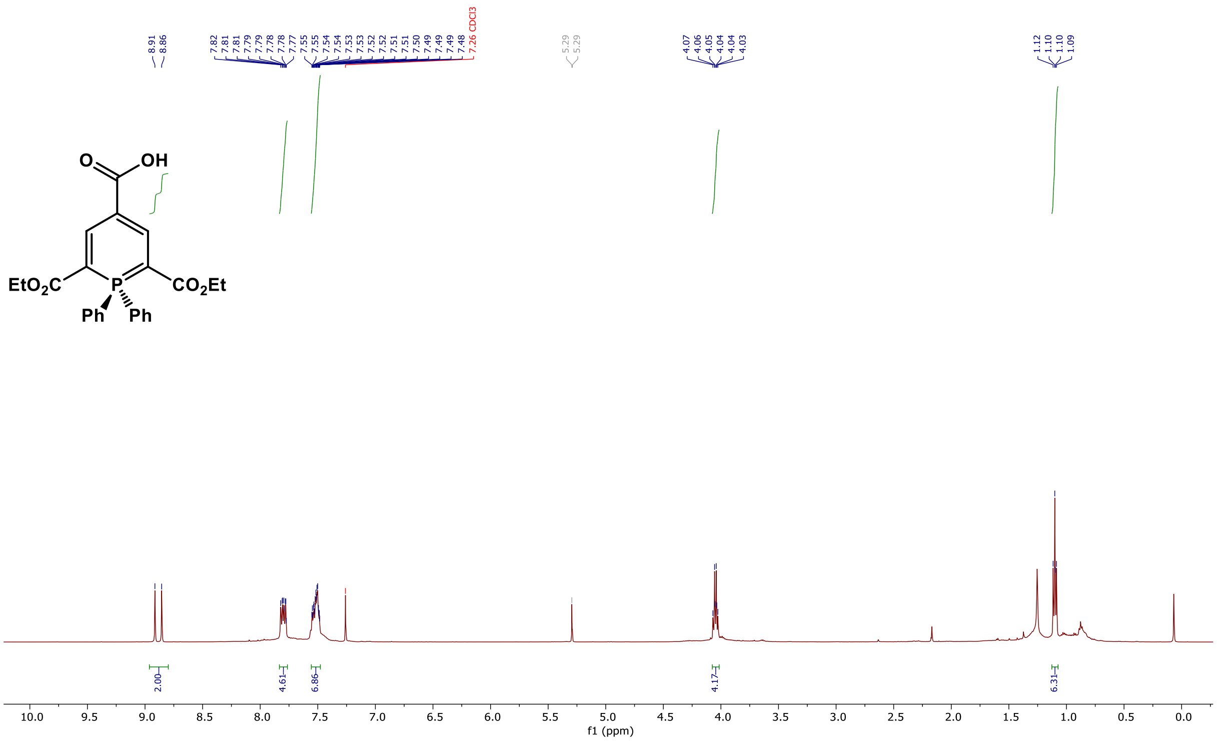

^{13}C NMR (126 MHz)

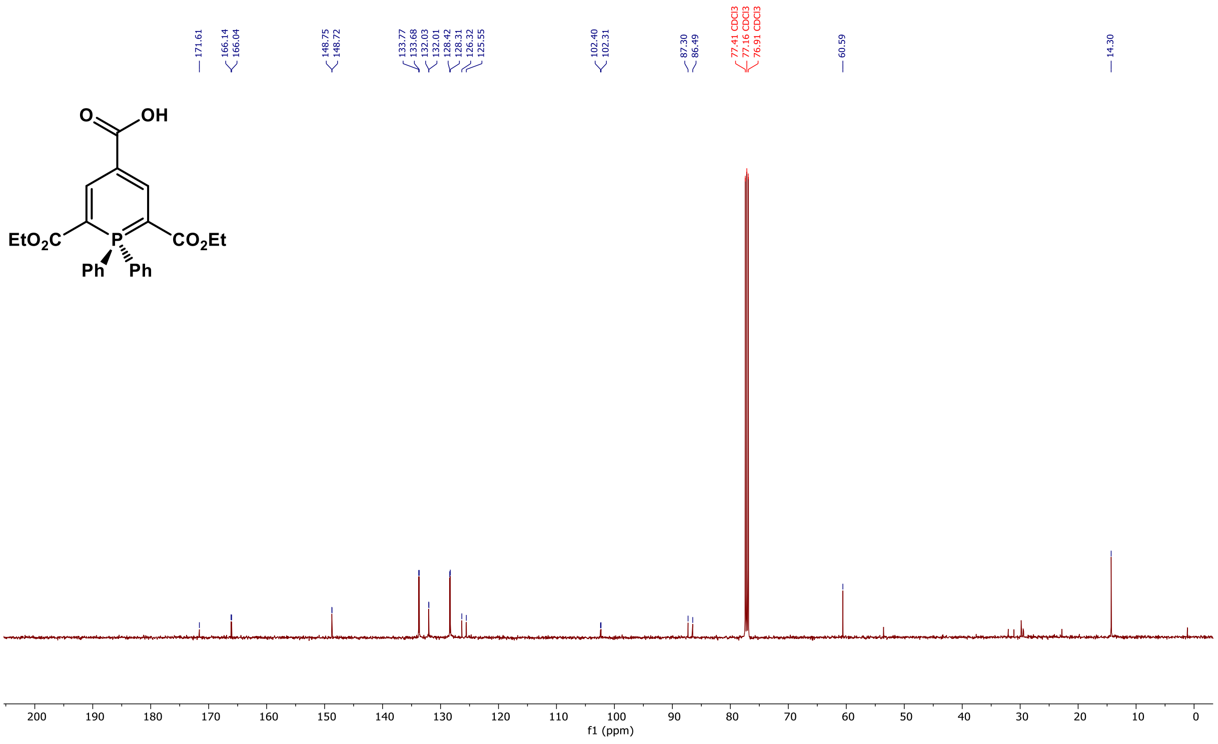

^{31}P NMR (202 MHz)

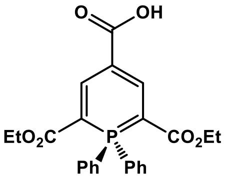

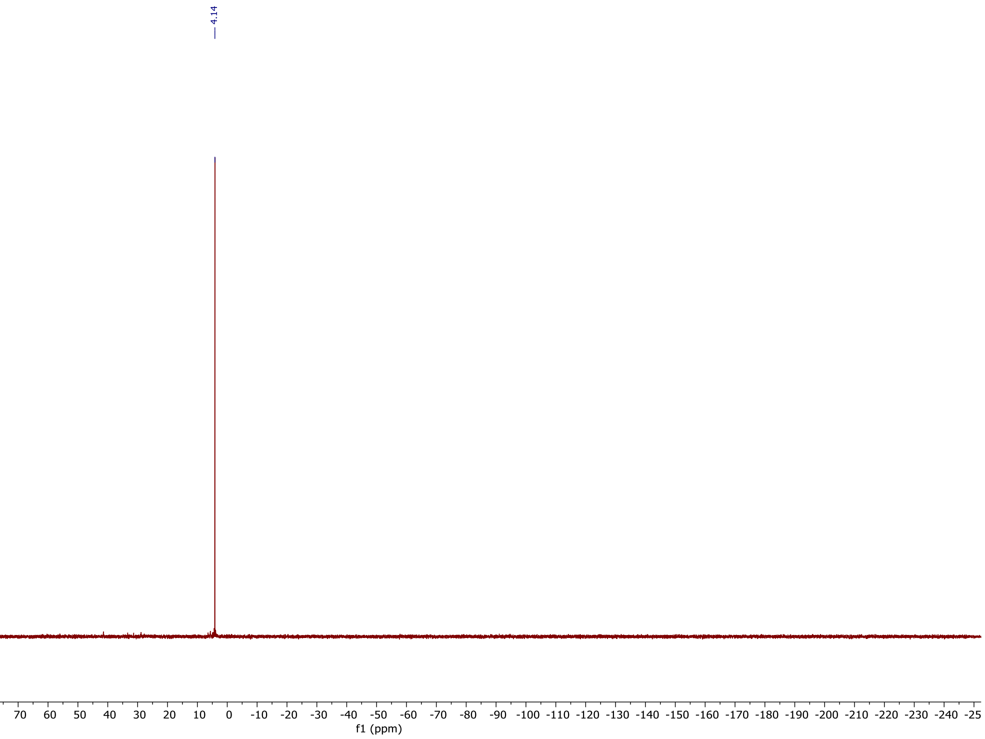

Supplement: Supplementary file 3 — Supplementary Data 1 [file 42004_2025_1822_MOESM3_ESM.pdf]
