# Supplementary material for: Functionalization of λ5-Phosphinines via metalation strategies
Source: Commun Chem. 2025 Dec 22;8:414. doi: 10.1038/s42004-025-01822-6 (PMC12748612; doi:10.1038/s42004-025-01822-6)

Functionalization of λ^5-Phosphinines *via* Metalation Strategies

Flavie Rambaud,[a] Bertrand Takam Fotie,[a] Robert Naumann,[b] Katja Heinze,[b] Dorian Didier*[a]

[a] Technische Universität Darmstadt, Clemens-Schöpf-Institut, Peter-Grünberg-Straße 4, 64287 Darmstadt

[b] Johannes Gutenberg Universität Mainz, Department of Chemistry, Duesbergweg 10-14, 55128 Mainz

Absorption and Emission Spectra of *para*-Substituted λ^5-Phosphinines

Spectrometer 1: Fluorescence spectra and absolute emission quantum yields for compounds 3k, 3w, 3z, 3aa, 5h, 5i and 7 were measured with a *FLS1000* spectrometer from *Edinburgh Instruments* equipped with a cooled photomultiplier detector PMT-980. A xenon arc lamp Xe2 (450 W) was used for excitation. Absolute luminescence quantum yields Φ were determined using an integration sphere from *Edinburgh Instruments*. The sample solutions were measured in quartz cuvettes with an optical path length of 1 cm. Relative uncertainty of Φ is estimated to be ± 10%.

Spectrometer 2: UV-Vis for compounds 2a, 2g, 2h, 3d, 3f, 3j, 3l, 3n, 3o, 3r, 3s, 3x and 5a were recorded on Analytik Jena Specord 600 UV-Vis spectrometer, **fluorescence spectra** were recorded on J&M TIDAS S700/CCD UV/NIR 2098 spectrometer combined with J&M TIDAS LSM monochromator with 75 W Xenon light source and thermo-controlled cuvette holder. Samples for emission and absorption measurements were contained in 1 cm quartz cuvette (Hellma Analytics).

<u>Compound 3k</u> (Spectrometer 1)

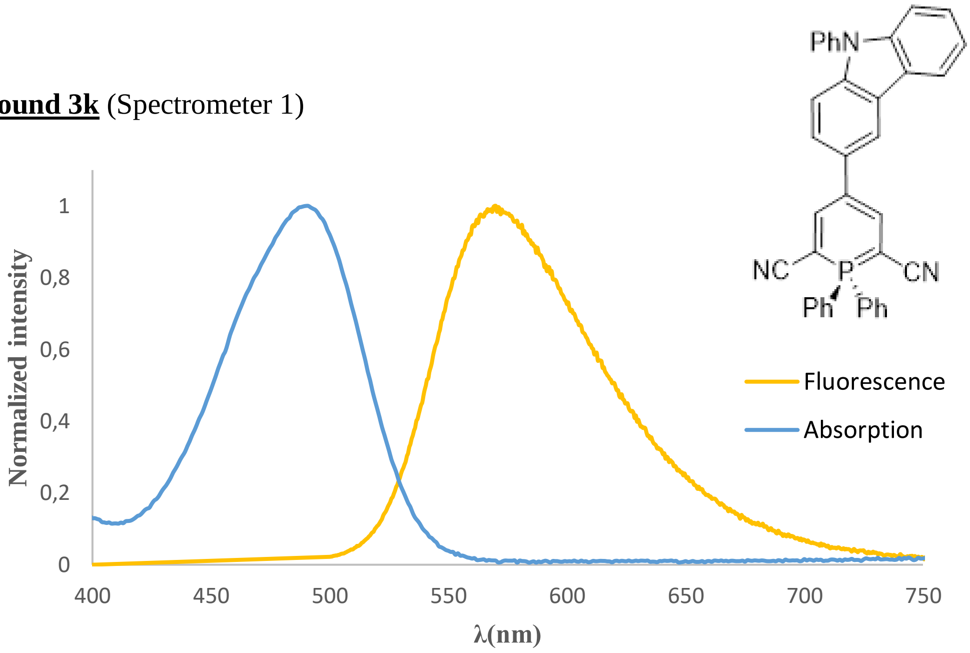

$\lambda_{abs,max}$/nm = 490

$\lambda_{em,max}$/nm = 568

<u>Compound 3w</u> (Spectrometer 1)

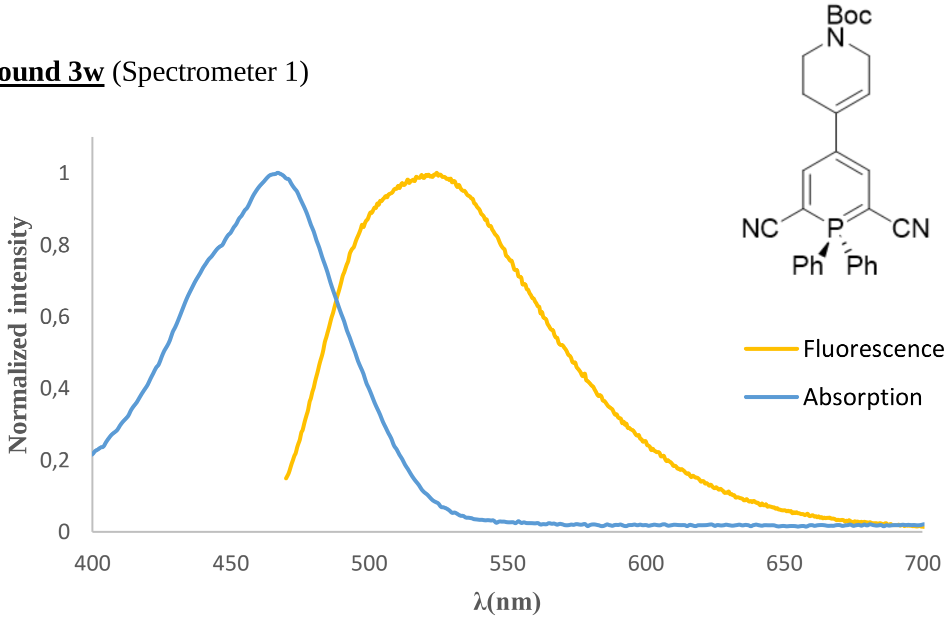

$\lambda_{abs,max}$/nm = 467

$\lambda_{em,max}$/nm = 525

Compound 3z (Spectrometer 1)

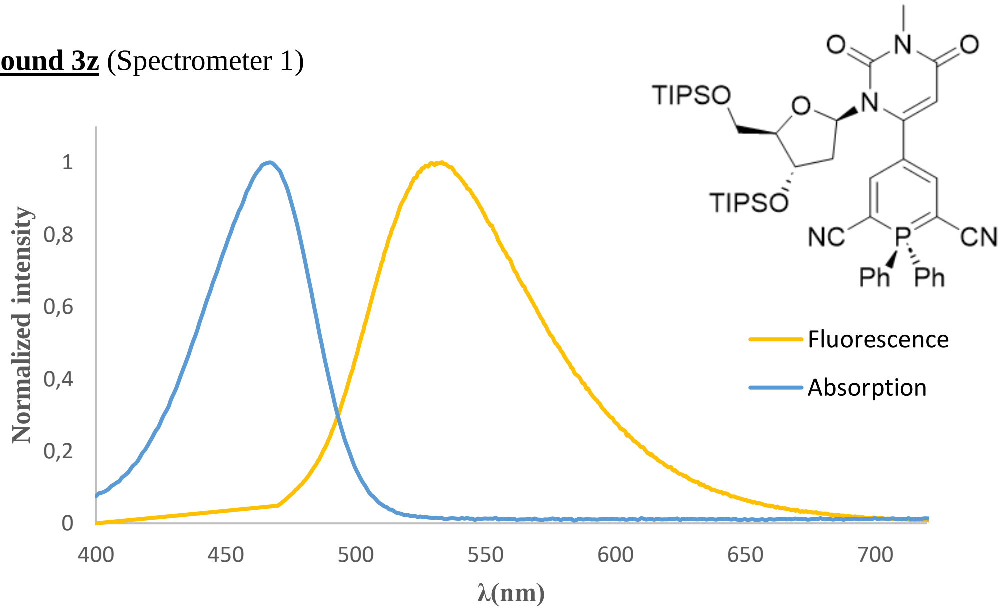

$\lambda_{abs,max}$/nm = 467

$\lambda_{em,max}$/nm = 532

Compound 3aa (Spectrometer 1)

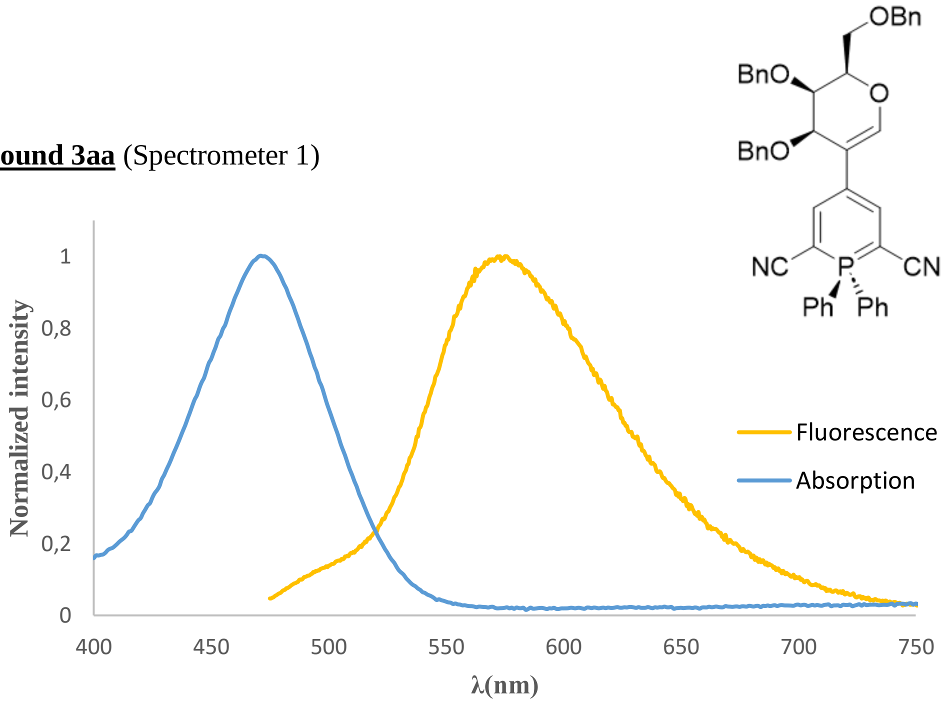

$\lambda_{abs,max}$/nm = 472

$\lambda_{em,max}$/nm = 573

Compound 5h (Spectrometer 1)

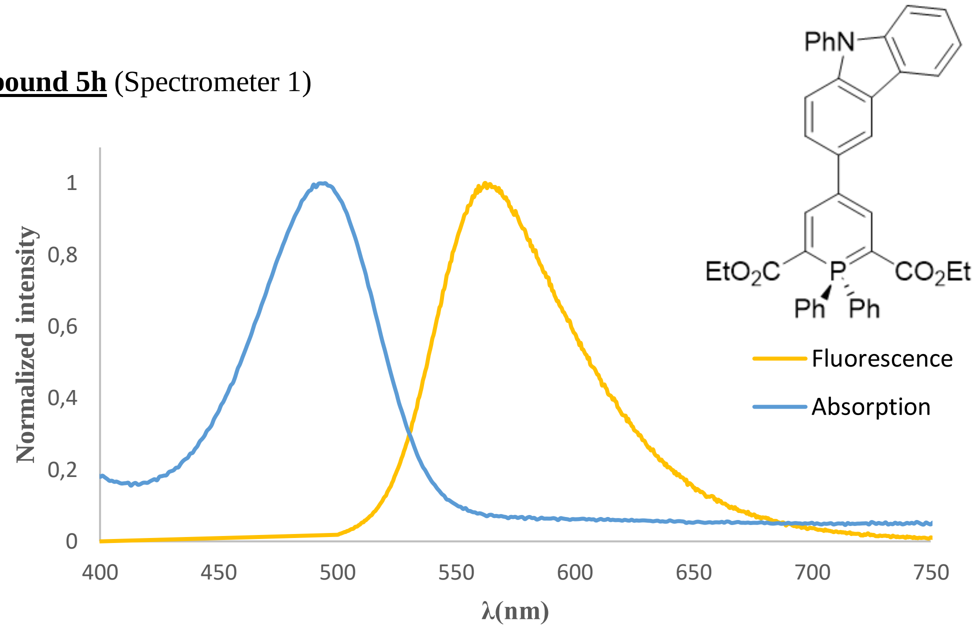

$\lambda_{abs,max}$/nm = 495

$\lambda_{em,max}$/nm = 563

Compound 5i (Spectrometer 1)

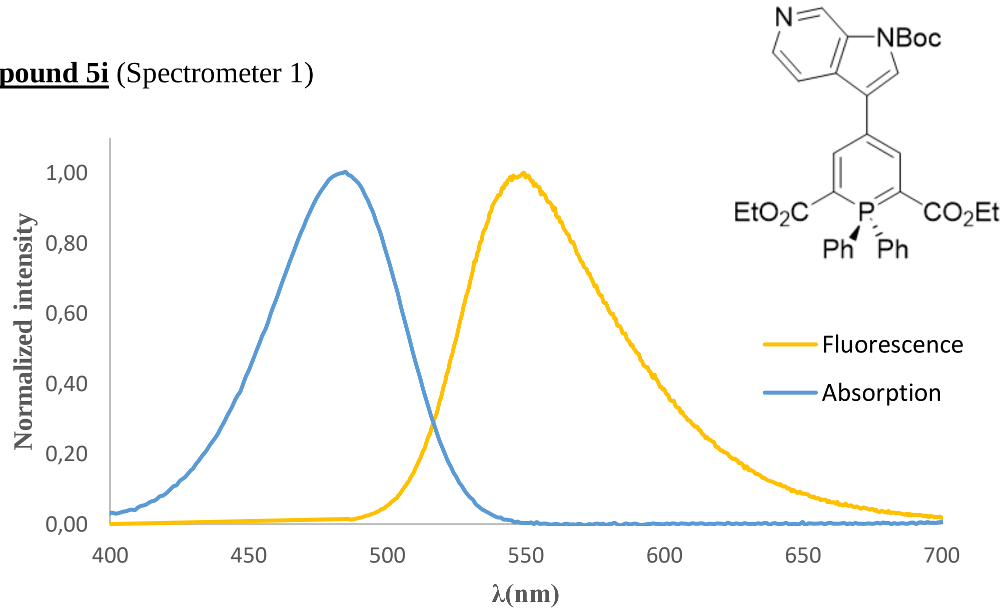

$\lambda_{abs,max}$/nm = 484

$\lambda_{em,max}$/nm = 548

Compound 7 (Spectrometer 1)

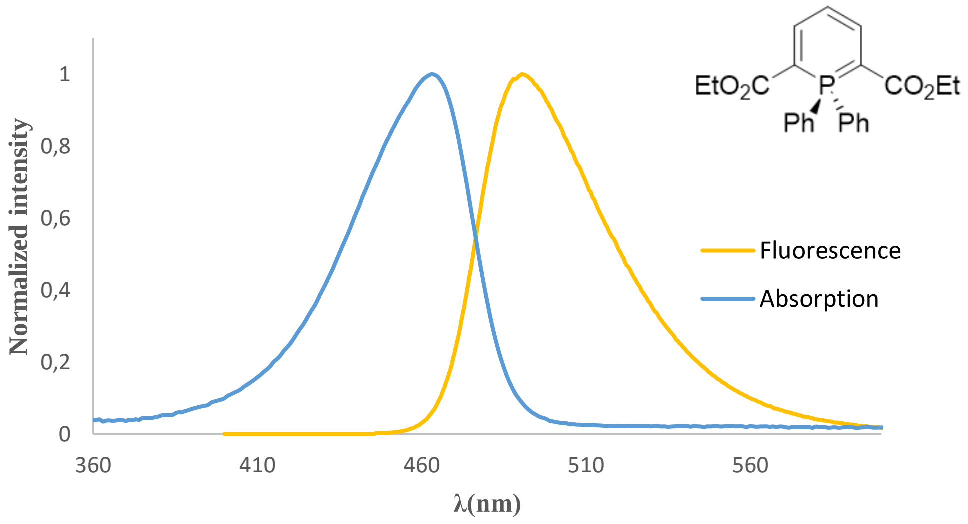

$\lambda_{abs,max}$/nm = 463

$\lambda_{em,max}$/nm = 490

Compound 3r (Spectrometer 2)

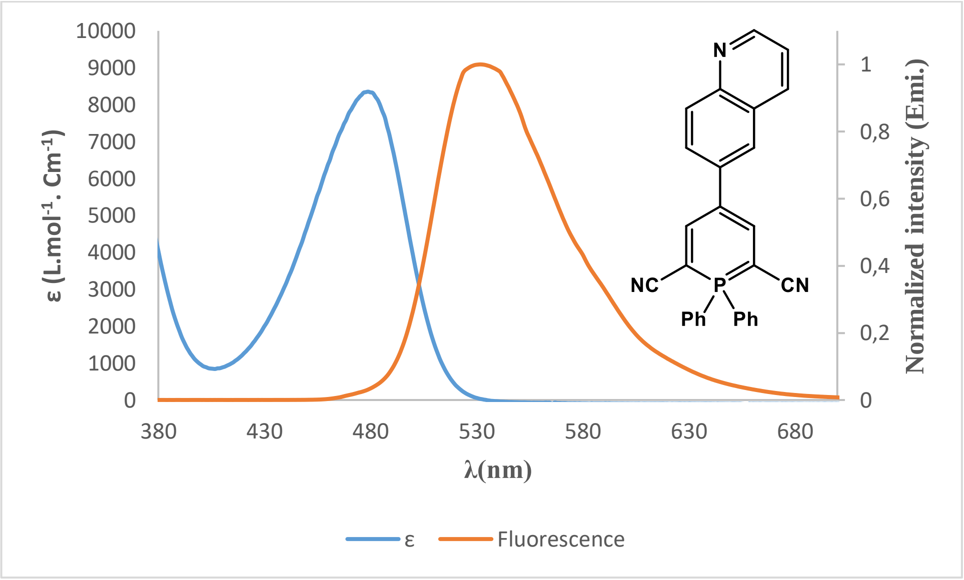

$\lambda_{abs,max}$/nm = 479

$\lambda_{em,max}$/nm = 532

A_{max} = 0.835178

ε = 8351.78 L.mol^{-1}.cm^{-1} Stokes-shifts= 2080 (cm^{-1}) or 53 (nm)

Compound 2h (Spectrometer 2)

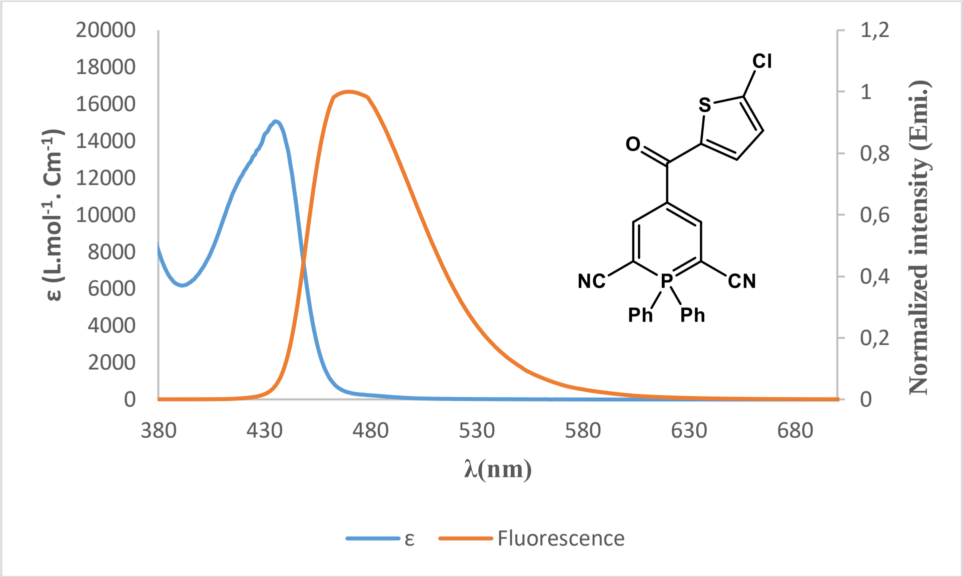

$\lambda_{abs,max}$/nm = 435

$\lambda_{em,max}$/nm = 470

A_{max} = 1.506429

ε = 15064.29 L.mol^{-1}.cm^{-1} Stokes-shifts= 1712 (cm^{-1}) or 35 (nm)

Compound 2g (Spectrometer 2)

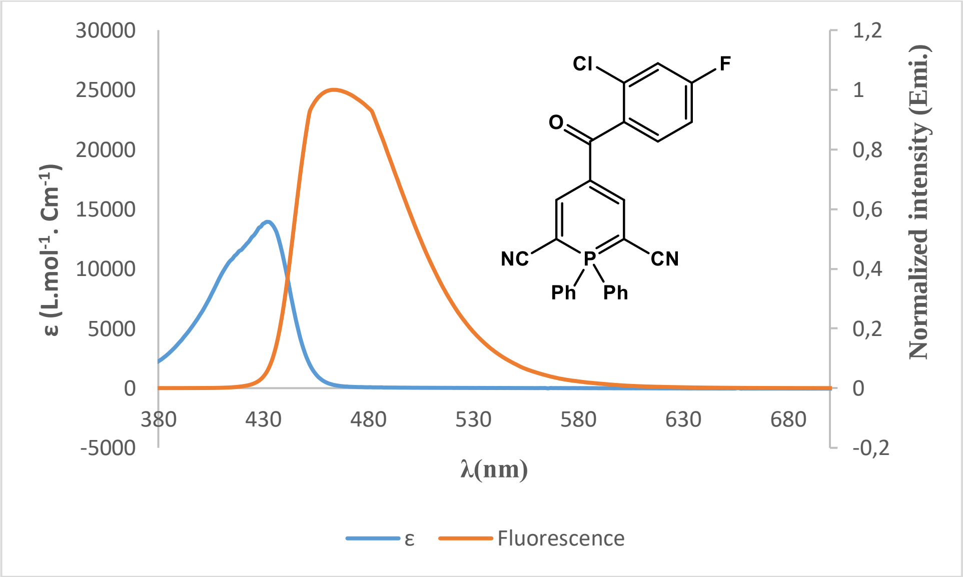

$\lambda_{abs,max}$/nm = 432

$\lambda_{em,max}$/nm = ca. 464

A_{max} = 1.393526

ε = 13935.26 L.mol^{-1}.cm^{-1} Stokes-shifts= 1596 (cm^{-1}) or 32 (nm)

Compound 3d (Spectrometer 2)

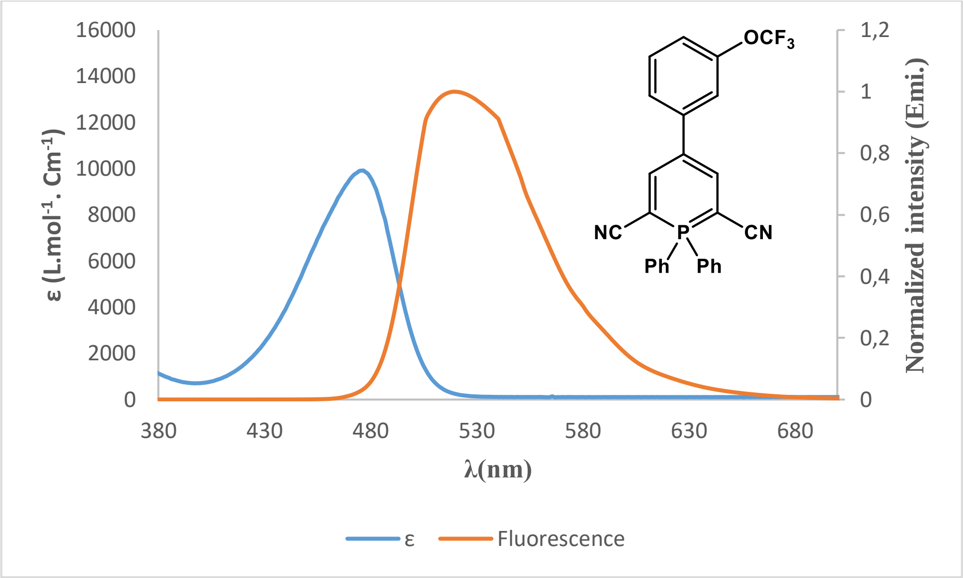

$\lambda_{abs,max}$/nm = 477

$\lambda_{em,max}$/nm = ca. 520

A_{max} = 0.990009

ε = 9900.09 L.mol^{-1}.cm^{-1} Stokes-shifts= 1734 (cm^{-1}) or 43 (nm)

Compound 3l (Spectrometer 2)

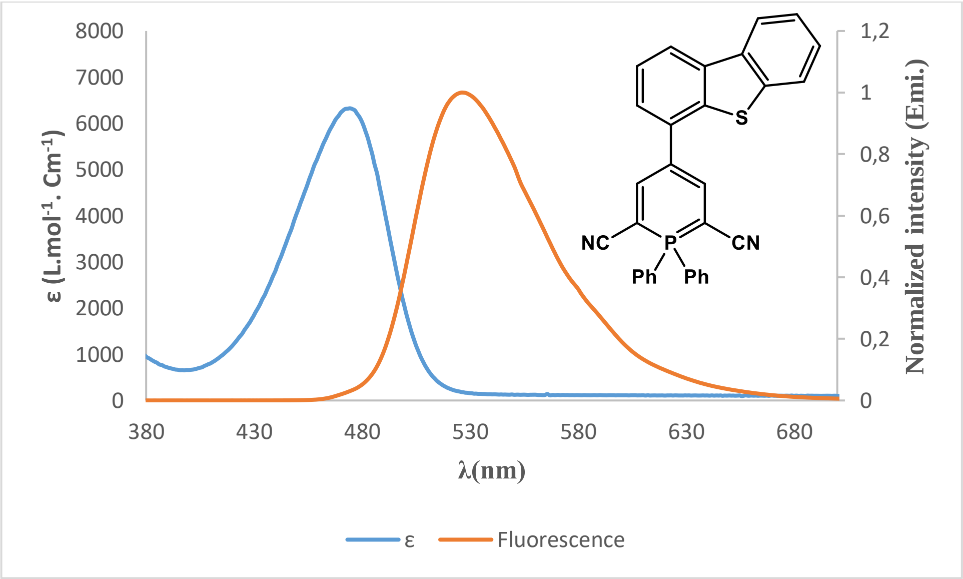

$\lambda_{abs,max}$/nm = 475

$\lambda_{em,max}$/nm = 526

A_{max} = 0.632545

ε = 6325.45 L.mol^{-1}.cm^{-1} Stokes-shifts= 2041 (cm^{-1}) or 51 (nm)

Compound 2a (Spectrometer 2)

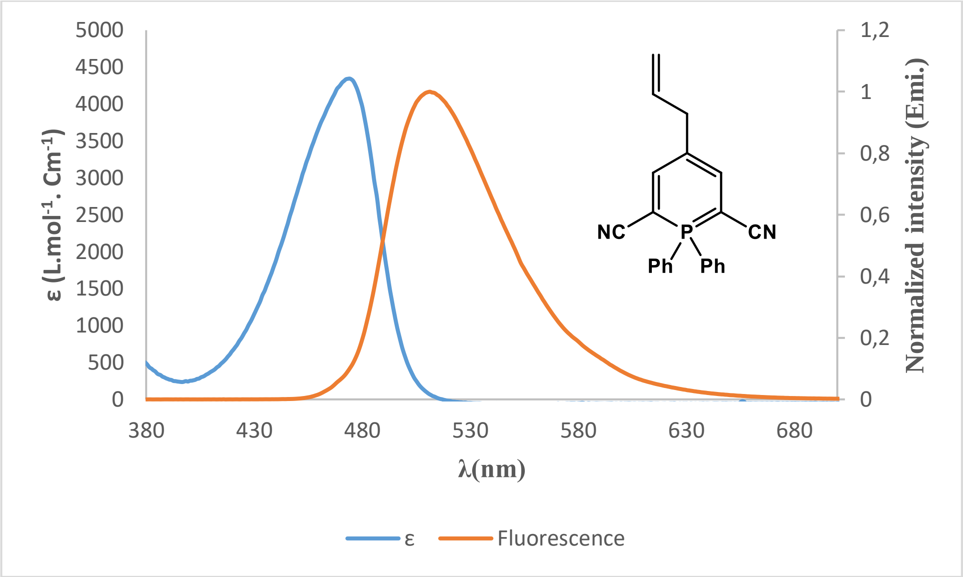

$\lambda_{abs,max}$/nm = 474

$\lambda_{em,max}$/nm = 512

A_{max} = 0.434507

ε = 4345.07 L.mol^{-1}.cm^{-1} Stokes-shifts= 1566 (cm^{-1}) or 38 (nm)

Compound 3s (Spectrometer 2)

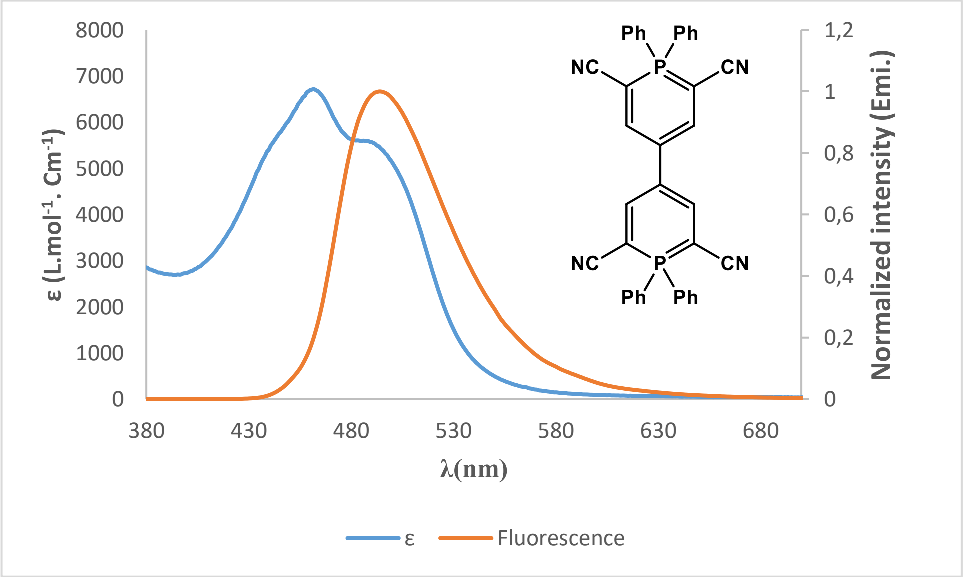

$\lambda_{abs,max}$/nm = 462

$\lambda_{em,max}$/nm = 494

A_{max} = 0.671528

ε = 6715.28 L.mol^{-1}.cm^{-1} Stokes-shifts= 1402 (cm^{-1}) or 32 (nm)

Compound 5a (Spectrometer 2)

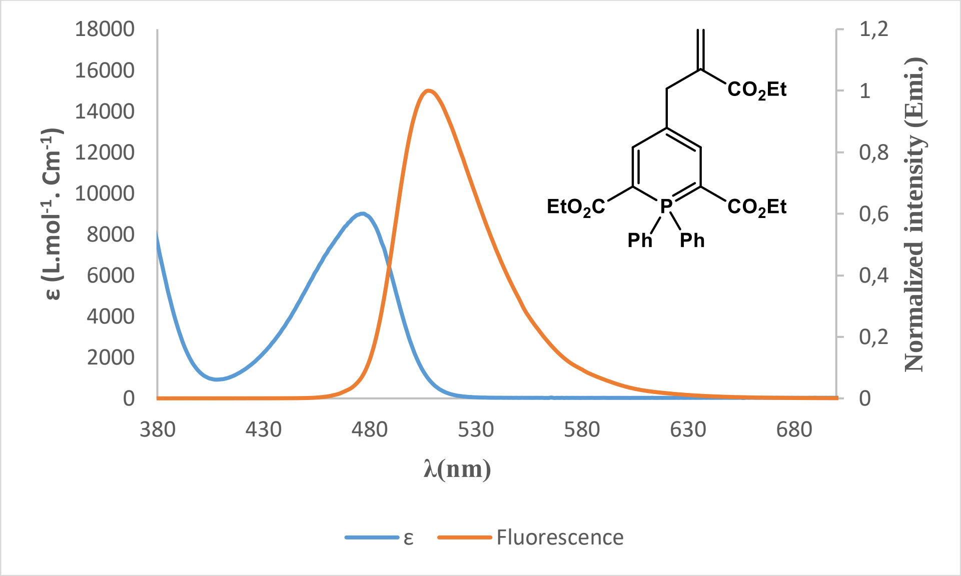

$\lambda_{abs,max}$/nm = 477

$\lambda_{em,max}$/nm = 508

A_{max} = 0.90059

ε = 9005.9 $L.mol^{-1}.cm^{-1}$ Stokes-shifts= 1279 (cm^{-1}) or 31 (nm)

Compound 3f (Spectrometer 2)

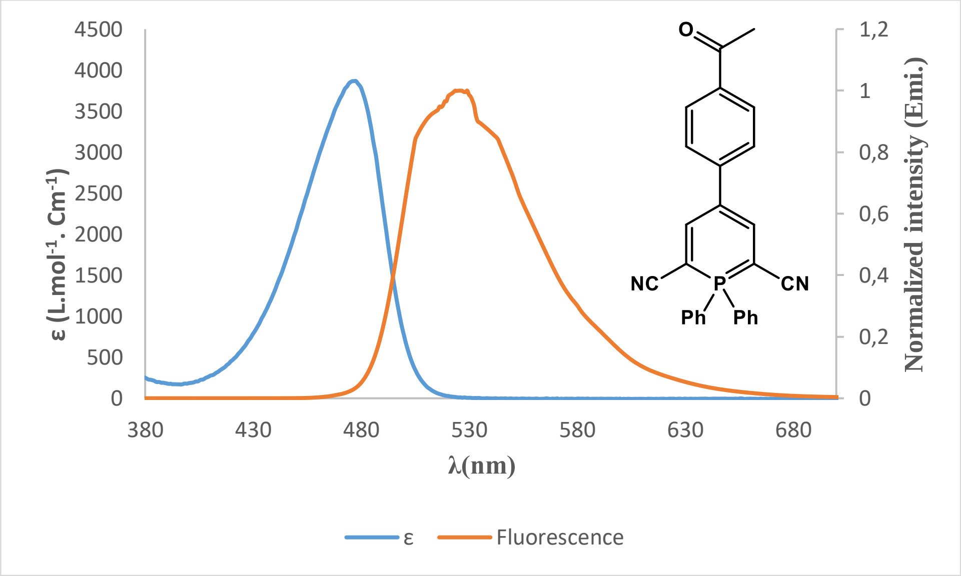

$\lambda_{abs,max}$/nm = 477

$\lambda_{em,max}$/nm = ca. 526

A_{max} = 0.386856

ε = 3868.56 $L.mol^{-1}.cm^{-1}$ Stokes-shifts= 1953 (cm^{-1}) or 49 (nm)

Compound 3x (Spectrometer 2)

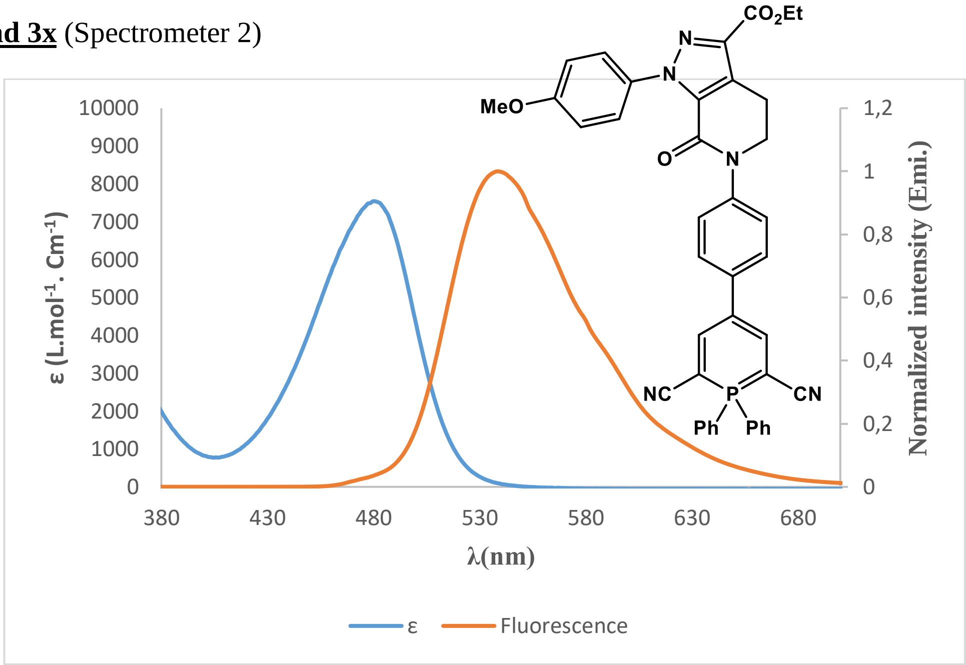

$\lambda_{abs,max}$/nm = 480

$\lambda_{em,max}$/nm = 539

A_{max} = 0.754348

ε = 7543.48 L.mol^{-1}.cm^{-1} Stokes-shifts= 2280 (cm^{-1}) or 59 (nm)

Compound 3j (Spectrometer 2)

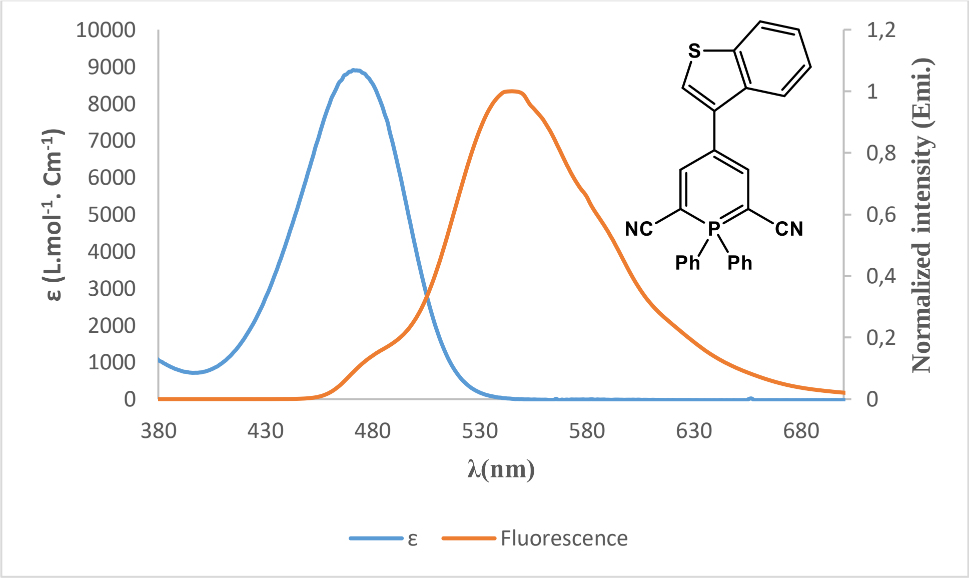

$\lambda_{abs,max}$/nm = 473

$\lambda_{em,max}$/nm = 546

A_{max} = 0.889501

ε = 8895.01 L.mol^{-1}.cm^{-1} Stokes-shifts= 2827 (cm^{-1}) or 73 (nm)

Compound 3n (Spectrometer 2)

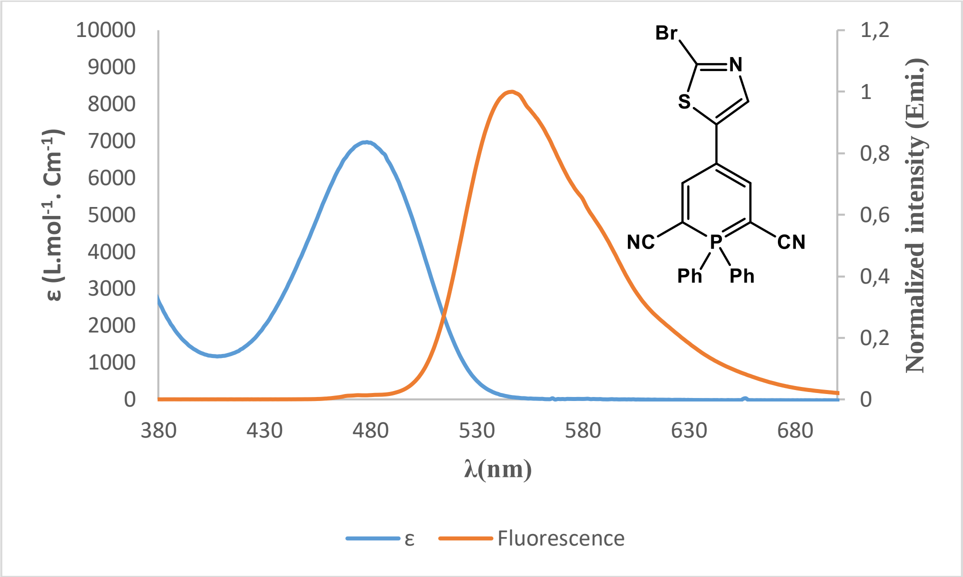

$\lambda_{abs,max}$/nm = 478

$\lambda_{em,max}$/nm = 547

A_{max} = 0.696896

ε = 6968.96 L.mol^{-1}.cm^{-1} Stokes-shifts= 2639 (cm^{-1}) or 69 (nm)

Compound 3o (Spectrometer 2)

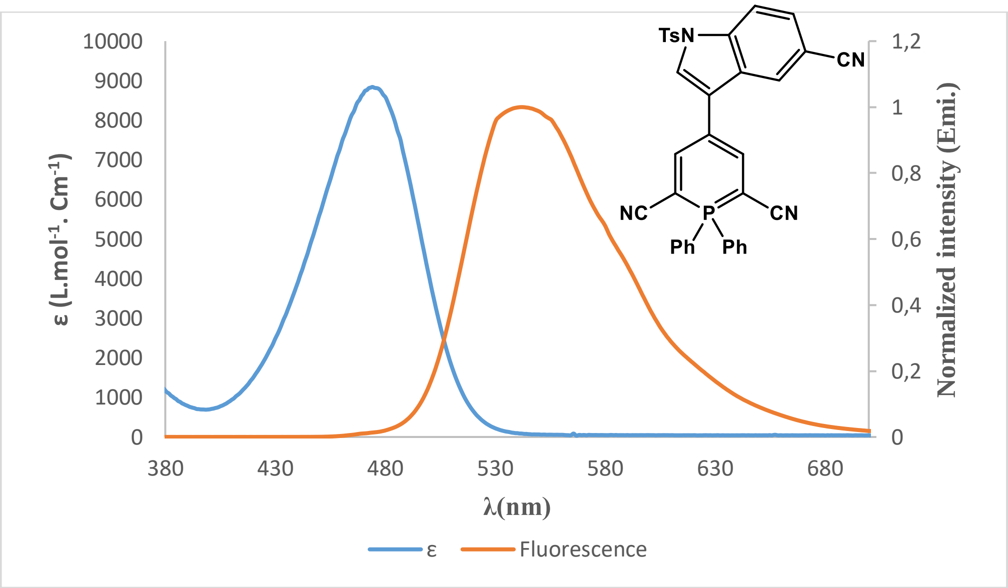

$\lambda_{abs,max}$/nm = 474

$\lambda_{em,max}$/nm = 542

A_{max} = 0.884101

ε = 8841.01 L.mol^{-1}.cm^{-1} Stokes-shifts= 2647 (cm^{-1}) or 68 (nm)

Absorption

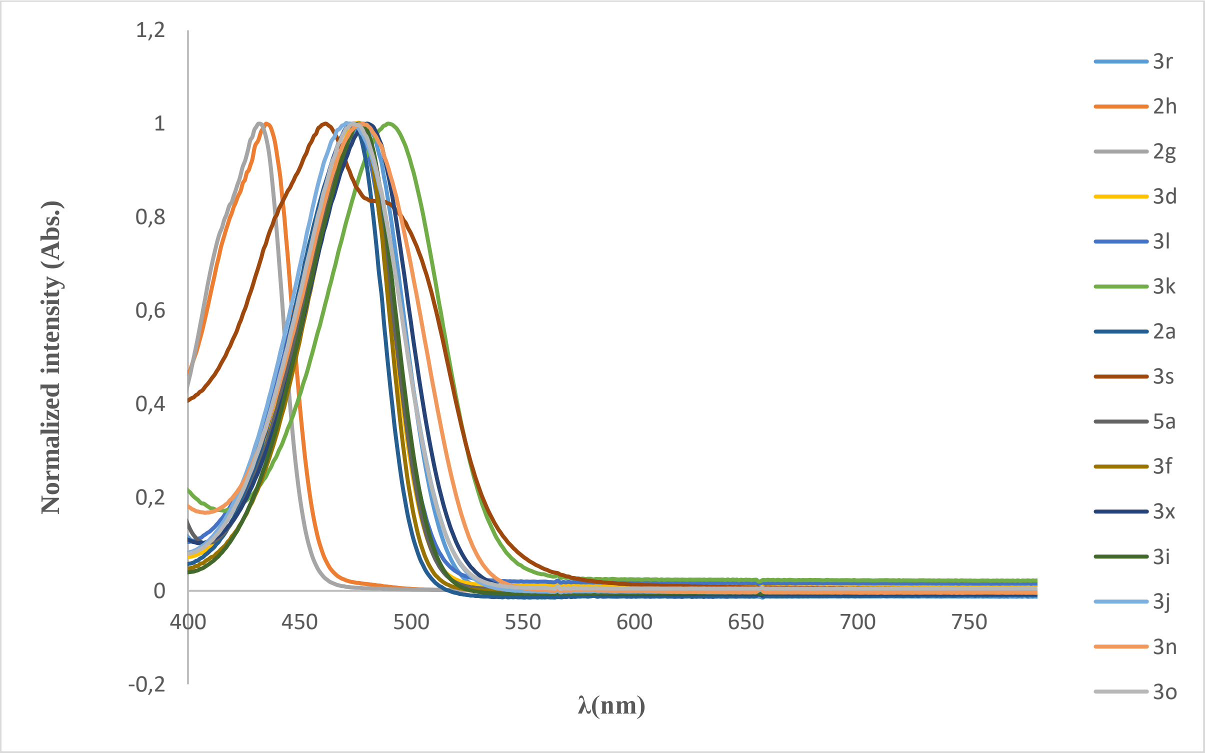

Emission (Fluorescence)

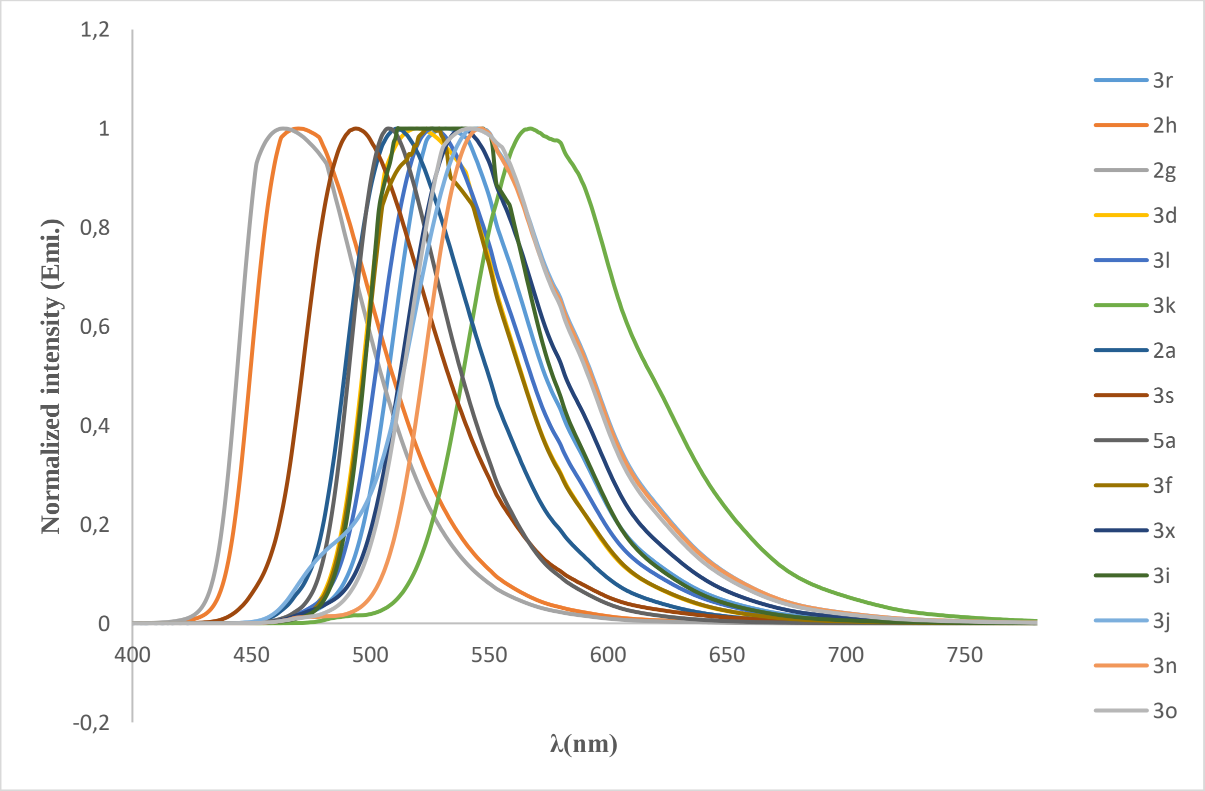

Supplement: Supplementary file 4 — Supplementary Data 2 [file 42004_2025_1822_MOESM4_ESM.pdf]
